# Chemoselective α,β‐Dehydrogenation of Saturated Amides

**DOI:** 10.1002/anie.201808794

**Published:** 2018-12-07

**Authors:** Christopher J. Teskey, Pauline Adler, Carlos R. Gonçalves, Nuno Maulide

**Affiliations:** ^1^ University of Vienna Institute of Organic Chemistry Währinger Strasse 38 1090 Vienna Austria

**Keywords:** amide activation, dehydrogenation, hypervalent iodine, seleninic acid, triflic anhydride

## Abstract

We report a method for the selective α,β‐dehydrogenation of amides in the presence of other carbonyl moieties under mild conditions. Our strategy relies on electrophilic activation coupled to in situ selective selenium‐mediated dehydrogenation. The α,β‐unsaturated products were obtained in moderate to excellent yields, and their synthetic versatility was demonstrated by a range of transformations. Mechanistic experiments suggest formation of an electrophilic Se^IV^ species.

More than 130 years have passed since Arthur Michael reported his groundbreaking studies on the conjugate addition of malonate nucleophiles.^1^ In the intervening time, much work has been devoted to the development of asymmetric variants of this reaction, which has become a staple transformation of the organic chemist's repertoire.^2^ Given the versatility of the carbonyl functional group, the Michael addition of an enolate to an unsaturated carbonyl group is one of the definitive methods for generating a 1,5‐dicarbonyl relationship, and as a consequence, such transformations can be frequently found in total syntheses.^3^

The mild conditions of conjugate addition, as the Michael reaction has come to be colloquially known, also lend this reaction well to other areas of research such as bioconjugation or dynamic combinatorial chemistry.^4^ As a result, methods to synthesize α,β‐unsaturated systems are highly prized. Convenient and efficient pathways include carbonyl olefination^5^ and olefin cross‐metathesis,^6^ although the ability to directly generate α,β‐unsaturated carbonyl compounds from their corresponding saturated counterparts by dehydrogenation adds considerable flexibility to synthetic planning. Pioneering work by Kingsbury and Cram on the concerted thermal elimination of alkyl sulfoxides^7^ later inspired similar work on selenoxide elimination.^8^ The Saegusa–Ito oxidation^9^ is another well‐known and often employed route to dehydrogenated carbonyl systems but is a two‐step procedure. Despite the original use of stoichiometric palladium, notable efforts have been made to develop efficient catalytic reactions based on much greener oxidants by the groups of Stahl^10^ and others.^11^ Although reliable, these methods often involve two steps or are limited to (cyclic) ketones. Other important contributions include the work of Nicolaou and co‐workers, who reported the use of 2‐iodoxybenzoic acid (IBX) to oxidise ketones to the corresponding α,β‐enones.^12^

When considering the direct dehydrogenation of carbonyl compounds beyond ketones and aldehydes, a major obstacle is the significantly reduced α‐acidity of esters, nitriles, or particularly carboxamides. Newhouse and co‐workers published a number of impressive reports showcasing how zinc enolates (generated by transmetalation from the corresponding lithium counterparts), including those derived from amides, can be dehydrogenated under palladium catalysis^13^ (Scheme 1 a).^14^ Furthermore, these authors have demonstrated this methodology to be applicable in total synthesis.^15^

**Scheme 1:**
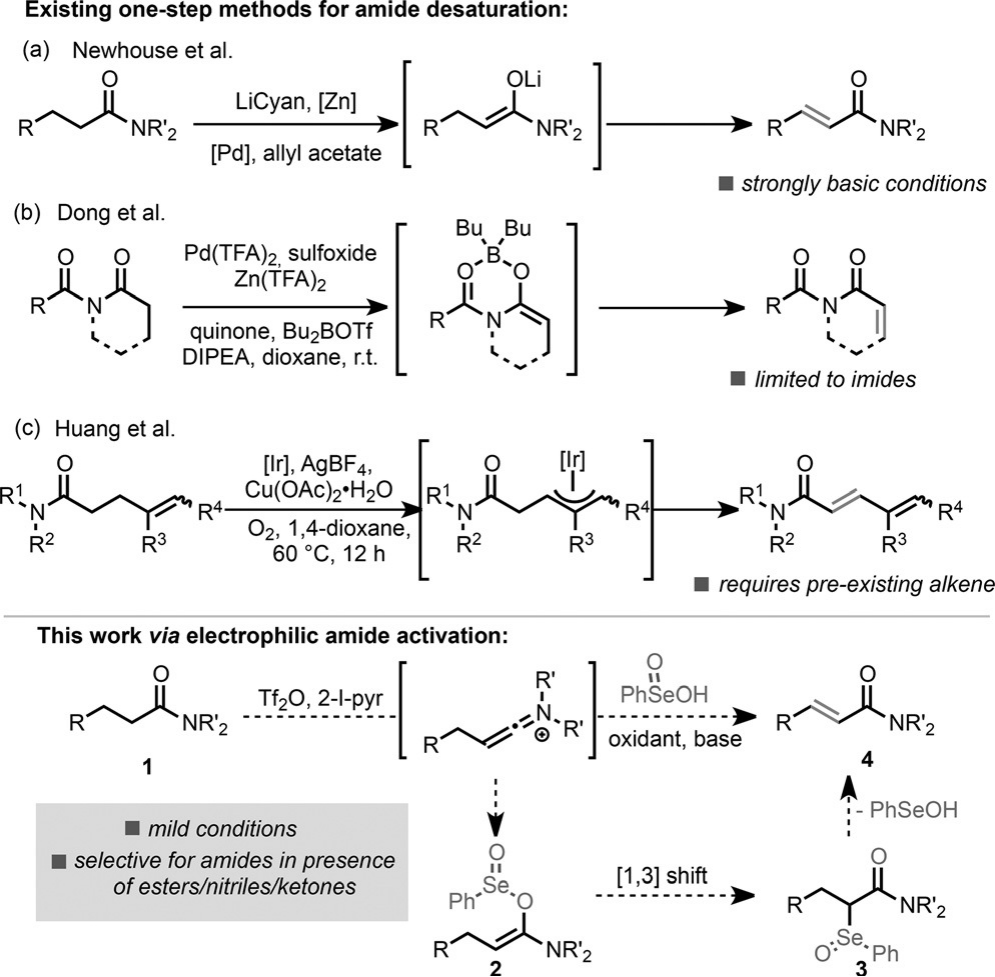
Approaches to amide dehydrogenation and work presented herein. DIPEA=diisopropylethylamine, 2‐I‐pyr=2‐iodopyridine, LiCyan=lithium *N*‐cyclohexyl anilide, TFA=trifluoroacetate, Tf_2_O=triflic anhydride.

Dong and Chen used a related strategy to desaturate *N*‐acyl lactams,^16^ relying on two‐point binding activation (Scheme 1 b), and most recently, the group of Huang showed that iridium catalysis could be used for desaturation in the specific case of γ,δ‐unsaturated amides (Scheme 1 c).^17^

We believed that there remained space for complementary methods that proceed under conditions tolerant of other carbonyl functional groups and that are applicable to structurally diverse amides. We envisaged the use of electrophilic amide activation^18^ owing to the possibilities it might afford in developing a strategy for chemoselective desaturation of amides in the presence of other carbonyl functional groups.^19^ Herein, we report a mild, room‐temperature α,β‐dehydrogenation of amides with broad applicability that is selective for amides, thus reaching beyond the scope of most current procedures as it does not rely on enolate formation. Our reaction design was based on the simple assumption that seleninic acid (PhSe(O)OH) might be nucleophilic enough to attack a keteniminium intermediate as shown in Scheme 1. This might lead to an enamine‐type species such as **2**, which could undergo an unusual [1,3]‐sigmatropic rearrangement.^20^ The resulting α‐selenated species **3** should already be in the oxidation state required for concerted elimination, ultimately affording the α,β‐dehydrogenated product in a single step.

In initial efforts using amide **1 a**, the desired dehydrogenated product **4 a** was already observed, albeit in yields never surpassing 50 % (Table 1, entry 1; see the Supporting Information for additional experiments). Indeed, the crude reaction mixture commonly contained variable amounts of both α‐hydroxylated amide **5** and α‐selenated amide **6**.

**Table 1:** Optimization of the reaction. 
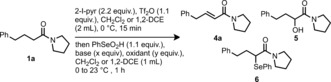

Entry	[Se]	Base	Oxidant	Yield [%]^[a]^			
	(equiv)	(equiv)	(equiv)	**4 a**	**5**	**6**	**1 a**
1	PhSeO_2_H (1)	Ag_2_CO_3_ (1.1)	–	48	20	–	41
2	PhSeO_2_H (1)	Et_3_N (1.1)	–	–	24	50	14
3	PhSeO_2_H (1)	Et_3_N (2.2)	PIDA (2.2)	22	12	–	25
4	PhSeO_2_H (1)	Et_3_N (2.2)	IBX (2.2)	29	45	–	21
**5**	**PhSeO_2_H (1)**	**Et_3_N (2.2)**	**DMP (2.2)**	**73**	**–**	**–**	**10**
6	–	Et_3_N (2.2)	DMP (2.2)	8	2	–	50

[a] Yields determined by ^1^H NMR analysis with bromoform as an internal standard. 1,2‐DCE=1,2‐dichloroethane, PIDA=iodobenzene diacetate.

In subsequent experiments, we noted that the use of triethylamine as a base (Table 1, entry 2) yielded none of the desaturated product (**4 a**) and 50 % of the α‐selenated amide **6** instead. This prompted us to try the same reaction with the addition of oxidants (see the Supporting Information for more details), with hypervalent iodine species affording the most promising results (Table 1, entries 3–6). With 2.2 equivalents of Dess–Martin periodinane (DMP), the α,β‐dehydrogenated product was reproducibly formed in 73 % NMR yield. This procedure avoided the formation of several other products, thus simplifying the purification process although the yields of isolated products were, in most cases, lower than those measured by NMR analysis owing to difficulties in separating the residual starting materials of nearly identical polarity.

With optimized reaction conditions in hand, our attention next turned to exploring the scope of the reaction (Scheme 2). Pleasingly, a broad range of tertiary amides were tolerated by this desaturation protocol. β‐Substitution with a cyclopentyl chain allowed the formation of **4 b**. Different cinnamyl amides could be prepared by this method (**4 c**–**e**). Notably, α‐substituted amides could also be dehydrogenated, and indene **4 f** and cyclobutene amide **4 g** were isolated in good yields. The 1,3‐diene **4 h** was also prepared in moderate yield. The conditions of the reaction tolerated the use of alkynes (**4 i**) and trifluoromethyl groups (**4 j**). Tertiary amides with other N‐substituents, including removable dibenzyl groups (**4 k**), morpholine (**4 l**), piperidine (**4 m**), or azepine (**4 n**), efficiently gave the corresponding desaturated amides. Aniline‐derived amides were also well tolerated, delivering the products in good yields (**4 o** and **4 p**).

**Scheme 2:**
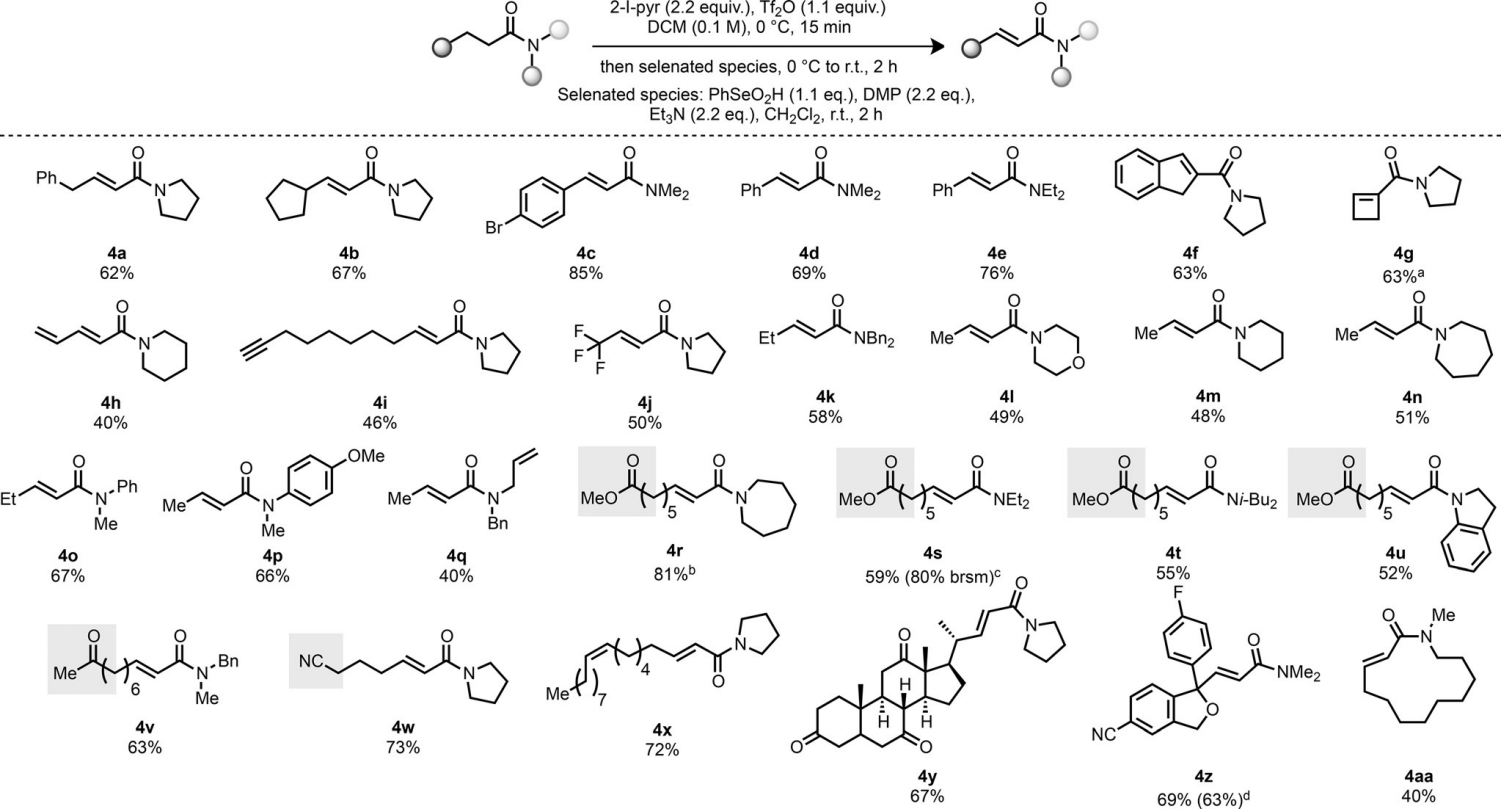
Scope of amide dehydrogenation. [a] On 1 mmol scale. [b] Reaction carried out at −20 °C. [c] Reaction carried out at −10 °C. [d] On 4.2 mmol scale. DCM=dichloromethane.

A unique feature of this α,β‐dehydrogenation reaction is its high chemoselectivity. Amides can be selectively desaturated even in the presence of commonly more reactive carbonyl functional groups, such as esters and ketones, in moderate to good yields. To the best of our knowledge, no method exists that allows this type of selectivity. Methyl esters were effectively spectators in the α,β‐dehydrogenation of different tertiary amides **4 r**–**4 u**. Furthermore, the presence of a ketone (**4 v**) or a nitrile (**4 w**) functional group did not divert the reaction away from the amide moiety. Amides derived from natural product feedstocks such as oleic acid (**4 x**) or dehydrocholic acid (**4 y**) could also be selectively desaturated in good isolated yields. An amide analogue of the blockbuster drug citalopram was also successfully dehydrogenated to afford product **4 z**. Finally, we subjected a 13‐membered lactam to our procedure and pleasingly obtained the desaturated product **4 aa** in moderate yield.

In order to further probe the selectivity of the reaction, we subjected a substrate containing both a secondary and a tertiary amide to the reaction conditions. Pleasingly, product **4 ab**, resulting from desaturation adjacent to the tertiary amide, was exclusively obtained (Scheme 3 a). We next sought to showcase the utility of these Michael acceptor products. Dihydroxylation proceeded smoothly to give product **7** in 69 % (Scheme 3 b).^21^ Asymmetric conjugate addition to unsaturated amides, as recently described by Harutyunyan and co‐workers,^22^ was realized on unsaturated amide **4 n** with EtMgBr, resulting in product **8** in 91 % yield and 96 % *ee* (Scheme 3 c). Cognizant of the pervasiveness of amides in nature,^23^ we were keen to apply our method to the synthesis of a natural product. We selected piperine (**13**; Scheme 3 d), a polyunsaturated amide responsible for the pungency of pepper. Piperine possesses antioxidant, anti‐inflammatory, and antidepressant properties and has been synthesized previously by a variety of strategies.^24^ Here, we propose an alternative three‐step synthesis starting from the commercially available aldehyde **9** (Scheme 3 c). After quantitative transformation of **9** into the allylic alcohol **10**, this compound was treated with the in situ prepared 1,1‐diamino alkene **11** in refluxing xylene to yield the γ,δ‐unsaturated amide **12** in a very good 73 % yield by a modified Claisen–Eschenmoser rearrangement.^25^ Further dehydrogenation of **12** delivered piperine (**13**) in moderate 42 % yield.

**Scheme 3:**
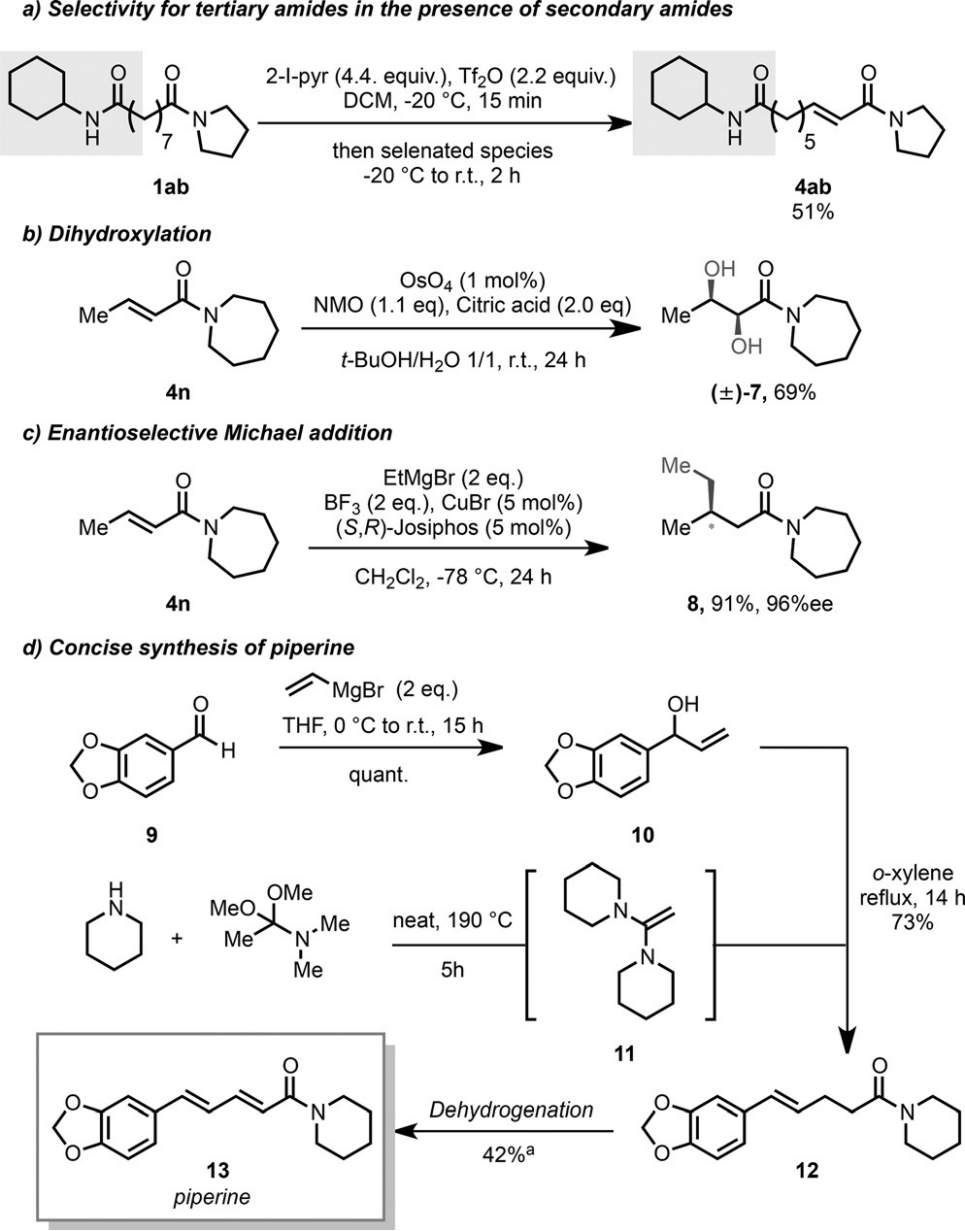
Derivatisation of products and application of the method. [a] NMR yield. NMO=*N*‐methylmorpholine *N*‐oxide.

We then turned our attention to the mechanism of the reaction. Preliminary work had demonstrated the formation of the α‐selenated amide **6** in 64 % yield by addition of selenenic acid, PhSeOH, onto a keteniminium ion (Scheme 4 a). We hypothesized that this occurred by a [1,3]‐sigmatropic rearrangement from intermediate **2′**. During this reaction, α‐hydroxylated product **5** was also formed in 25 %, possibly by nucleophilic attack of PhSeOH on the α‐position of intermediate **2′** followed by hydrolysis. These results led us to postulate pathway (i) (Scheme 4) for the dehydrogenation mechanism by simple analogy, the only difference being the oxidation state of the selenium atom. However, during the reaction discovery process, it was observed that in the absence of an oxidant, the α‐selenated amide **6** remained the major product (Table 1, entry 2). Given that pathway (i) generates PhSeOH (**18**), we believe it possible that DMP could play two distinct roles: 1) trapping of **18**, which is likely more nucleophilic than seleninic acid, and 2) oxidation of any **6** that is produced. In support of this hypothesis, we observed that DMP was able to oxidise α‐selenated amide **6** to desaturated amide **4 a** in moderate yield (Scheme 4 c).

**Scheme 4:**
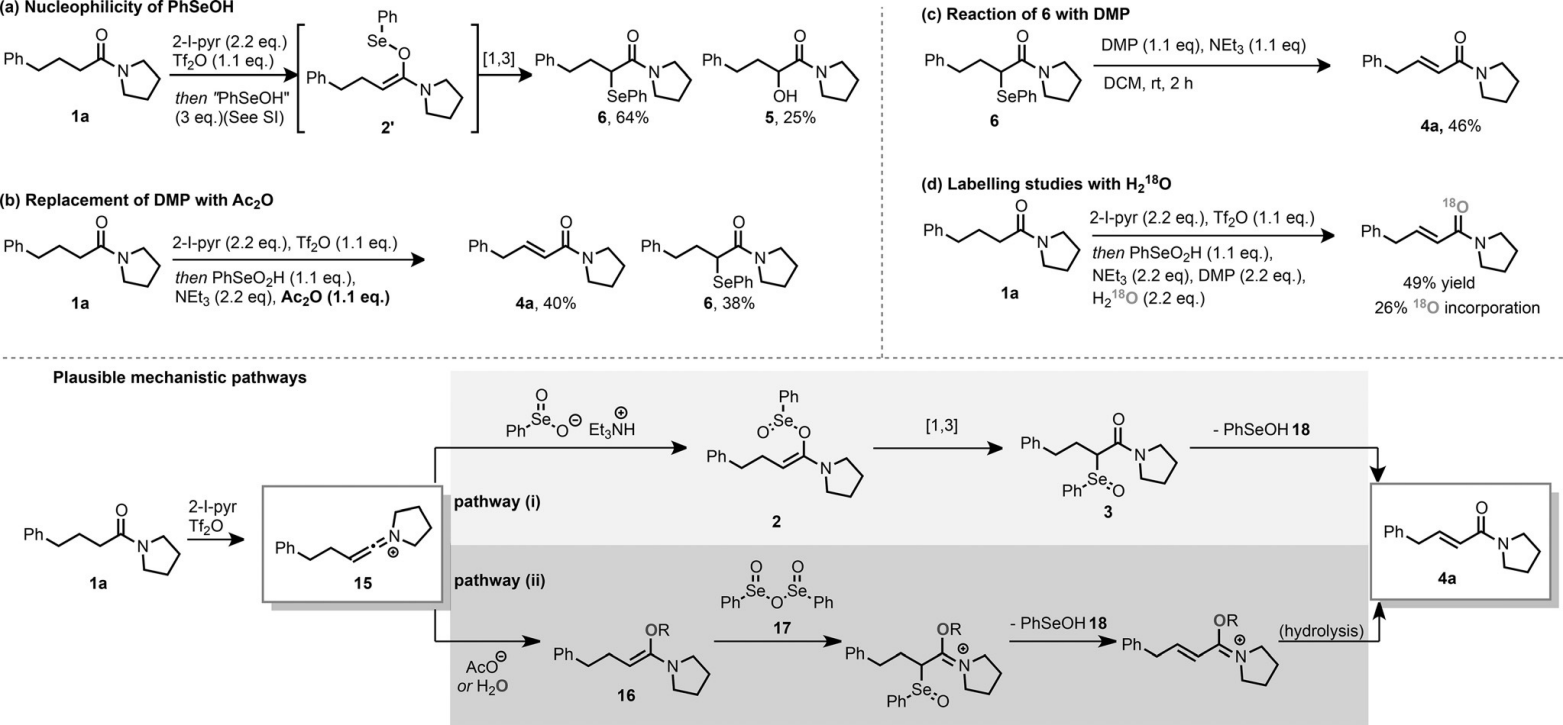
Mechanistic experiments.

Alternatively, because it has been demonstrated that iodylbenzoic acid can be used as an oxidant to generate benzene seleninic anhydride (BSA) in situ from diphenyl diselenide (PhSeSePh),^26^ we believe that we may also be generating BSA (**17**) under our reaction conditions.^27^

From the labelling studies we undertook with H_2_^18^O (Scheme 4 d), we established that keteniminium species **15** is likely attacked by either water or acetate released by the DMP, to give the enamine/enol (acetate) hybrid **16**.^28^ Conceivably, reaction of **16** with **17** would generate an intermediate that leads to product **4 a** (Scheme 4, pathway (ii)). Upon replacing DMP with acetic anhydride (Scheme 4 b), we generated a mixture of **6** and **4 a**. This further suggests that an electrophilic, oxidized selenium species (likely the mixed anhydride) plays some role in the reaction, but also emphasizes that DMP is important, either to oxidise **6** to **4 a**, or to trap and re‐oxidise species **18** and lower‐oxidation‐state selenium species.^29^ We speculate that the modest efficiency of the reaction in Scheme 4 c indicates the major role of the DMP to be a secondary oxidant of species **18**, which avoids side reactions of the keteniminium intermediate **15** with lower‐oxidation‐state selenium species.

In conclusion, we have reported a novel method for the synthesis of α,β‐unsaturated amides from the corresponding saturated amide starting materials. The reaction proceeds by electrophilic activation followed by a unique selenium‐mediated dehydrogenation. This process conveys good functional group tolerance and, significantly, enables the desaturation of amides in the presence of esters, ketones, and nitriles. We also applied this method to the synthesis of the natural product piperine. Current experiments allude to a mechanism that involves attack onto an electrophilic Se^IV^ species. Further investigations in this direction are underway in our laboratory and will be reported in due course.

## Conflict of interest

The authors declare no conflict of interest.

## Supporting information

As a service to our authors and readers, this journal provides supporting information supplied by the authors. Such materials are peer reviewed and may be re‐organized for online delivery, but are not copy‐edited or typeset. Technical support issues arising from supporting information (other than missing files) should be addressed to the authors.

Supplementary
