# Supplementary material for: Chemoselective α,β‐Dehydrogenation of Saturated Amides
Source: Angew Chem Int Ed Engl. 2018 Dec 7;58(2):447–51. doi: 10.1002/anie.201808794 (PMC6348382; doi:10.1002/anie.201808794)
Supplement: Supplementary file 1 — Supplementary [file ANIE-58-447-s001.pdf]

Angewandte Chemie
Eine Zeitschrift der Gesellschaft Deutscher Chemiker

Supporting Information

Chemoselective α,β-Dehydrogenation of Saturated Amides

*Christopher J. Teskey[+], Pauline Adler[+], Carlos R. Gonçalves, and Nuno Maulide**

anie_201808794_sm_miscellaneous_information.pdf

Table of Contents

1. **General Information** **2**
2. **Optimization** **3**
3. **Amides** **6**
   - 3.1. Synthesis of Amides 6
   - 3.2. Characterization 7
4. **Products of desaturation** **17**
   - 4.1. General Procedure C 17
   - 4.2. Characterization 18
   - 4.3. Application of our methodology 32
     - 4.3.1. Dihydroxylation 32
     - 4.3.2. Enantioselective Michael addition 32
     - 4.3.3. Synthesis of piperine 35
5. **Labelling experiments** **37**
6. **References** **38**
7. **Spectra** **39**

1. General Information

Unless otherwise stated, all glassware was flame-dried before use and all reactions were performed under an atmosphere of argon. All solvents were distilled from appropriate drying agents prior to use. Triflic anhydride was distilled over P_4O_{10} prior to use. All other reagents were used as received from commercial suppliers unless otherwise stated. Reaction progress was monitored by thin layer chromatography (TLC) performed on aluminium plates coated with silica gel F_{254} with 0.2 mm thickness. Chromatograms were visualized by fluorescence quenching with UV light at 254 nm or by staining using potassium permanganate or phosphomolybdic acid. Flash column chromatography was performed using silica gel 60 (230-400 mesh, Merck and co.). Neat infra-red spectra were recorded using a Perkin-Elmer Spectrum 100 FT-IR spectrometer. Wavenumbers ($\mathbf{v_{max}}$) are reported in cm^{-1}. Mass spectra were obtained using a Finnigan MAT 8200 or (70 eV) or an Agilent 5973 (70 eV) spectrometer, using electrospray ionization (ESI). All ^{1}H NMR, ^{13}C NMR and ^{77}Se NMR spectra were recorded using a Bruker AV-400 or AV-600 spectrometer at 300K. Chemical shifts were given in parts per million (ppm, δ), referenced to the solvent peak of $CDCl_3$, defined at δ = 7.26 ppm (^{1}H NMR) and δ = 77.16 (^{13}C NMR). Coupling constants are quoted in Hz (*J*). ^{1}H NMR splitting patterns were designated as singlet (s), doublet (d), triplet (t), quartet (q), pentet (p). Splitting patterns that could not be interpreted or easily visualized were designated as multiplet (m) or broad (br). Selected ^{13}C NMR spectra were recorded using the attached proton test (APT) to facilitate the confirmation and assignment of the structure.

2. Optimization

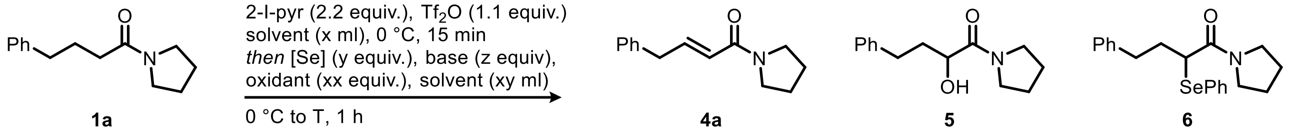

Entry	Solvent 1	[Se]	Base	Oxidant/Add.	Solvent 2	T (°C)	1a[a]	4a[a]	5[a]	6[a]
**1**	DCE (2 mL)	$PhSeO_2H$ (2 eq.)	Ag_2CO_3 (2.2 eq.)	-	DCE (1 mL)	r.t.	15	40	15	ND
**2**	DCE (2 mL)	$PhSeO_2H$ (1 eq.)	Ag_2CO_3 (1.1 eq.)	-	DCE (1 mL)	r.t.	15	48	20	ND
**3**	DCE (2 mL)	$PhSeO_2H$ (2 eq.)	Cs_2CO_3 (2,2 eq.)	-	DCE (1 mL)	r.t.	30	20	0	62
**4**	DCE (2 mL)	$PhSeO_2H$ (2 eq.)	$CaCO_3$ (2,2 eq.)	-	DCE (1 mL)	r.t.	38	0	29	0
**5**	DCE (2 mL)	$PhSeO_2H$ (2 eq.)	Ag_2O (2,2 eq.)	-	DCE (1 mL)	r.t.	30	44	0	27
**6**	DCE (2 mL)	$PhSeO_2H$ (2 eq.)	AgF (2,2 eq.)	-	DCE (1 mL)	r.t.	25	27	26	19
**7**	DCE (2 mL)	$PhSeO_2H$ (1 eq.)	Proton sponge (1,1 eq.)	-	DCE (1 mL)	r.t.	16	traces	0	28
**8**	DCE (2 mL)	$PhSeO_2H$ (1 eq.)	DBU (1.1 eq.)	-	DCE (1 mL)	r.t.	15	0	27	50
**9**	DCE (2 mL)	$PhSeO_2H$ (1 eq.)	NEt_3 (1.1 eq.)	-	DCE (1 mL)	r.t.	14	0	24	50
**10**	DCE (2 mL)	$PhSeO_2H$ (1 eq.)	DIPA (1.1 eq.)	-	DCE (1 mL)	r.t.	14	0	25	50
**11**	DCE (2 mL)	$PhSeO_2H$ (1 eq.)	NEt_3 (2.2 eq.)	Oxone (2.2 equiv), $Ru(bpy)_3PF_6$ (2 mol%), blue LEDs	DCE (1 mL)	r.t.	12	10	10	ND
**12**	DCE (2 mL)	$PhSeO_2H$ (0.5 eq.)	NEt_3 (2.2 eq.)	Oxone (2.2 equiv), blue LEDs	DCE (1 mL)	r.t.	17	10	15	ND
**13**	DCE (2 mL)	$PhSeO_2H$ (1 eq.)	NEt_3 (2.2 eq.)	PIDA (2.2 eq.)	DCE (1 mL)	r.t.	25	22	12	ND
**14**	DCE (2 mL)	$PhSeO_2H$ (1 eq.)	NEt_3 (2.2 eq.)	Selectfluor (2.2 eq.)	DCE (1 mL)	r.t.	100	0	0	0
**15**	DCE (2 mL)	$PhSeO_2H$ (1 eq.)	NEt_3 (2.2 eq.)	Sodium persulfate (2.2 equiv)	DCE (1 mL)	r.t.	25	0	40	ND
**16**	DCM (2 mL)	$PhSeO_2H$ (1 eq.)	NEt_3 (2.2 eq.)	Koser's (2.2 equiv)	DCM (1 mL)	r.t.	60	0	18	ND
**17**	DCM (2 mL)	$PhSeO_2H$ (1 eq.)	NEt_3 (2.2 eq.)	DMP (2.2 eq.)	DCM (1 mL)	r.t.	12	59	0	ND
**18**	DCM (2 mL)	$PhSeO_2H$ (1 eq.)	NEt_3 (2.2 eq.)	H_2O_2-urea (2.2 eq.)	DCM (1 mL)	r.t.	11	0	54	ND
**19**	DCM (2 mL)	$PhSeO_2H$ (1 eq.)	NEt_3 (2.2 eq.)	DMP (2.2 eq.)	DMF (1 mL)	r.t.	11	42	0	ND
**20**	DCM (2 mL)	$PhSeO_2H$ (1 eq.)	NEt_3 (2.2 eq.)	DMP (2.2 eq.)	MeCN (1 mL)	r.t.	10	61	2	ND
**21**	DCM (2 mL)	$PhSeO_2H$ (1.5 eq.)	NEt_3 (3 eq.)	DMP (3.0 eq.)	DCM (1 mL)	r.t.	10	27	0	ND

**22**	DCM (2 mL)	$PhSeO_2H$ (1 eq.)	NEt_3 (1.1 eq.)	DMP (1.1 eq.)	DCM (1 mL)	r.t.	7	39	12	ND
**23**	DCM (2 mL)	$PhSeO_2H$ (1 eq.)	NEt_3 (2.2 eq.)	DMP (1.1 eq.)	DCM (1 mL)	r.t.	10	55	5	ND
**24**	DCM (2 mL)	-	NEt_3 (2.2 eq.)	DMP (2.2 eq.)	DCM (1 mL)	r.t.	50	8	2	ND
**25**	DCM (2 mL)	PhSeSePh (0.5 eq.)	NEt_3 (4.0 eq.)	DMP (4 eq.)	MeCN (1 mL)	r.t.	15	0	0	ND
**26**	DCM (2 mL)	$PhSeO_2H$ (3 eq.)	-	-	-	r.t.	25	0	38	0
**27**	DCM (2 mL)	$PhSeO_2H$ (1.1 eq.)	NEt_3 (1.1 eq.)	TEMPO (1.1 eq.)	DCM (1 mL)	r.t.	messy			
**28**	DCM (2 mL)	$PhSeO_2H$ (1.1 eq.)	NEt_3 (1.1 eq.)	PIFA (1.1 eq.)	DCM (1 mL)	r.t.	18	30	n.d.	
**29**	DCM (2 mL)	$PhSeO_2H$ (1.1 eq.)	NEt_3 (1.1 eq.)	$TBAIO_4$ (1.1 eq.)	DCM (1 mL)	r.t.	9	28	27	
**30**	DCM (2 mL)	$PhSeO_2H$ (1 eq.)	NEt_3 (2.2 eq.)	DMP (2.2 eq.)	DCM (1 mL)	r.t.	16	62	10	<19%
**31**	DCM (2 mL)	$PhSeO_2H$ (1 eq.)	NEt_3 (2.2 eq.)	DMP (2.2 eq.)	DCM (1 mL)	r.t.	15	63	8	
**32**	DCM (2 mL)	$PhSeO_2H$ (1 eq.)	NEt_3 (2.2 eq.)	IBX (2.2 eq.)	DCM (1 mL)	r.t.	21	29	45	
**33**	DCM (2 mL)	$PhSeO_2H$ (1 eq.)	NEt_3 (2.2 eq.)	DMP (2.2 eq.)	DCM (1 mL)	r.t.	15	50	13	
**34**	DCM (4 mL)	$PhSeO_2H$ (1 eq.)	NEt_3 (2.2 eq.)	DMP (2.2 eq.)	DCM (2 mL)	r.t.	17	66	11	
**35**	DCM (2 mL)	$PhSeO_2H$ (1 eq.)	NEt_3 (2.2 eq.)	DMP (3 eq.)	DCM (1 mL)	r.t.	17	69	-	
**36**[b]	DCE (2 mL)	$PhSeO_2H$ (1 eq.)	NEt_3 (2.2 eq.)	DMP (2.2 eq.)	DCM (1 mL)	r.t.	45	21	-	
**37**[c]	DCM (2 mL)	$PhSeO_2H$ (1 eq.)	NEt_3 (2.2 eq.)	DMP (2.2 eq.)	DCM (1 mL)	r.t.	28	22	-	
**38**	DCM (2 mL)	$MeSeO_2H$ (1 eq.)	NEt_3 (2.2 eq.)	DMP (2.2 eq.)	DCM (1 mL)	r.t.	20	21	-	
**39**	DCM (4 mL)	$PhSeO_2H$ (1 eq.)	2.6-lutidine (2.2 eq.)	DMP (3 eq.)	DCM (2 mL)	r.t.	22	59	4	
**40**	DCM (4 mL)	$PhSeO_2H$ (1 eq.)	NEt_3 (2.2 eq.)	DMP (3 eq.)	DCM (2 mL)	r.t.	23	65	-	
**41**	CH_3CN (4 mL)	$PhSeO_2H$ (1 eq.)	NEt_3 (2.2 eq.)	DMP (3 eq.)	CH_3CN (2 mL)	r.t.	45	37	-	
**42**	DCM (2 mL)	$PhSeO_2H$ (1 eq.)	NEt_3 (2.2 eq.)	TFAA (1 eq.)	DCM (1 mL)	r.t.	31	14	0	29
**43**	DCM (2 mL)	$PhSeO_2H$ (1 eq.)	NEt_3 (2.2 eq.)	PTS Anhydride (1 eq.)	DCM (1 mL)	r.t.	28	5	22	21
**44**	DCM (2 mL)	$PhSeO_2H$ (1 eq.)	NEt_3 (2.2 eq.)	Yamaguchi's reagent (1 eq.)	DCM (1 mL)	r.t.	39	4	0	30
**45**	DCM (2 mL)	$PhSeO_2H$ (1 eq.)	NEt_3 (2.2 eq.)	Pivalic anhydride (1 eq.)	DCM (1 mL)	r.t.	24	9	0	41
**46**	DCM (2 mL)	$PhSeO_2H$ (1 eq.)	NEt_3 (2.2 eq.)	Tf_2O (1 eq.)	DCM (1 mL)	r.t.	30	16	0	20

**47**	DCM (2 mL)	Benzene seleninic anhydride (1 eq.)		Tf_2O (1 eq.)	-	r.t.	25	6	4	5
**48**	DCM (2 mL)	Benzene seleninic anhydride (1 eq.)	NEt_3 (2.2 eq.)	Tf_2O (1 eq.)	DCM (1 mL)	r.t.	15	-	0	20
**49**	DCM (2 mL)	Benzene seleninic anhydride (1 eq.)	NEt_3 (2.2 eq.) + DMP (2eq.)	Tf_2O (1 eq.)	DCM (1 mL)	r.t.	5	45	-	13
**50**	DCM (2 mL)	Benzene seleninic anhydride (1 eq.)	AcOH (1 eq.) + NEt_3 (2.2 eq.)	Tf_2O (1 eq.)	DCM (1 mL)	r.t.	23	5	30	30

[a]NMR yield (in %) determined with the use of bromoform as an internal reference, [b]2-F-Py used instead of 2-I-Py, [c]2-Cl-Py used instead of 2-I-Py. TFAA: trifluoroacetic anhydride; PTS Anhydride: *para*-toluene sulfonic anhydride; Yamaguchi's reagent: 2,4,6-trichlorobenzoyl chloride; Tf_2O: trifluoromethane sulfonic anhydride.

3. Amides

3.1. Synthesis of Amides

General Procedure A:

To a solution of the amine (1.00 eq.) and triethylamine (2.00 eq.) in dichloromethane (0.1 M) at 0°C, the corresponding acyl chloride (1.20 eq.) was added dropwise and the resulting reaction mixture was allowed to warm to room temperature while stirring overnight (14 h). After this time, a saturated aqueous solution of sodium bicarbonate was added and the biphasic system was separated. The aqueous phase was extracted with dichloromethane (1×) and the organic phases were combined and dried over anhydrous sodium sulfate. The dried solution was filtered and concentrated under reduced pressure. The resulting crude material was purified by flash column chromatography on silica gel to afford the desired compound.

General Procedure B:

To a solution of the amine (1.00 eq.), triethylamine (1.00 eq.), hydroxybenzotriazole (HOBt, 1.00 eq.) and 1-ethyl-3-(3-dimethylaminopropyl) carbodiimide hydrochloride (EDCI*HCl, 1.00 eq.) in DCM (0.1 M), the corresponding carboxylic acid was added and the resulting solution was stirred at room temperature overnight (14 h). After this time, the organic solution was washed sequentially with 0.5 M aqueous hydrochloric acid, saturated aqueous sodium bicarbonate and saturated aqueous sodium chloride. The washed solution was dried over anhydrous sodium sulfate, filtered and concentrated under reduced pressure. The resulting crude material was purified by flash column chromatography on silica gel (heptane/ethyl acetate) to afford the desired compound.

Given the difficulty of separating the dehydrogenated products from the starting materials, we have reported R_f values in a solvent system which we found best provided some separation between the starting materials and products.

3.2. Characterization

4-Phenyl-1-(pyrrolidin-1-yl)butan-1-one (1a)

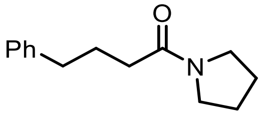

General Procedure B was carried out on 12 mmol scale; purification by silica gel flash chromatography (EtOAc/heptanes 1:9 to 3:7) yielded the product as a colourless oil (2.16 g, 83 %) with R_f = 0.4 (acetone/heptanes 2:3). All analytical data were in good accordance with data reported in the literature.[1]

3-Cyclopentyl-1-(pyrrolidin-1-yl)propan-1-one (1b)

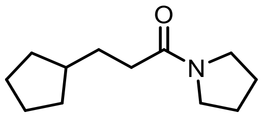

General Procedure A was carried out on 3 mmol scale (relative to the amine); purification by silica gel flash chromatography (EtOAc/heptanes 1:9 to 3:7) yielded the product as a colourless oil (580 mg, quant. yield) with R_f = 0.27 (acetone/heptanes 2:3). All analytical data were in good accordance with data reported in the literature.[1]

3-(4-Bromophenyl)-*N*,*N*-dimethylpropanamide (1c)

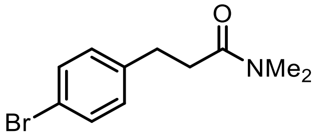

General Procedure B was carried out on 3 mmol scale (relative to the amine); purification by silica gel flash chromatography (EtOAc/heptanes 1:19 to 2:8) yielded the product as a white crystalline solid (730 mg, 95%) with R_f = 0.36 (acetone/heptanes 2:3). All analytical data were in good accordance with data reported in the literature.[2]

***N*,*N*-Dimethyl-3-phenylpropanamide (1d)**

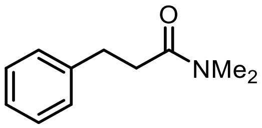

General Procedure A was carried out on 3.3 mmol scale (relative to the amine); purification by silica gel flash chromatography (EtOAc/heptanes 1:19 to 3:7) yielded the product as a pale yellow oil (586 mg, quant. yield) with R_f = 0.25 (acetone/heptanes 2:3). All analytical data were in good accordance with data reported in the literature.[2]

***N,N*-Diethyl-3-phenylpropanamide (1e)**

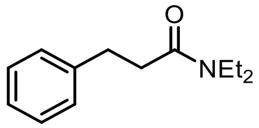

General Procedure A was carried out on 5 mmol scale (relative to the amine); purification by silica gel flash chromatography (EtOAc/heptanes 1:19 to 2:8) yielded the product as a yellow oil (980 mg, 96%) with R_f = 0.34 (acetone/heptanes 2:3). All analytical data were in good accordance with data reported in the literature.[2]

(2,3-Dihydro-1H-inden-2-yl)(pyrrolidin-1-yl)methanone (1f)

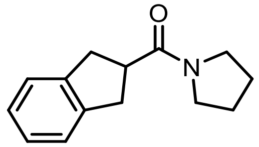

General Procedure B was carried out on 1.6 mmol scale; purification by silica gel flash chromatography (EtOAc/heptanes 1:19 to 1:9) yielded the product as a white crystalline solid (230 mg, 66%) with R_f = 0.38 (acetone/heptanes 2:3). **^{1}H NMR (600 MHz, $CDCl_3$)**: δ 7.21 – 7.18 (m, 2H), 7.16 – 7.12 (m, 2H), 3.53 (dt, *J* = 13.7, 6.9 Hz, 4H), 3.47 – 3.38 (m, 1H), 3.35 – 3.29 (m, 2H), 3.16 – 3.09 (m, 2H), 1.99 (p, *J* = 6.8 Hz, 2H), 1.89 (p, *J* = 6.8 Hz, 2H); **^{13}C NMR (151 MHz, $CDCl_3$):** δ 173.2, 142.1 (2C), 126.5 (2C), 124.3 (2C), 46.7, 46.1, 43.7, 36.4 (2C), 26.3, 24.4; **IR (neat) ν_{max}:** 3071, 3043, 2973, 2861, 1621, 1477, 1435, 1335, 1312, 1226, 1191, 1011, 910, 740; **HRMS (ESI+):** exact mass calculated for $[M+Na]^+$ ($C_{14}H_{17}NNaO$) requires *m/z* 238.1202, found *m/z* 238.1205.

Cyclobutyl(pyrrolidin-1-yl)methanone (1g)

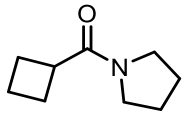

General Procedure B was carried out on 2.0 mmol scale; purification by silica gel flash chromatography (EtOAc/heptanes 1:19 to 2:8) yielded the product as a colourless oil (220 mg, 74%) with R_f = 0.36 (acetone/heptanes 2:3). **^{1}H NMR (600 MHz, $CDCl_3$)**: δ 3.40 (t, *J* = 6.9 Hz, 2H), 3.28 (t, *J* = 6.8 Hz, 2H), 3.15 (p, *J* = 8.6 Hz, 1H), 2.33 – 2.25 (m, 2H), 2.12 – 2.05 (m, 2H), 1.95 – 1.75 (m, 6H); **^{13}C NMR (151 MHz, $CDCl_3$):** δ 173.3, 45.9, 45.7, 38.5, 26.2, 24.7 (2C), 24.3, 18.1; **IR (neat) ν_{max}:** 2972, 2946, 2871, 1632, 1433, 1341, 1312, 1257, 1226, 1191, 1171, 751; **HRMS (ESI+):** exact mass calculated for $[M+Na]^+$ ($C_9H_{15}NNaO$) requires *m/z* 176.1046, found *m/z* 176.1049.
All analytical data were in good accordance with data reported in the literature.[3]

1-(Piperidin-1-yl)pent-4-en-1-one (1h)

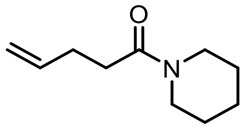 General Procedure B was carried out on 5.0 mmol scale; purification by silica gel flash chromatography (EtOAc/heptanes 1:49 to 1:9) yielded the product as a yellow oil (753 mg, 90%) with R_f = 0.46 (acetone/heptanes 2:3). All analytical data were in good accordance with data reported in the literature.[4]

1-(Pyrrolidin-1-yl)undec-10-yn-1-one (1i)

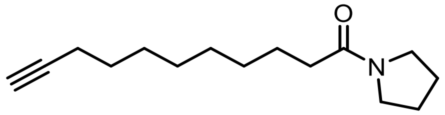 General Procedure B was carried out on 5.0 mmol scale; purification by silica gel flash chromatography (EtOAc/heptanes 1:19 to 2:8) yielded the product as a yellow oil (941 mg, 80%) with R_f = 0.44 (acetone/heptanes 1:2); **^{1}H NMR (400 MHz, $CDCl_3$)**: δ 3.44 (t, *J* = 6.9 Hz, 2H), 3.39 (t, *J* = 6.8 Hz, 2H), 2.27 – 2.20 (m, 2H), 2.16 (td, *J* = 7.1, 2.6 Hz, 2H), 1.97 – 1.89 (m, 3H), 1.87 – 1.79 (m, 2H), 1.68 – 1.59 (m, 2H), 1.55 – 1.46 (m, 2H), 1.41 – 1.26 (m, 8H); **^{13}C NMR (101 MHz, $CDCl_3$):** δ 171.9, 84.9, 68.2, 46.7, 45.7, 34.9, 29.6, 29.4, 29.1, 28.8, 28.6, 26.3, 25.0, 24.5, 18.5; **IR (neat) ν_{max}:** 3288, 3227, 2927, 2855, 1634, 1427, 1341, 1252, 1226, 1193, 1170, 914, 857, 725; **HRMS (ESI+):** exact mass calculated for $[M+Na]^+$ ($C_{15}H_{25}NNaO$) requires *m/z* 258.1828, found *m/z* 258.1829.

4,4,4-Trifluoro-1-(pyrrolidin-1-yl)butan-1-one (1j)

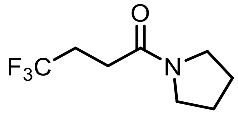 General Procedure B was carried out on 1.6 mmol scale; purification by silica gel flash chromatography (EtOAc/heptanes 1:4 to 1:1) yielded the product as a yellow oil (310mg, 79%) with R_f = 0.6 (acetone/heptanes 1:2); **^{1}H NMR (400 MHz, $CDCl_3$)**: δ 3.47 (t, *J* = 6.9 Hz, 2H), 3.41 (t, *J* = 6.8 Hz, 2H), 2.55 – 2.48 (m, 4H), 2.02 – 1.93 (m, 2H), 1.87 (qd, *J* = 6.8, 0.9 Hz, 2H); **^{13}C NMR (151 MHz, $CDCl_3$):** δ 168.3, 127.3 (q, *J* = 275 Hz), 46.7, 46.0, 29.4 (q, *J* = 29.4 Hz), 27.4 (q, *J* = 2.8 Hz), 26.2, 24.5; **^{19}F NMR (659 MHz, $CDCl_3$)**: δ -66.7; **IR (neat) ν_{max}:** 2960, 2878, 1639, 1443, 1380, 1344; **HRMS (ESI+):** exact mass calculated for $[M+H]^+$ ($C_8H_{13}NOF_3$) requires *m/z* 196.0944 found *m/z* 196.0945.

_N,N_-Dibenzylpentanamide (1k)

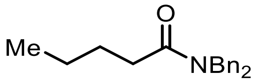 General Procedure A was carried out on 5 mmol scale (relative to the amine); purification by silica gel flash chromatography (EtOAc/heptanes 1:19 to 2:8) yielded the product as a yellow oil (1.40 g, quant. yield) with R_f = 0.50 (acetone/heptanes 2:3). All analytical data were in good accordance with data reported in the literature.[5]

1-Morpholinobutan-1-one (1l)

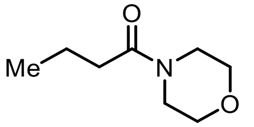 General Procedure A was carried out on 3.0 mmol scale; purification by silica gel flash chromatography (EtOAc/heptanes 1:5 to 1:1) yielded the product as a yellow oil (480 mg, 80%) with R_f = 0.50 (acetone/heptanes 1:2). All analytical data were in good accordance with data reported in the literature.[6]

1-(Piperidin-1-yl)butan-1-one (1m)

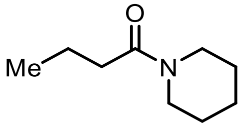 General Procedure A was carried out on 2 mmol scale; purification by silica gel flash chromatography (EtOAc/heptanes 1:19 to 1:4) yielded the product as a colourless oil (310 mg, 99%) with R_f = 0.44 (acetone/heptanes 2:3). All analytical data were in good accordance with data reported in the literature.[7]

1-(Azepan-1-yl)butan-1-one (1n)

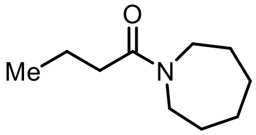 General Procedure A was carried out on 2.0 mmol scale; purification by silica gel flash chromatography (EtOAc/heptanes 1:5 to 2:3) yielded the product as a yellow oil (330 mg, quant. yield) with R_f = 0.65 (acetone/heptanes 1:2). All analytical data were in good accordance with data reported in the literature.[7]

N-Methyl-N-phenylpentanamide (1o)

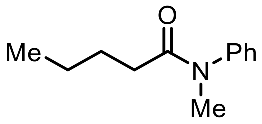

General Procedure A was carried out on 4.0 mmol scale; purification by silica gel flash chromatography (EtOAc/heptanes 1:9 to 1:4) yielded the product as an orange oil (650 mg, 85%) with R_f = 0.48 (acetone/heptanes 2:3). All analytical data were in good accordance with data reported in the literature.[8]

N-(4-Methoxyphenyl)-N-methylbutyramide (1p)

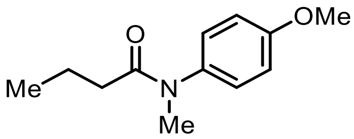

NaH (60% in paraffin oil, 248 mg, 6.2 mmol, 2.0 eq.) was added to a solution of *N*-(4-methoxyphenyl)butyramide (600 mg, 3.1 mmol, 1.0 eq., prepared in 57% yield according to general procedure A) in tetrahydrofuran (15 mL) at 0 °C and stirred for 4 hours. Subsequently methyl iodide (0.39 ml, 6.2 mmol, 1.0 eq.) was slowly added to the mixture and stirred for 12 hours. The mixture was poured into 50 mL of water, the aqueous layer extracted with dichloromethane (3 x 25 mL) and the organic phases were combined and dried over anhydrous sodium sulfate. The dried solution was filtered and concentrated under reduced pressure. The resulting crude material was purified by flash column chromatography on silica gel (EtOAc/heptanes 1:9 to 3:7) to yield the product as a yellow oil (462 mg, 72%) with R_f = 0.40 (acetone/heptanes 2:3); **^{1}H NMR (600 MHz, $CDCl_3$)**: δ 7.08 – 7.05 (m, 2H), 6.92 – 6.88 (m, 2H), 3.81 (s, 3H), 3.20 (s, 3H), 2.03 – 1.99 (m, 2H), 1.60 – 1.52 (m, 2H), 0.80 (t, *J* = 7.4 Hz, 3H); **^{13}C NMR (151 MHz, $CDCl_3$):** δ 173.6, 158.9, 137.2, 128.5 (2C), 114.9 (2C), 55.6, 37.5, 36.0, 19.0, 14.0; **IR (neat) ν_{max}:** 2961, 2934, 2874, 1651, 1510, 1462, 1420, 1385, 1292, 1246, 1171, 1129, 1105, 1036, 839; **HRMS (ESI+):** exact mass calculated for $[M+Na]^+$ ($C_{12}H_{17}NNaO_2$) requires *m/z* 230.1151, found *m/z* 230.1153.

N-Allyl-N-benzylbutyramide (1q)

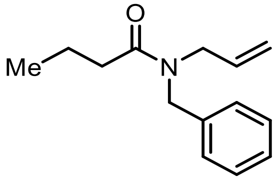

General Procedure A was carried out on 6.1 mmol scale; purification by silica gel flash chromatography (EtOAc/heptanes 3:10 to 1:2) gave the product as a yellow oil (1.43 g, 82%) with an

R_f = 0.6 (acetone/heptanes 1:2). All analytical data were in good accordance with data reported in the literature.[5]

Methyl 9-(azepan-1-yl)-9-oxononanoate (1r)

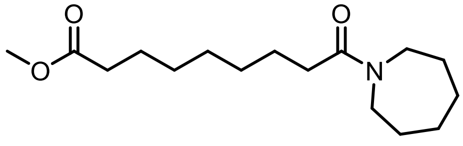

General Procedure B was carried out on a 0.82 mmol scale; purification by silica gel flash chromatography (EtOAc/heptanes 1:9 to 3:7) yielded the product as a colourless oil (237 mg, 72%) with R_f = 0.35 (acetone/heptanes 1:2); **^{1}H NMR (600 MHz, CDCl$_3$):** δ 3.66 (s, 3H), 3.53 – 3.49 (m, 2H), 3.43 – 3.40 (m, 2H), 2.30 – 2.28 (m, 4H), 1.74 – 1.68 (m, 4H), 1.62 (dt, *J* = 13.7, 6.8 Hz, 4H), 1.59 – 1.52 (m, 4H), 1.33 – 1.32 (m, 6H); **^{13}C NMR (151 MHz, CDCl$_3$):** δ 174.4, 172.8, 51.6, 48.0, 46.1, 34.2, 33.4, 29.5, 29.4, 29.2, 29.1, 27.8, 27.2, 27.0, 25.5, 25.0; **IR (neat) ν_{max}:** 2925, 2854, 1736, 1637, 1426, 1357; **HRMS (ESI+):** exact mass calculated for $[M+H]^+$ ($C_{16}H_{30}NO_3$) requires *m/z* 284.2220 found *m/z* 284.2218.

Methyl 9-(diethylamino)-9-oxononanoate (1s)

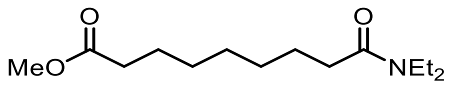

General Procedure B was carried out on 1.0 mmol scale; purification by silica gel flash chromatography (EtOAc/heptanes 1:9 to 1:2) yielded the product as a colourless oil (178 mg, 69%) with R_f = 0.66 (acetone/heptanes 1:1). **^{1}H NMR (400 MHz, CDCl$_3$)**: δ 3.65 (s, 3H), 3.36 (q, *J* = 7.2 Hz, 2H), 3.28 (q, *J* = 7.2 Hz, 2H), 2.27 (app q, *J* = 7.6 Hz, 4H), 1.67–1.56 (m, 4H), 1.36–1.28 (m, 6H), 1.16 (t, *J* = 7.2 Hz, 3H), 1.09 (t, *J* = 7.2 Hz, 3H); **^{13}C NMR (100 MHz, CDCl$_3$):** δ 174.4, 172.3, 51.5, 42.1, 40.1, 34.2, 33.2, 29.4, 29.2, 29.1, 25.5, 25.0, 14.5, 13.2; **IR (neat) ν_{max}:** 2932, 2856, 1738, 1640, 1460, 1432, 1378, 1362, 1262, 1222, 1199, 1171; **HRMS (ESI+):** exact mass calculated for $[M+Na]^+$ ($C_{14}H_{27}NO_3Na$) requires *m/z* 280.1883, found *m/z* 280.1883.

Methyl 9-(diisobutylamino)-9-oxononanoate (1t)

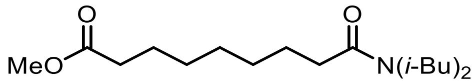

General Procedure B was carried out on 1.0 mmol scale; purification by silica gel flash chromatography (EtOAc/heptanes 1:9 to 1:1) yielded the product as a colourless oil (313 mg, 94%) with R_f = 0.44 (acetone/heptanes 1:2); **^{1}H NMR (400 MHz, CDCl$_3$)**: δ 3.68 – 3.60 (m, 3H), 3.21 – 3.13 (m, 2H), 3.09 – 3.02 (m, 2H), 2.33 – 2.23 (m, 4H), 2.05 – 1.82 (m, 2H), 1.67 – 1.52

(m, 4H), 1.36 – 1.25 (m, 6H), 0.90 (dd, *J* = 9.4, 4.6 Hz, 6H), 0.85 (dd, *J* = 9.5, 4.8 Hz, 6H); **^{13}C NMR (101 MHz, CDCl$_3$):** δ 174.4, 173.5, 55.7, 53.2, 51.5, 34.2, 33.5, 29.4, 29.2, 29.1, 28.1, 26.7, 25.7, 25.0, 20.3 (2C), 20.2 (2C); **IR (neat) ν_{max}:** 2955, 2931, 2870, 1739, 1642, 1465, 1422, 1387, 1366, 1242, 1199, 1171, 1099; **HRMS (ESI+):** exact mass calculated for $[M+Na]^+$ ($C_{18}H_{35}NO_3Na$) requires *m*/*z* 336.2509, found *m*/*z* 336.2509.

Methyl 9-(indolin-1-yl)-9-oxononanoate (1u)

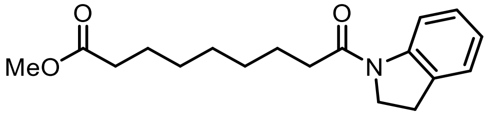

General Procedure B was carried out on 1.0 mmol scale; purification by silica gel flash chromatography (EtOAc/heptanes 1:9 to 1:1) yielded the product as an off-white solid (230 mg, 76%) with R_f = 0.40 (acetone/heptanes 1:1); **^{1}H NMR (600 MHz, CDCl$_3$):s** δ 8.23 (d, *J* = 8.0 Hz, 1H), 7.20 – 7.13 (m, 2H), 7.02 – 6.97 (m, 1H), 4.04 (t, *J* = 8.5 Hz, 2H), 3.66 (s, 3H), 3.18 (t, *J* = 8.4 Hz, 2H), 2.40 (t, *J* = 7.5 Hz, 2H), 2.30 (t, *J* = 7.5 Hz, 2H), 1.76 – 1.69 (m, 2H), 1.65 – 1.59 (m, 2H), 1.41 – 1.32 (m, 6H); **^{13}C NMR (151 MHz, CDCl$_3$):** δ 174.4, 171.5, 143.2, 131.1, 127.7, 124.6, 123.6, 117.1, 51.6, 48.1, 36.0, 34.2, 29.3, 29.2, 29.1, 28.2, 25.0, 24.6; **IR (neat) ν_{max}:** 2932, 2856, 1736, 1660, 1599, 1482, 1461, 1409, 1338, 1263, 1198, 1169, 1116, 1096, 756; **HRMS (ESI+):** exact mass calculated for $[M+Na]^+$ ($C_{18}H_{25}NNaO_3$) requires *m*/*z* 326.1727, found *m*/*z* 326.1726.

***N*-Benzyl-*N*-methyl-10-oxoundecanamide (1v)**

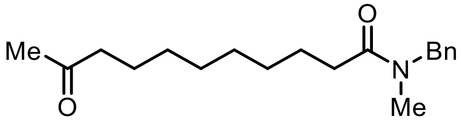

Prepared from *N*-benzyl-*N*-methylundec-10-enamide according to the following procedure: To a solution of amide (949 mg, 3.3 mmol, 1.0 eq.) and $Pd(OAc)_2$ (74.1 mg, 0.33 mmol, 0.1 eq.) in 10 mL DMSO/water (0.33M, 9/1, v/v) in a Schlenk tube under oxygen atmosphere was added TFA (0.25 ml, 0.33 mmol, 0.1 eq.) and the reaction was heated to 70 °C for 15 hours. The reaction mixture was quenched with water and extracted with ethyl acetate. The organic phases were combined and washed with brine then dried over anhydrous sodium sulfate. The dried solution was filtered and concentrated under reduced pressure. The resulting crude material was purified by flash column chromatography on silica gel (DMA mixture [90% DCM, 10% methanol, 1% NH_2OH]/DCM: 0 to 20%) to afford the desired compound as a yellow oil (230 mg, 23%, 97% BRSM) with R_f = 0.60 (acetone/heptanes 1:1).

^{1}H NMR (600 MHz, CDCl$_3$): δ 7.39 – 7.33 (m, 1H), 7.32 – 7.20 (m, 3H), 7.15 (d, *J* = 7.5 Hz, 1H), 4.58 (s, 1H), 4.53 (s, 1H), 2.93 (s, 1H), 2.90 (s, 2H), 2.44 – 2.30 (m, 4H), 2.12 (s, 2H), 2.11 (s, 1H), 1.70 – 1.60 (m, 2H),

1.58 – 1.51 (m, 2H), 1.38 – 1.23 (m, 8H); **^{13}C NMR (151 MHz, $CDCl_3$):** δ 209.5, 173.8, 173.4, 137.7, 136.9, 129.0, 128.7, 128.1, 127.7, 127.4, 126.4, 53.5, 50.9, 43.9, 35.0, 34.0, 33.6, 33.2, 30.0, 29.5, 29.5, 29.4, 29.3, 29.2, 29.2, 25.5, 25.2, 23.9; **IR (neat) ν_{max}:** 2927, 2855, 1713, 1644, 1493, 1453, 1404, 1358, 1262, 1168, 1121, 1079, 735, 700; **HRMS (ESI+):** exact mass calculated for $[M+Na]^+$ ($C_{19}H_{29}NNaO_2$) requires *m/z* 326.2091, found *m/z* 326.2092.

7-Oxo-7-(pyrrolidin-1-yl)heptanenitrile (1w)

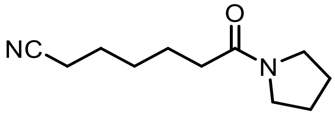

General Procedure B was carried out on 1.0 mmol scale; purification by silica gel flash chromatography (EtOAc/heptanes 1:9 to 1:1) yielded the product as a white crystalline solid (155 mg, 88%) with R_f = 0.44 (acetone/heptanes 1:2); All analytical data were in good accordance with data reported in the literature. [7]

(*Z*)-1-(Pyrrolidin-1-yl)docos-13-en-1-one (1x)

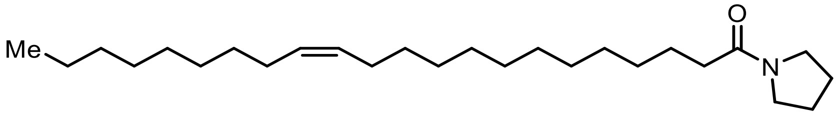

General Procedure B was carried out on a 4.0 mmol scale; purification by silica gel flash chromatography (EtOAc/heptanes 1:9 to 1:1) yielded the product as a yellow oil (1.62 g, 82%) with R_f = 0.35 (acetone/heptanes 1:2); **^{1}H NMR (700 MHz, $CDCl_3$)**: δ 5.34 (t, *J* = 4.5 Hz, 2H), 3.46 (t, *J* = 6.9 Hz, 2H), 3.40 (t, *J* = 6.8 Hz, 2H), 2.27 – 2.22 (m, 2H), 2.01 (dd, *J* = 12.2, 6.5 Hz, 4H), 1.97 – 1.91 (m, 2H), 1.84 (dd, *J* = 10.7, 4.2 Hz, 2H), 1.67 – 1.61 (m, 2H), 1.38-1.20 (m, 28H), 0.88 (t, *J* = 6.9 Hz, 3H); **^{13}C NMR (151 MHz, $CDCl_3$):** δ 172.0, 130.0, 46.7, 45.7, 35.0, 32.0, 29.9, 29.7, 29.7, 29.6, 29.4, 27.3, 26.3, 25.1, 24.5, 22.8, 14.2; **IR (neat) ν_{max}:** 2921, 2851, 1646, 1425, 1343; **HRMS (ESI+):** exact mass calculated for $[M+H]^+$ ($C_{26}H_{50}NO$) requires *m/z* 392.3887, found *m/z* 392.3890.

(9*S*,10*S*,13*R*,14*S*,17*R*)-10,13-Dimethyl-17-((*R*)-5-oxo-5-(pyrrolidin-1-yl)pentan-2-yl)dodecahydro-3H-cyclopenta[a]phenanthrene-3,7,12(2H,4H)-trione (1y)

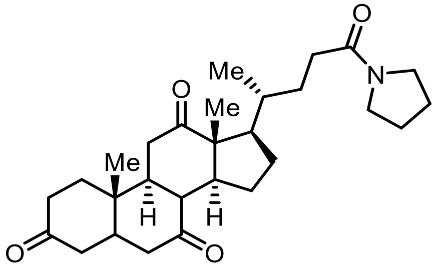

General Procedure B was carried out on 1.0 mmol scale; purification by silica gel flash chromatography (EtOAc/MeOH 1:0 to 95:5) yielded the product as a white solid (269 mg, 59%) with R_f = 0.15 (EtOAc); **^{1}H NMR (400 MHz, $CDCl_3$)**: δ 3.43 (dt, *J* = 15.3, 7.0 Hz, 4H), 2.95–2.80 (m, 3H), 2.38–1.79 (m, 20H), 1.66–1.55 (m, 1H), 1.45–1.32 (m, 6H), 1.31–1.21 (m, 1H), 1.07 (s, 3H), 0.86 (d, *J* = 6.3 Hz, 3H); **^{13}C NMR (100 MHz, $CDCl_3$):** δ 212.2, 209.1, 208.8, 172.0, 57.1, 51.9, 49.1, 47.0, 46.7, 45.8, 45.7, 45.7, 45.1, 42.9, 38.8, 36.6, 36.1, 35.6, 35.4, 31.7, 30.4, 27.6, 26.3, 25.3, 24.5, 22.0, 19.0, 12.1; **IR (neat) ν_{max}:** 2968, 2928, 2866, 1700, 1633, 1422, 1383, 1338, 1283; **HRMS (ESI+):** exact mass calculated for $[M+Na]^+$ ($C_{28}H_{41}NO_4Na$) requires *m/z* 478.2928, found *m/z* 478.2924.

3-(5-Cyano-1-(4-fluorophenyl)-1,3-dihydroisobenzofuran-1-yl)-*N*,*N*-dimethylpropanamide (1z)

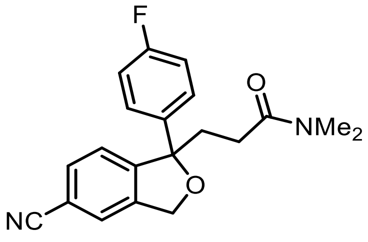

To a solution of 1-(4-fluorophenyl)-1,3-dihydroisobenzofuran-5-carbonitrile (1.2 g, 5 mmol, 1.0 eq.) in DMF (3 mL) was added sodium hydride (60% in paraffin oil, 40 mg, 0.1 mmol, 0.2 eq.) and the resulting mixture was stirred at room temperature (23 °C) for 30 min. Then, a solution of *N*,*N*-dimethylacrylamide (515 µL, 5 mmol, 1.0 eq.) in DMF (2 mL) was added in one portion. After 16 h, the reaction was quenched by addition of water. The biphasic mixture was extracted with ethyl acetate (3×) and the combined organic extracts were concentrated under reduced pressure. Flash column chromatography of the crude residue over silica gel (EtOAc/heptanes 1:1 to 8:2) afforded the product as an off-white foamy liquid (1.45 g, 86%) with R_f = 0.42 (acetone/heptanes 1:1).

^{1}H NMR (600 MHz, $CDCl_3$): δ 7.58 (br d, *J* = 7.9 Hz, 1H), 7.49 (br s, 1H), 7.45–7.39 (m, 3H), 7.03–6.98 (m, 2H), 5.19 (d, *J* = 12.9 Hz, 1H), 5.15 (d, *J* = 12.9 Hz, 1H), 2.86 (s, 3H), 2.85 (s, 3H), 2.61–2.54 (m, 1H), 2.47–2.41 (m, 1H), 2.25 (t, *J* = 8.0 Hz, 2H); **^{13}C NMR (150 MHz, $CDCl_3$):** δ 172.2, 162.2 (d, *J* = 246 Hz), 149.3, 140.0,

138.9 (d, J = 3.1 Hz), 132.1, 126.8 (d, J = 8.1 Hz, 2C), 125.3, 122.9, 118.7, 115.6 (d, J = 21.4 Hz, 2C), 111.9, 90.7, 71.3, 37.2, 36.2, 35.5, 28.0; **^{19}F NMR (659 MHz, CDCl$_3$):** δ –115.1; **IR (neat) ν_{max}:** 2927, 2230, 1640, 1506, 1400, 1225, 1159, 1145, 1046, 1030, 1014, 838, 600; **HRMS (ESI+):** exact mass calculated for $[M+Na]^+$ ($C_{20}H_{19}FN_2O_2Na$) requires m/z 361.1323, found m/z 361.1321.

1-Methylazacyclotridecan-2-one (1aa)

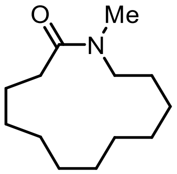

Sodium hydride (60% in paraffin oil, 88 mg, 2.2 mmol, 1.1 eq.) was added to a solution of lactam (395 mg, 2.0 mmol, 1.0 eq.) in DMF (10 mL) at 0 °C and stirred for 1 hours. Subsequently methyl iodide (377 µL, 6.0 mmol, 3.0 eq.) was slowly added to the mixture and stirred for 12 hours. The mixture was poured into 50 mL of water, the aqueous layer extracted with dichloromethane (3x25 mL) and the organic phases were combined and dried over anhydrous sodium sulfate. The dried solution was filtered and concentrated under reduced pressure. The resulting crude material was purified by flash column chromatography on silica gel to afford the desired compound as an off-white solid (232 mg, 55%) with R_f = 0.30 (acetone/heptanes 2:3); All analytical data were in good accordance with data reported in the literature.[9]

***N*-Cyclohexyl-9-oxo-9-(pyrrolidin-1-yl)nonanamide (1ab)**

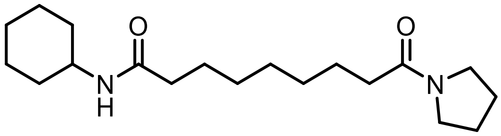

General Procedure B was carried out on a 5.0 mmol scale to give methyl 9-oxo-9-(pyrrolidin-1-yl)nonanoate as an yellow oil (1.21 g, 95% yield) with R_f = 0.35 (acetone/heptanes 2:3) after column chromatography on silica gel (EtOAc/heptanes 1:3 to 1:1). All analytical data were in good accordance with data reported in the literature.[5] Subsequent hydrolysis of the methyl ester (1.21 g, 4.8 mmol, 1.0 eq.) with lithium hydroxide (0.22 g, 5 mmol, 1.1 equiv) in a tetrahydrofuran/water mixture (1/1 – 10 mL, v/v) gave the corresponding carboxylic acid. The crude material then used directly in general procedure B to give the amide, **1ab**, after silica gel flash chromatography (EtOAc/heptanes 1:2 to 2:1) as a white solid (1.15 g, 73%) with R_f = 0.45 (acetone/heptanes 2:3); **^{1}H NMR (600 MHz, CDCl$_3$):** δ 5.30 (m, 1H), 3.80 – 3.73 (m, 1H), 3.45 (t, J = 6.9 Hz, 2H), 3.40 (t, J = 6.8 Hz, 2H), 2.26 – 2.22 (m, 2H), 2.13 – 2.10 (m, 2H), 1.96 – 1.93 (m, 2H), 1.92 – 1.88 (m,

2H), 1.84 (p, J = 6.8 Hz, 2H), 1.71 – 1.67 (m, 2H), 1.64 – 1.60 (m, 6H), 1.35 – 1.32 (m, 6H), 1.18 – 1.06 (m, 4H). **^{13}C NMR (151 MHz, $CDCl_3$):** δ 172.2, 171.9, 48.2, 46.7, 45.7, 37.2, 34.9, 33.4, 29.4, 29.2, 29.2, 26.3, 25.9, 25.7, 25.0, 25.0, 24.6; **IR (neat) ν_{max}:** 3285, 2926, 2853, 1622, 1542; **HRMS (ESI+):** exact mass calculated for $[M+H]^+$ ($C_{19}H_{35}N_2O_2$) requires *m/z* 323.2693 found *m/z* 323.2689.

4. Products of desaturation

4.1. General Procedure C

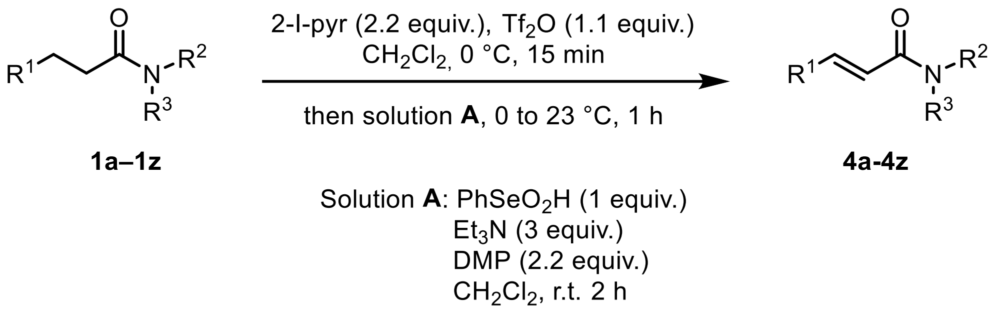

All reactions were run on a 0.2 mmol scale unless otherwise indicated.

To a solution of amide **1** (1.0 eq.) and 2-iodopyridine (2.2 eq.) in dichloromethane (2 mL, 0.1 M) at 0 °C was added trifluoromethanesulfonic anhydride (1.1 eq.) and the resulting mixture was stirred at 0 °C and colouration of the solution was observed. After 15 min, a previously prepared solution of **A** (1 mL, 0.2 M of $PhSeO_2H$) was added and the reaction mixture was stirred at 0 °C for 5 min. The resulting mixture was allowed to warm to ambient temperature (25 °C) over the course of 5 minutes to 1 h, after which time a saturated aqueous solution of sodium carbonate (5 mL) was added. The biphasic mixture was separated and the aqueous phase was extracted with dichloromethane (2 × 5 mL). The combined organic phases were dried over anhydrous sodium sulfate, the dried solution was filtered and the filtrate was concentrated under reduced pressure. The crude residue was purified by flash column chromatography (SiO_2, heptanes/ethyl acetate) to afford the compounds **4**. In most cases, the dehydrogenated amide **4** was isolated with remnants of the corresponding starting material (typically 5 – 10%). A further purification was necessary in most cases and was carried out on preparative TLC, typically using an acetone/heptanes system – to both determine the yield and for full characterization of the product – see individual compounds for details.

Preparation of solution **A**: To a suspension of the seleninic acid (1 eq.) in dichloromethane (1 mL for 0.2 mmol of the seleninic acid) was added the triethylamine (3 eq.). Solubilisation of the suspension was observed and then Dess-Martin periodinane (2.2 eq.) was added at room temperature and stirred 2 hours.

4.2. Characterization

(*E*)-4-Phenyl-1-(pyrrolidin-1-yl)but-2-en-1-one (4a)

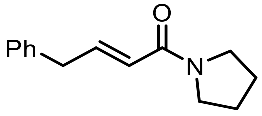

The product was prepared according to General Procedure C on 0.2 mmol scale. Following purification by silica gel flash chromatography (EtOAc/heptanes 1:9 to 4:6) and a further purification on preparative TLC (acetone/heptanes 1:1), the product was isolated as an off-white solid (26.7 mg, 62%) with R_f = 0.40 (acetone/heptanes 3:7); **^{1}H NMR (600 MHz, CDCl$_3$)**: δ 7.31 – 7.26 (m, 2H), 7.23 – 7.15 (m, 3H), 7.09 – 7.02 (m, 1H), 6.08 (dd, *J* = 15.1, 1.4 Hz, 1H), 3.54 – 3.48 (m, 4H), 3.45 (t, *J* = 6.8 Hz, 2H), 1.96 – 1.88 (m, 2H), 1.87 – 1.79 (m, 2H); **^{13}C NMR (151 MHz, CDCl$_3$):** δ 164.6, 143.7, 138.5, 128.8 (2C), 128.6 (2C), 126.5, 122.9, 46.5, 45.9, 38.6, 26.2, 24.4; **IR (neat) ν_{max}:** 3026, 2970, 2950, 2871, 1659, 1608, 1494, 1425, 1338, 1306, 1252, 1227, 1192, 1169, 1037, 981, 915, 866, 847, 816, 795, 750, 701; **HRMS (ESI+):** exact mass calculated for $[M+H]^+$ ($C_{14}H_{18}NO$) requires *m*/*z* 216.1383, found *m*/*z* 216.1383.

2-Hydroxy-4-phenyl-1-(pyrrolidin-1-yl)butan-1-one (5)

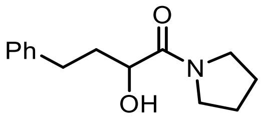

To a solution of amide **1a** (43.5 mg, 0.2 mmol, 1.0 eq.) and 2-iodopyridine (46.8 µL, 0.44 mmol, 2.2 eq.) in dichloromethane (2 mL, 0.1M) at 0 °C was added trifluoromethanesulfonic anhydride (37 µL, 0.22 mmol, 1.1 eq.) and the resulting mixture was stirred at 0 °C and colouration of the solution was observed. After 15 min, 2,6-lutidine *N*-oxide (22.4 µL, 0.2 mmol, 1.0 eq.) was added in one portion and the reaction mixture was stirred at 0 °C for 5 min. After this time, and the observation of colour-change, tetrabutyl ammonium hydroxide (480 mg, 0.6 mmol, 3 eq.). The resulting mixture was allowed to warm to ambient temperature (25 °C) over the course of 1 h, after which time a saturated aqueous solution of ammonium chloride (5 mL) was added. The biphasic mixture was separated and the aqueous phase was extracted with dichloromethane (2 × 5 mL). The combined organic phases were dried over anhydrous sodium sulfate, the dried solution was filtered and the filtrate was concentrated under reduced pressure. The crude residue was purified by silica gel flash chromatography (EtOAc/heptanes 1:9 to 2:3) to afford the compound **5** (28.0 mg. 60%) as a yellow oil with R_f = 0.35 (acetone/heptanes 3:7); **^{1}H NMR (600 MHz, CDCl$_3$):** δ 7.31 – 7.24 (m, 2H), 7.23 – 7.17 (m, 3H), 4.15 (td, *J* = 8.8, 2.7 Hz, 1H), 3.73 (d, *J* = 7.3 Hz, 1H), 3.57 – 3.50 (m, 1H), 3.43 – 3.38 (m, 1H), 3.31 – 3.25 (m, 1H), 3.18 – 3.12 (m, 1H), 2.88 – 2.82 (m, 1H), 2.81 – 2.75 (m, 1H), 1.96 – 1.78 (m, 6H); **^{13}C NMR (151 MHz, CDCl$_3$)**: δ 172.8, 141.6, 128.7 (2C), 128.5 (2C), 126.1,

68.5, 46.4, 45.9, 36.2, 31.3, 26.2, 24.0; **HRMS (ESI+):** exact mass calculated for $[M+Na]^+$ ($C_{14}H_{19}NNaO_2$) requires *m/z* 256.1308, found *m/z* 256.1304.

All analytical data were in good accordance with data reported in the literature.[10]

4-Phenyl-2-(phenylselanyl)-1-(pyrrolidin-1-yl)butan-1-one (6)

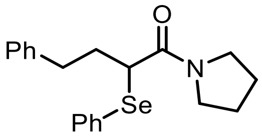

To a solution of amide **1a** (43.5 mg, 0.2 mmol, 1.0 eq.) and 2-iodopyridine (46.8 µL, 0.44 mmol, 2.2 eq.) in dichloromethane (2 mL, 0.1M) at 0 °C was added trifluoromethanesulfonic anhydride (37 µL, 0.22 mmol, 1.1 eq.) and the resulting mixture was stirred at 0 °C and colouration of the solution was observed. After 15 min, a previously prepared solution **B** (see below) was added and the reaction mixture was stirred at 0 °C for 5 min. The resulting mixture was allowed to warm to ambient temperature (25 °C) over the course of 1 h, after which time a saturated aqueous solution of ammonium chloride (5 mL) was added. The biphasic mixture was separated and the aqueous phase was extracted with dichloromethane (2 × 5 mL). The combined organic phases were dried over anhydrous sodium sulfate, the dried solution was filtered and the filtrate was concentrated under reduced pressure. The crude residue was purified by silica gel flash chromatography (EtOAc/heptanes 1:9 to 2:3) to afford compound **6** (23 mg, 64%) as a yellow oil with R_f 0.50 (acetone/heptanes 3:7).

Preparation of solution **B**: To a solution of the diselenide (187 mg, 0.6 mmol, 3 eq.) in dichloromethane (1 mL) was added the hydrogen peroxide solution (30% solution, 61.3 µL, 0.6 mmol 3 equiv.) at room temperature. The mixture was stirred 20 minutes until a yellow solid has appeared. Anhydrous magnesium sulfate (120 mg) was added. The mixture was stirred 10 more minutes before addition; **^{1}H NMR (600 MHz, CDCl$_3$)**: δ 7.55 (dd, *J* = 8.0, 1.1 Hz, 2H), 7.33 – 7.29 (m, 1H), 7.27 – 7.23 (m, 4H), 7.17 (t, *J* = 7.4 Hz, 1H), 7.11 (d, *J* = 7.1 Hz, 2H), 3.67 (dd, *J* = 9.3, 5.5 Hz, 1H), 3.48 – 3.43 (m, 1H), 3.40 – 3.34 (m, 1H), 3.12 – 3.07 (m, 1H), 3.00 – 2.95 (m, 1H), 2.79 – 2.73 (m, 1H), 2.65 – 2.58 (m, 1H), 2.51 – 2.44 (m, 1H), 2.13 – 2.05 (m, 1H), 1.81 – 1.66 (m, 4H); **^{13}C NMR (151 MHz, CDCl$_3$):** δ 169.6, 141.1, 136.0 (2C), 129.1 (2C), 128.6 (2C), 128.6, 128.5 (2C), 127.7, 126.1, 46.4, 46.1, 42.1, 34.2, 33.8, 26.0, 24.3; **^{77}Se NMR (115 MHz, CDCl$_3$):** δ 413.1; **IR (neat) ν_{max}:** 3057; 3025; 2968, 2948, 2926, 2870, 1633, 1578, 1495, 1476, 1427, 1341, 1254, 1227, 1190, 1168, 1022, 741, 696; **HRMS (ESI+):** exact mass calculated for $[M+Na]^+$ ($C_{20}H_{23}NOSeNa$) requires *m/z* 396.0837, found *m/z* 396.0837.

(*E*)-3-Cyclopentyl-1-(pyrrolidin-1-yl)prop-2-en-1-one (4b)

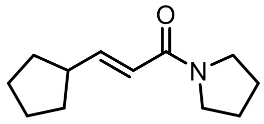

The product was prepared according to General Procedure C on 0.2 mmol scale. Following purification by silica gel flash chromatography (EtOAc/heptanes 1:9 to 2:3), the product was isolated as a colourless oil (25.9 mg, 67% yield) with R_f = 0.22 (acetone/heptanes 2:3); **^{1}H NMR (600 MHz, CDCl$_3$):** δ 6.89 (dd, *J* = 14.1, 8.2 Hz, 1H), 6.06 (d, *J* = 14.5 Hz, 1H), 3.51 (d, *J* = 6.2 Hz, 4H), 2.63 – 2.52 (m, 1H), 1.99 – 1.91 (m, 2H), 1.89 – 1.77 (m, 4H), 1.73 – 1.66 (m, 2H), 1.63 – 1.55 (m, 2H), 1.46 – 1.37 (m, 2H); **^{13}C NMR (151 MHz, CDCl$_3$):** δ 165.2, 150.2, 119.8, 46.6, 45.9, 43.2, 32.8 (2C), 26.3, 25.4 (2C), 24.5; **IR (neat) ν_{max}:** 2949, 2868, 1735, 1659, 1614, 1424, 1337, 1226, 1193, 981; **HRMS (ESI+):** exact mass calculated for $[M+Na]^+$ ($C_{12}H_{19}NNaO$) requires *m/z* 216.1364, found *m/z* 216.1354.

(*E*)-3-(4-Bromophenyl)-*N*,*N*-dimethylacrylamide (4c)

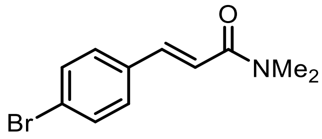

The product was prepared according to General Procedure C on 0.2 mmol scale. Following purification by silica gel flash chromatography (acetone/heptanes 1:9 to 1:5), the product was isolated as a white solid (43 mg, 85%) with R_f = 0.32 (acetone/heptanes 2:3); **^{1}H NMR (400 MHz, CDCl$_3$)**: δ 7.60 (d, *J* = 15.5 Hz, 1H), 7.52 – 7.47 (m, 2H), 7.41 – 7.36 (m, 2H), 6.87 (d, *J* = 15.4 Hz, 1H), 3.17 (s, 3H), 3.07 (s, 3H); **^{13}C NMR (151 MHz, CDCl$_3$):** δ 166.5, 141.2, 134.4, 132.1 (2C), 129.4 (2C), 123.8, 118.2, 37.6, 36.1; **IR (neat) ν_{max}:** 2920, 2851, 1647, 1610, 1584, 1561, 1485, 1388, 1134, 1066, 983, 810, 799; **HRMS (ESI+):** exact mass calculated for $[M+Na]^+$ ($C_{11}H_{12}BrNNaO$) requires *m/z* 275.9994, found *m/z* 275.9990.

***N*,*N*-Dimethylcinnamamide (4d)**

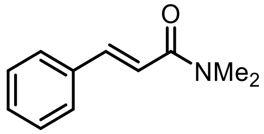

The product was prepared according to General Procedure C on 0.2 mmol scale. Following purification by silica gel flash chromatography (EtOAc/heptanes 1:4 to 1:1) and a further purification on preparative TLC (acetone/heptanes 2:3), the product was isolated as an off-white solid (24.0 mg, 69%) with R_f = 0.16 (acetone/heptanes 2:3); **^{1}H NMR (600 MHz, CDCl$_3$):** δ 7.67 (d, *J* = 15.4 Hz, 1H), 7.53 (dd, *J* = 8.0, 1.2 Hz, 2H), 7.40 – 7.32 (m, 3H), 6.89 (d, *J* = 15.4 Hz, 1H), 3.18 (s, 3H), 3.07 (s, 3H);

^{13}C NMR (151 MHz, CDCl$_3$): δ 166.8, 142.5, 135.5, 129.7, 128.9 (2C), 127.9 (2C), 117.6, 37.6, 36.1; **IR (neat) ν_{max}:** 3058, 3026, 2924, 2854, 1713, 1651, 1611, 1498, 1449, 1414, 1394, 1257, 1141, 977, 763, 706, 685, 569, 526, 485; **HRMS (ESI+):** exact mass calculated for $[M+Na]^+$ ($C_{11}H_{13}NNaO$) requires *m/z* 198.0895, found *m/z* 198.0886.

***N*,*N*-Diethylcinnamamide (4e)**

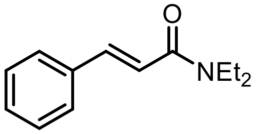

The product was prepared according to General Procedure C on 0.2 mmol scale. Following purification by silica gel flash chromatography (EtOAc/heptanes 1:5 to 4:6) and a further purification on preparative TLC (acetone/heptanes 1:1), the product was isolated as yellow oil (31.0 mg, 76%) with R_f = 0.29 (acetone/heptanes 2:3); **^{1}H NMR (400 MHz, CDCl$_3$):** δ 7.71 (d, *J* = 15.4 Hz, 1H), 7.56 – 7.49 (m, 2H), 7.41 – 7.33 (m, 3H), 6.83 (d, *J* = 15.4 Hz, 1H), 3.56 – 3.39 (m, 4H), 1.26 (t, *J* = 6.9 Hz, 3H), 1.19 (t, *J* = 7.0 Hz, 3H); **^{13}C NMR (101 MHz, CDCl$_3$):** δ 165.9, 142.5, 135.7, 129.6, 128.9 (2C), 127.9 (2C), 118.0, 42.5, 41.2, 15.2, 13.4; **IR (neat) ν_{max}:** 3079, 3059, 3027, 2973, 2931, 2874, 1740, 1648, 1607, 1453, 1429, 1245, 1139, 763; **HRMS (ESI+):** exact mass calculated for $[M+H]^+$ ($C_{13}H_{18}NO$) requires *m/z* 302.1751, found *m/z* 302.1750.

(1H-Inden-2-yl)(pyrrolidin-1-yl)methanone (4f)

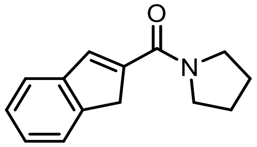

The product was prepared according to General Procedure C on 0.2 mmol scale. Following purification by silica gel flash chromatography (acetone/heptanes 1:5 to 2:3) and a further purification on preparative TLC (acetone/heptanes 1:1), the product was isolated as an off-white solid (21.0 mg, 63%) with R_f = 0.29 (acetone/heptanes 2:3); **^{1}H NMR (600 MHz, CDCl$_3$)**: δ 7.50 (d, *J* = 7.1 Hz, 1H), 7.46 (d, *J* = 6.8 Hz, 1H), 7.34 – 7.26 (m, 2H), 7.22 (s, 1H), 3.80 (d, *J* = 1.1 Hz, 2H), 3.72 (t, *J* = 6.3 Hz, 2H), 3.64 (t, *J* = 6.5 Hz, 2H), 2.01 – 1.91 (m, 4H); **^{13}C NMR (151 MHz, CDCl$_3$):** δ 165.6, 143.6, 143.6, 142.2, 134.9, 126.8, 126.6, 124.1, 122.6, 49.0, 46.9, 40.7, 26.8 24.3; **IR (neat) ν_{max}:** 3051, 2957, 2876, 1716, 1594, 1551, 1457, 1421, 1384, 1218, 1121, 1016, 920, 750, 710; **HRMS (ESI+):** exact mass calculated for $[M+Na]^+$ ($C_{14}H_{15}NNaO$) requires *m/z* 236.1046, found *m/z* 236.1043.

Cyclobut-1-en-1-yl(pyrrolidin-1-yl)methanone (4g)

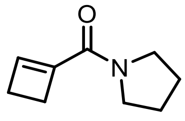 The product was prepared according to General Procedure C on 1.0 mmol scale. Following purification by silica gel flash chromatography (acetone/heptanes 1:5 to 2:3) and a further purification on preparative TLC (acetone/heptanes 1:1), the product was isolated as a colourless oil (95.0 mg, 63%) with R_f = 0.32 (acetone/heptanes 2:3); **^{1}H NMR (600 MHz, CDCl$_3$)**: δ 6.44 (s, 1H), 3.55 (t, J = 6.9 Hz, 2H), 3.50 (t, J = 6.9 Hz, 2H), 2.82 – 2.75 (m, 2H), 2.44 – 2.40 (m, 2H), 1.97 – 1.91 (m, 2H), 1.87 – 1.77 (m, 2H); **^{13}C NMR (151 MHz, CDCl$_3$):** δ 161.9, 142.4, 141.2, 47.0, 46.4, 30.9, 27.0, 26.5, 24.0; **IR (neat) ν_{max}:** 2964, 2926, 2871, 1721, 1617, 1571, 1418, 1340, 1227, 1190, 914, 865, 729, 865; **HRMS (ESI+):** exact mass calculated for $[M+Na]^+$ ($C_9H_{13}NNaO$) requires m/z 174.0889, found m/z 174.0887.

(*E*)-1-(Piperidin-1-yl)penta-2,4-dien-1-one (4h)

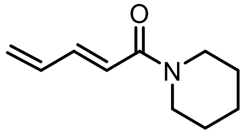 The product was prepared according to General Procedure C on 0.2 mmol scale. Following purification by silica gel flash chromatography (EtOAc/heptanes 1:10 to 2:3) and a further purification on preparative TLC (acetone/heptanes 1:1), the product was isolated as a colourless oil (13.2 mg, 40%) with R_f = 0.34 (acetone/heptanes 2:3); **^{1}H NMR (400 MHz, CDCl$_3$):** δ 7.25 – 7.16 (m, 1H), 6.53 – 6.35 (m, 2H), 5.59 – 5.49 (m, 1H), 5.43 – 5.36 (m, 1H), 3.68 – 3.46 (m, J = 32.2, 25.4 Hz, 4H), 1.69 – 1.62 (m, 2H), 1.62 – 1.54 (m, 4H); **^{13}C NMR (151 MHz, CDCl$_3$):** δ 165.4, 142.5, 135.5, 123.7, 121.8, 47.1, 43.4, 26.9, 25.7, 24.8; **IR (neat) ν_{max}:** 3086, 3056, 3001, 2933, 2854, 1781, 1754, 1648, 1611, 1437, 1410, 1265, 1137, 1121, 1019, 852; **HRMS (ESI+):** exact mass calculated for $[M+H]^+$ ($C_{10}H_{16}NO$) requires m/z 166.1226, found m/z 166.1232.

(*E*)-1-(Pyrrolidin-1-yl)undec-2-en-10-yn-1-one (4i)

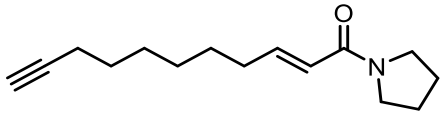 The product was prepared according to General Procedure C on 0.2 mmol scale. Following purification by silica gel flash chromatography (EtOAc/heptanes 1:10 to 3:7) and a further purification on preparative TLC (acetone/heptanes 1:1), the product was isolated as a yellow oil (21.0 mg, 46%) with R_f = 0.35 (acetone/heptanes 1:2); **^{1}H NMR (600 MHz, CDCl$_3$)**: δ 6.94 – 6.84 (m, 1H),

6.08 (dt, *J* = 15.1, 1.4 Hz, 1H), 3.53 – 3.48 (m, 4H), 2.22 – 2.14 (m, 4H), 1.98 – 1.90 (m, 3H), 1.88 – 1.81 (m, 2H), 1.54 – 1.48 (m, 2H), 1.48 – 1.42 (m, 2H), 1.42 – 1.37 (m, 2H), 1.35 – 1.30 (m, 2H); **^{13}C NMR (151 MHz, CDCl$_3$):** δ 165.0, 145.7, 121.8, 84.7, 68.3, 46.6, 45.9, 32.4, 28.7, 28.6, 28.5, 28.4, 26.2, 24.4, 18.5; **IR (neat) ν$_{max}$:** 3297, 3221, 3040, 2969, 2929, 2112, 1659, 1610, 1424, 1420, 1337, 1226, 1191, 980, 691, 626; **HRMS (ESI+):** exact mass calculated for [M+Na]$^+$ ($C_{15}H_{23}NNaO$) requires *m/z* 256.1672, found *m/z* 256.1668.

(*E*)-4,4,4-Trifluoro-1-(pyrrolidin-1-yl)but-2-en-1-one (4j)

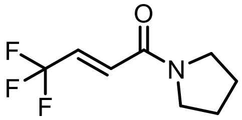

The product was prepared according to General Procedure C on 0.2 mmol scale. Following purification by silica gel flash chromatography (acetone/heptanes 1:5 to 1:1) and a further purification on preparative TLC (DCM/methanol 200:3), the product was isolated as a yellow oil (23.0 mg, 60%) with R_f = 0.6 (acetone/heptanes 1:2); **^{1}H NMR (600 MHz, CDCl$_3$)**: δ 6.86 – 6.74 (m, 2H), 3.59 – 3.56 (m, 4H), 2.04 – 1.99 (m, 2H), 1.94 – 1.90 (m, 2H); **^{13}C NMR (151 MHz, CDCl$_3$):** δ 168.3, 129.0 (q, *J* = 5.5 Hz), 129.0 (q, *J* = 35 Hz), 122.8 (q, *J* = 270 Hz), 46.9, 46.4, 26.2, 24.4; **^{19}F NMR (565 MHz, CDCl$_3$)**: δ -64.9 (d, *J* = 5.0 Hz); **IR (neat) ν$_{max}$:** 2929, 2881, 1723, 1684, 1626, 1447, 1344; **HRMS (ESI+):** exact mass calculated for [M+H]$^+$ ($C_8H_{11}NOF_3$) requires *m/z* 194.0787, found *m/z* 194.0788.

***N*,*N*-Dibenzylcyclobut-1-ene-1-carboxamide (4k)**

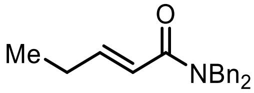

The product was prepared according to General Procedure C on 0.2 mmol scale. Following purification by silica gel flash chromatography (EtOAc/heptanes 1:5 to 1:1) and a further purification on preparative TLC (acetone/heptanes 1:1), the product was isolated as a yellow oil (32.3 mg, 58%) with R_f = 0.38 (acetone/heptanes 2:3); **^{1}H NMR (400 MHz, CDCl$_3$):** δ 7.42 – 7.15 (m, 10H), 7.11 (dt, *J* = 15.0, 6.5 Hz, 1H), 6.27 (dt, *J* = 15.0, 1.6 Hz, 1H), 4.65 (s, 2H), 4.51 (s, 2H), 2.27 – 2.18 (m, 2H), 1.04 (t, *J* = 7.4 Hz, 3H); **^{13}C NMR (101 MHz, CDCl$_3$):** δ 167.6, 149.4, 137.6, 137.0, 129.0 (2C), 128.7 (2C), 128.5 (2C), 127.7, 127.5, 126.7 (2C), 119.4, 50.0, 48.6, 25.8, 12.8; **IR (neat) ν$_{max}$:** 3085, 3062, 3028, 2964, 2930, 2874, 1654, 1618, 1495, 1419, 1359, 1278, 1294, 1079, 972, 952, 747, 732, 695, 613, 531, 457; **HRMS (ESI+):** exact mass calculated for [M+H]$^+$ ($C_{19}H_{22}NO$) requires *m/z* 280.1701, found *m/z* 280.1699.

(*E*)-1-Morpholinobut-2-en-1-one (4l)

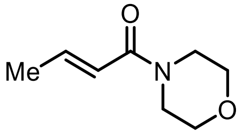

The product was prepared according to General Procedure C on 0.2 mmol scale. Following purification by silica gel flash chromatography (acetone/heptanes 1:4 to 1:1) and a further purification on preparative TLC (acetone/heptanes 2:3), the product was isolated as an orange solid (15.1 mg, 49%) with R_f = 0.4 (acetone/heptanes 1:2); **^{1}H NMR (600 MHz, $CDCl_3$)**: δ 6.94 – 6.87 (m, 1H), 6.23 (dq, *J* = 15.0, 1.6 Hz, 1H), 3.62 (d, *J* = 75.2 Hz, 8H), 1.89 (dd, *J* = 6.9, 1.7 Hz, 3H); **^{13}C NMR (151 MHz, $CDCl_3$):** δ 165.8, 142.3, 121.1, 66.9, 46.2, 42.3, 18.4; **IR (neat) ν_{max}:** 2961, 2851, 1646, 1549, 1530; **HRMS (ESI+):** exact mass calculated for $[M+H]^+$ ($C_8H_{14}NO_2$) requires *m/z* 156.1019, found *m/z* 156.1019.

(*E*)-1-(Piperidin-1-yl)but-2-en-1-one (4m)

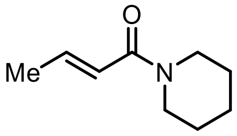

The product was prepared according to General Procedure C on 0.2 mmol scale. Following purification by silica gel flash chromatography (EtOAc/heptanes 1:9 to 2:3) and a further purification on preparative TLC (acetone/heptanes 1:1), the product was isolated as a yellow oil (14.7 mg, 48%) with R_f = 0.38 (acetone/heptanes 2:3); **^{1}H NMR (600 MHz, $CDCl_3$):** δ 6.83 (dq, *J* = 14.9, 6.8 Hz, 1H), 6.27 (dq, *J* = 14.9, 1.7 Hz, 1H), 3.59 (s, 2H), 3.48 (s, 2H), 1.87 (dd, *J* = 6.8, 1.7 Hz, 3H), 1.68 – 1.63 (m, 2H), 1.58 – 1.54 (m, 4H); **^{13}C NMR (151 MHz, $CDCl_3$):** δ 165.7, 140.9, 122.1, 47.0, 43.2, 26.8, 25.7, 24.8, 18.4; **IR (neat) ν_{max}:** 3051, 3000, 2931, 2853, 1735, 1660, 1611, 1432, 1283, 1249, 1218, 1140, 1101, 1024, 964, 972, 852; **HRMS (ESI+):** exact mass calculated for $[M+H]^+$ ($C_9H_{16}NO$) requires *m/z* 154.1229, found *m/z* 154.1229.

(*E*)-1-(Azepan-1-yl)but-2-en-1-one (4n)

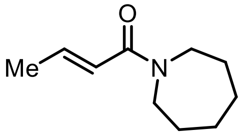

The product was prepared according to General Procedure C on 0.2 mmol scale. Following purification by silica gel flash chromatography (acetone/heptanes 1:5 to 1:1), the product was isolated as a yellow oil (16.8 mg, 51%) with R_f = 0.6 (acetone/heptanes 1:2); **^{1}H NMR (600 MHz, $CDCl_3$)**: δ 6.90 (dq, *J* = 20.7, 6.9 Hz, 1H), 6.32 – 6.17 (m, 1H), 3.59 – 3.54 (m, 2H), 3.50 – 3.46 (m, 2H), 1.87 (dd, *J* =

6.9, 1.7 Hz, 3H), 1.76 – 1.68 (m, 4H), 1.60 – 1.51 (m, 4H); **^{13}C NMR (151 MHz, CDCl$_3$):** δ 166.5, 141.1, 122.1, 47.9, 46.3, 29.4, 27.7, 27.1, 26.7, 18.3; **IR (neat) ν$_{max}$:** 2925, 2854, 1659, 1607, 1481, 1448, 1425; **HRMS (ESI+): HRMS (ESI+):** exact mass calculated for [M+Na]$^+$ ($C_{10}H_{17}NNaO$) requires *m/z* 190.1202, found *m/z* 190.1200.

(*E*)-*N*-Methyl-*N*-phenylpent-2-enamide (4o)

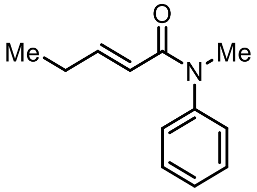

The product was prepared according to General Procedure C on 0.2 mmol scale. Following purification by silica gel flash chromatography (EtOAc/heptanes 1:9 to 3:7), the product was isolated as a pale yellow oil (25.2 mg, 67%) with R_f = 0.38 (acetone/heptanes 2:3); **^{1}H NMR (600 MHz, CDCl$_3$):** δ 7.40 (t, *J* = 7.7 Hz, 2H), 7.32 (t, *J* = 7.4 Hz, 1H), 7.17 (d, *J* = 7.3 Hz, 2H), 6.93 (dt, *J* = 15.1, 6.6 Hz, 1H), 5.72 (d, *J* = 15.1 Hz, 1H), 3.33 (s, 3H), 2.13 – 2.01 (m, 2H), 0.92 (t, *J* = 7.4 Hz, 3H); **^{13}C NMR (151 MHz, CDCl$_3$):** δ 166.4, 147.5, 143.9, 129.6 (2C), 127.5 (3C), 120.6, 37.5, 25.5, 12.7; **IR (neat) ν$_{max}$:** 3061, 3037, 3011, 2964, 2931, 2875, 2856, 1726, 1661, 1629, 1595, 1495, 1419, 1369, 1291, 1119, 1025, 975, 851, 772, 700, 664, 567; **HRMS (ESI+):** exact mass calculated for [M+H]$^+$ ($C_{12}H_{16}NO$) requires *m/z* 190.1226, found *m/z* 190.1224.

(*E*)-*N*-(4-Methoxyphenyl)-*N*-methylbut-2-enamide (4p)

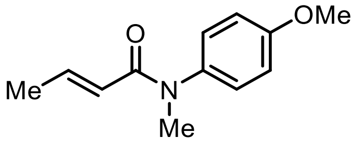

The product was prepared according to General Procedure C on 0.2 mmol scale. Following purification by silica gel flash chromatography (EtOAc/heptanes 1:9 to 3:7), the product was isolated as a yellow oil (27.1 mg, 66%) with R_f = 0.26 (acetone/heptanes 2:3); **^{1}H NMR (700 MHz, CDCl$_3$)**: δ 7.09 – 7.06 (m, 2H), 6.93 – 6.86 (m, 3H), 5.73 (d, *J* = 15.0 Hz, 1H), 3.83 (s, 3H), 3.28 (s, 3H), 1.72 (d, *J* = 6.3 Hz, 3H); **^{13}C NMR (176 MHz, CDCl$_3$):** δ 166.5, 158.8, 141.0, 136.7, 128.6, 128.5, 122.8, 114.8 (2C), 55.6, 37.6, 18.1; **IR (neat) ν$_{max}$:** 3048, 3018, 3001, 2958, 2925, 2875, 2851, 2841, 1730, 1664, 1629, 1509, 1444, 1369, 1288, 1243, 1170, 1129, 1104, 1029, 965, 837, 766, 732, 680, 608, 567; **HRMS (ESI+):** exact mass calculated for [M+H]$^+$ ($C_{12}H_{16}NO_2$) requires *m/z* 206.1176, found *m/z* 206.1177.

(*E*)-*N*-Allyl-*N*-benzylbut-2-enamide (4q)

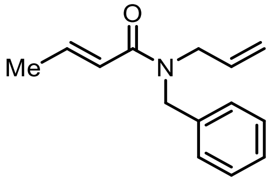

The product was prepared according to General Procedure C on 0.2 mmol scale. Following purification by silica gel flash chromatography (acetone/heptanes 1:5 to 1:2), and a further purification on preparative TLC (acetone/heptanes 1:2), the product was isolated as a yellow oil (17.1 mg, 40%) with R_f = 0.60 (acetone/heptanes 1:2); **^{1}H NMR (700 MHz, CDCl$_3$)**: (rotameric effects present) δ 7.36 (t, *J* = 7.2 Hz, 1H), 7.32 – 7.28 (m, 1H), 7.25 (m, 1H), 7.19 (d, *J* = 7.3 Hz, 1H), 7.01 (dq, *J* = 13.6, 6.7 Hz, 1H), 6.23 (d, *J* = 14.9 Hz, 1H), 5.84 – 5.71 (m, 1H), 5.23 – 5.10 (m, 2H), 4.64 – 4.54 (m, 2H), 4.04 (d, *J* = 5.5 Hz, 1H), 3.88 (d, *J* = 3.3 Hz, 1H), 1.87 (dd, *J* = 34.7, 6.7 Hz, 3H); **^{13}C NMR (151 MHz, CDCl$_3$):** δ 167.2, 167.1, 142.8, 142.5, 137.8, 137.2, 133.2, 133.0, 129.0, 128.7, 128.4, 127.7, 127.4, 126.6, 121.8, 121.8, 117.6, 117.0, 50.1, 49.1, 48.8, 48.3, 18.4; **IR (neat) ν_{max}**; 2969, 2929, 1661, 1619, 1447, 1416; **HRMS (ESI+):** exact mass calculated for $[M+H]^+$ ($C_{14}H_{18}NO$) requires *m/z* 216.1383, found *m/z* 216.1380.

Methyl (E)-9-(azepan-1-yl)-9-oxonon-7-enoate (4r)

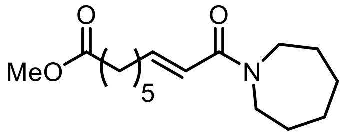

The product was prepared according to General Procedure C at -20°C (NaCl and ice bath ratio of 1:3) on 0.2 mmol scale. Following purification by silica gel flash chromatography (acetone/heptanes 1:5 to 1:1), the product was isolated as a yellow oil (45.5 mg, 81%) with R_f = 0.35 (acetone/heptanes 1:2); **^{1}H NMR (700 MHz, CDCl$_3$):** δ 6.90 – 6.83 (m, 1H), 6.21 (dt, *J* = 15.0, 1.4 Hz, 1H), 3.65 (s, 3H), 3.58 – 3.55 (m, 2H), 3.50 – 3.46 (m, 2H), 2.29 (t, *J* = 7.5 Hz, 2H), 2.23 – 2.17 (m, 2H), 1.72 (dq, *J* = 24.0, 6.1 Hz, 4H), 1.63 (dd, *J* = 15.3, 7.6 Hz, 2H), 1.58 – 1.53 (m, 4H), 1.47 (dt, *J* = 15.1, 7.5 Hz, 2H), 1.37 – 1.33 (m, 2H); **^{13}C NMR (151 MHz, CDCl$_3$):** δ 174.3, 166.6, 145.9, 120.8, 51.6, 51.58, 48.0, 46.4, 34.1, 32.4, 29.4, 28.8, 28.2, 27.8, 27.1, 26.7, 24.8; **IR (neat) ν_{max}:** 2929, 2856, 1736, 1657, 1613, 1430; **HRMS (ESI+):** exact mass calculated for $[M+H]^+$ ($C_{16}H_{28}NO_3$) requires *m/z* 282.2064 found *m/z* 282.2046.

Methyl (*E*)-9-(diethylamino)-9-oxonon-7-enoate (4s)

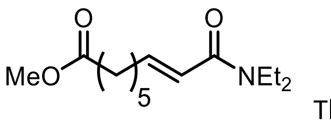 The product was prepared according to General Procedure C at -10 °C (acetone/ice bath) on 0.155 mmol scale. Following purification by silica gel flash chromatography (EtOAc/heptanes 1:5 to 1:2) and a further purification on preparative TLC (acetone/heptanes 1:1), the product was isolated as a colourless oil (23.3 mg, 59%) with R_f = 0.60 (acetone/heptanes 1:2); **^{1}H NMR (700 MHz, CDCl$_3$):** δ 6.90 – 6.86 (m, 1H), 6.18 (d, *J* = 15.0, 1H), 3.66 (s, 3H), 3.42 (q, *J* = 7.0 Hz, 2H), 3.36 (q, *J* = 7.0 Hz, 2H), 2.30 (t, *J* = 7.5 Hz, 2H), 2.21 (td, J = 8.0, 1.0 Hz, 2H), 1.66 – 1.61 (m, 2H), 1.50 – 1.44 (m, 2H), 1.38 – 1.33 (m, 2H), 1.19 (t, *J* = 7.0 Hz, 3H), 1.14 (t, *J* = 7.0 Hz, 3H); **^{13}C NMR (176 MHz, CDCl$_3$):** δ 174.3, 166.0, 145.9, 120.8, 51.6, 42.3, 40.9, 34.1, 32.4, 28.8, 28.2, 24.9, 15.0, 13.3; **IR (neat) ν_{max}:** 2971, 2930, 2857, 1735, 1659, 1617, 1430, 1361, 1253, 1219, 1201, 1172, 1136, 1097, 978; **HRMS (ESI+):** exact mass calculated for $[M+H]^+$ ($C_{14}H_{26}NO_3$) requires *m*/*z* 256.1907, found *m*/*z* 256.1907.

Ethyl (*E*)-9-(diisobutylamino)-9-oxonon-7-enoate (4t)

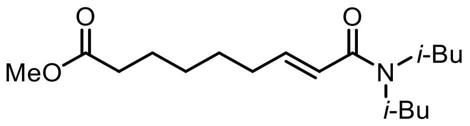 The product was prepared according to General Procedure C on 0.2 mmol scale. Following purification by silica gel flash chromatography (acetone/heptanes 1:5 to 1:2) and a further purification on preparative TLC (acetone/heptanes 1:1), the product was isolated as a yellow oil (32.0 mg, 55%) with R_f = 0.45 (acetone/heptanes 1:2); **^{1}H NMR (600 MHz, CDCl$_3$)**: δ 6.91 – 6.76 (m, 1H), 6.19 (d, *J* = 15.0 Hz, 1H), 3.64 (s, 3H), 3.21 (d, *J* = 7.5 Hz, 2H), 3.12 (d, *J* = 7.5 Hz, 2H), 2.28 (t, *J* = 7.5 Hz, 2H), 2.21 – 2.16 (m, 2H), 2.04 – 1.96 (m, 1H), 1.92 – 1.86 (m, 1H), 1.65 – 1.58 (m, 2H), 1.48 – 1.42 (m, 2H), 1.36 – 1.29 (m, 2H), 0.88 (d, *J* = 6.7 Hz, 6H), 0.86 (d, *J* = 6.7 Hz, 6H); **^{13}C NMR (151 MHz, CDCl$_3$):** δ 174.2, 167.0, 145.6, 121.0, 56.0, 54.5, 51.6, 34.0, 32.4, 28.9, 28.8, 28.1, 27.0, 24.8, 20.4 (2C), 20.2 (2C); **IR (neat) ν_{max}:** 2955, 2869, 1735, 1653, 1618, 1465, 1437, 1420, 1386, 1231, 1170, 1139, 1100, 977, 819; **HRMS (ESI+):** exact mass calculated for $[M+Na]^+$ ($C_{18}H_{33}NNaO_3$) requires *m*/*z* 334.2353, found *m*/*z* 334.2351.

Methyl (*E*)-9-(indolin-1-yl)-9-oxonon-7-enoate (4u)

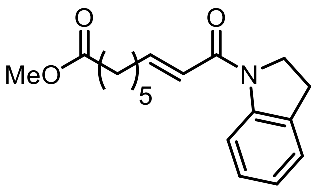The product was prepared according to General Procedure C on 0.2 mmol scale. Following purification by silica gel flash chromatography (EtOAc/heptanes 1:9 to 2:3) and a further purification on preparative TLC (acetone/heptanes 1:1), the product was isolated as a yellow oil (31.6 mg, 52%) with R_f = 0.34 (acetone/heptanes 2:3); **^{1}H NMR (600 MHz, CDCl$_3$):** δ 8.36 – 8.00 (m, 1H), 7.22 – 7.16 (m, 2H), 7.07 – 6.99 (m, 2H), 6.22 (d, *J* = 14.1 Hz, 1H), 4.15 (t, *J* = 8.5 Hz, 2H), 3.69 – 3.66 (m, 3H), 3.25 – 3.15 (m, 2H), 2.33 – 2.25 (m, 4H), 1.65 (dt, *J* = 15.2, 7.5 Hz, 2H), 1.55 – 1.49 (m, 2H), 1.38 (dt, *J* = 9.5, 7.6 Hz, 2H); **^{13}C NMR (151 MHz, CDCl$_3$):** δ 174.2, 147.5, 143.3, 131.5, 127.7, 124.6, 123.8, 122.3, 117.6, 51.6, 48.2, 34.1, 34.1, 32.4, 28.8, 28.1, 24.8; **IR (neat) ν_{max}:** 3061, 3028, 2998, 2926, 2853, 1735, 1661, 1624, 1595, 1479, 1461, 1400, 1338, 1291, 1260, 1199, 1167, 1119, 975, 754, 605, 580; **HRMS (ESI+):** exact mass calculated for $[M+H]^+$ ($C_{18}H_{24}NO_3$) requires *m/z* 302.1751, found *m/z* 302.1750.

(*E*)-*N*-Benzyl-*N*-methyl-10-oxoundec-2-enamide (4v)

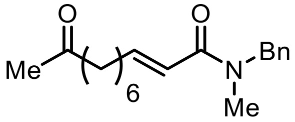The product was prepared according to General Procedure C on 0.2 mmol scale. Following purification by silica gel flash chromatography (EtOAc/heptanes 1:9 to 2:3) and a further purification on preparative TLC (acetone/heptanes 1:1), the product was isolated as a yellow oil (37.7 mg, 63%) with R_f = 0.55 (acetone/heptanes 2:3); **^{1}H NMR (600 MHz, CDCl$_3$):** δ 7.38 – 7.26 (m, 3H), 7.26 – 7.15 (m, 2H), 7.00 – 6.90 (m, 1H), 6.25 (dd, *J* = 33.2, 15.0 Hz, 1H), 4.66 (d, *J* = 16.6 Hz, 1H), 4.57 (d, *J* = 19.6 Hz, 1H), 2.98 (s, 3H), 2.46 – 2.35 (m, 2H), 2.22 (dd, *J* = 14.2, 7.2 Hz, 1H), 2.19 – 2.06 (m, 4H), 1.60 – 1.50 (m, 2H), 1.46 (dd, *J* = 14.7, 7.3 Hz, 1H), 1.41 (dd, *J* = 14.4, 7.3 Hz, 1H), 1.36 – 1.28 (m, 4H); **^{13}C NMR (151 MHz, CDCl$_3$):** δ 209.3, 167.5, 167.0, 147.1, 147.0, 137.6, 137.0, 129.0, 128.7, 128.2, 127.7, 127.4, 126.6, 120.4, 120.3, 53.5, 51.2, 43.8, 35.0, 34.2, 32.6, 32.5, 30.0, 29.1, 29.1, 29.0, 28.3, 28.3, 23.8; **IR (neat) ν_{max}:** 3061, 3028, 2998, 2926, 2853, 1712, 1657, 1618, 1495, 1452, 1400, 1357, 1122, 1029, 975, 735, 700; **HRMS (ESI+):** exact mass calculated for $[M+H]^+$ ($C_{19}H_{28}NO_2$) requires *m/z* 302.2115, found *m/z* 302.2109.

(*E*)-7-Oxo-7-(pyrrolidin-1-yl)hept-5-enenitrile (4w)

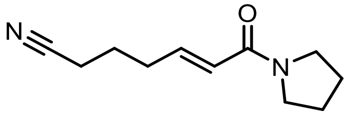
The product was prepared according to General Procedure C on 0.2 mmol scale. Following purification by silica gel flash chromatography (EtOAc/heptanes 1:9 to 2:3) and a further purification on preparative TLC (acetone/heptanes 1:1), the product was isolated as a white crystalline solid (29.0 mg, 73%) with R_f = 0.22 (acetone/heptanes 1:2); **^{1}H NMR (600 MHz, CDCl$_3$)**: δ 6.79 (dt, *J* = 14.7, 7.2 Hz, 1H), 6.19 (dt, *J* = 15.1, 1.4 Hz, 1H), 3.51 (t, *J* = 6.9 Hz, 4H), 2.41 – 2.34 (m, 4H), 1.99 – 1.92 (m, 2H), 1.90 – 1.79 (m, 4H); **^{13}C NMR (151 MHz, CDCl$_3$):** δ 164.3, 142.2, 123.9, 119.3, 46.6, 46.0, 30.9, 26.2, 24.4, 24.1, 16.6; **IR (neat) ν_{max}:** 2970, 2951, 2940, 2876, 2244, 1717, 1660, 1599, 1430, 1338, 1227, 1191, 975; **HRMS (ESI+):** exact mass calculated for [M+Na]$^+$ ($C_{11}H_{16}N_2NaO$) requires *m/z* 215.1155, found *m/z* 215.1150.

(2*E*,13*Z*)-1-(Pyrrolidin-1-yl)docosa-2,13-dien-1-one (4x)

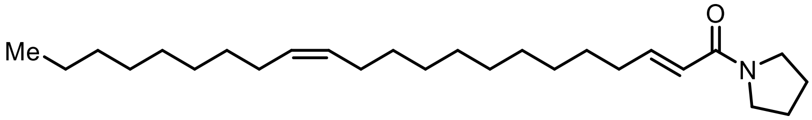
The product was prepared according to General Procedure C on 0.2 mmol scale. Following purification by silica gel flash chromatography (acetone/heptanes 1:5 to 2:1), the product was isolated as a yellow oil (56.8 mg, 72%) with R_f = 0.45 (acetone/heptanes 1:2); **^{1}H NMR (600 MHz, CDCl$_3$)**: 6.93 – 6.89 (m, 1H), 6.08 (d, *J* = 15.2 Hz, 1H), 5.62 (m, 1H), 5.44 (dd, *J* = 15.4, 7.2 Hz, 1H), 4.03 (m, 1H), 3.52 (m, 3H), 2.20 (dt, *J* = 15.2, 7.7 Hz, 2H), 2.02 (m, 2H), 1.96 (dt, *J* = 13.8, 7.0 Hz, 2H), 1.86 (m, 2H), 1.47 – 1.42 (m, 2H), 1.26 (m, 26H), 0.88 (t, *J* = 6.4Hz, 3H); **^{13}C NMR (101 MHz, CDCl$_3$) δ**: 165.1, 145.9, 130.0, 130.0, 121.7, 46.6, 45.8, 32.5, 32.0, 29.9, 29.6, 29.5, 29.4, 29.3, 28.5, 27.3, 26.2, 24.4, 22.8, 14.2; **IR (neat) ν_{max}:** 2924, 2853, 1660, 1606, 1439. **HRMS (ESI+):** exact mass calculated for [M+Na]$^+$ ($C_{26}H_{47}NNaO$) requires *m/z* 412.3550, found *m/z* 412.3549.

(8*R*,9*S*,10*S*,13*R*,14*S*,17*R*)-10,13-Dimethyl-17-((*R*,*E*)-5-oxo-5-(pyrrolidin-1-yl)pent-3-en-2-yl)dodecahydro-3H-cyclopenta[a]phenanthrene-3,7,12(2H,4H)-trione (4y)

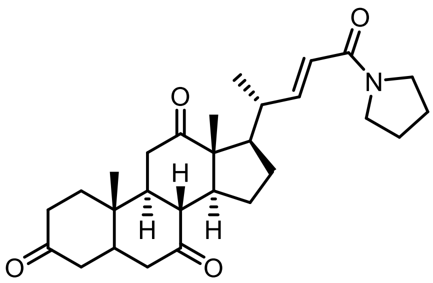

The product was prepared according to General Procedure C on 0.2 mmol scale. Following purification by silica gel flash chromatography (EtOAc/MeOH 1:0 to 95:5), the product, as an inseparable mixture with starting material (15%), was isolated (74.0 mg, 67%) with R_f = 0.15 (EtOAc); **^{1}H NMR (600 MHz, CDCl$_3$):** δ 6.88 (dd, *J* = 15.1, 8.7 Hz, 1H), 6.06 (d, *J* = 15.1 Hz, 1H), 3.54 – 3.49 (m, 4H), 2.95 – 2.78 (m, 3H), 2.39 – 1.82 (m, 18H), 1.62 (td, *J* = 14.2, 4.8 Hz, 1H), 1.43 – 1.27 (m, 5H), 1.08 (s, 3H), 1.05 (d, *J* = 6.8 Hz, 3H); **^{13}C NMR (151 MHz, CDCl$_3$):** δ 211.8, 209.1, 208.8, 165.0, 150.0, 120.4, 56.9, 51.2, 49.0, 47.0, 46.7, 46.0, 45.6, 45.3, 45.1, 42.9, 39.6, 38.7, 36.7, 36.1, 35.4, 26.7, 26.3, 25.1, 24.5, 22.1, 20.5, 12.6; **IR (neat) ν_{max}:** 2962, 2931, 2873, 1705, 1657, 1611, 1430, 1387; **HRMS (ESI+):** exact mass calculated for $[M+Na]^+$ ($C_{28}H_{39}NNaO_4$) requires *m/z* 476.2777, found *m/z* 476.2790.

(*E*)-3-(5-Cyano-1-(4-fluorophenyl)-1,3-dihydroisobenzofuran-1-yl)-*N*,*N*-dimethylacrylamide (4z)

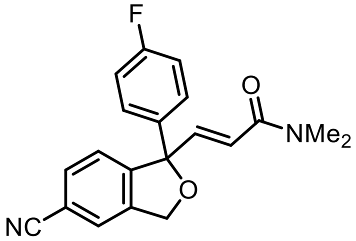

The product was prepared according to General Procedure C on both 0.2 mmol and 4.18 mmol scale. Following purification by silica gel flash chromatography (acetone/heptanes 1:5 to 1:1), the product was isolated as an off-white foamy solid (46.7 mg, 69% or 884 mg, 63%) with R_f = 0.39 (acetone/heptanes 1:1); **^{1}H NMR (400 MHz, CDCl$_3$):** δ 7.64 – 7.60 (m, 1H), 7.57 (s, 1H), 7.34 – 7.28 (m, 3H), 7.23 (d, *J* = 14.8 Hz, 1H), 7.05 – 7.00 (m, 2H), 6.67 (d, *J* = 14.8 Hz, 1H), 5.26 (d, *J* = 12.9 Hz, 1H), 5.18 (d, *J* = 12.9 Hz, 1H), 3.08 (s, 3H), 3.01 (s, 3H); **^{13}C NMR (101 MHz, CDCl$_3$):** δ 165.8, 162.7 (d, *J* = 248 Hz), 147.6, 144.2, 140.0, 137.4 (d, *J* = 3.2 Hz, 2C), 132.3, 128.6 (d, *J* = 8.3 Hz), 125.5, 124.0, 119.8, 118.6, 115.7 (d, *J* = 21.6 Hz, 2C), 112.5, 90.6, 71.5, 37.6, 36.0; **^{19}F NMR (565 MHz, CDCl$_3$):** δ -113.4; **IR (neat) ν_{max}:** 3051, 2929, 2860, 2230, 1781, 1657, 1615, 1506, 1410, 1396, 1265, 1225, 1160, 1141, 1085, 1029, 1013, 978, 910, 832, 729, 700, 613, 597, 589, 559, 518; **HRMS (ESI+):** exact mass calculated for $[M+H]^+$ ($C_{20}H_{18}FN_2O_2$) requires *m/z* 337.1343, found *m/z* 337.1347.

(*E*)-1-Methylazacyclotridec-3-en-2-one (4aa)

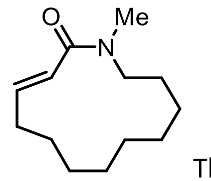

The product was prepared according to General Procedure C on 0.2 mmol scale. Following purification by silica gel flash chromatography (EtOAc/heptanes 1:5 to 1:1) and a further purification on preparative TLC (acetone/heptanes 1:1), the product was isolated as a yellow oil (17.1 mg, 40%) with R_f = 0.26 (acetone/heptanes 2:3); **^{1}H NMR (400 MHz, CDCl$_3$)**: δ 6.71 – 6.58 (m, 1H), 6.28 (dt, *J* = 15.3, 1.2 Hz, 1H), 3.38 (t, *J* = 6.6 Hz, 2H), 2.94 (s, 3H), 2.28 – 2.20 (m, 2H), 1.64 – 1.56 (m, 2H), 1.55 – 1.46 (m, 2H), 1.39 – 1.21 (m, 10H); **^{13}C NMR (101 MHz, CDCl$_3$):** δ 168.4, 144.1, 123.0, 48.4, 33.3, 33.0, 26.9 – 26.5 (m), 26.2, 25.4, 25.4, 23.4; **IR (neat) ν_{max}:** 2924, 2856, 1735, 1654, 1614, 1438, 1395, 1198, 1168, 1106, 976, 820, 742, 701; **HRMS (ESI+):** exact mass calculated for $[M+Na]^+$ ($C_{13}H_{23}NNaO$) requires *m/z* 232.1672, found *m/z* 232.1670.

(*E*)-*N*-Cyclohexyl-9-oxo-9-(pyrrolidin-1-yl)non-7-enamide (4ab)

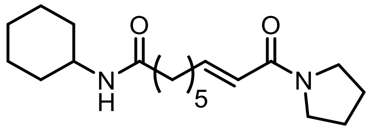

The product was prepared according to General Procedure C at -20°C (NaCl and ice bath ratio of 1/3) on 0.1 mmol scale. Following purification by silica gel flash chromatography (acetone/heptanes 1:2 to 4:1), the product was isolated as a yellow oil (16.3 mg, 51%) with R_f = 0.40 (acetone/heptanes 3:2); **^{1}H NMR (400 MHz, CDCl$_3$):** δ 6.90 – 6.71 (m, 1H), 6.02 (dt, *J* = 15.1, 1.5 Hz, 1H), 5.23 – 5.21 (m, *J* = 6.0 Hz, 1H), 3.69 (dt, *J* = 10.6, 9.3 Hz, 1H) , 3.45 (dd, *J* = 11.8, 6.7 Hz, 3H), 2.16 – 2.10 (m, 2H), 2.07 – 2.03 (m, 2H), 1.92 – 1.85 (m, 2H), 1.82 – 1.78 (m, 2H), 1.65 – 1.60 (m, 2H), 1.58 – 1.52 (m, 2H), 1.43 – 1.33 (m, 2H), 1.32 – 1.22 (m, 6H), 1.11 – 1.02 (m, 2H), 0.81 – 0.78 (m, 2H); **^{13}C NMR (151 MHz, CDCl$_3$):** δ 172.1, 165.1, 145.7, 121.9, 48.2, 46.7, 46.0, 37.1, 33.4, 32.3, 29.9, 28.8, 28.2, 26.3, 25.7, 25.0, 24.5; **IR (neat) ν_{max}:** 2925, 2854, 1736, 1637, 1426, 1357; **HRMS (ESI+):** exact mass calculated for $[M+H]^+$ ($C_{19}H_{33}N_2O_2$) requires *m/z* 321.2537 found *m/z* 321.2531.

4.3. Application of our methodology

4.3.1. *Dihydroxylation*

Procedure: Diol **7** was prepared following the procedure reported by Sharpless *et al.* starting from 0.3 mmol of the desaturated amide **4q**.[11]

1-(Azepan-1-yl)-2,3-dihydroxybutan-1-one (7)

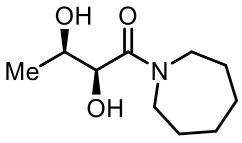

(±) Following purification by silica gel flash chromatography (acetone/heptanes 1:4 to 2:1), the product was isolated as a yellow oil (40.0 mg, 69%) with R_f = 0.35 (acetone/heptanes 1:2); **^{1}H NMR (700 MHz, MeOD)**: δ 4.24 (d, *J* = 4.6 Hz, 1H), 3.94 (qd, *J* = 6.4, 4.7 Hz, 1H), 3.69 – 3.62 (m, 2H), 3.55 (ddd, *J* = 14.5, 7.0, 5.3 Hz, 1H), 3.42 (ddd, *J* = 13.7, 8.0, 4.4 Hz, 1H), 2.91 (d, *J* = 15.7 Hz, 1H), 2.80 (d, *J* = 15.7 Hz, 1H), 1.80 – 1.70 (m, 4H), 1.60 (tdd, *J* = 16.6, 12.0, 4.2 Hz, 4H), 1.18 (d, *J* = 6.4 Hz, 3H); **^{13}C NMR (176 MHz, MeOD):** δ 173.4, 73.7, 69.8, 47.7, 43.8, 30.3, 28.4, 28.1, 27.3, 19.2; **IR (neat) ν_{max}:** 3411, 3301, 3260, 2929, 2857, 1723, 1608, 1442, 1440, 1373, 1200; **HRMS (ESI+):** exact mass calculated for $[M+H]^+$ ($C_{10}H_{20}NO_3$) requires *m/z* 202.1438 found *m/z* 202.1438.

4.3.2. *Enantioselective Michael addition*

Procedure: Amide **8** was prepared following the procedure reported by Harutyunyan *et al.* starting from 0.2 mmol of the desaturated amide **4n**.[12]

(*S*)-1-(Azepan-1-yl)-3-methylpentan-1-one (8)

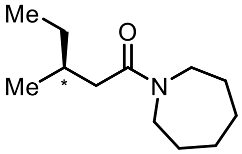

Following purification by silica gel flash chromatography (acetone/heptanes 1:5 to 1:1), the compound was isolated as a yellow oil (36.0 mg, 91%) with R_f = 0.6 (acetone/heptanes 1:2); **^{1}H NMR (600 MHz, $CDCl_3$)**: δ 3.56 – 3.50 (m, 2H), 3.47 – 3.41 (m, 2H), 2.29 (dd, *J* = 14.7, 5.9 Hz, 1H), 2.12 (dd, *J* = 14.7, 8.0 Hz, 1H), 1.98 (dd, *J* = 13.4, 6.8 Hz, 1H), 1.70 (dd, *J* = 11.3, 5.7 Hz, 4H), 1.58 - 1.57 (m, 3H), 1.43 – 1.37 (m, 1H), 1.30 – 1.14 (m, 2H), 0.95 – 0.88 (m, 6H); **^{13}C NMR (151 MHz, $CDCl_3$):** δ 172.4, 48.1, 46.1, 40.3, 32.0, 29.8, 29.4, 27.8, 27.1, 26.9, 19.6, 11.6; **IR (neat) ν_{max}:** 2958, 2927, 2855, 1638, 1453, 1424; **HRMS (ESI+):** exact mass calculated for $[M+H]^+$ ($C_{12}H_{24}NO$) requires *m/z* 198.1852, found *m/z* 198.1850.

Report

Sample Information

Sample Name	: Carlos_CR813F1_Lux-1
Sample ID	:
Vial#	: 2
Injection Volume	: 6
Data File	: Carlos_CR813F1_Lux-1_18.07.2018_1_002.lcd
Method File	: Run_100DD_F1_Pos4.lcm
Batch File	: 18.07.2018_1.lcb
Report Format File	: Lux-Cellulose-1(OD-H)_98Hep_2IPA_F1.lsr
Date Acquired	: 18.07.2018 11:39:01
Date Processed	: 18.07.2018 12:26:45

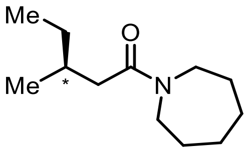

Method Description:
Column:Lux-Cellulose1 (Chiralcel OD-H) 250x4,6mm
Solvent System: n-Heptan+0,1%IPA/IPA 98:2
Flow:1 ml/min

Chromatogram

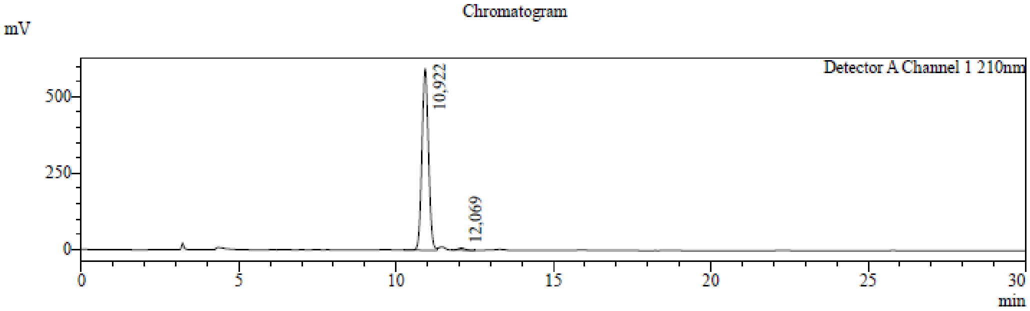

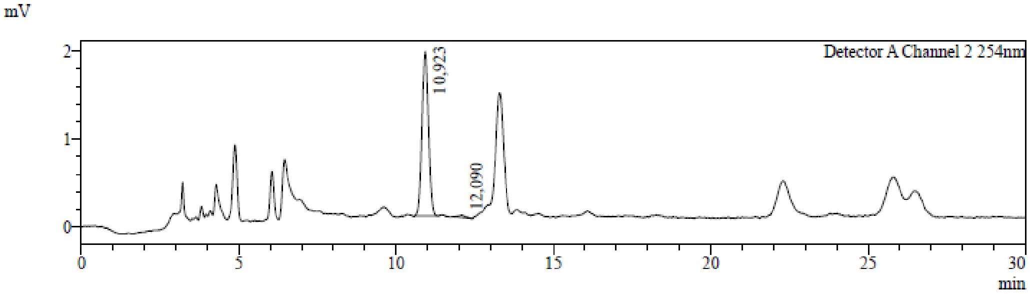

Peak Table

Detector A Channel 1 210nm

Peak#	Ret. Time	Area	Area%
1	10.922	8667989	98.845
2	12.069	101288	1.155
Total		8769278	100.000

Detector A Channel 2 254nm

Peak#	Ret. Time	Area	Area%
1	10.923	27011	98.863
2	12.090	311	1.137
Total		27322	100.000

Report

Sample Information

Sample Name	: Carlos_CR810F1_Rac_Lux-1
Sample ID	:
Vial#	: 1
Injection Volume	: 6
Data File	: Carlos_CR810F1_Rac_Lux-1_18.07.2018_1_003.lcd
Method File	: Run_100DD_F1_Pos4.lcm
Batch File	: 18.07.2018_1.lcb
Report Format File	: Lux-Cellulose-1(OD-H)_98Hep_2IPA_F1.lsr
Date Acquired	: 18.07.2018 12:09:26
Date Processed	: 18.07.2018 12:29:00

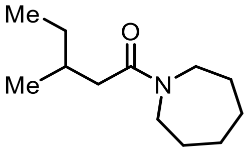

(±)

Method Description:
Column:Lux-Cellulose1 (Chiralcel OD-H) 250x4,6mm
Solvent System: n-Heptan+0,1%IPA/IPA 98:2
Flow:1 ml/min

Chromatogram

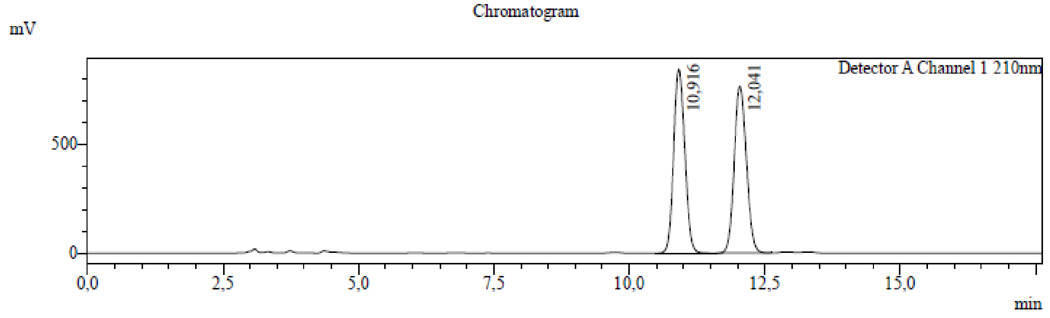

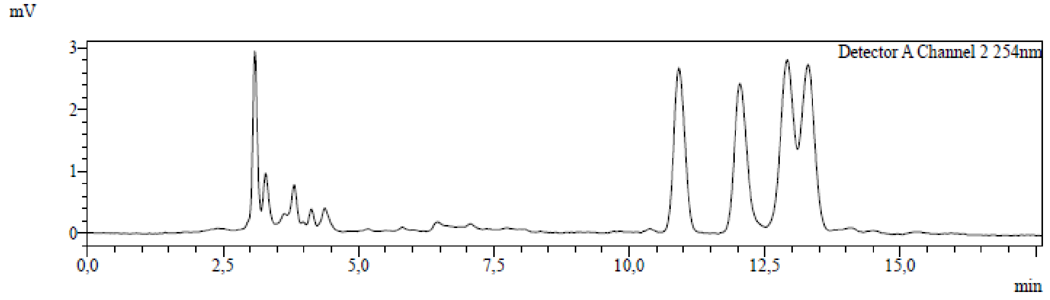

Peak Table

Detector A Channel 1 210nm

Peak#	Ret. Time	Area	Area%
1	10,916	12019868	50,050
2	12,041	11995984	49,950
Total		24015852	100,000

Detector A Channel 2 254nm

Peak#	Ret. Time	Area	Area%
Total			

4.3.3. Synthesis of piperine

Procedure: Allyl alcohol **10** was prepared following the procedure reported by Quayle *et al.*[13]

1-(Benzo[d][1,3]dioxol-5-yl)prop-2-en-1-ol (10)

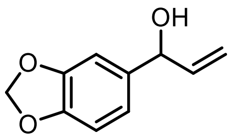

Following purification by silica gel flash chromatography (acetone/heptanes 1:5 to 1:1), the product was isolated as a yellow oil (1.78 g, 96%) with R_f = 0.55 (acetone/heptanes 1:2); **^{1}H NMR (600 MHz, $CDCl_3$)**: δ 6.87 (d, *J* = 1.5 Hz, 1H), 6.83 (dd, *J* = 8.0, 1.3 Hz, 1H), 6.78 (d, *J* = 7.9 Hz, 1H), 6.02 (ddd, *J* = 17.1, 10.3, 5.8 Hz, 1H), 5.95 (s, 2H), 5.34 (dt, *J* = 17.1, 1.4 Hz, 1H), 5.19 (dt, *J* = 10.3, 1.3 Hz, 1H), 5.12 (d, *J* = 5.2 Hz, 1H).

All analytical data were in good accordance with data reported in the literature.[13]

Procedure: Amide **12** was prepared following the procedure reported by Trauner *et al.*[14]

(*E*)-5-(benzo[d][1,3]dioxol-5-yl)-1-(piperidin-1-yl)pent-4-en-1-one (12)

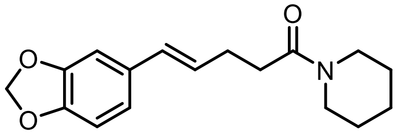

Following purification by silica gel flash chromatography (Acetone/heptanes 1:5 to 1:1), the compound was isolated as a yellow oil (1.05 g, 73%) with R_f = 0.35 (acetone/heptanes 1:2); **^{1}H NMR (600 MHz, $CDCl_3$)**: δ 6.89 (d, *J* = 1.5 Hz, 1H), 6.76 (dd, *J* = 8.0, 1.5 Hz, 1H), 6.73 (d, *J* = 8.0 Hz, 1H), 6.34 (d, *J* = 15.8 Hz, 1H), 6.09 (dt, *J* = 15.7, 6.9 Hz, 1H), 5.93 (s, 2H), 3.58 – 3.55 (m, 2H), 3.42 – 3.39 (m, 2H), 2.52 (ddd, *J* = 8.2, 7.1, 1.5 Hz, 2H), 2.48 – 2.45 (m, 2H), 1.66 – 1.62 (m, 2H), 1.54 (dd, *J* = 13.5, 7.6 Hz, 4H).

All analytical data were in good accordance with data reported in the literature.[15]

(2*E*,4*E*)-5-(benzo[d][1,3]dioxol-5-yl)-1-(piperidin-1-yl)penta-2,4-dien-1-one (13)

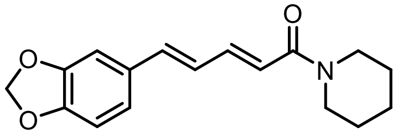

The product was prepared according to General Procedure C on 0.2 mmol scale. The yield of the reaction was determined to be 48% by NMR using mesitylene as an internal standard (24.0 mg, 2.0 eq.). Due to contamination with 2-iodobenzoic acid, a small sample was purified by preparative TLC (run twice in solvent system 3:1:1 heptane/ethyl acetate/dichloromethane) for analytical purposes; **^{1}H NMR (700 MHz, $CDCl_3$):** δ 7.40 (dd, *J* = 14.6, 9.6 Hz, 1H), 6.98 (d, *J* = 1.3 Hz, 1H),

6.89 (dd, *J* = 8.0, 1.4 Hz, 1H), 6.79 – 6.73 (m, 3H), 6.44 (d, *J* = 14.7 Hz, 1H), 5.98 (s, 2H), 3.64 (s, 2H), 3.53 (s, 2H), 1.66 (dd, *J* = 11.1, 5.8 Hz, 2H), 1.61 – 1.57 (m, 4H); **^{13}C NMR (176 MHz, $CDCl_3$):** δ 165.6, 148.4, 148.3, 142.6, 138.4, 131.2, 125.5, 122.7, 120.2, 108.7, 105.8, 101.4, 47.1, 43.4, 26.9, 25.8, 24.8; **HRMS (ESI+):** exact mass calculated for $[M+H]^+$ ($C_{17}H_{20}NO_3$) requires *m*/*z* 286.1438 found *m*/*z* 286.1437.

All analytical data were in good accordance with data reported in the literature.[16]

5. Labelling experiments

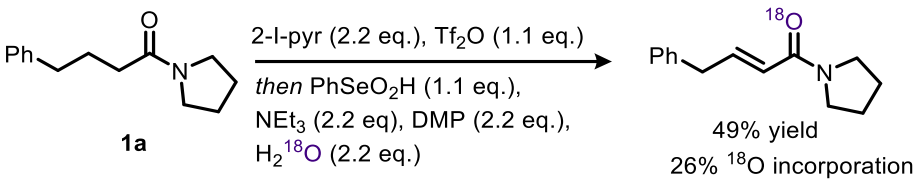

Mass Spectrum List Report

Analysis Info

		Acquisition Date	6/14/2018 3:33:25 PM
Analysis Name	E:\Data\MS_MessService_Archiv_2018_550_xxx\579\57967000001.d		
Method	tune_low_MS_Service_06_18.m	Operator	msc
Sample Name	PAAD935	Instrument / Ser#	maXis 16
Comment	Adler/Maulide<br>Ergebnis: +/- 5ppm<br>ACN/MeOH + 1% H2O		

Acquisition Parameter

Source Type	ESI	Ion Polarity	Positive	Set Nebulizer	0.4 Bar
Focus	Not active	Set Capillary	4500 V	Set Dry Heater	180 °C
Scan Begin	50 m/z	Set End Plate Offset	-500 V	Set Dry Gas	4.0 l/min
Scan End	1900 m/z	Set Collision Cell RF	500.0 Vpp	Set Divert Valve	Source

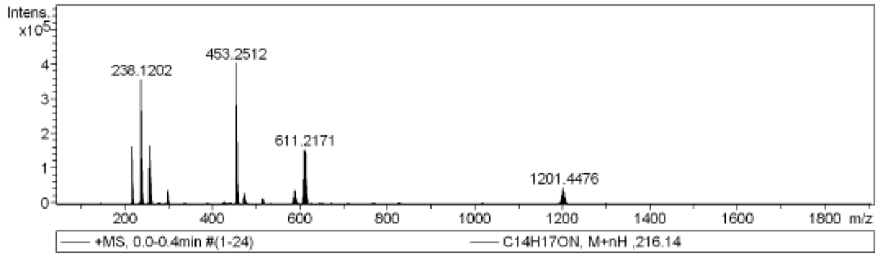

#	m/z	I	
1	216.1383	162791	[MH]$^+$ $C_{14}H_{18}N^{16}O^+$ 216.1383
2	218.1427	56173	[MH]$^+$ $C_{14}H_{18}N^{18}O^+$ 218.1425
3	238.1202	355781	[MNa]$^+$ $C_{14}H_{17}NNa^{16}O^+$ 238.1202
4	239.1234	55650	
5	240.1251	125918	[MNa]$^+$ $C_{14}H_{17}NNa^{18}O^+$ 240.1245
6	256.1307	100507	
7	258.1350	165374	
8	260.1392	73892	
9	298.1412	37390	
10	300.1455	34013	
11	453.2512	404600	
12	454.2543	126202	
13	455.2556	281359	
14	456.2587	85524	
15	457.2606	57608	
16	473.2658	29963	
17	589.2350	36918	
18	591.2369	36728	
19	609.2175	68266	
20	610.2210	40636	
21	611.2171	154528	
22	612.2202	57761	
23	613.2189	148903	
24	614.2219	51656	
25	615.2217	52799	
26	1199.4470	38746	
27	1200.4498	30068	
28	1201.4476	45182	
29	1202.4504	30533	
30	1203.4496	34004	

#	m/z	I
1	216.1383	2000
2	217.1416	314
3	218.1450	27

Bruker Compass DataAnalysis 4.0 printed: 7/18/2018 11:54:40 AM Page 1 of 1

6. References

[1] B. Peng, D. Geerdink, C. Farès, N. Maulide, *Angew. Chem. Int. Ed.* **2014**, *53*, 5462–5466.

[2] W. Yao, X. Ma, L. Guo, X. Jia, A. Hu, Z. Huang, *Tetrahedron Lett.* **2016**, *57*, 2919–2921.

[3] D. McConnell, M. Impagnatiello, D. Kessler, O. Kraemer, S. Schneider, L. Van Der Veen, U. Weyer-Czernilofsky, T. Wunberg, *Thiazolyl-Dihydro-Indazoles*, **2009**, WO/2009/112565.

[4] F. Buxton, T. Ehara, P. Ganju, A. Hallett, O. Irie, A. Iwasaki, T. Kanazawa, K. Masuya, K. Nonomura, J. Sakaki, et al., *2-Cyanopyrrolopyrimidines and Pharmaceutical Uses Thereof*, **2004**, WO/2004/069256.

[5] A. de la Torre, D. Kaiser, N. Maulide, *J. Am. Chem. Soc.* **2017**, *139*, 6578–6581.

[6] J. Britton, J. M. Chalker, C. L. Raston, *Chem. - Eur. J.* **2015**, *21*, 10660–10665.

[7] V. Tona, A. de la Torre, M. Padmanaban, S. Ruider, L. González, N. Maulide, *J. Am. Chem. Soc.* **2016**, *138*, 8348–8351.

[8] J.-S. Li, Y.-D. Da, G.-Q. Chen, Q. Yang, Z.-W. Li, F. Yang, P.-M. Huang, *ChemistrySelect* **2017**, *2*, 1770–1773.

[9] J. P. Célérier, M. G. Richaud, G. Lhommet, *Synthesis* **1983**, *1983*, 195–197.

[10] B. Ma, T. Miao, Y. Sun, Y. He, J. Liu, Y. Feng, H. Chen, Q.-H. Fan, *Chem. - Eur. J.* **2014**, *20*, 9969–9978.

[11] P. Dupau, R. Epple, A. A. Thomas, V. V. Fokin, K. B. Sharpless, *Adv. Synth. Catal.* **2002**, *344*, 421–433.

[12] M. Rodríguez-Fernández, X. Yan, J. F. Collados, P. B. White, S. R. Harutyunyan, *J. Am. Chem. Soc.* **2017**, *139*, 14224–14231.

[13] C. D. Edlin, J. Faulkner, M. Helliwell, C. K. Knight, J. Parker, P. Quayle, J. Raftery, *Tetrahedron* **2006**, *62*, 3004–3015.

[14] S. N. Gradl, J. J. Kennedy-Smith, J. Kim, D. Trauner, *Synlett* **2002**, 411–414.

[15] B. Das, A. Kashinatham, P. Madhusudhan, *Tetrahedron Lett.* **1998**, *39*, 677–678.

[16] P. S.-W. Leung, Y. Teng, P. H. Toy, *Org. Lett.* **2010**, *12*, 4996–4999.

7. Spectra

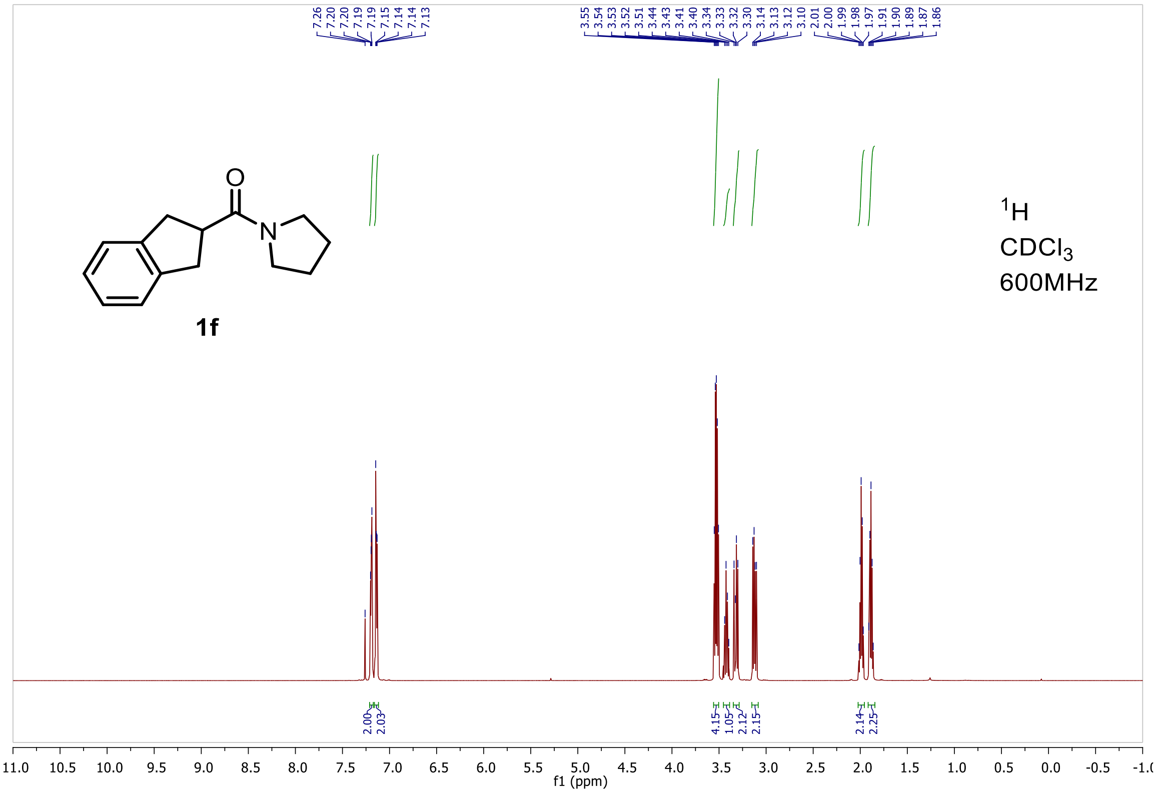

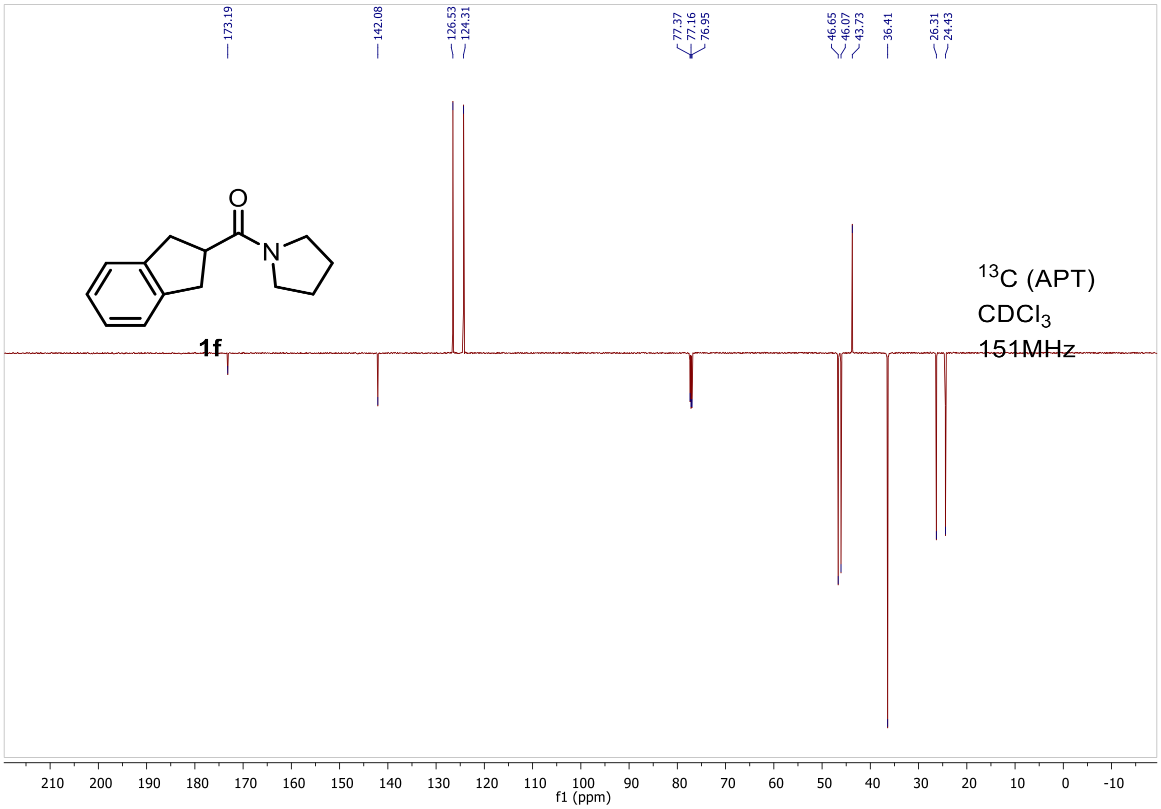

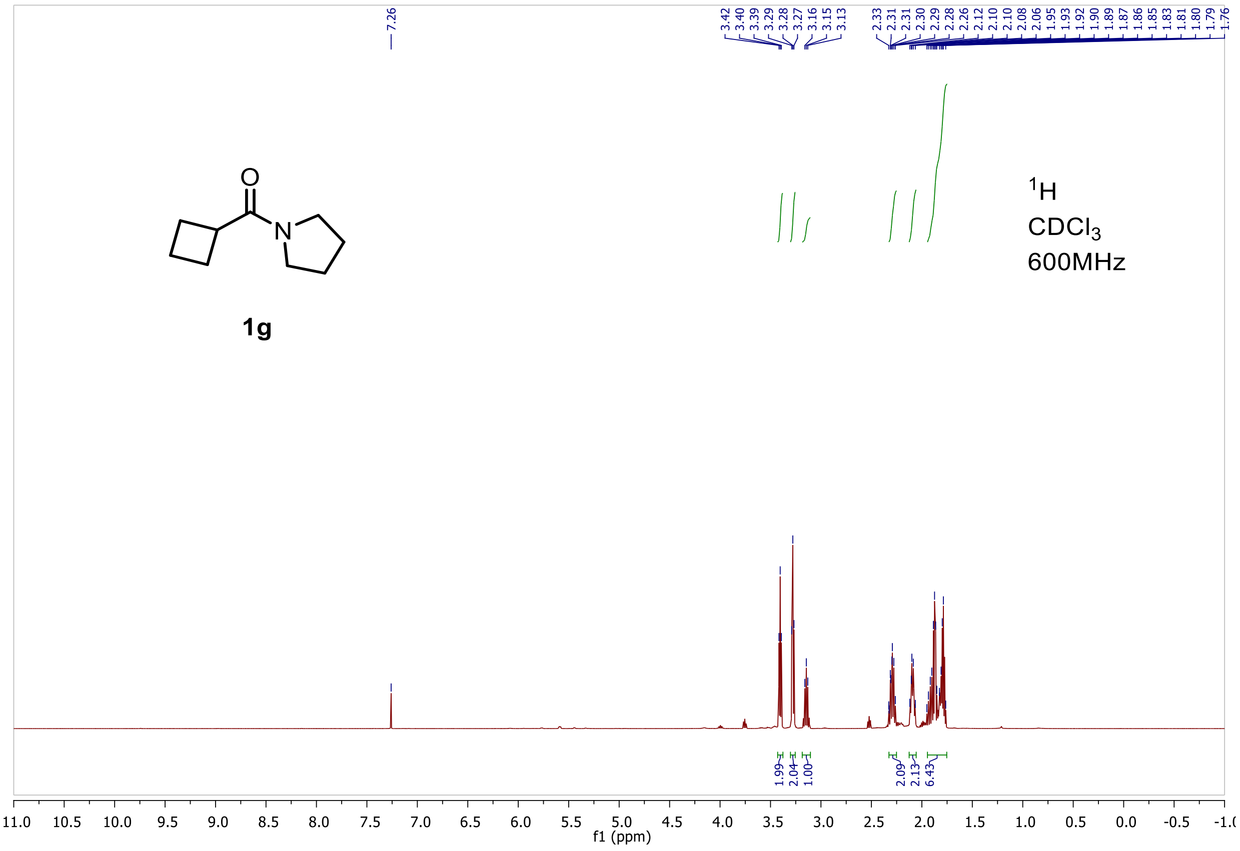
7.26
3.42 3.40 3.39 3.29 3.28 3.27 3.16 3.15 3.13
2.33 2.31 2.31 2.30 2.29 2.28 2.26 2.12 2.10 2.08 2.06 1.95 1.93 1.92 1.90 1.89 1.87 1.86 1.85 1.83 1.81 1.80 1.79 1.76
O
N
1g
^{1}H
$CDCl_3$
600MHz
1.99 2.04 1.00 2.09 2.13 6.43
f1 (ppm)

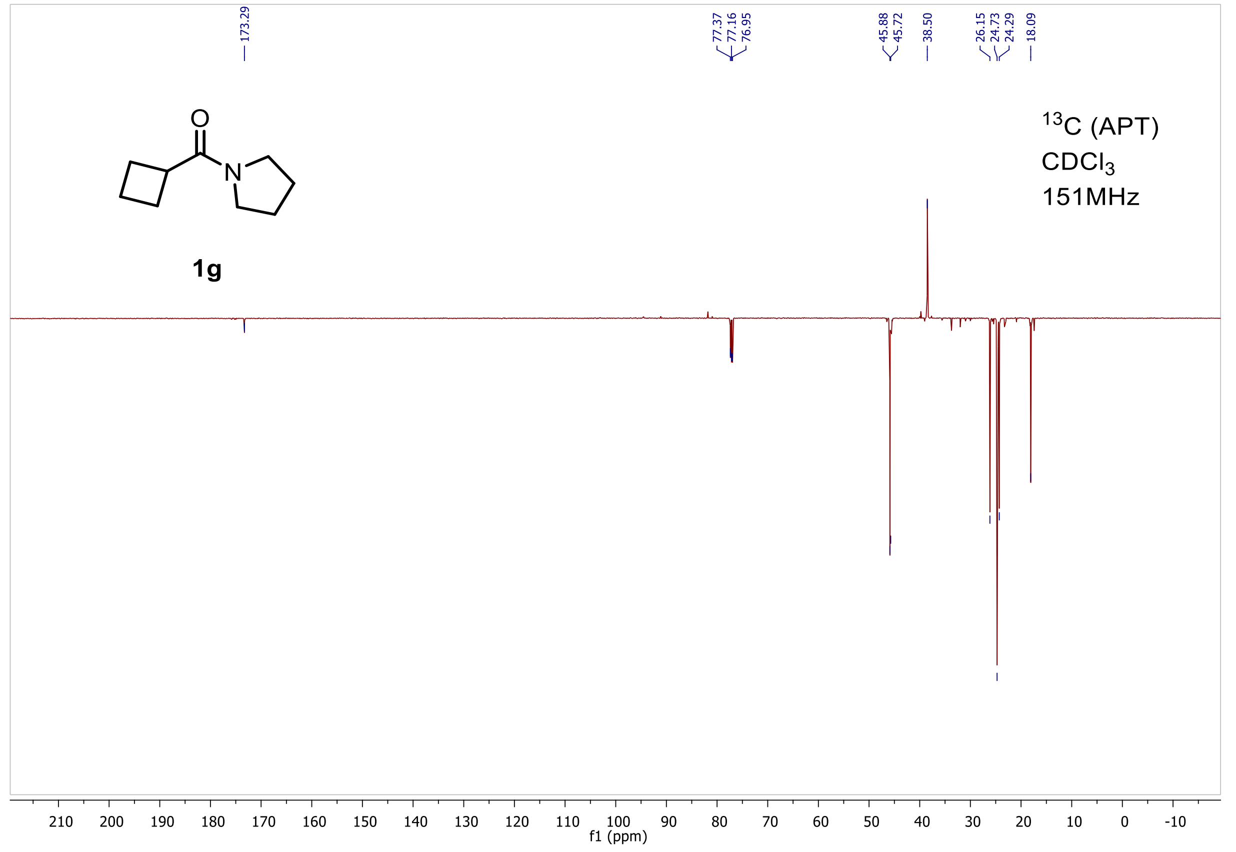
173.29
77.37 77.16 76.95
45.88 45.72 38.50
26.15 24.73 24.29 18.09
O
N
1g
^{13}C (APT)
$CDCl_3$
151MHz
f1 (ppm)

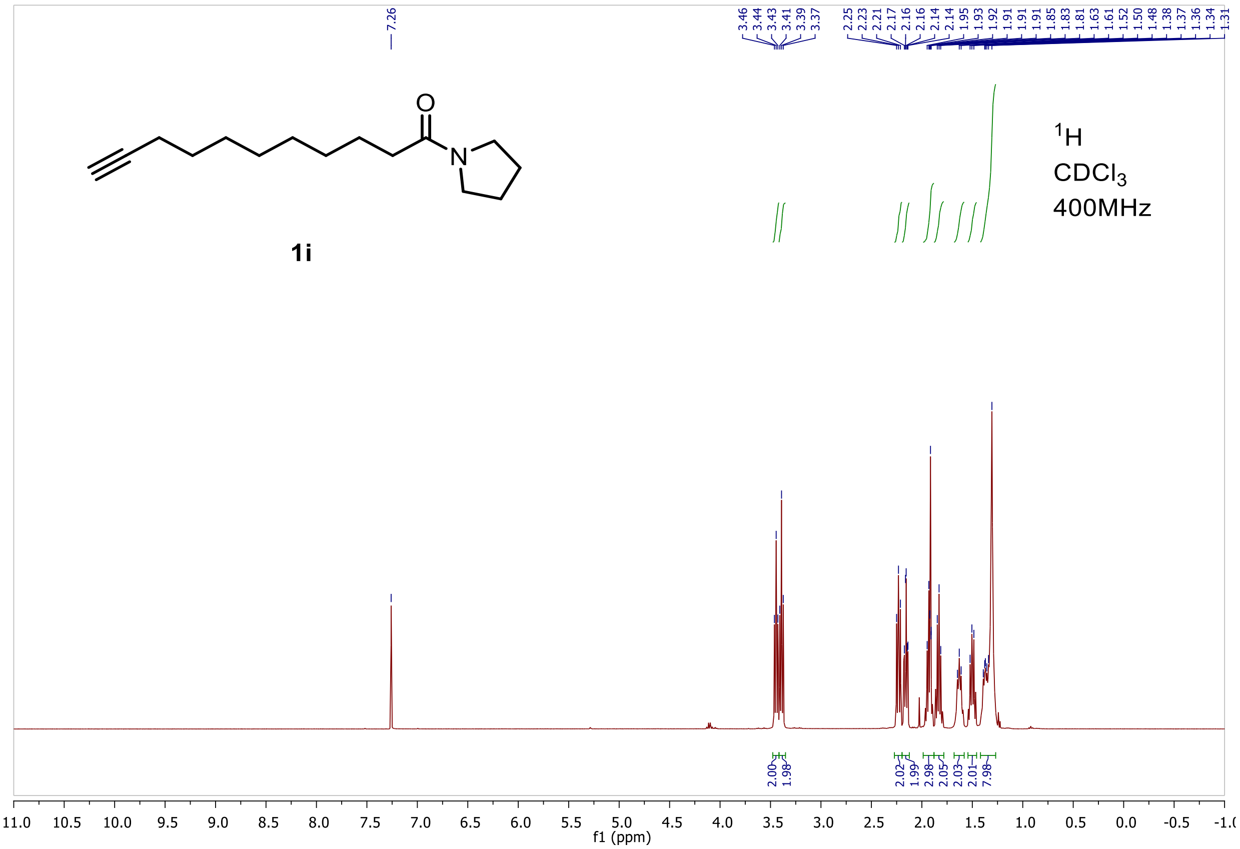
1i
1H
CDCl3
400MHz
f1 (ppm)

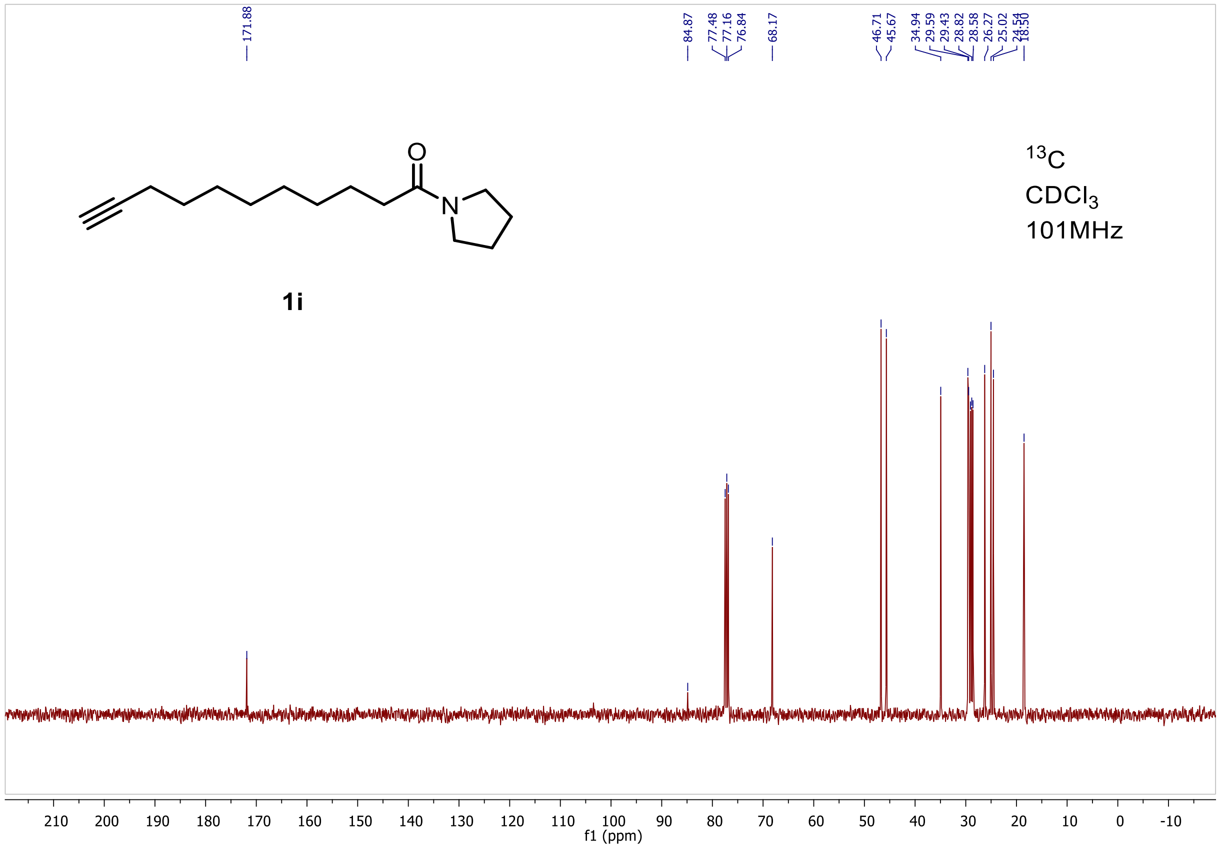
1i
13C
CDCl3
101MHz
f1 (ppm)

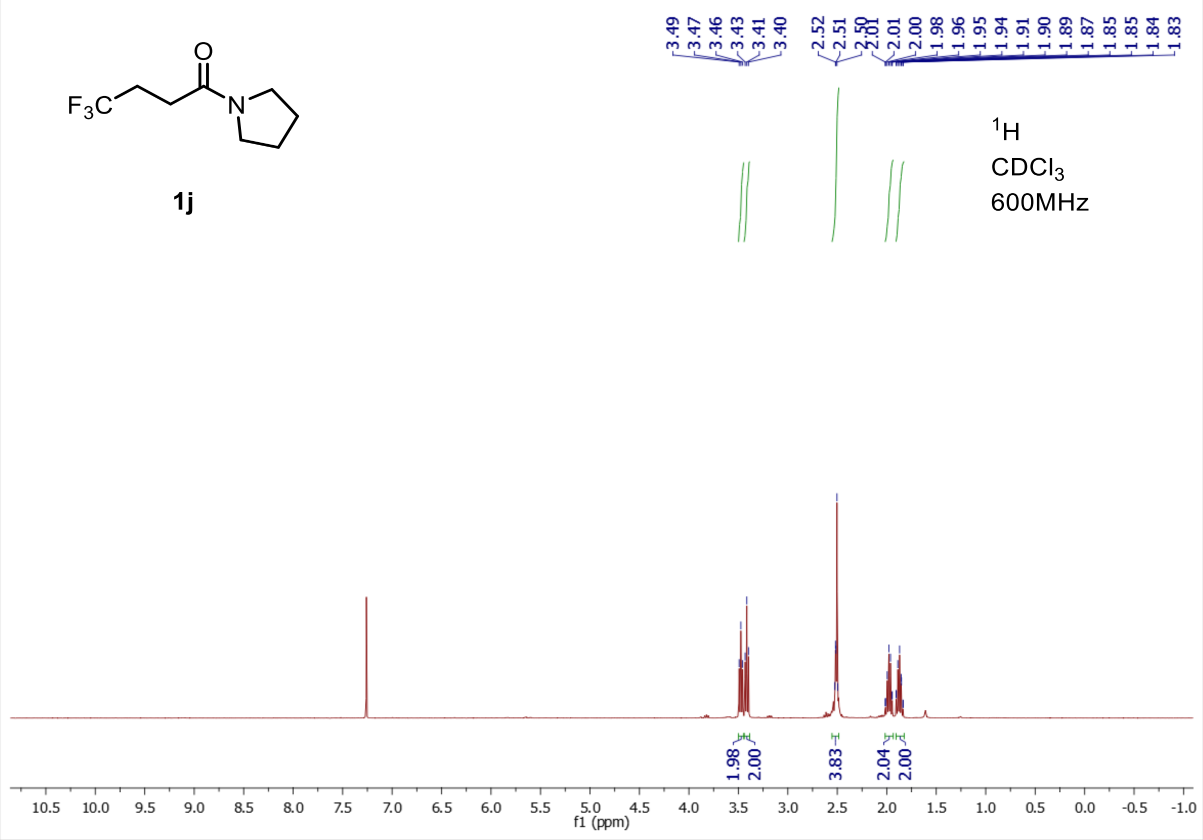
3.49 3.47 3.46 3.43 3.41 3.40 2.52 2.51 2.50 2.01 2.01 2.00 1.98 1.96 1.95 1.94 1.91 1.90 1.89 1.87 1.85 1.85 1.84 1.83
F3C
O
N
1j
1H
CDCl3
600MHz
1.98 2.00 3.83 2.04 2.00
10.5 10.0 9.5 9.0 8.5 8.0 7.5 7.0 6.5 6.0 5.5 5.0 4.5 4.0 3.5 3.0 2.5 2.0 1.5 1.0 0.5 0.0 -0.5 -1.0
f1 (ppm)

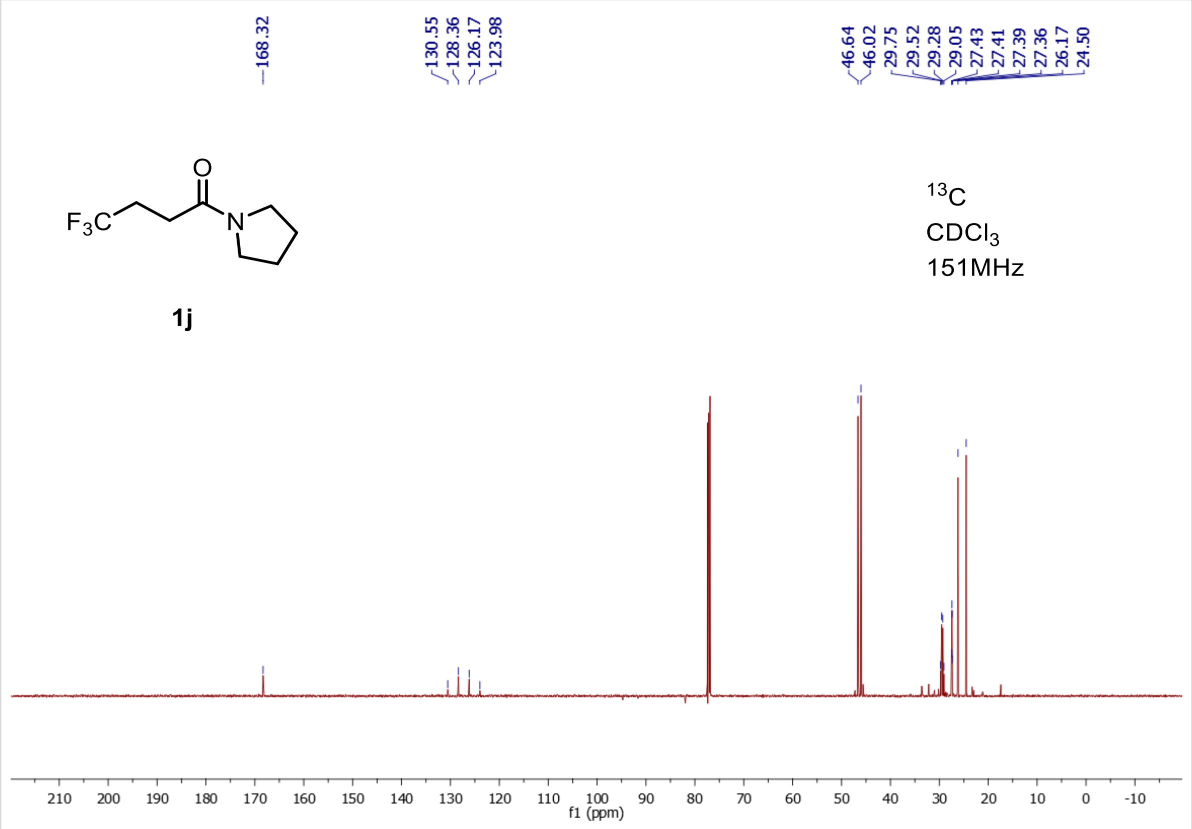
168.32
130.55 128.36 126.17 123.98
46.64 46.02 29.75 29.52 29.28 29.05 27.43 27.41 27.39 27.36 26.17 24.50
F3C
O
N
1j
13C
CDCl3
151MHz
210 200 190 180 170 160 150 140 130 120 110 100 90 80 70 60 50 40 30 20 10 0 -10
f1 (ppm)

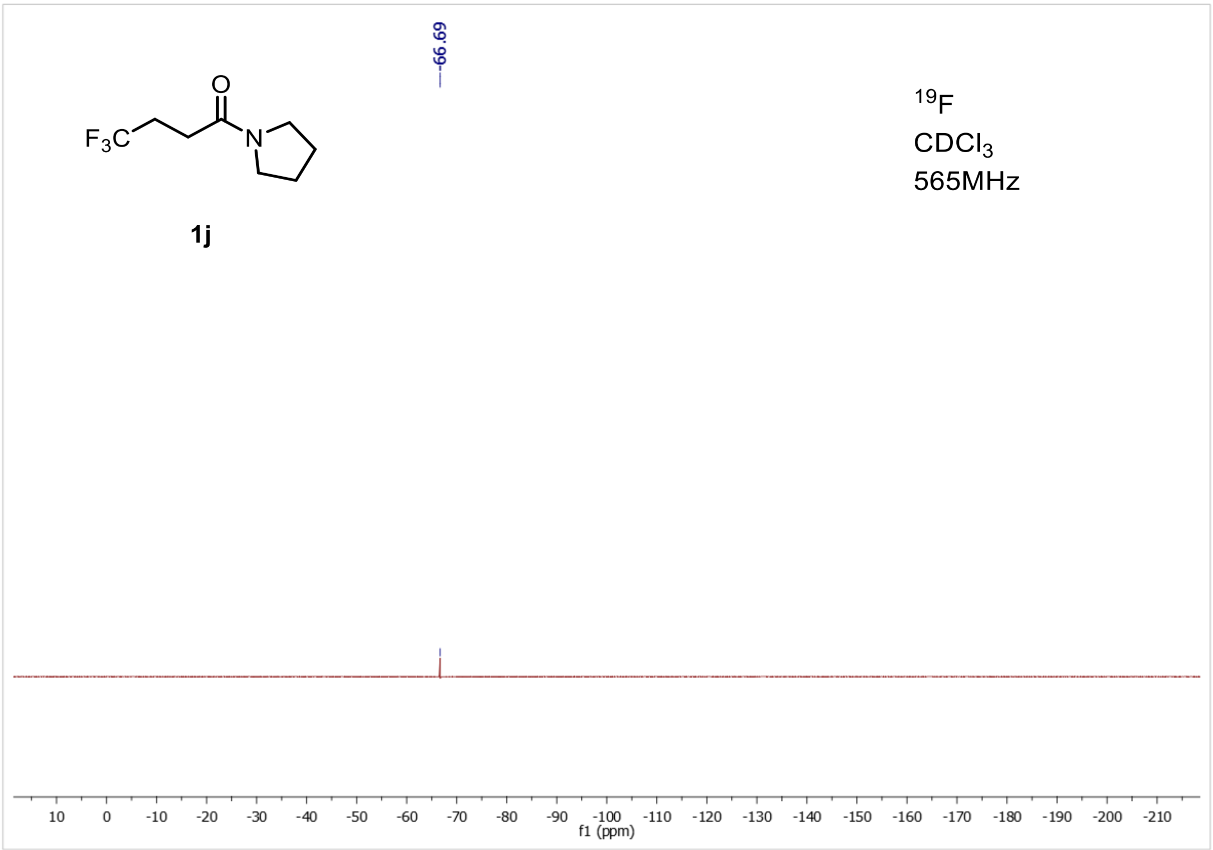

1j
−66.69
^{19}F
CDCl$_3$
565MHz
10 0 -10 -20 -30 -40 -50 -60 -70 -80 -90 -100 -110 -120 -130 -140 -150 -160 -170 -180 -190 -200 -210
f1 (ppm)

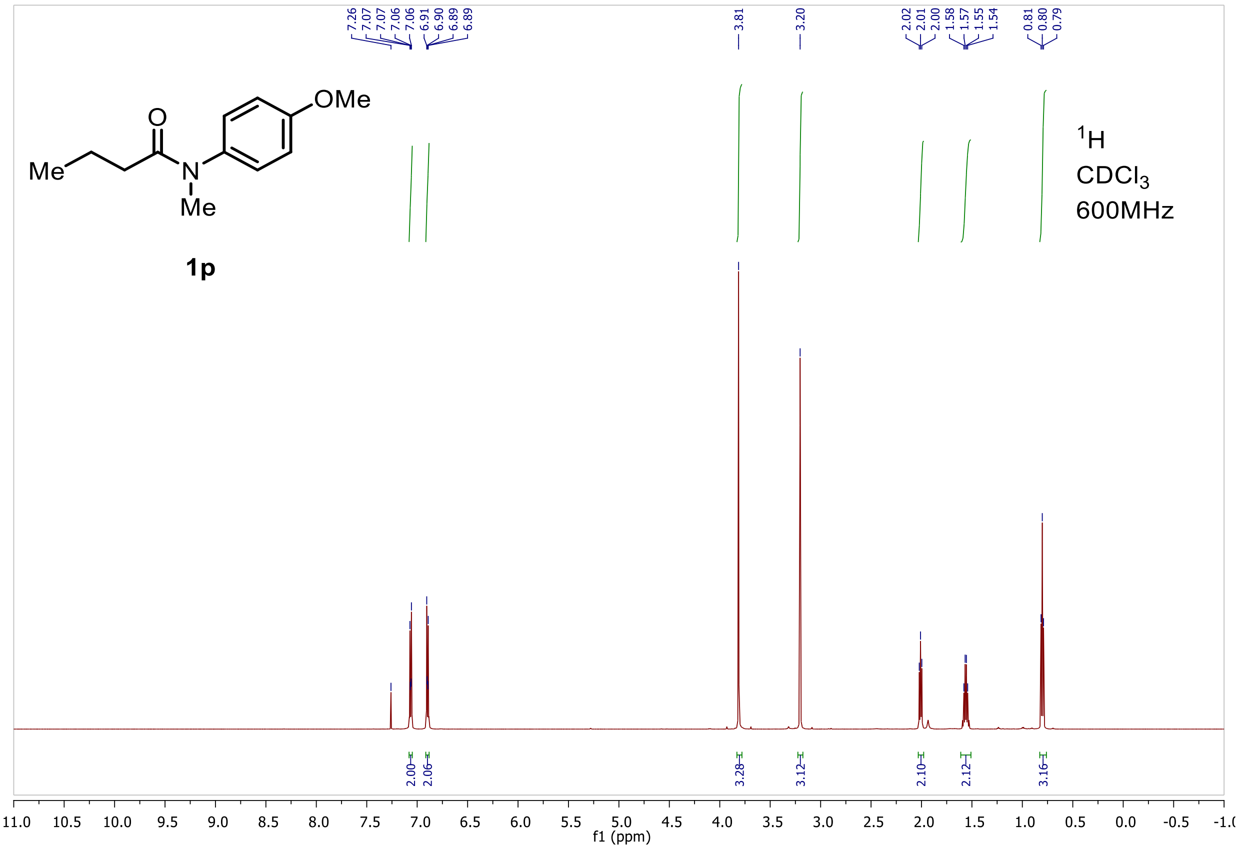

OMe
O
Me
N
Me
1p
^{1}H
CDCl$_3$
600MHz
f1 (ppm)

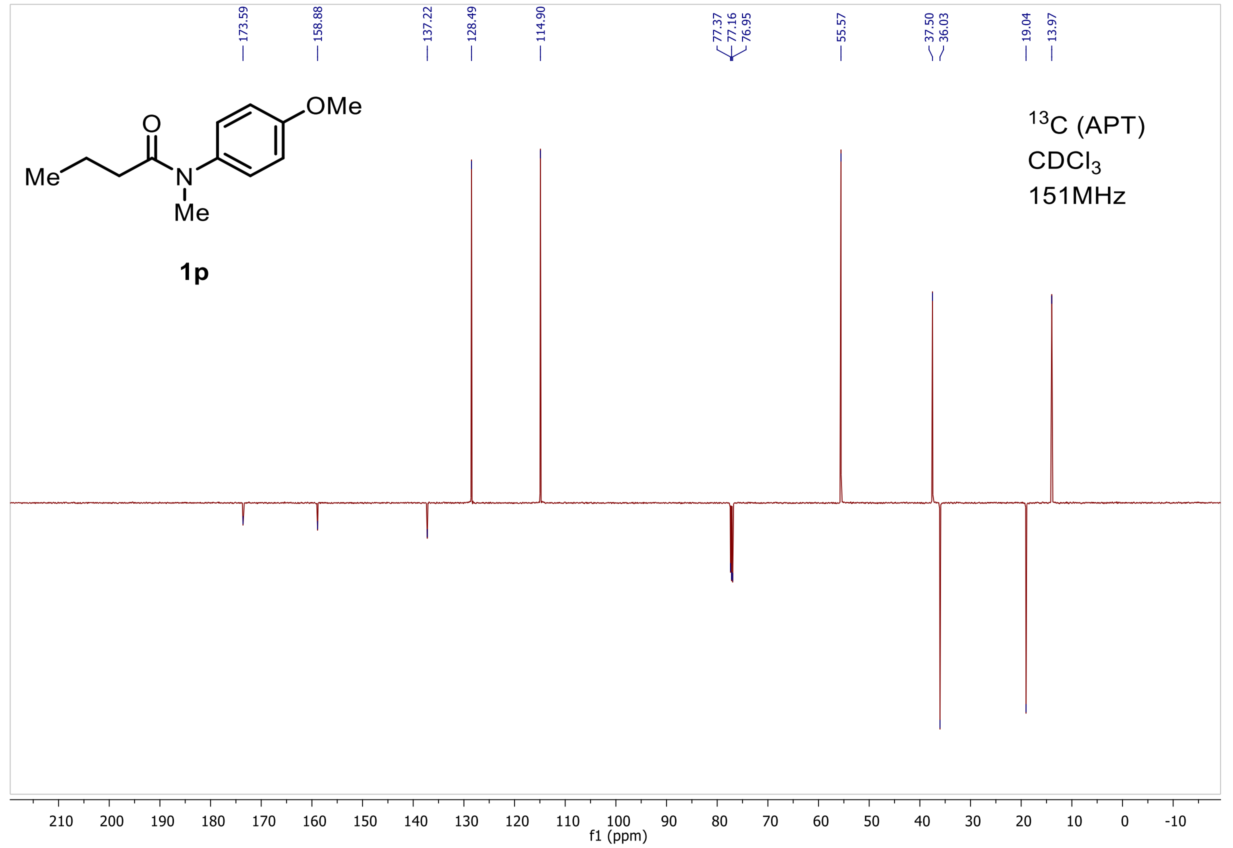

OMe
O
Me
N
Me
1p
^{13}C (APT)
CDCl$_3$
151MHz
f1 (ppm)

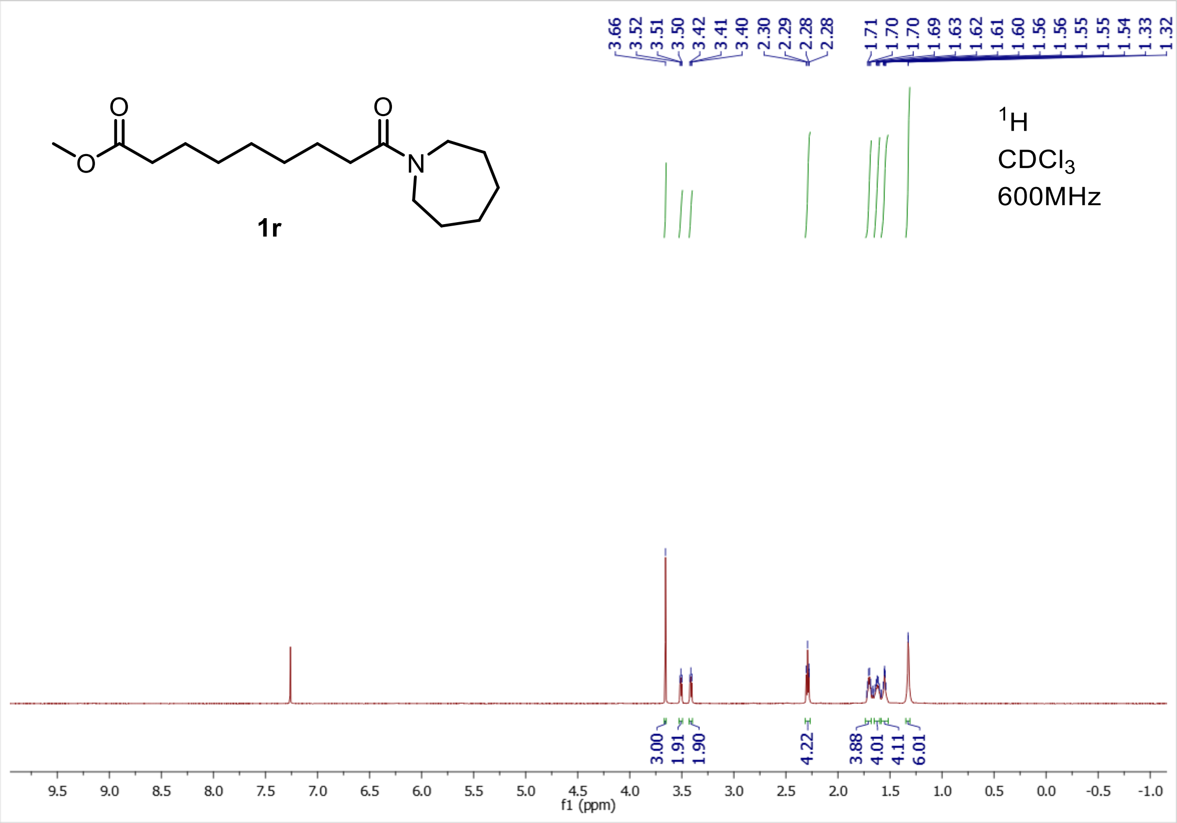
3.66
3.52
3.51
3.50
3.42
3.41
3.40
2.30
2.29
2.28
2.28
1.71
1.70
1.70
1.69
1.63
1.62
1.61
1.60
1.56
1.56
1.55
1.55
1.54
1.33
1.32
1r
^{1}H
$CDCl_3$
600MHz
3.00
1.91
1.90
4.22
3.88
4.01
4.11
6.01
f1 (ppm)

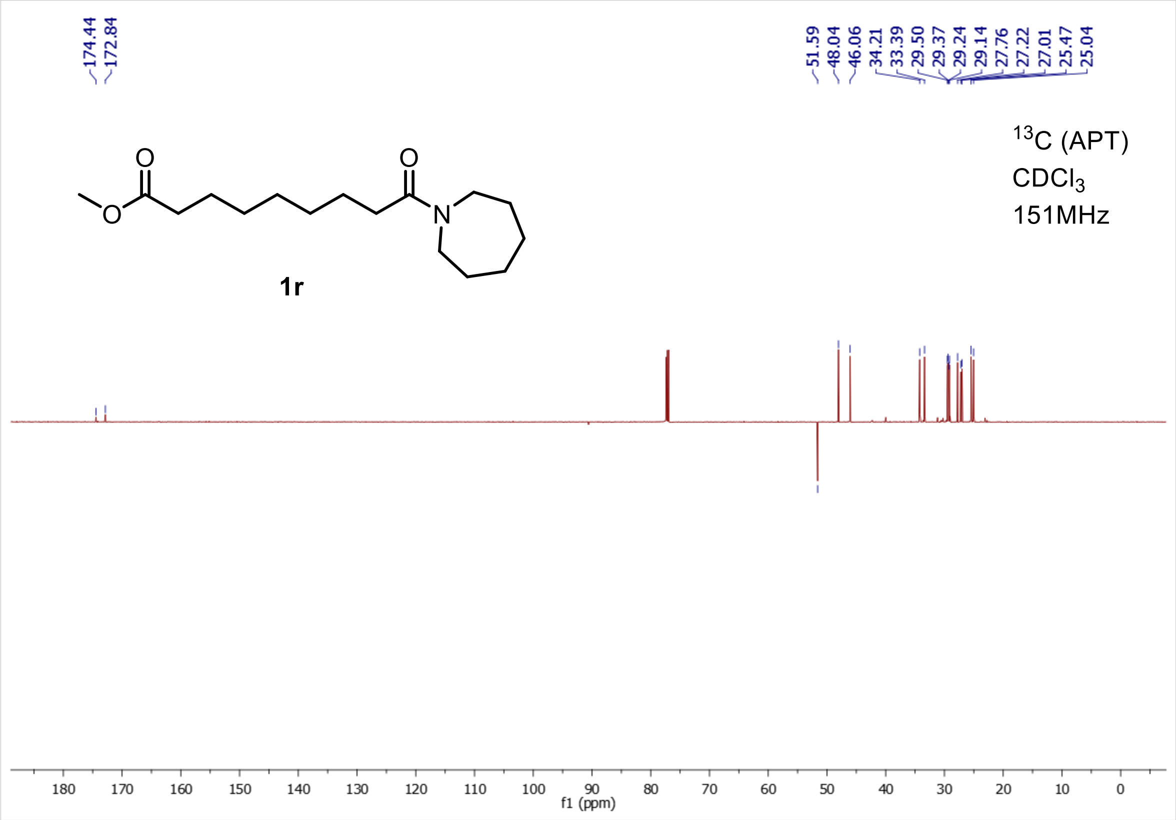
174.44
172.84
51.59
48.04
46.06
34.21
33.39
29.50
29.37
29.24
29.14
27.76
27.22
27.01
25.47
25.04
1r
^{13}C (APT)
$CDCl_3$
151MHz
f1 (ppm)

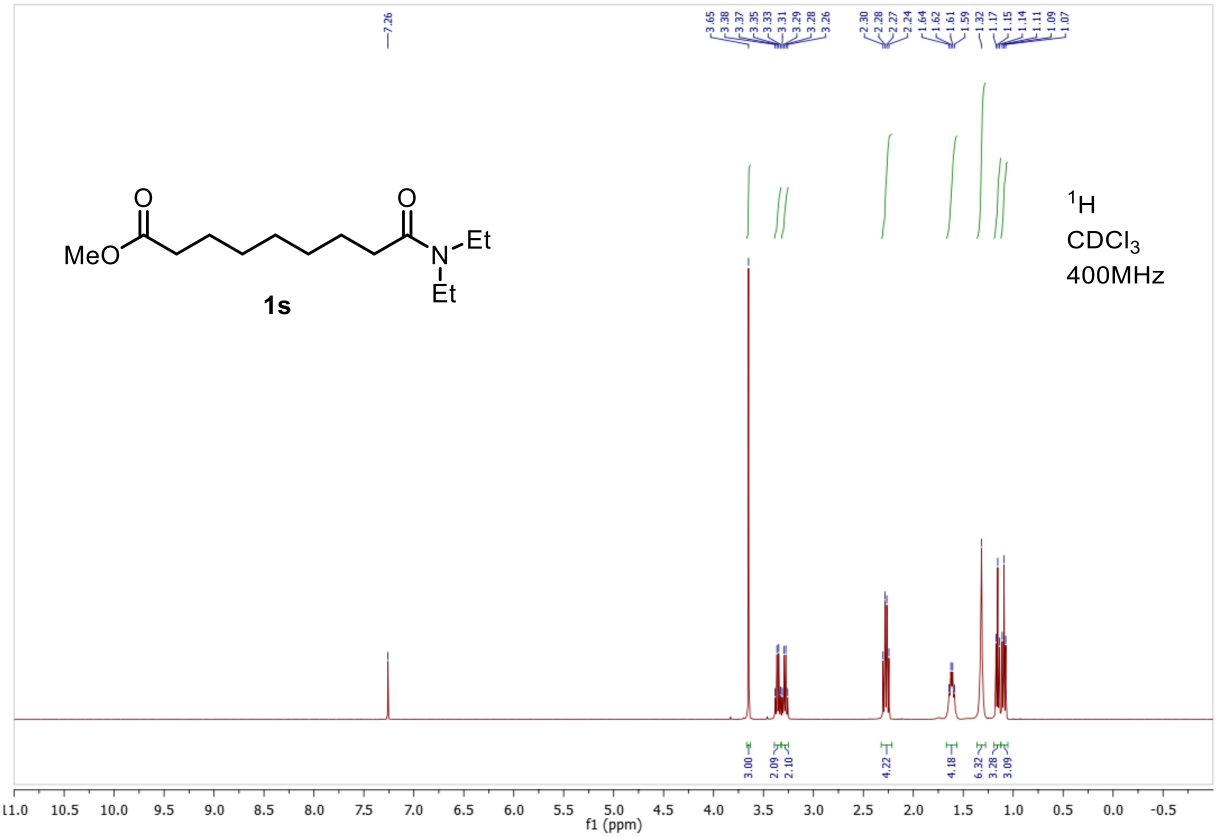
1s
MeO
Et
N
1H
CDCl3
400MHz
f1 (ppm)

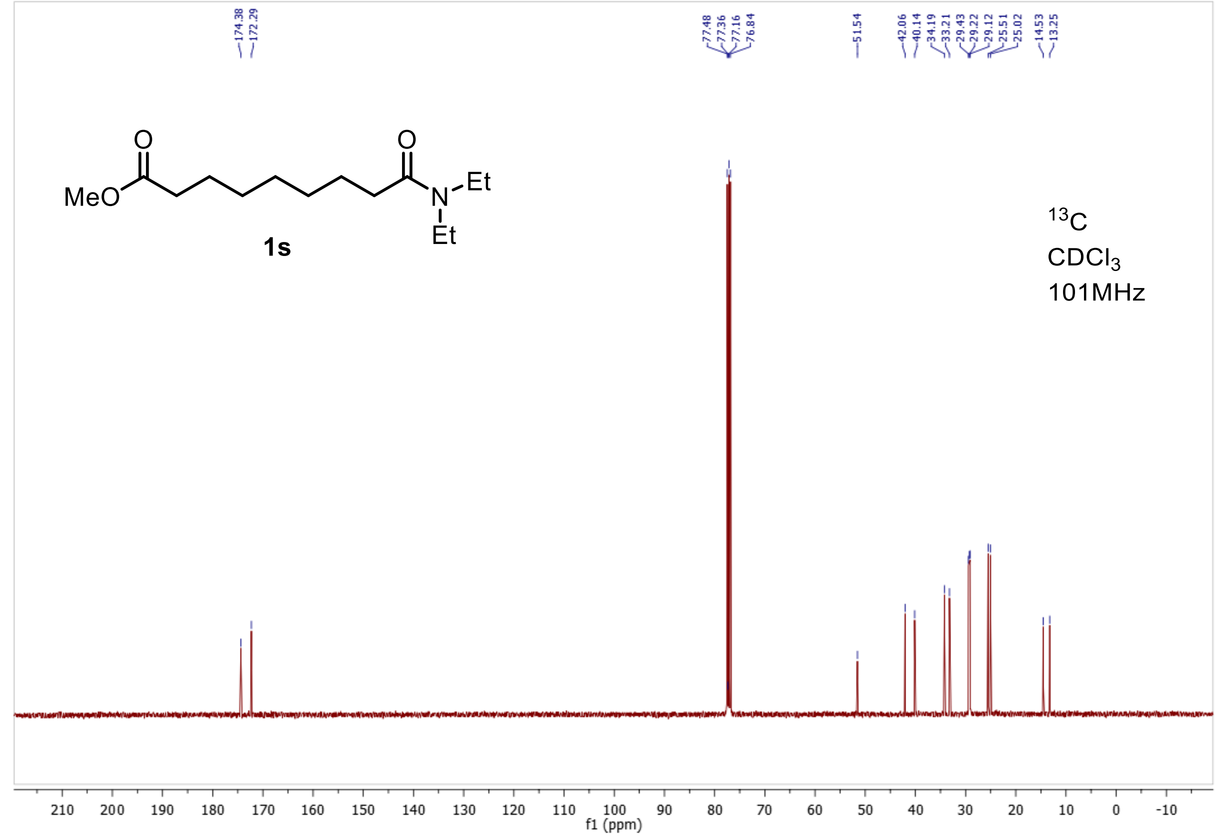
1s
MeO
Et
N
13C
CDCl3
101MHz
f1 (ppm)

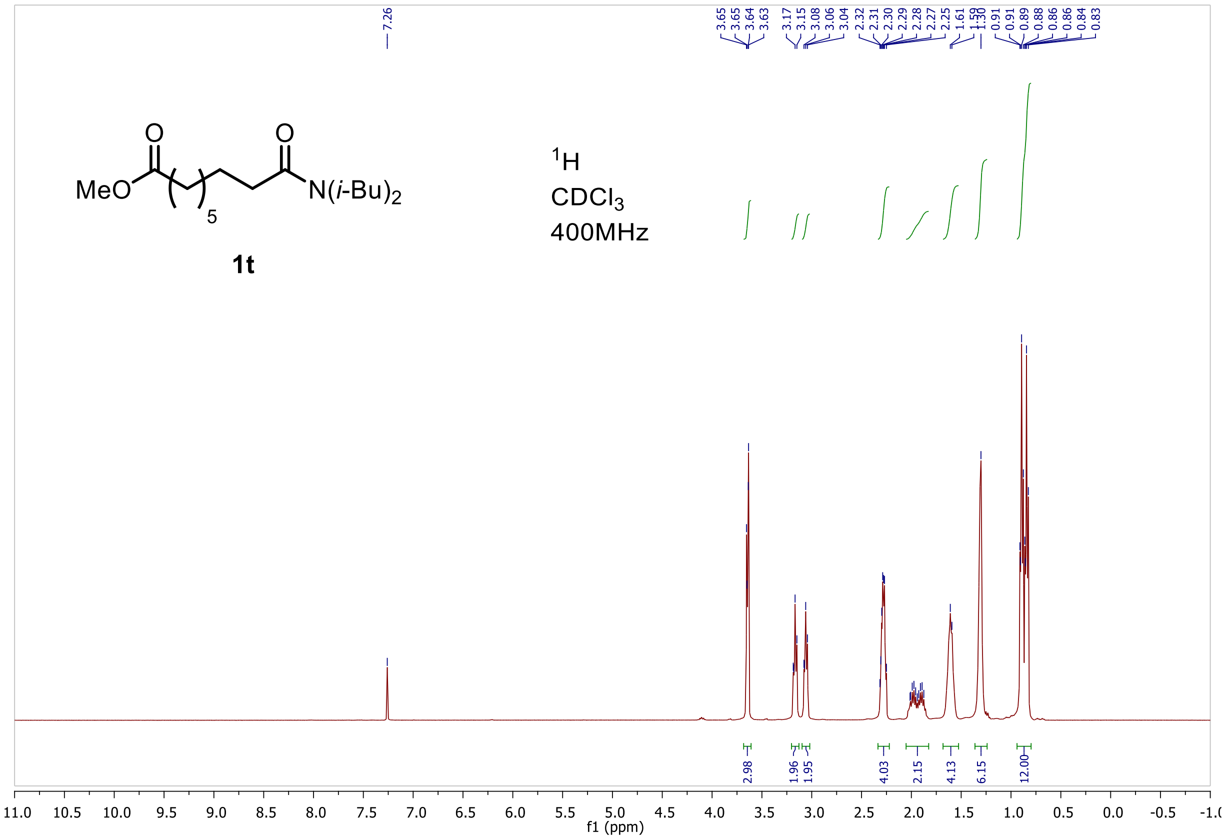

MeO
N(i-Bu)2
5
1t
1H
CDCl3
400MHz
f1 (ppm)

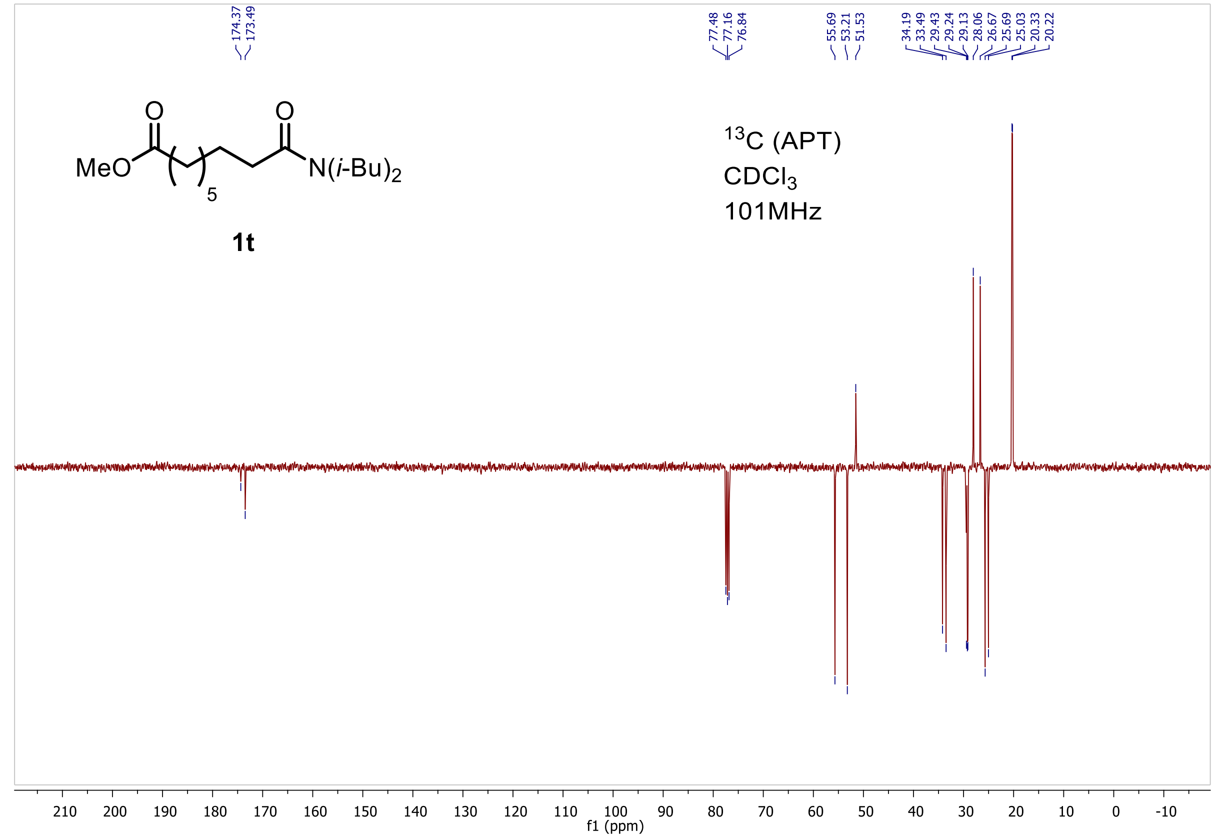

MeO
N(i-Bu)2
5
1t
13C (APT)
CDCl3
101MHz
f1 (ppm)

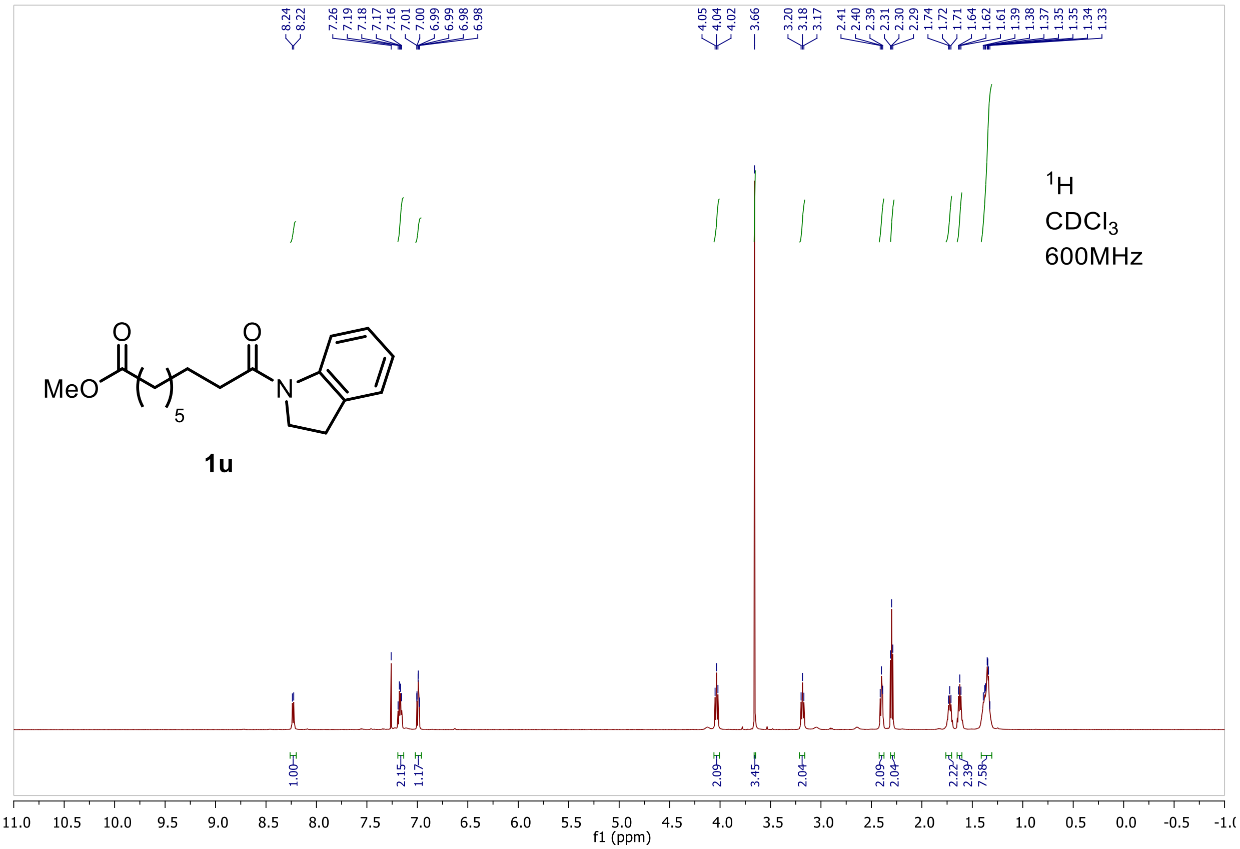
8.24
8.22
7.26
7.19
7.18
7.17
7.16
7.01
7.00
6.99
6.99
6.98
6.98
4.05
4.04
4.02
3.66
3.20
3.18
3.17
2.41
2.40
2.39
2.31
2.30
2.29
1.74
1.72
1.71
1.64
1.62
1.61
1.39
1.38
1.37
1.35
1.35
1.34
1.33
^{1}H
$CDCl_3$
600MHz
MeO
O
O
N
5
1u
1.00
2.15
1.17
2.09
3.45
2.04
2.09
2.04
2.22
2.39
7.58
f1 (ppm)

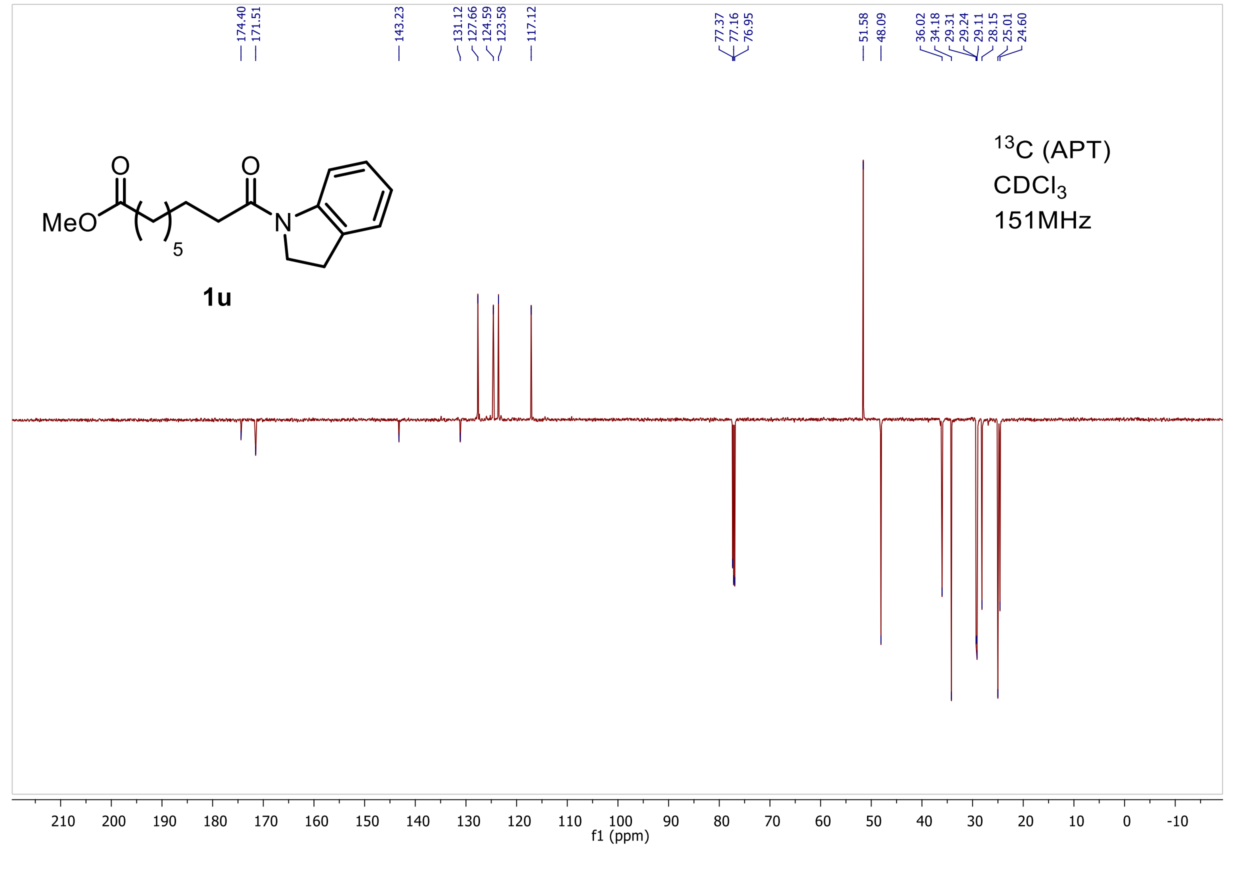
174.40
171.51
143.23
131.12
127.66
124.59
123.58
117.12
77.37
77.16
76.95
51.58
48.09
36.02
34.18
29.31
29.24
29.11
28.15
25.01
24.60
^{13}C (APT)
$CDCl_3$
151MHz
MeO
O
O
N
5
1u
f1 (ppm)

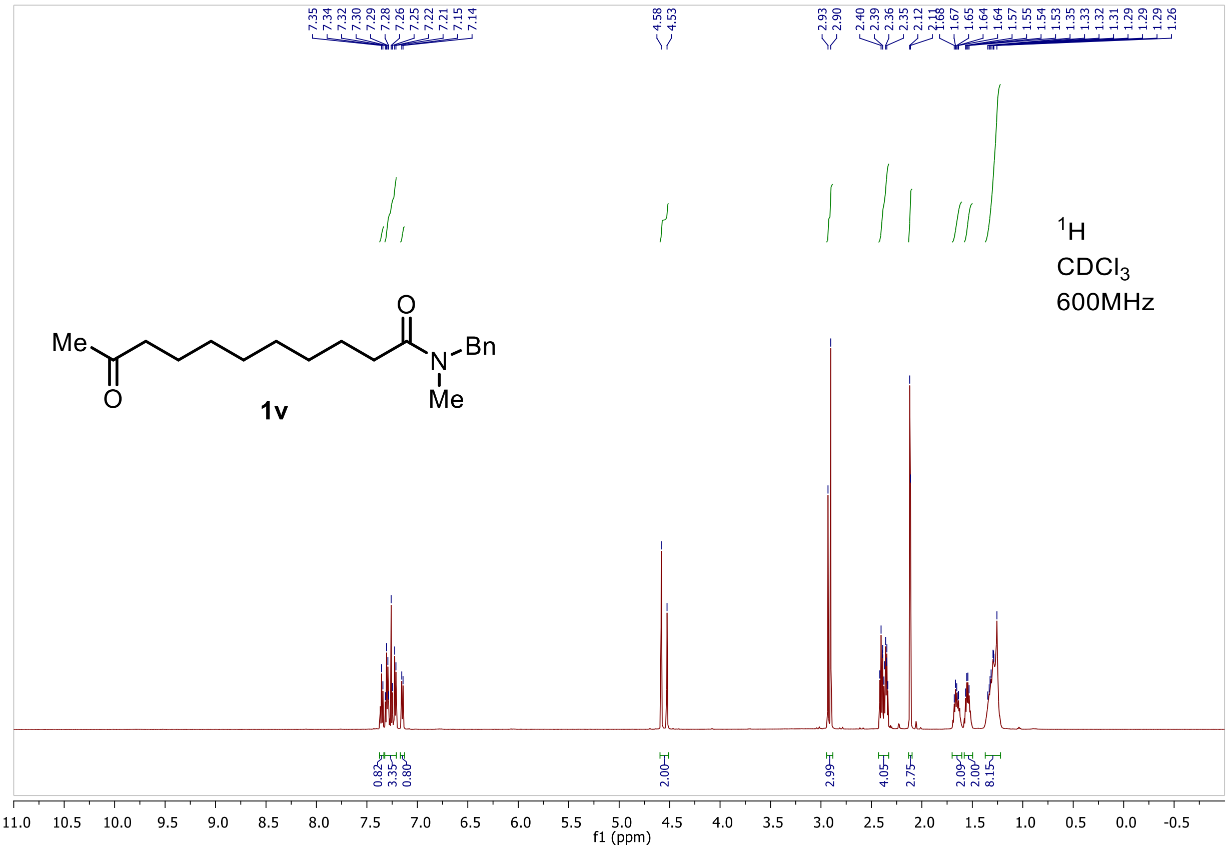
Me
O
N
Bn
Me
1v
^{1}H
CDCl$_3$
600MHz
f1 (ppm)

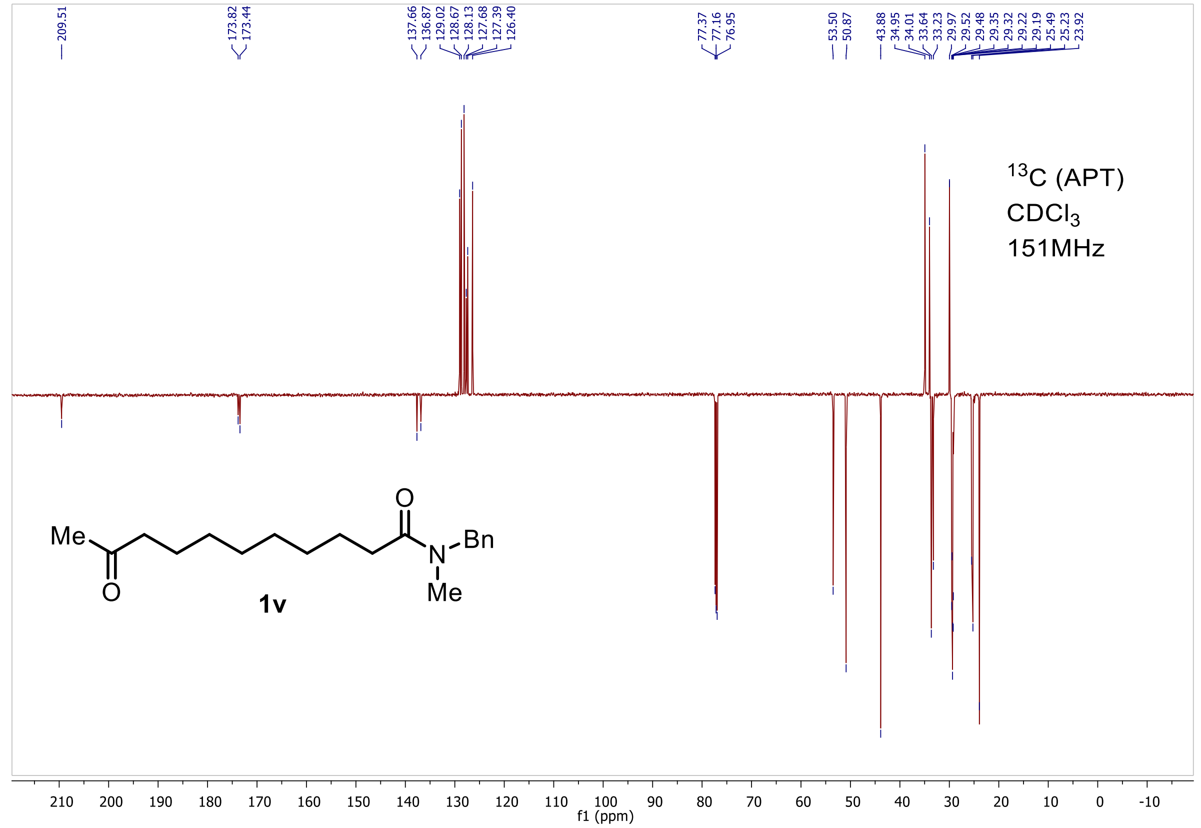
Me
O
N
Bn
Me
1v
^{13}C (APT)
CDCl$_3$
151MHz
f1 (ppm)

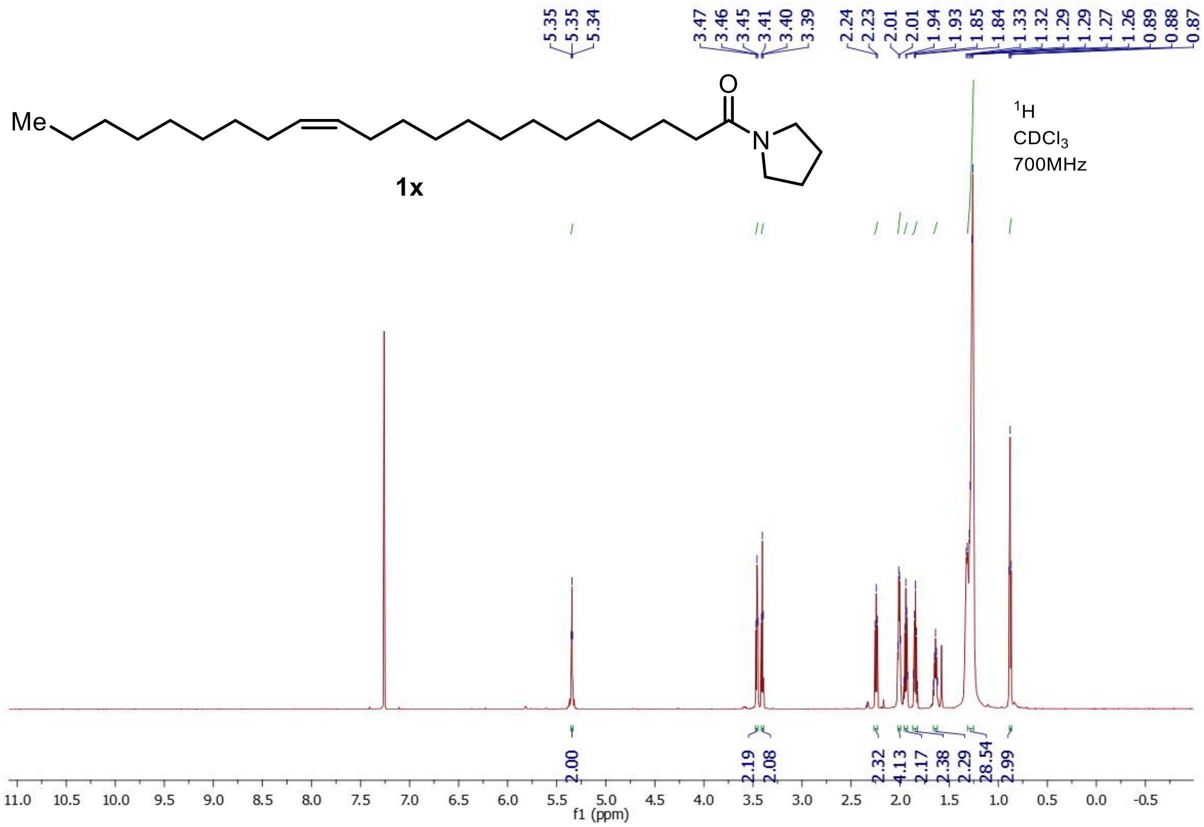
5.35
5.35
5.34
3.47
3.46
3.45
3.41
3.40
3.39
2.24
2.23
2.01
2.01
1.94
1.93
1.85
1.84
1.33
1.32
1.29
1.29
1.27
1.26
0.89
0.88
0.87
Me
O
N
1x
^{1}H
$CDCl_3$
700MHz
2.00
2.19
2.08
2.32
4.13
2.17
2.38
2.29
28.54
2.99
f1 (ppm)

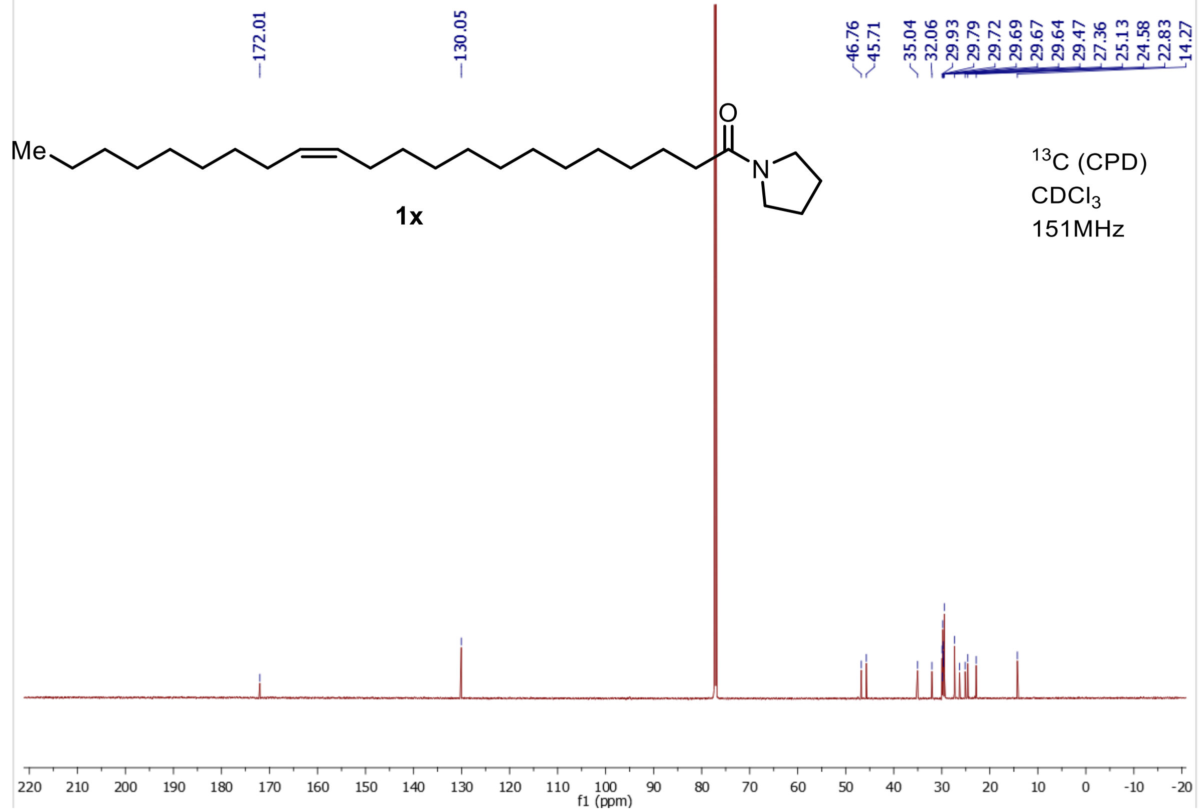
172.01
130.05
46.76
45.71
35.04
32.06
29.93
29.79
29.72
29.69
29.67
29.64
29.47
27.36
25.13
24.58
22.83
14.27
Me
O
N
1x
^{13}C (CPD)
$CDCl_3$
151MHz
f1 (ppm)

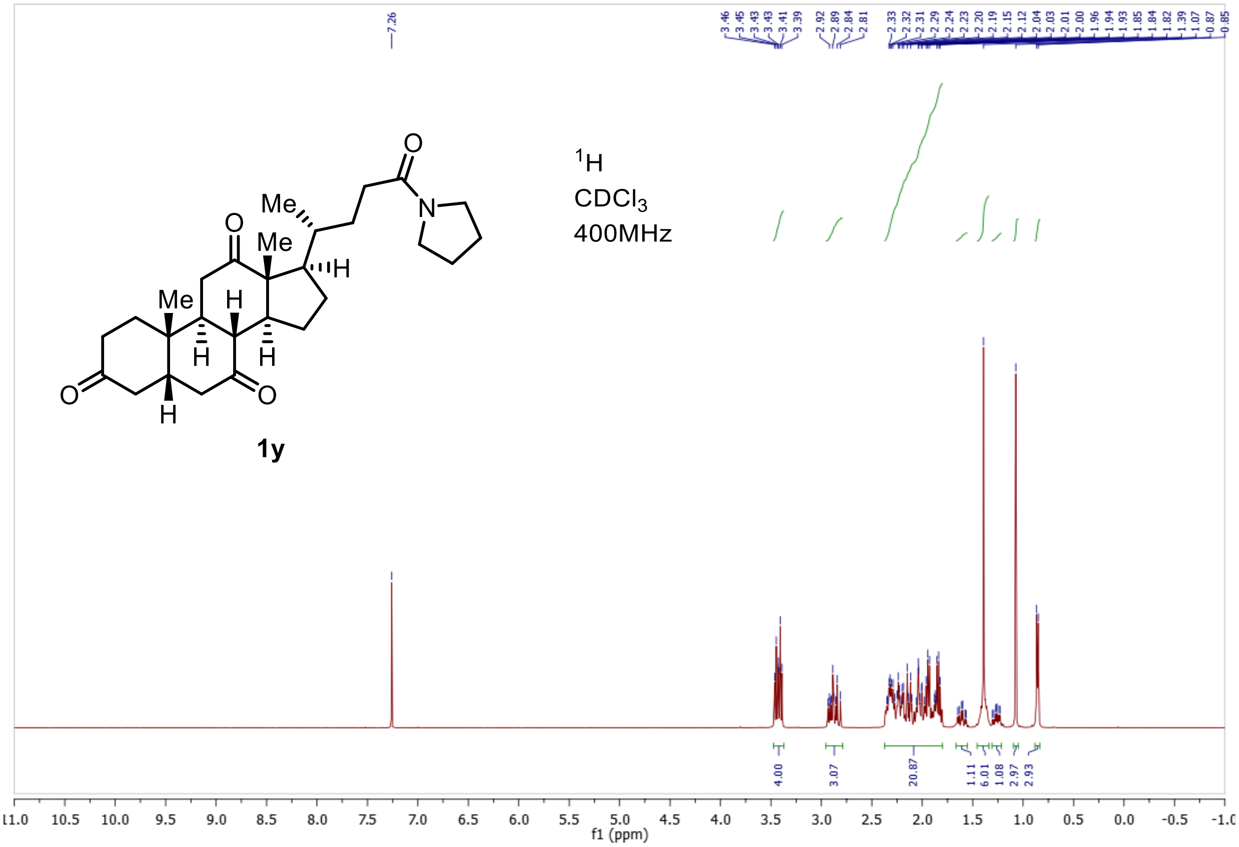

1H
CDCl3
400MHz
1y
Me
O
N
H
f1 (ppm)

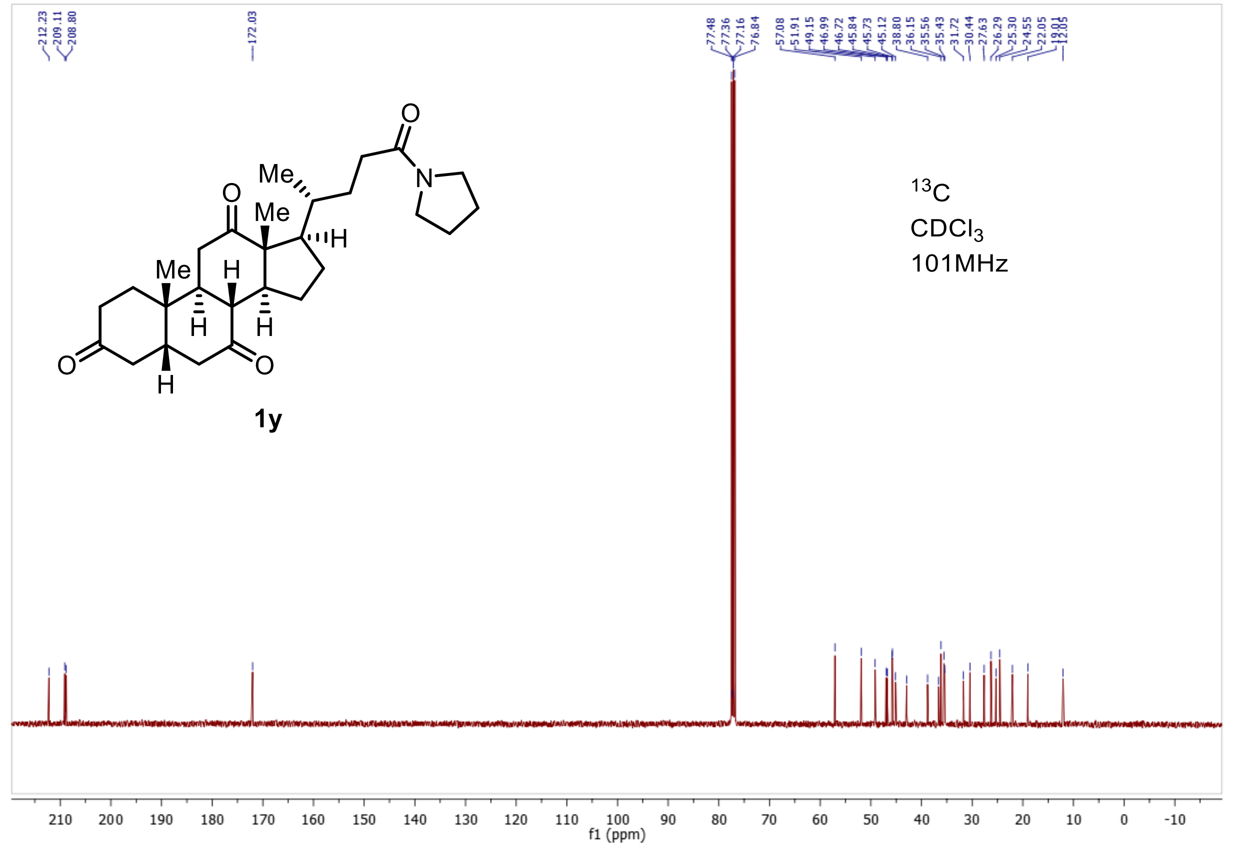

13C
CDCl3
101MHz
1y
Me
O
N
H
f1 (ppm)

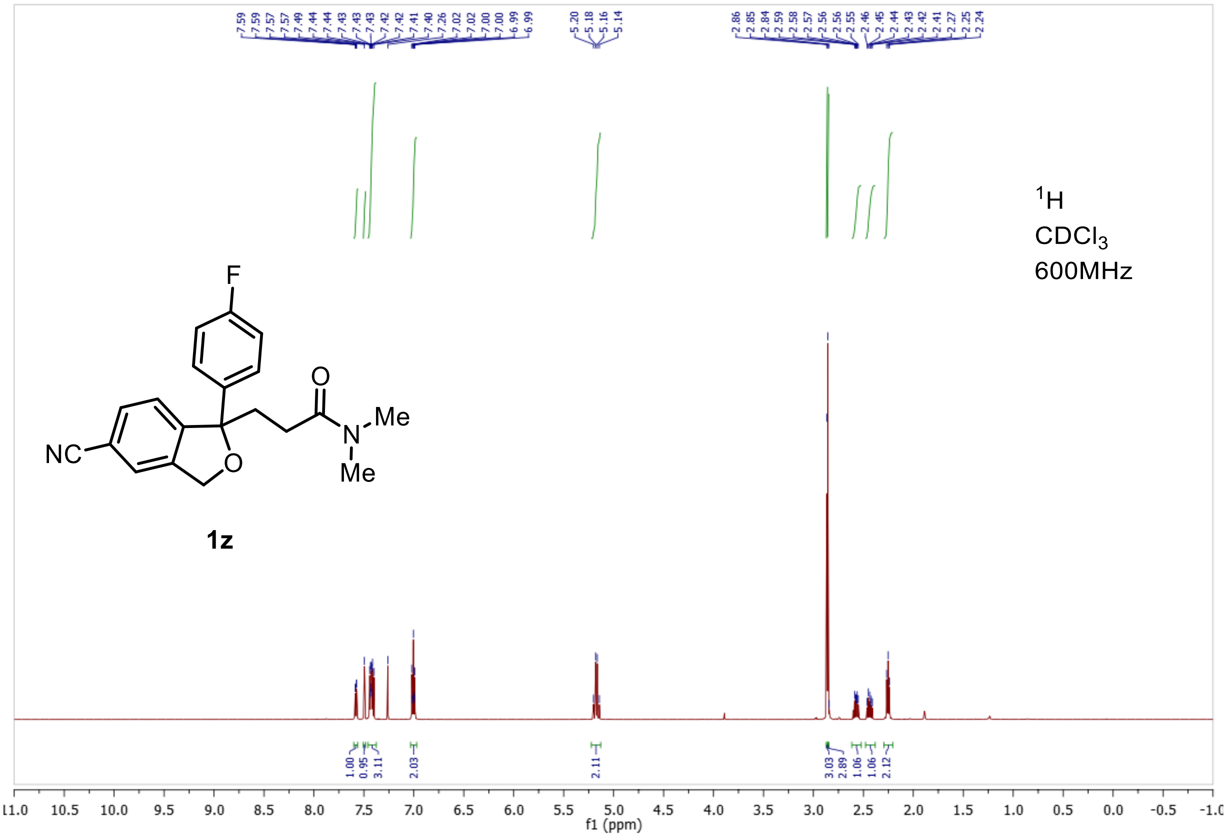

1z

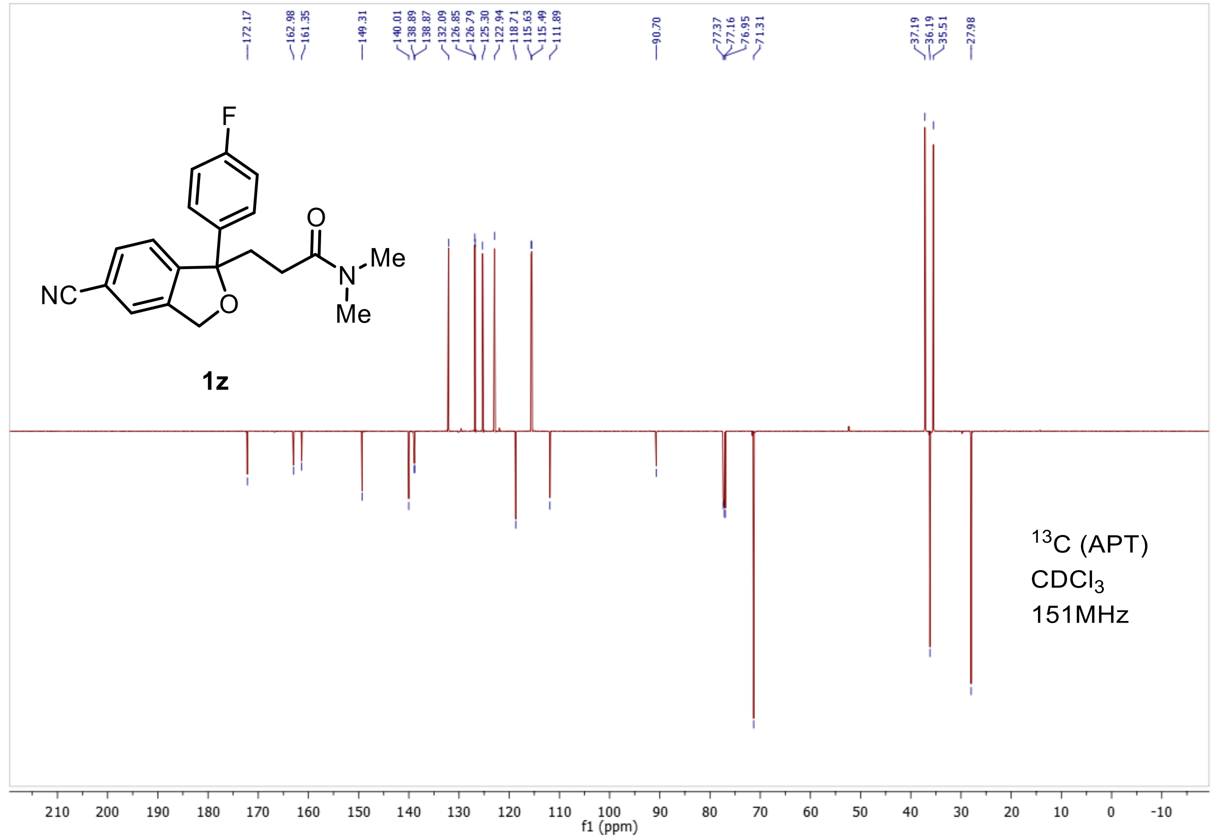

1z

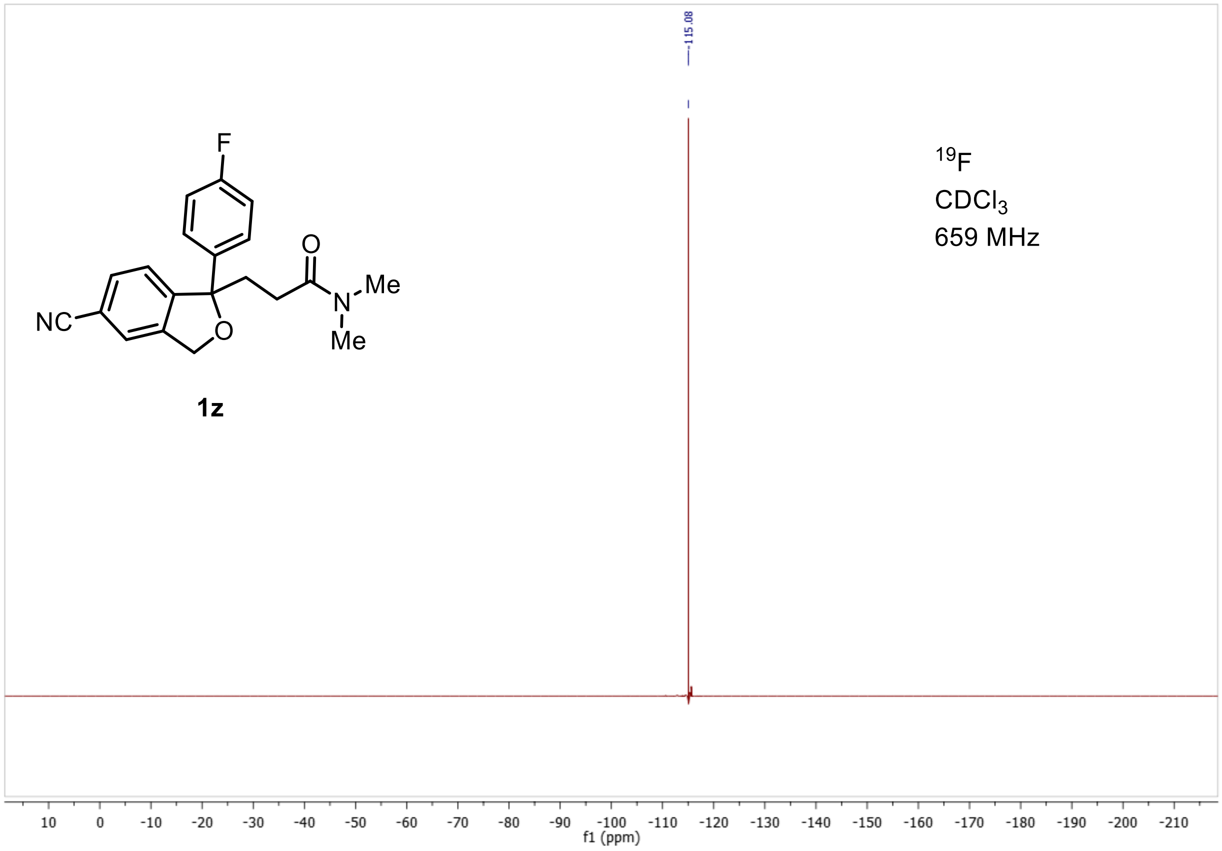
F
O
NC
O
N
Me
Me
1z
-115.08
^{19}F
CDCl$_3$
659 MHz
10 0 -10 -20 -30 -40 -50 -60 -70 -80 -90 -100 -110 -120 -130 -140 -150 -160 -170 -180 -190 -200 -210
f1 (ppm)

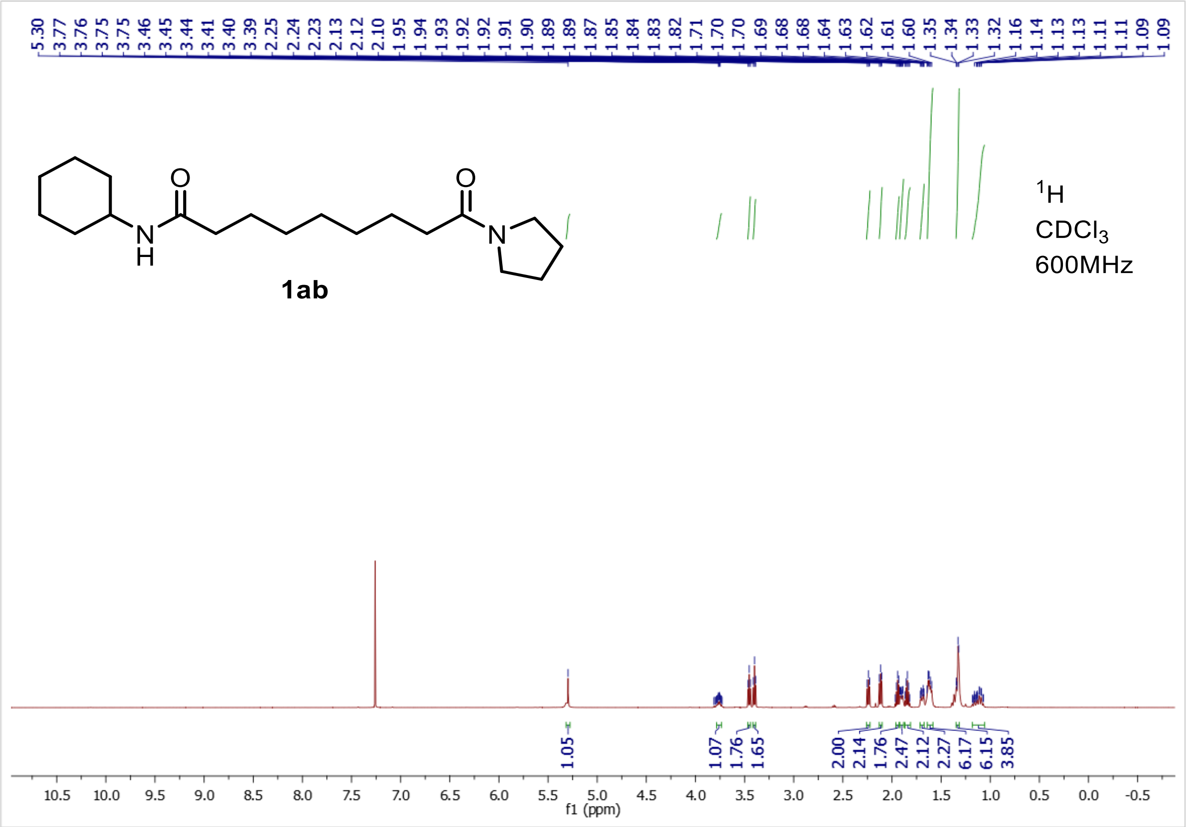

1ab
^{1}H
$CDCl_3$
600MHz
f1 (ppm)

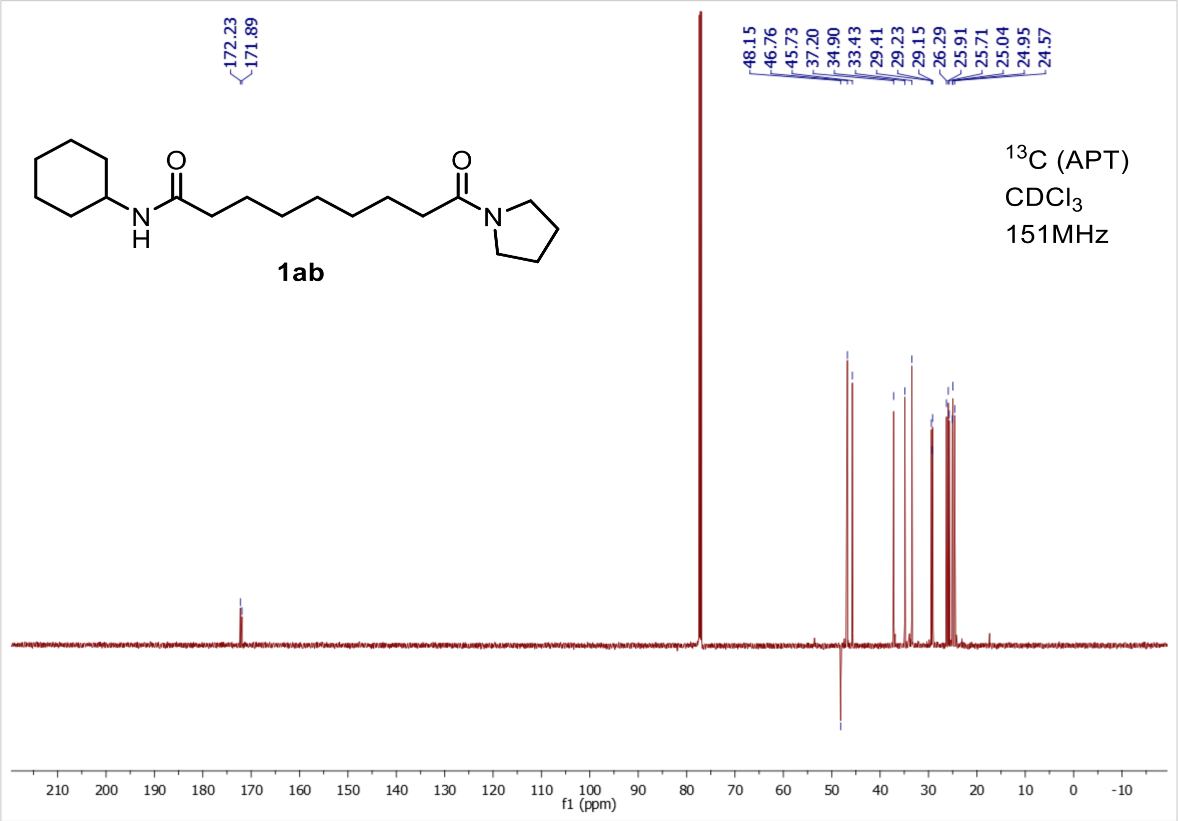

172.23
171.89
48.15
46.76
45.73
37.20
34.90
33.43
29.41
29.23
29.15
26.29
25.91
25.71
25.04
24.95
24.57
1ab
^{13}C (APT)
$CDCl_3$
151MHz
f1 (ppm)

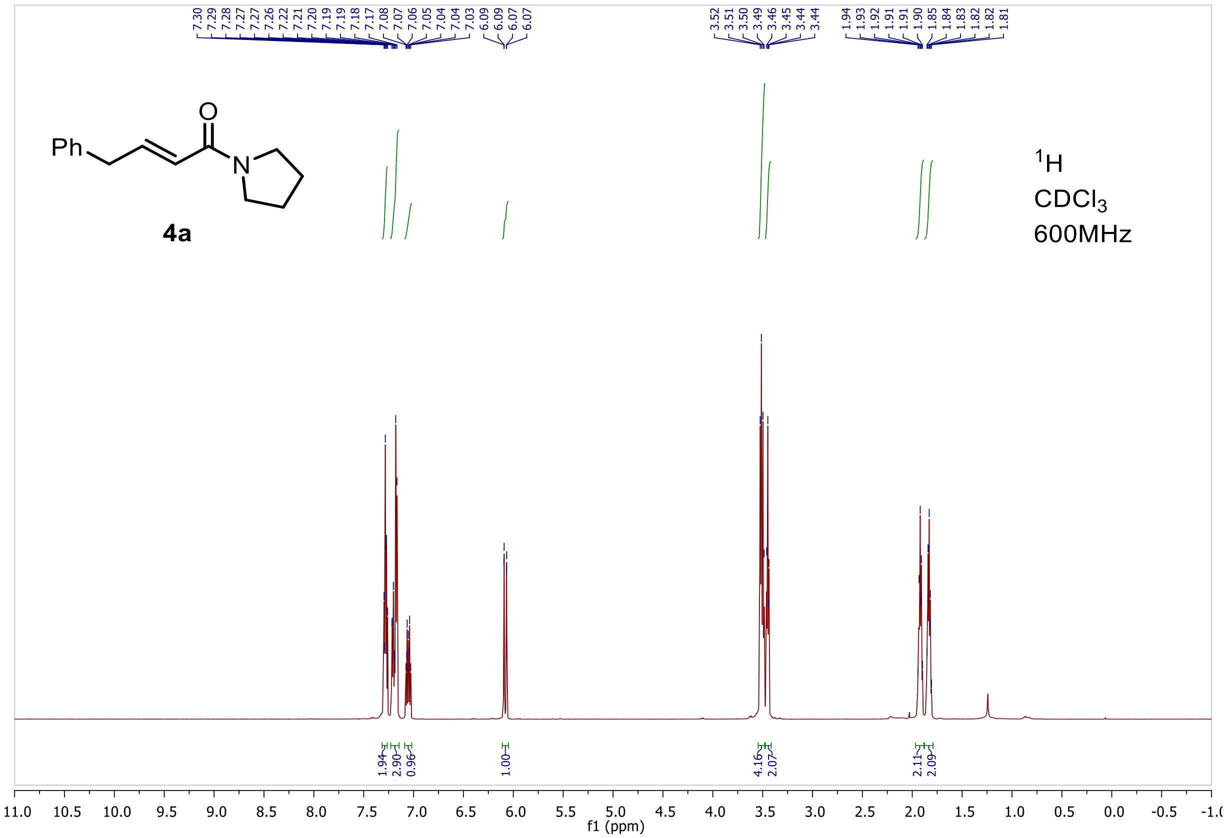
7.30 7.29 7.28 7.27 7.27 7.26 7.22 7.21 7.20 7.19 7.19 7.18 7.17 7.08 7.07 7.06 7.05 7.04 7.04 7.03 6.09 6.09 6.07 6.07
3.52 3.51 3.50 3.49 3.46 3.45 3.44 3.44
1.94 1.93 1.92 1.91 1.91 1.90 1.85 1.84 1.83 1.82 1.82 1.81
Ph
O
N
4a
1H
CDCl3
600MHz
1.94 2.90 0.96
1.00
4.16 2.07
2.11 2.09
f1 (ppm)

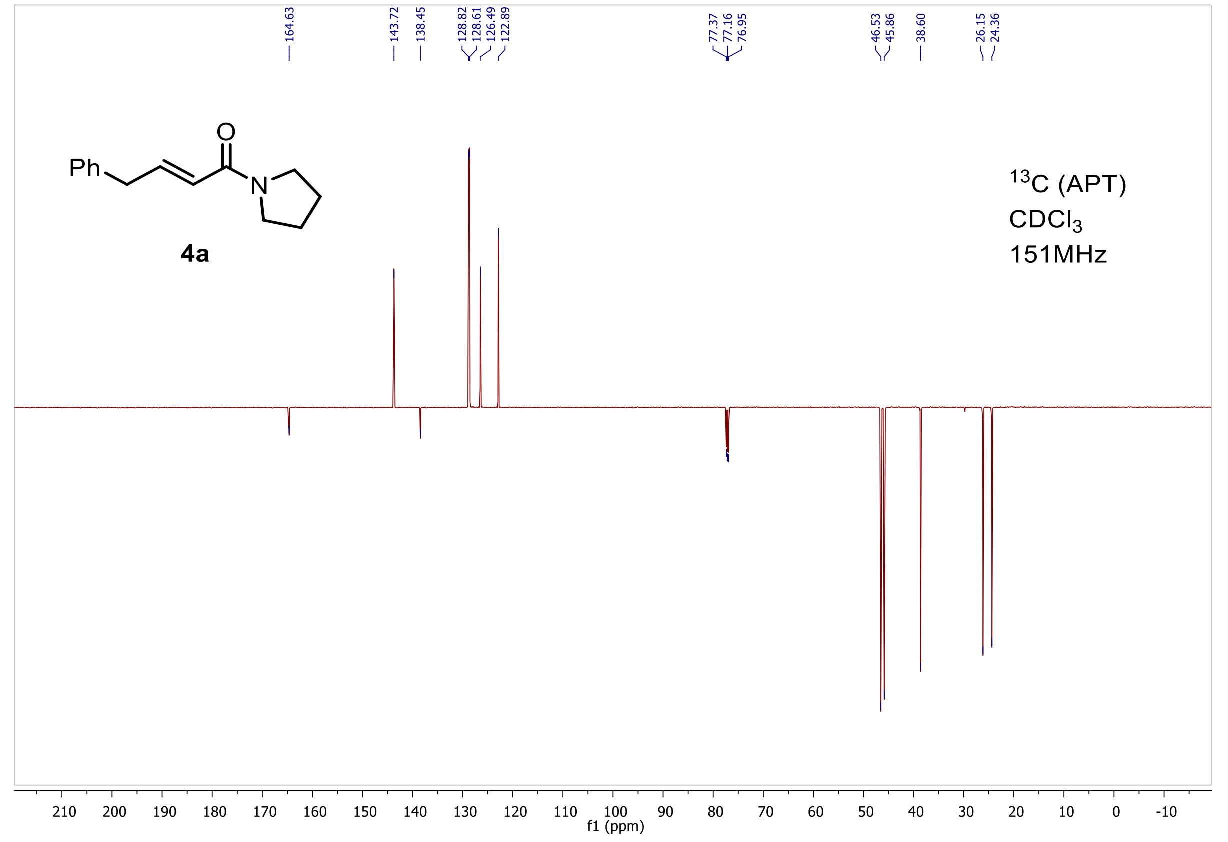
164.63
143.72
138.45
128.82 128.61 126.49 122.89
77.37 77.16 76.95
46.53 45.86
38.60
26.15 24.36
Ph
O
N
4a
13C (APT)
CDCl3
151MHz
f1 (ppm)

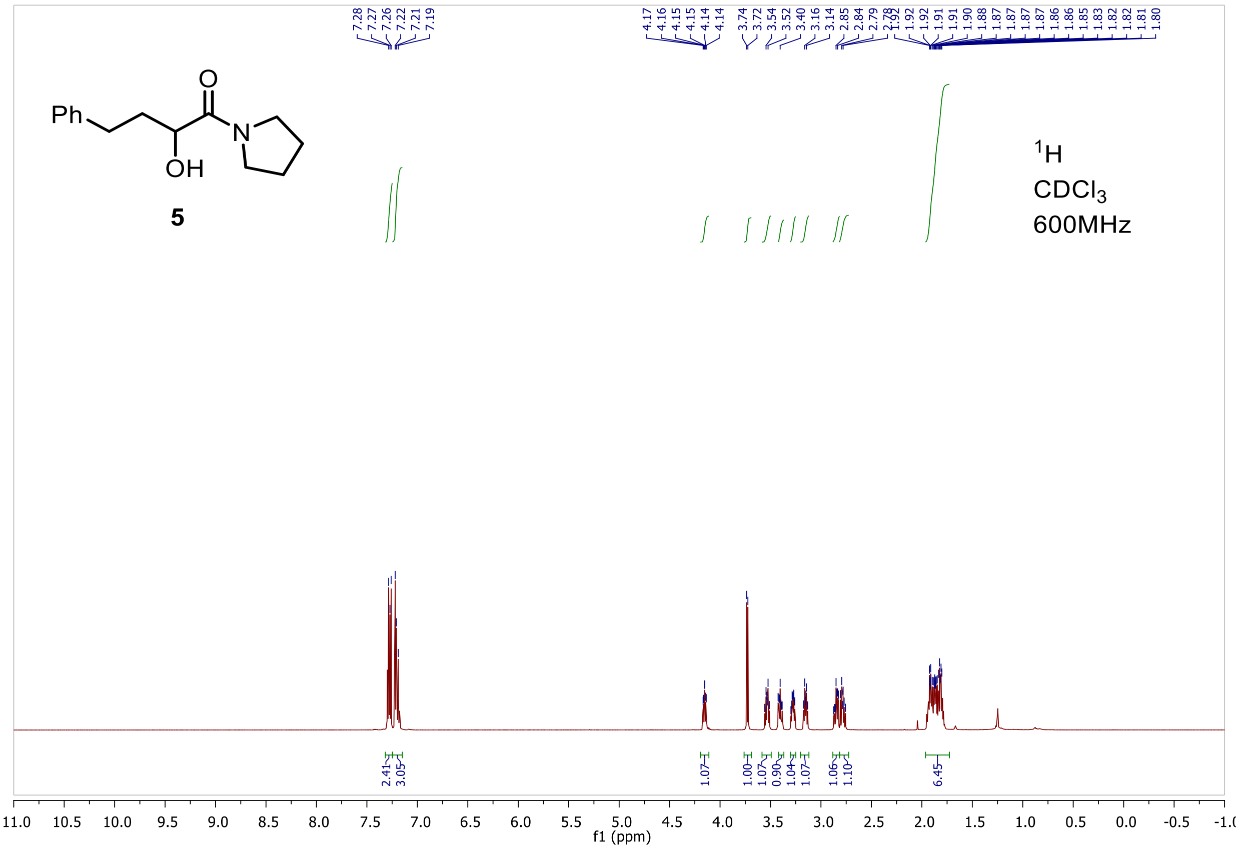
Ph
O
N
OH
5
¹H
CDCl3
600MHz
f1 (ppm)

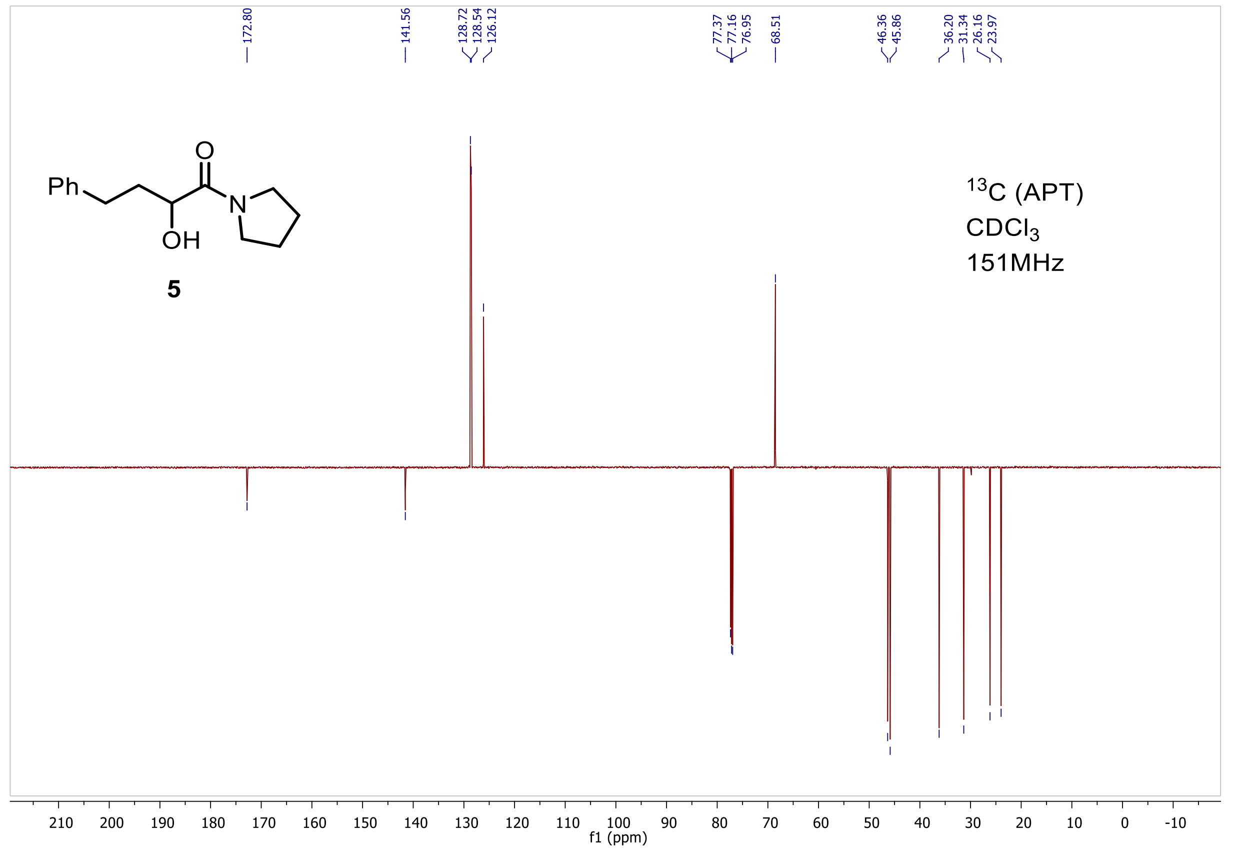
Ph
O
N
OH
5
13C (APT)
CDCl3
151MHz
f1 (ppm)

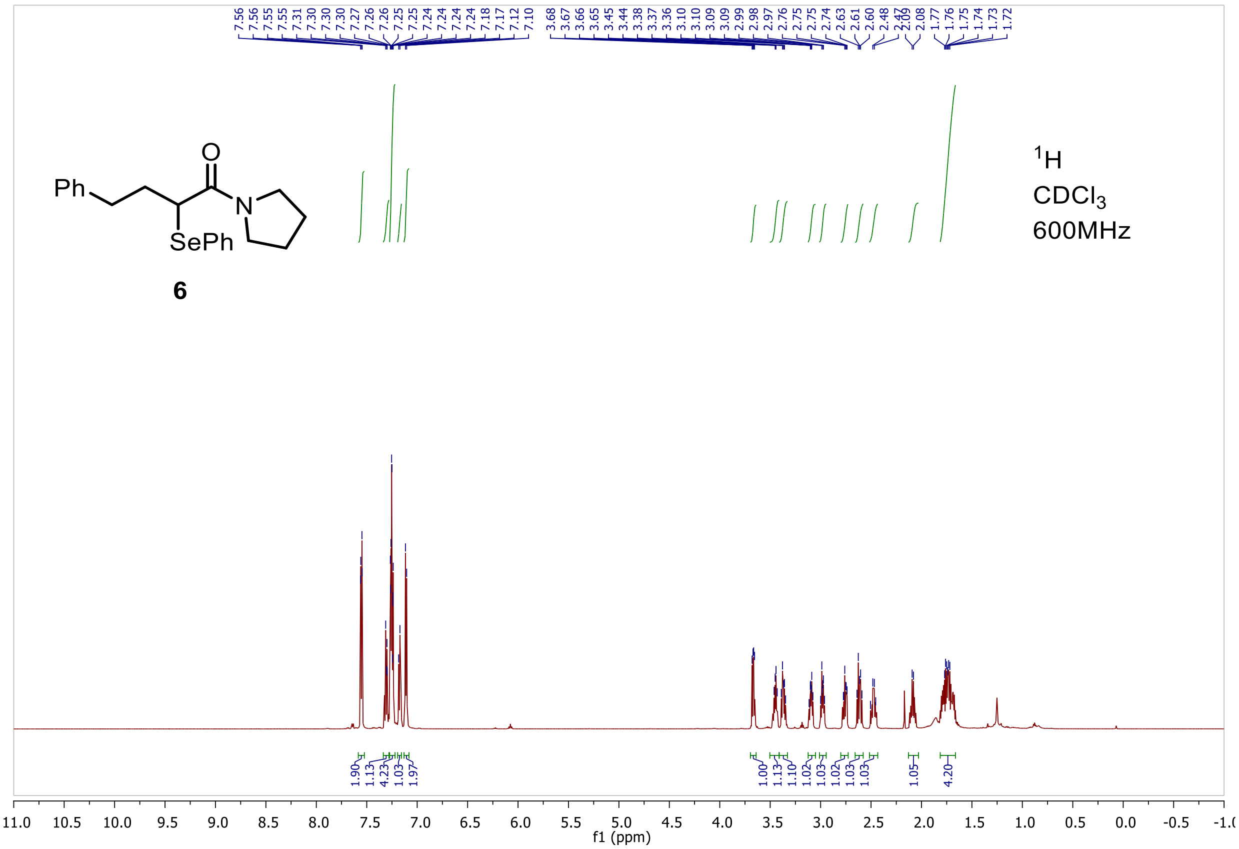

Ph
O
N
SePh
6
¹H
CDCl3
600MHz
f1 (ppm)

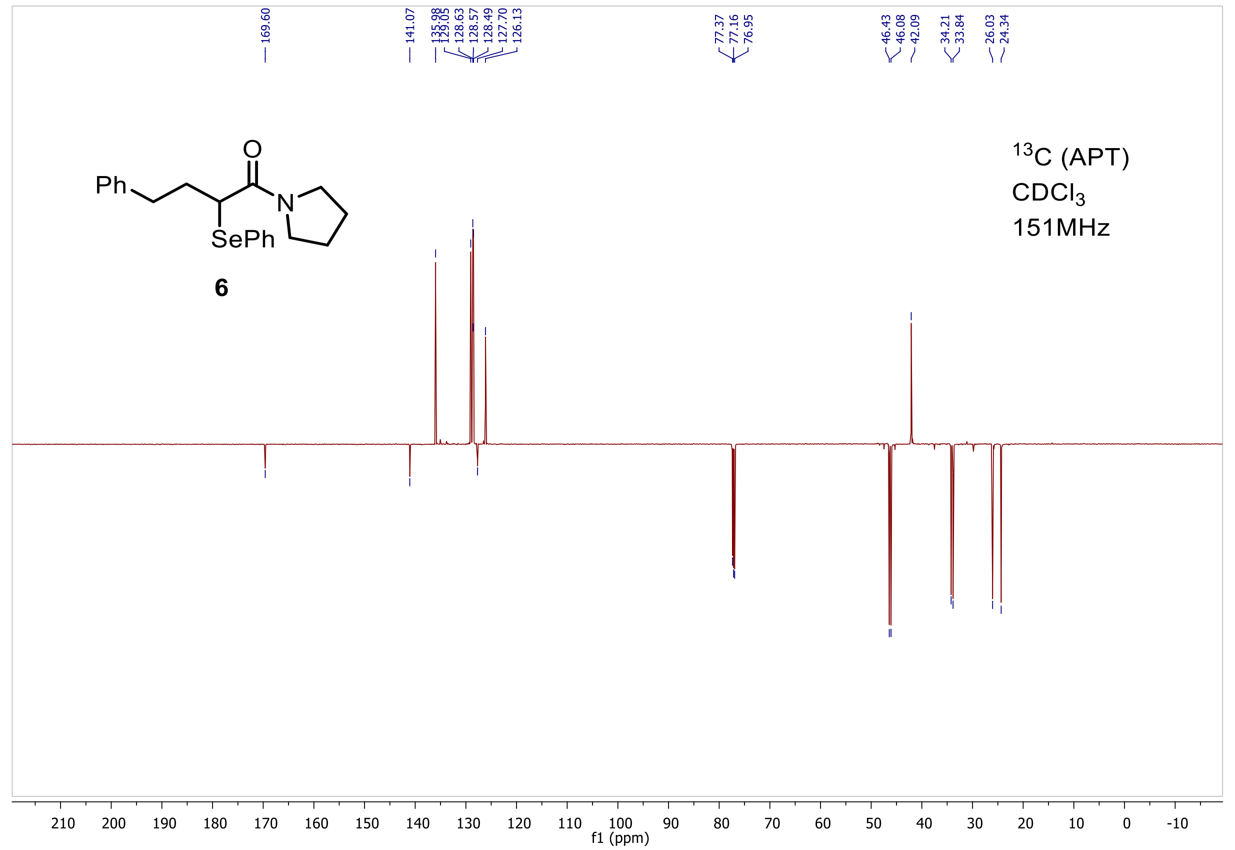

Ph
O
N
SePh
6
13C (APT)
CDCl3
151MHz
f1 (ppm)

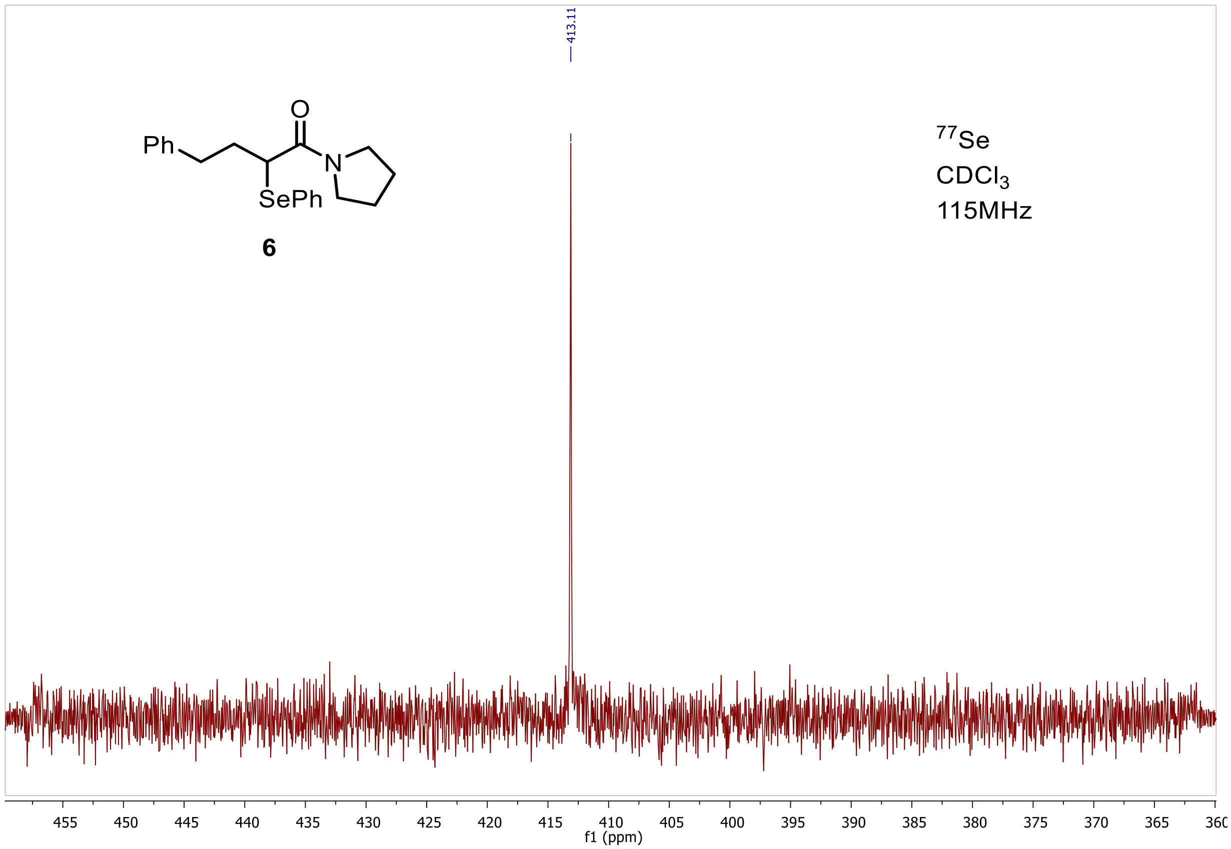

413.11
Ph
O
N
SePh
6
^{77}Se
CDCl$_3$
115MHz
455
450
445
440
435
430
425
420
415
410
405
400
395
390
385
380
375
370
365
360
f1 (ppm)

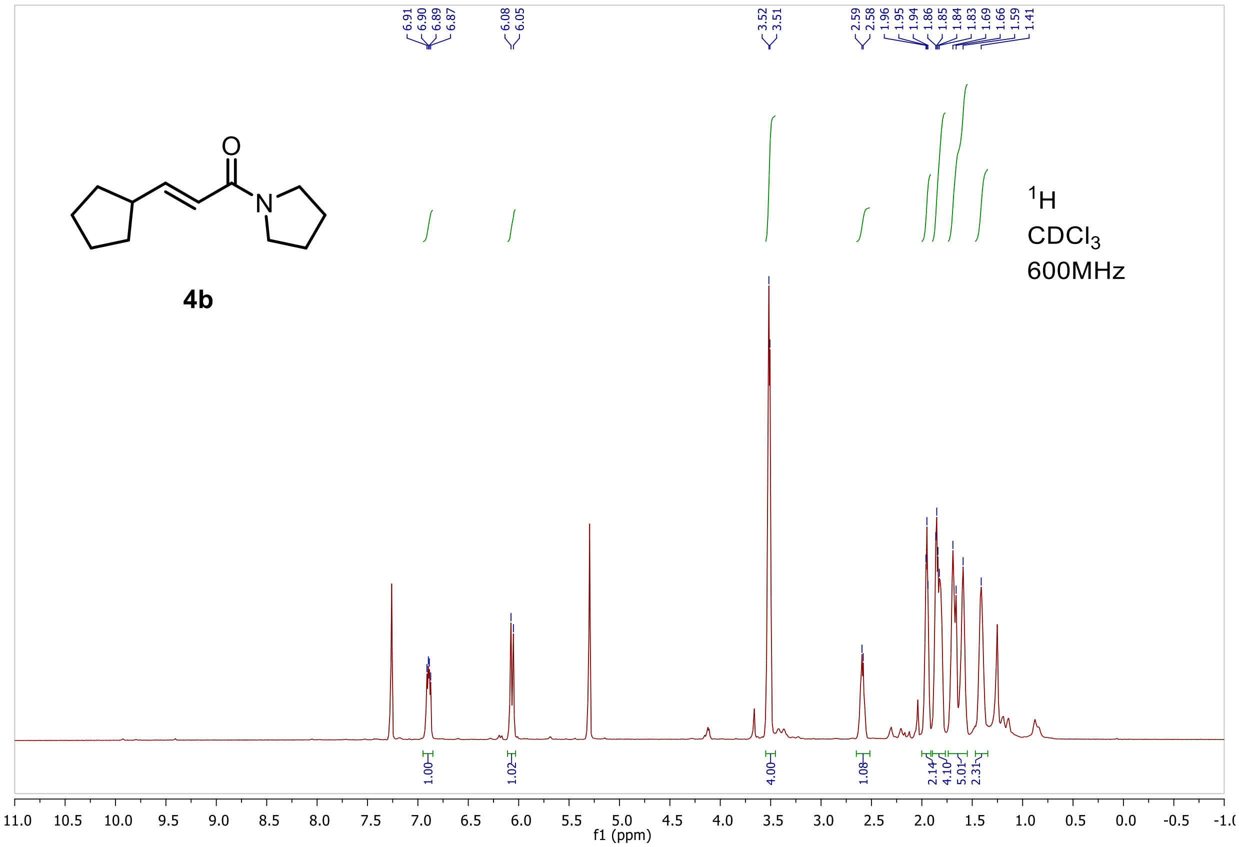
6.91
6.90
6.89
6.87
6.08
6.05
3.52
3.51
2.59
2.58
1.96
1.95
1.94
1.86
1.85
1.84
1.83
1.69
1.66
1.59
1.41
O
N
4b
^{1}H
$CDCl_3$
600MHz
1.00
1.02
4.00
1.08
2.14
4.10
5.01
2.31
11.0 10.5 10.0 9.5 9.0 8.5 8.0 7.5 7.0 6.5 6.0 5.5 5.0 4.5 4.0 3.5 3.0 2.5 2.0 1.5 1.0 0.5 0.0 -0.5
f1 (ppm)

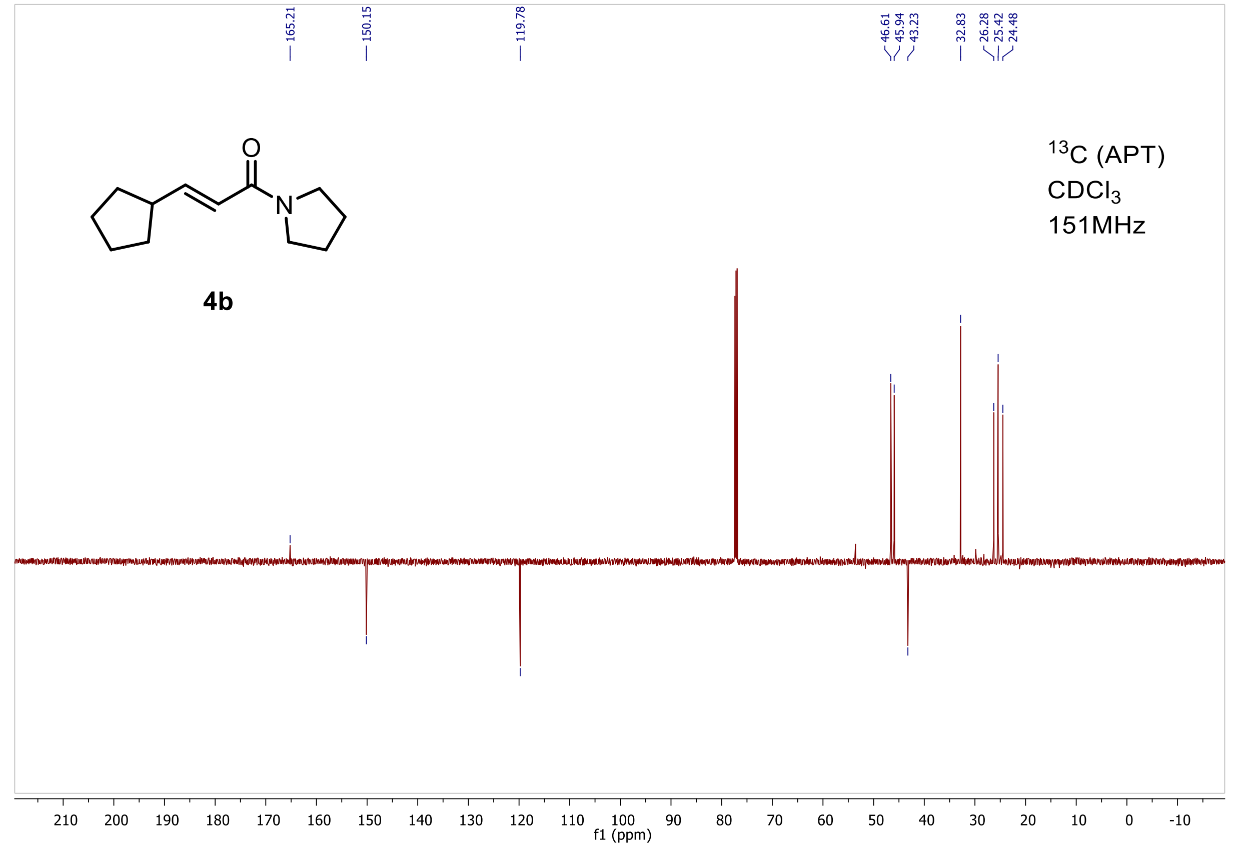
165.21
150.15
119.78
46.61
45.94
43.23
32.83
26.28
25.42
24.48
O
N
4b
^{13}C (APT)
$CDCl_3$
151MHz
210 200 190 180 170 160 150 140 130 120 110 100 90 80 70 60 50 40 30 20 10 0 -10
f1 (ppm)

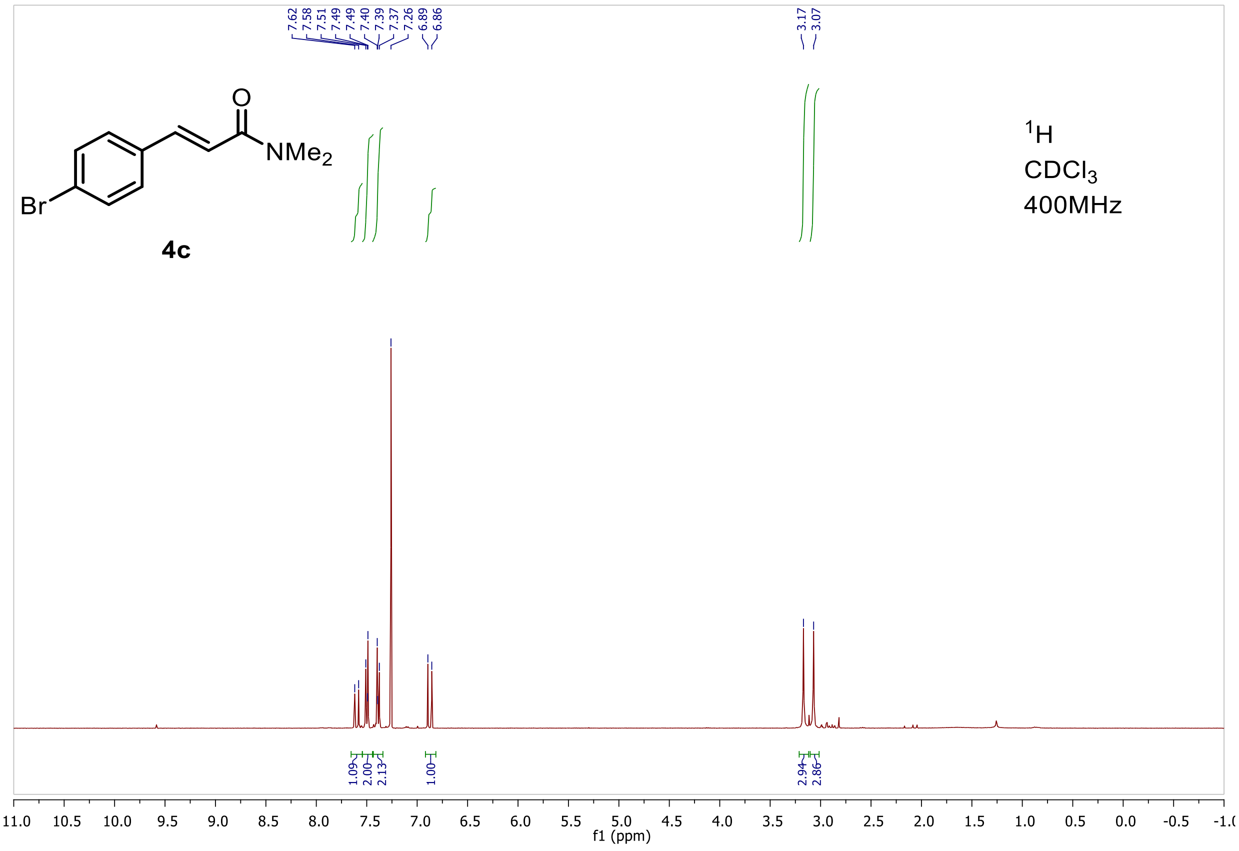
7.62
7.58
7.51
7.49
7.49
7.40
7.39
7.37
7.26
6.89
6.86
3.17
3.07
O
NMe2
Br
4c
1H
CDCl3
400MHz
1.09
2.00
2.13
1.00
2.94
2.86
11.0 10.5 10.0 9.5 9.0 8.5 8.0 7.5 7.0 6.5 6.0 5.5 5.0 4.5 4.0 3.5 3.0 2.5 2.0 1.5 1.0 0.5 0.0 -0.5
f1 (ppm)

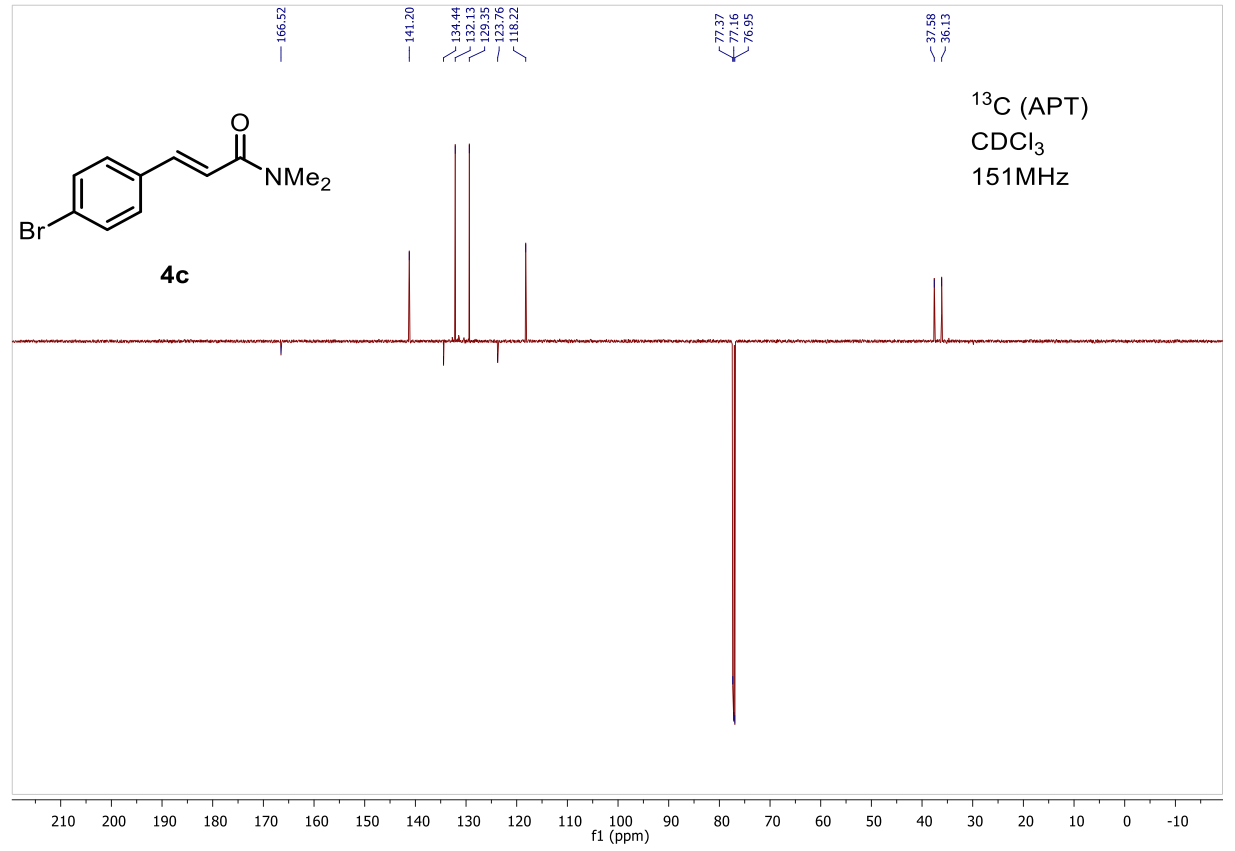
166.52
141.20
134.44
132.13
129.35
123.76
118.22
77.37
77.16
76.95
37.58
36.13
O
NMe2
Br
4c
13C (APT)
CDCl3
151MHz
210 200 190 180 170 160 150 140 130 120 110 100 90 80 70 60 50 40 30 20 10 0 -10
f1 (ppm)

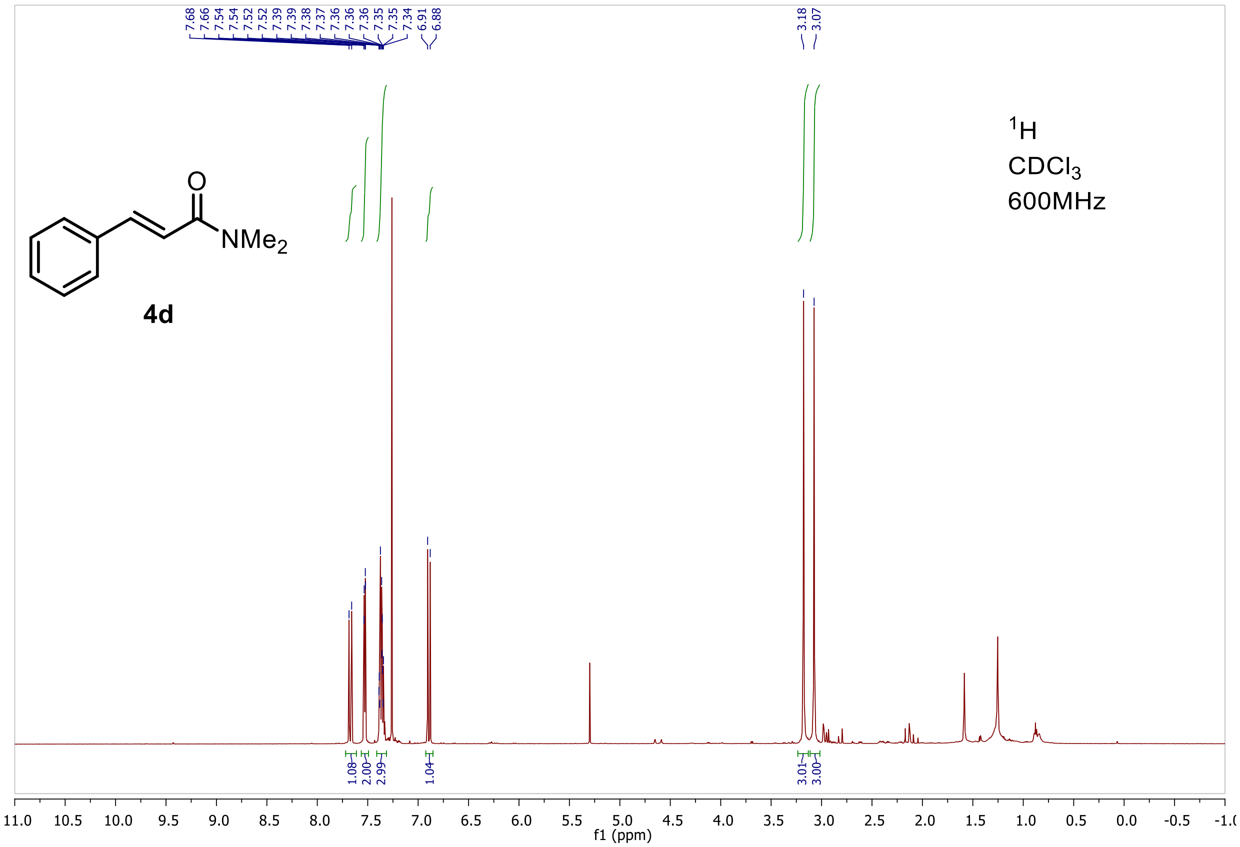

7.68 7.66 7.54 7.54 7.52 7.52 7.39 7.39 7.38 7.37 7.36 7.36 7.36 7.35 7.35 7.34 6.91 6.88
3.18 3.07
NMe2
O
4d
1H
CDCl3
600MHz
1.08 2.00 2.99 1.04 3.01 3.00
f1 (ppm)

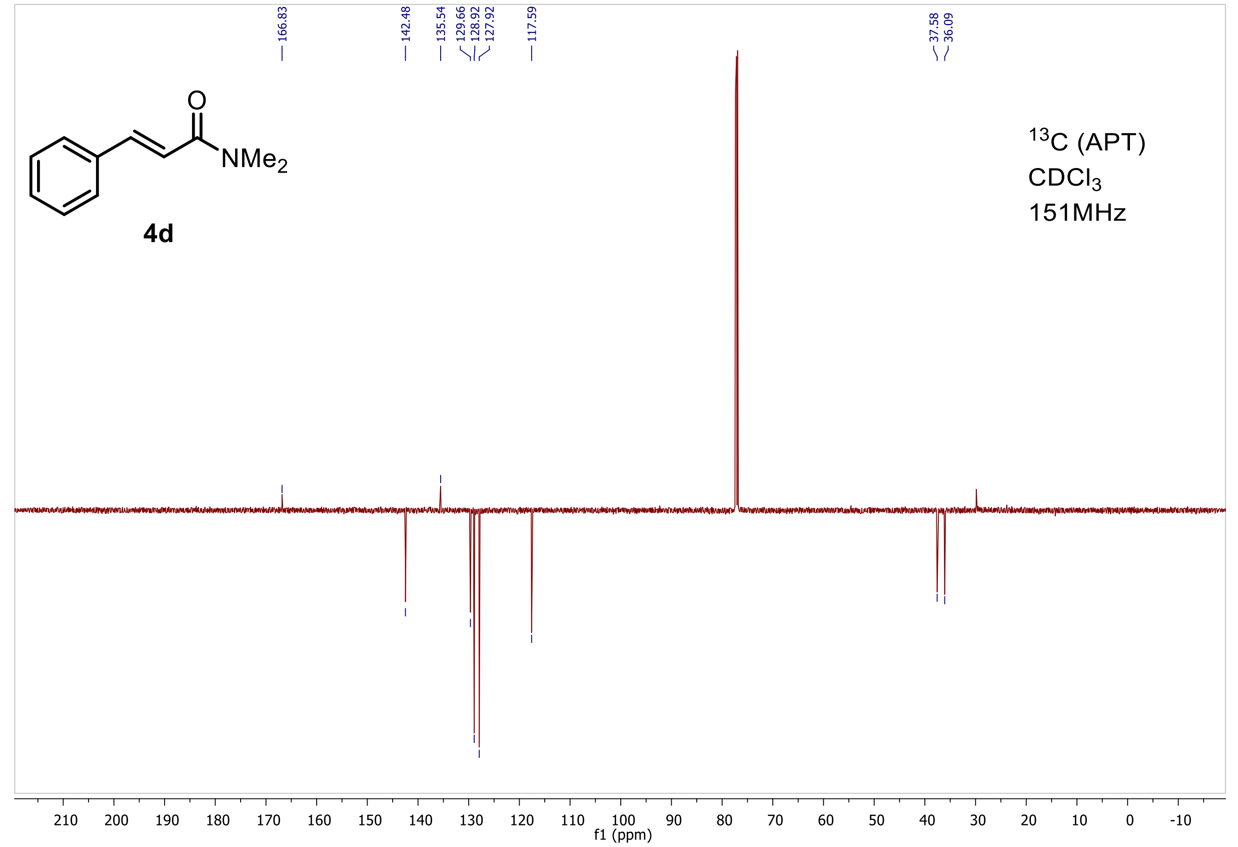

166.83 142.48 135.54 129.66 128.92 127.92 117.59 37.58 36.09
NMe2
O
4d
13C (APT)
CDCl3
151MHz
f1 (ppm)

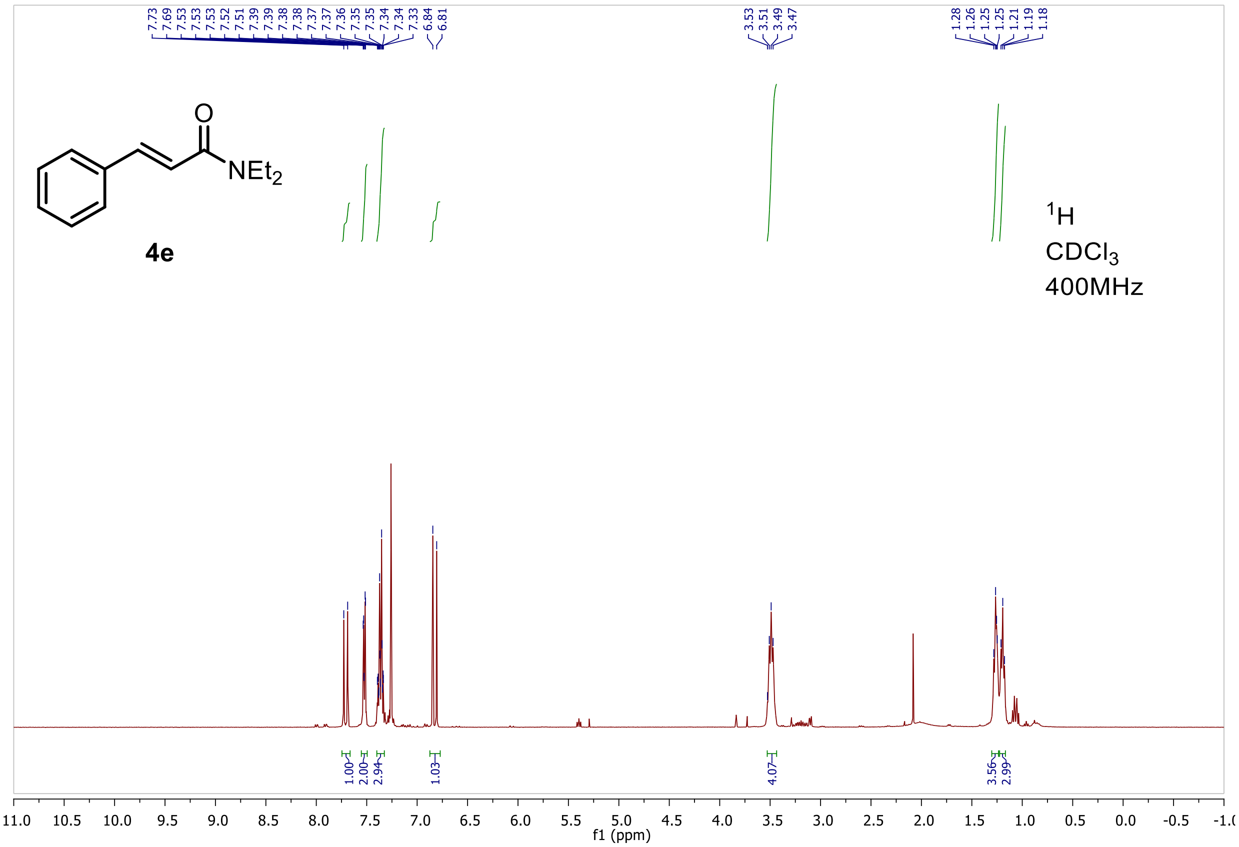
7.73
7.69
7.53
7.53
7.53
7.52
7.51
7.39
7.39
7.38
7.38
7.37
7.37
7.36
7.35
7.35
7.34
7.34
7.33
6.84
6.81
3.53
3.51
3.49
3.47
1.28
1.26
1.25
1.25
1.21
1.19
1.18
O
NEt2
4e
1H
CDCl3
400MHz
1.00
2.00
2.94
1.03
4.07
3.56
2.99
11.0 10.5 10.0 9.5 9.0 8.5 8.0 7.5 7.0 6.5 6.0 5.5 5.0 4.5 4.0 3.5 3.0 2.5 2.0 1.5 1.0 0.5 0.0 -0.5
f1 (ppm)

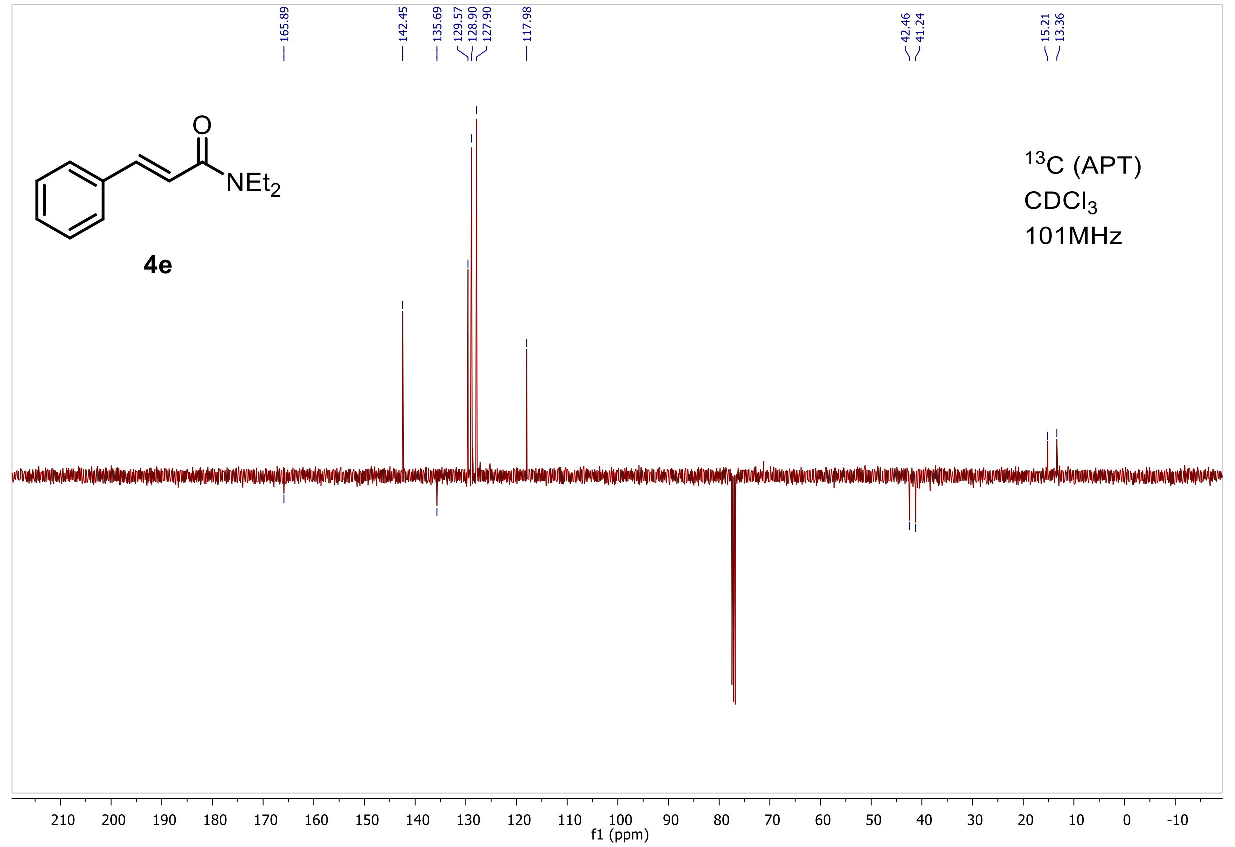
165.89
142.45
135.69
129.57
128.90
127.90
117.98
42.46
41.24
15.21
13.36
O
NEt2
4e
13C (APT)
CDCl3
101MHz
210 200 190 180 170 160 150 140 130 120 110 100 90 80 70 60 50 40 30 20 10 0 -10
f1 (ppm)

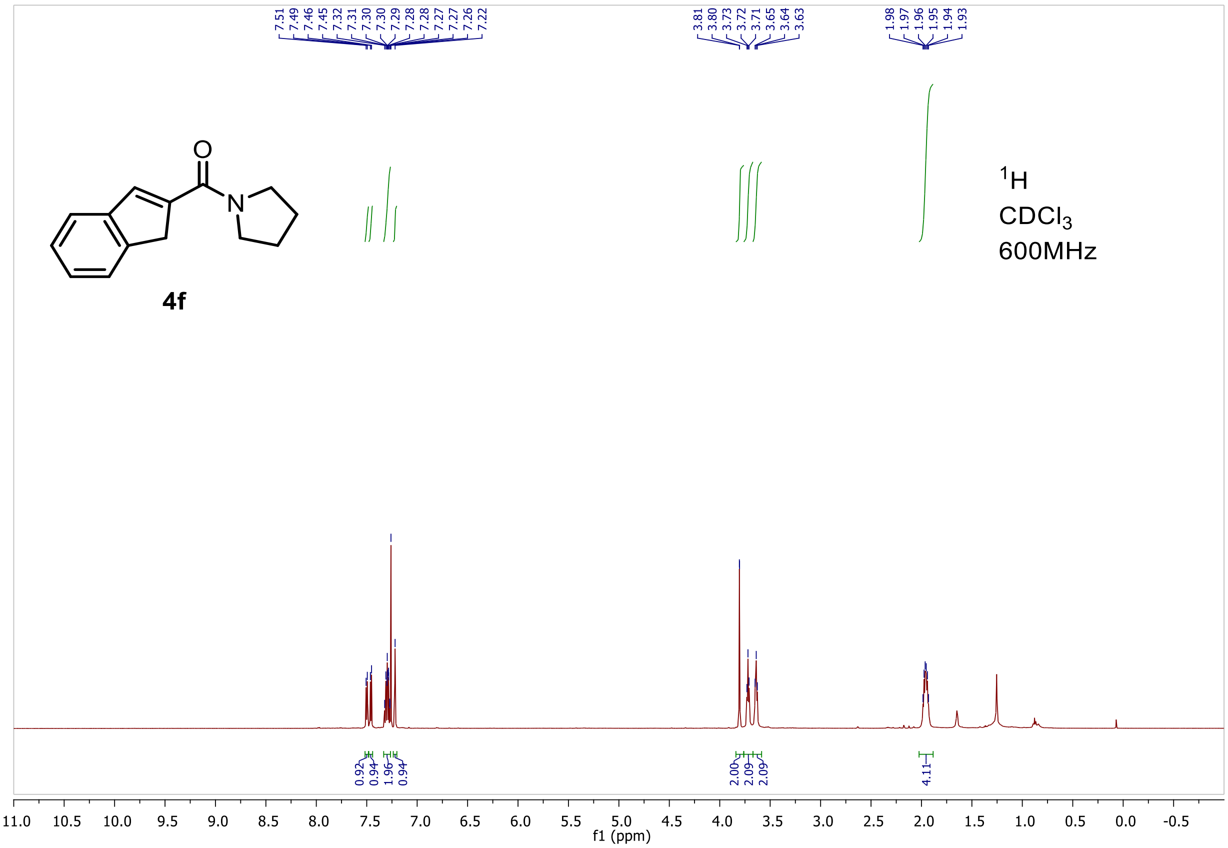
7.51 7.49 7.46 7.45 7.32 7.31 7.30 7.30 7.29 7.28 7.28 7.27 7.27 7.26 7.22
3.81 3.80 3.73 3.72 3.71 3.65 3.64 3.63
1.98 1.97 1.96 1.95 1.94 1.93
O
N
4f
^{1}H
CDCl$_3$
600MHz
0.92 0.94 1.96 0.94
2.00 2.09 2.09
4.11
11.0 10.5 10.0 9.5 9.0 8.5 8.0 7.5 7.0 6.5 6.0 5.5 5.0 4.5 4.0 3.5 3.0 2.5 2.0 1.5 1.0 0.5 0.0 -0.5
f1 (ppm)

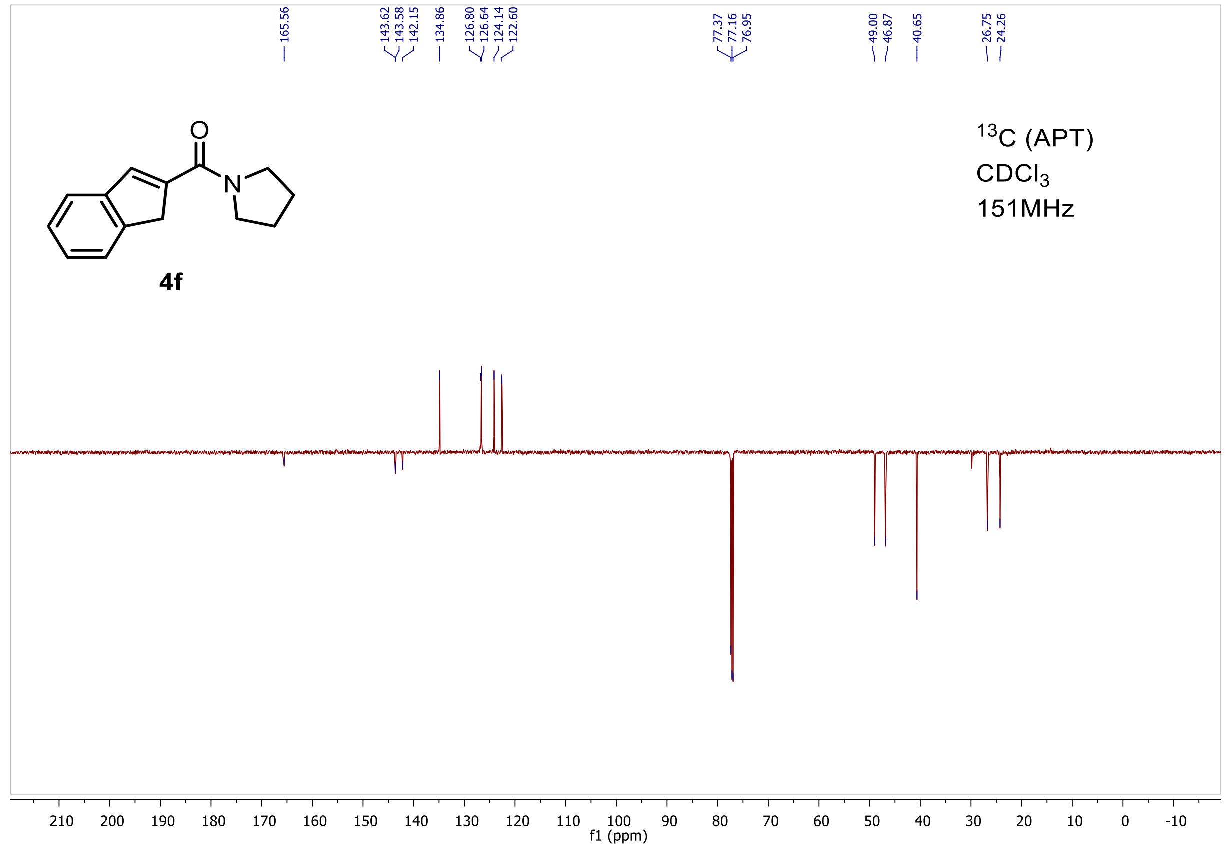
165.56
143.62 143.58 142.15
134.86
126.80 126.64 124.14 122.60
77.37 77.16 76.95
49.00 46.87
40.65
26.75 24.26
O
N
4f
^{13}C (APT)
CDCl$_3$
151MHz
210 200 190 180 170 160 150 140 130 120 110 100 90 80 70 60 50 40 30 20 10 0 -10
f1 (ppm)

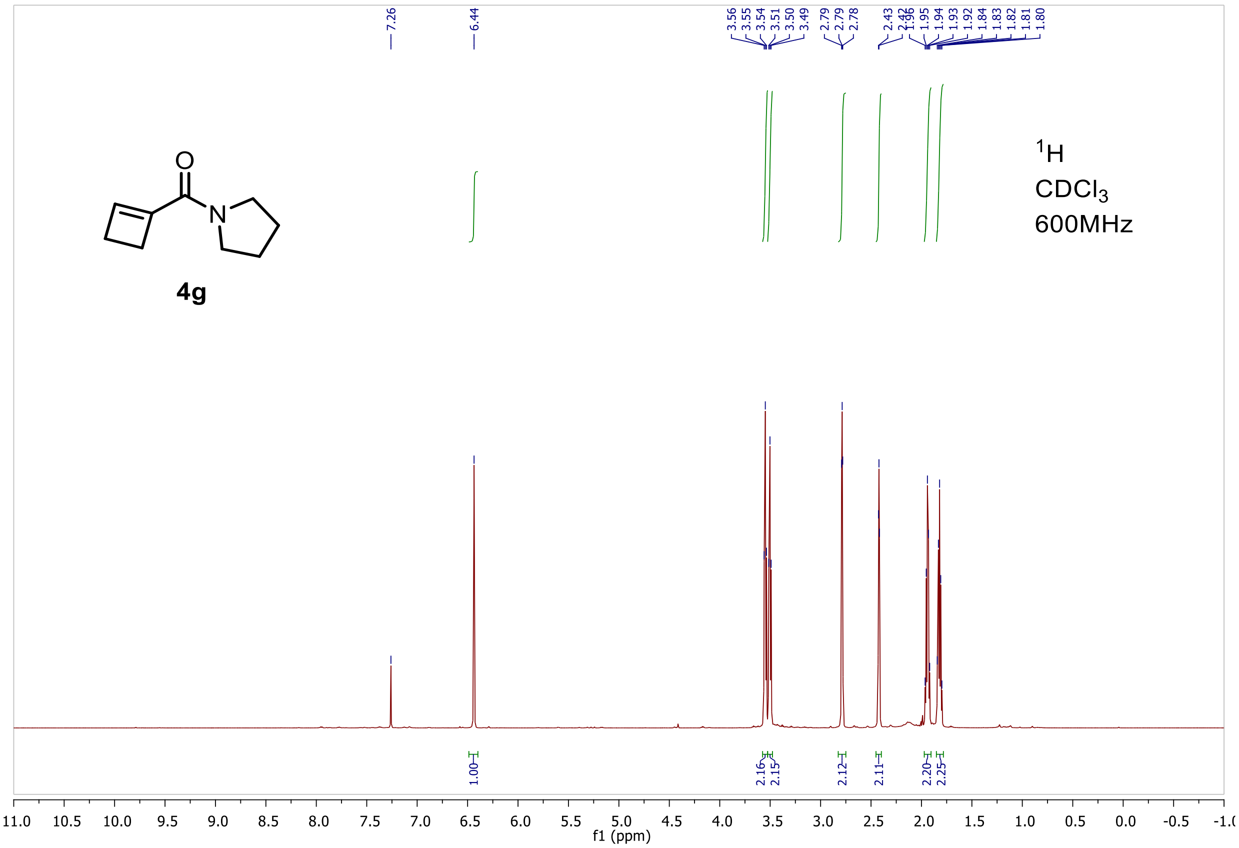

7.26
6.44
3.56 3.55 3.54 3.51 3.50 3.49
2.79 2.79 2.78
2.43 2.42
1.96 1.95 1.94 1.93 1.92 1.84 1.83 1.82 1.81 1.80
4g
^{1}H
$CDCl_3$
600MHz
1.00
2.16 2.15
2.12
2.11
2.20 2.25
f1 (ppm)

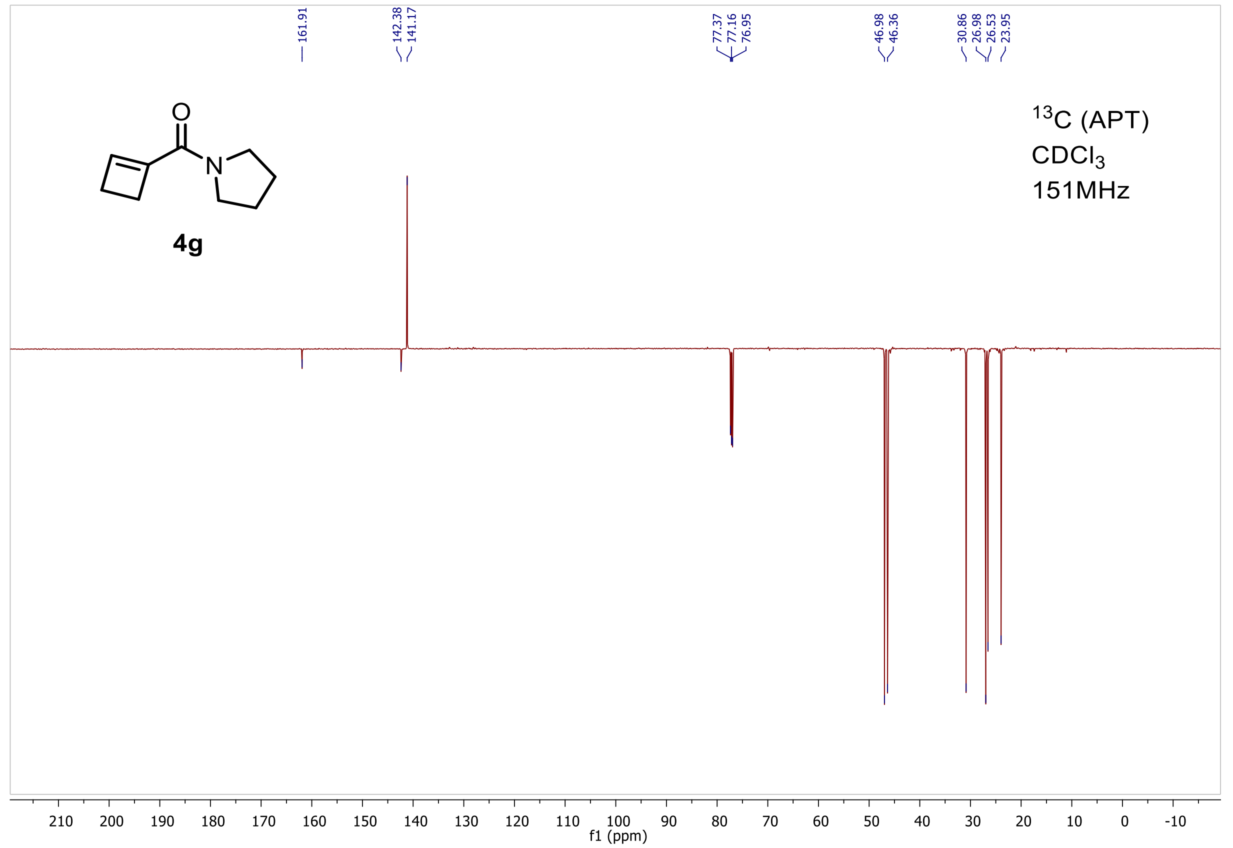

161.91
142.38 141.17
77.37 77.16 76.95
46.98 46.36
30.86 26.98 26.53 23.95
4g
^{13}C (APT)
$CDCl_3$
151MHz
f1 (ppm)

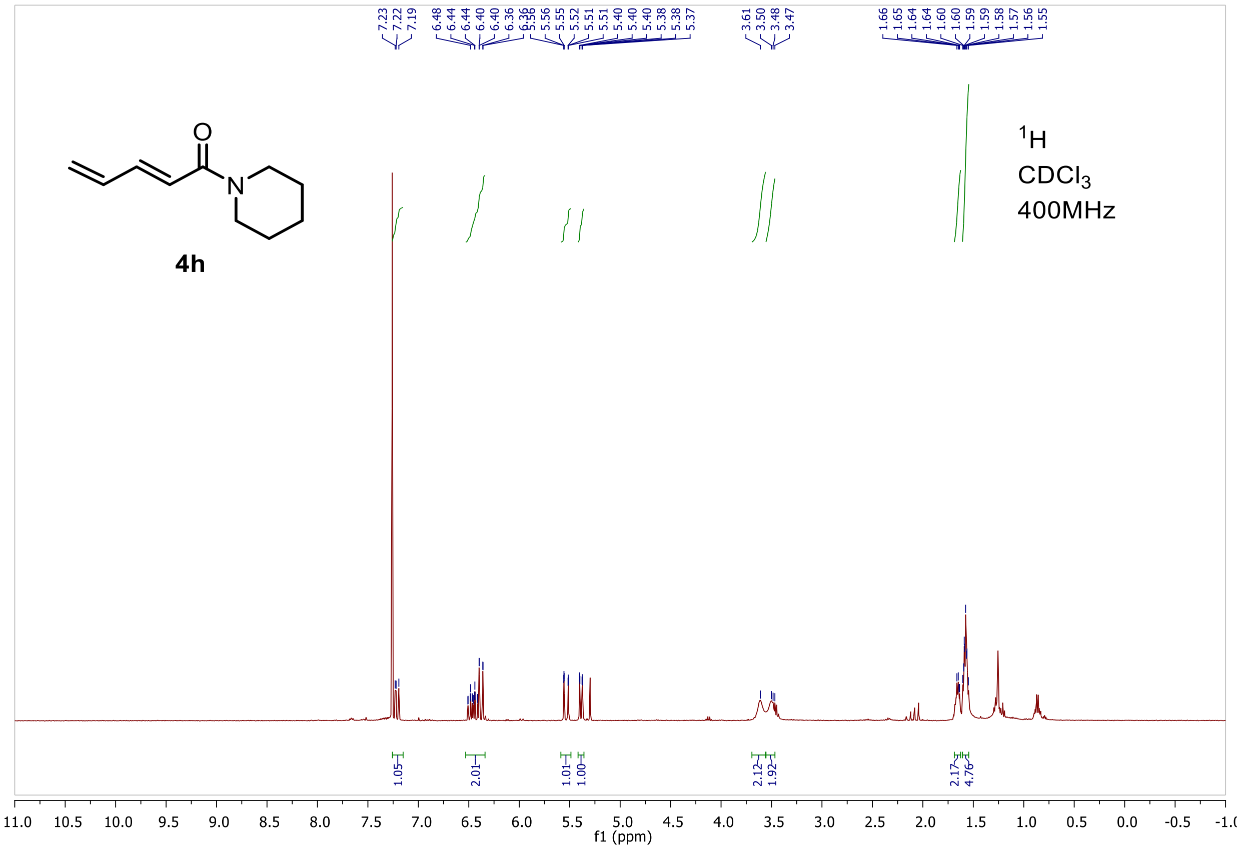

4h
^{1}H
$CDCl_3$
400MHz
f1 (ppm)

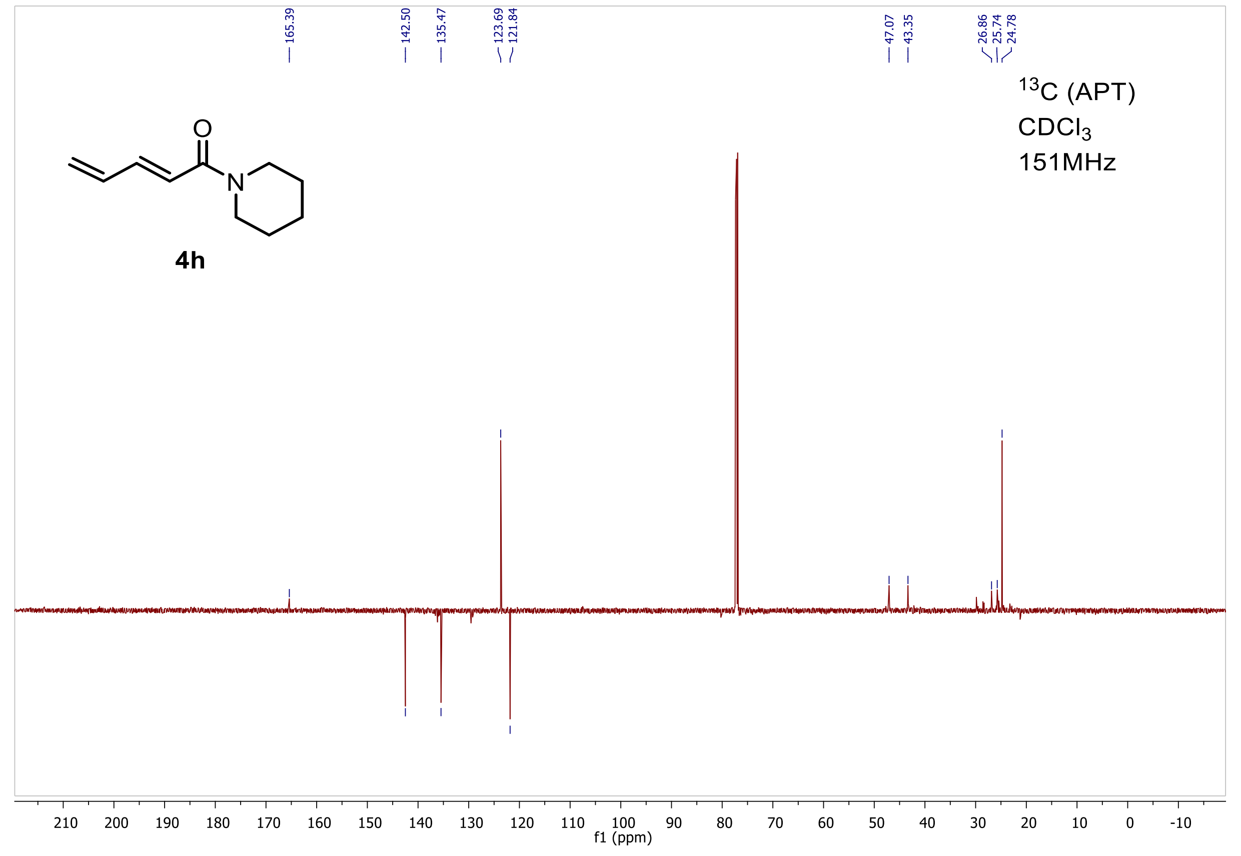

4h
^{13}C (APT)
$CDCl_3$
151MHz
f1 (ppm)

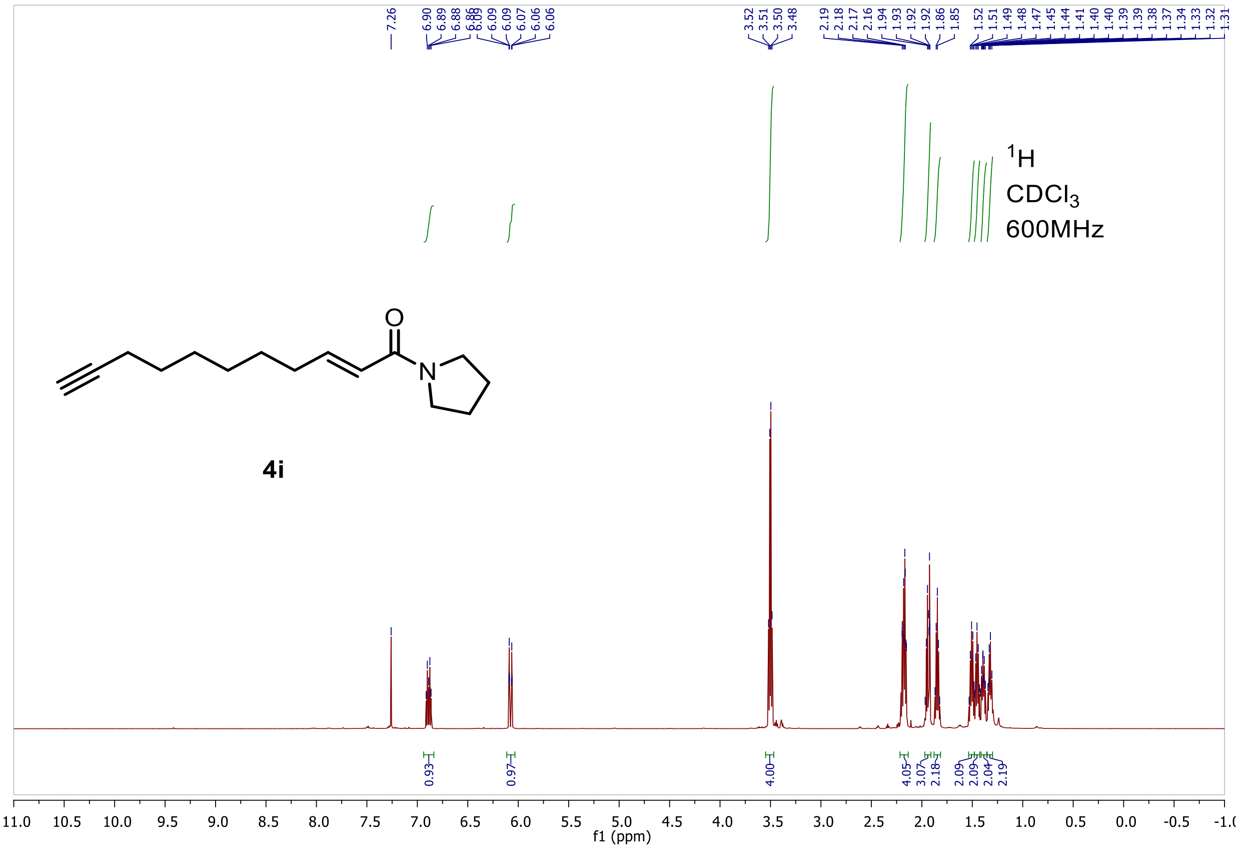

^{1}H
CDCl$_3$
600MHz
4i
f1 (ppm)

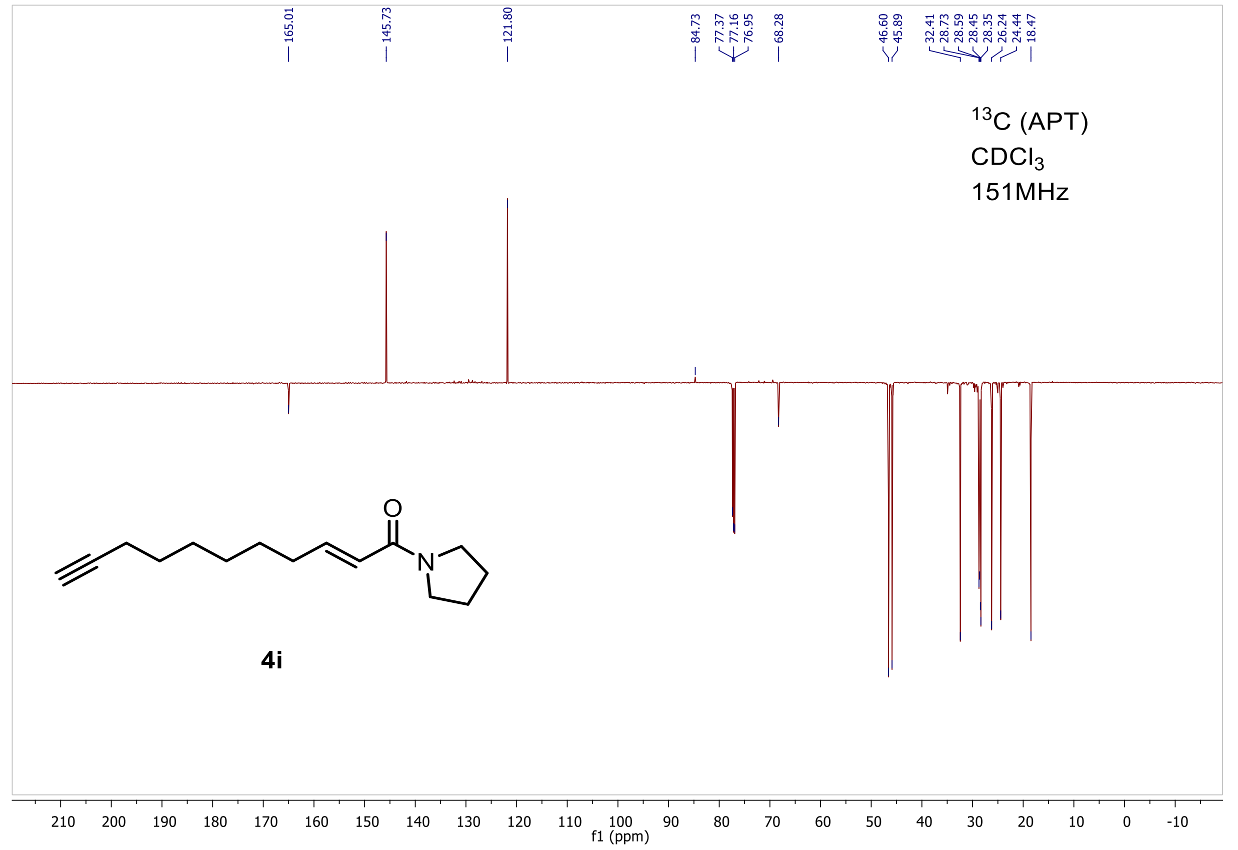

^{13}C (APT)
CDCl$_3$
151MHz
4i
f1 (ppm)

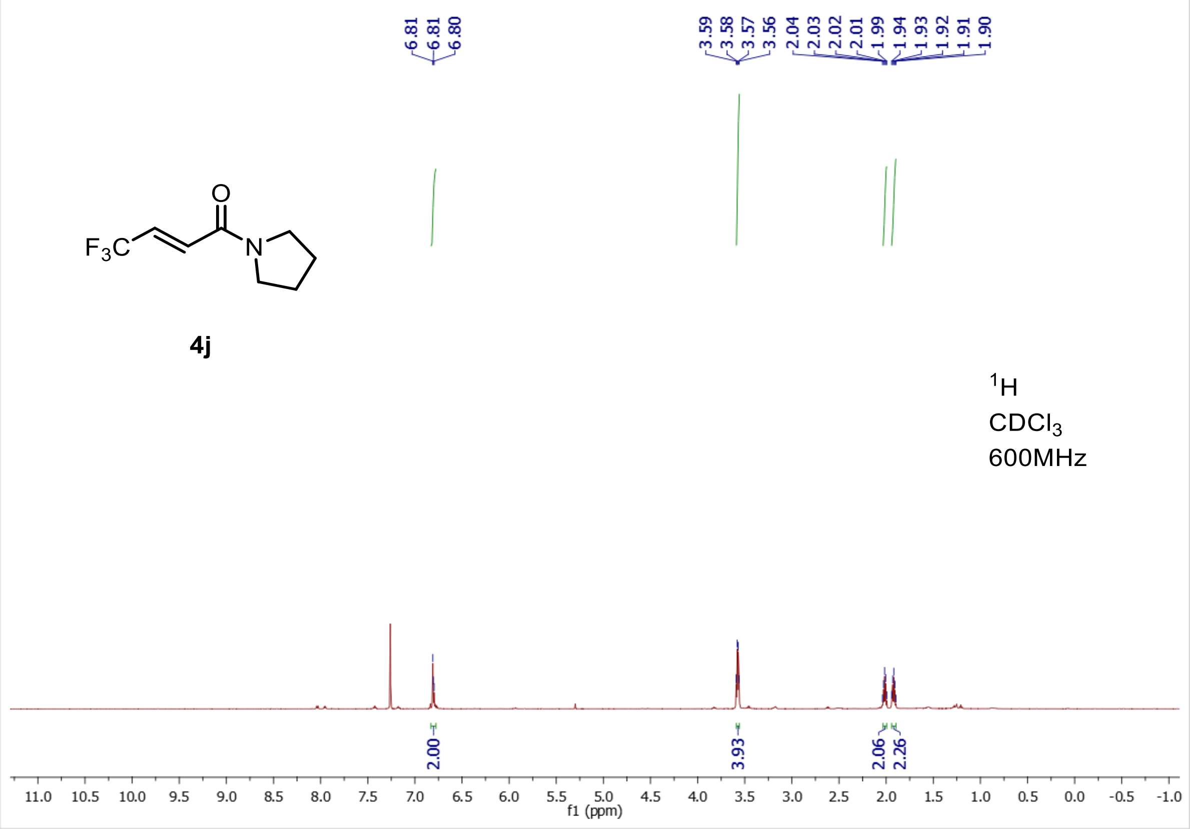
6.81
6.81
6.80
3.59
3.58
3.57
3.56
2.04
2.03
2.02
2.01
1.99
1.94
1.93
1.92
1.91
1.90
F_3C
O
N
4j
^1H
$CDCl_3$
600MHz
2.00
3.93
2.06
2.26
f1 (ppm)

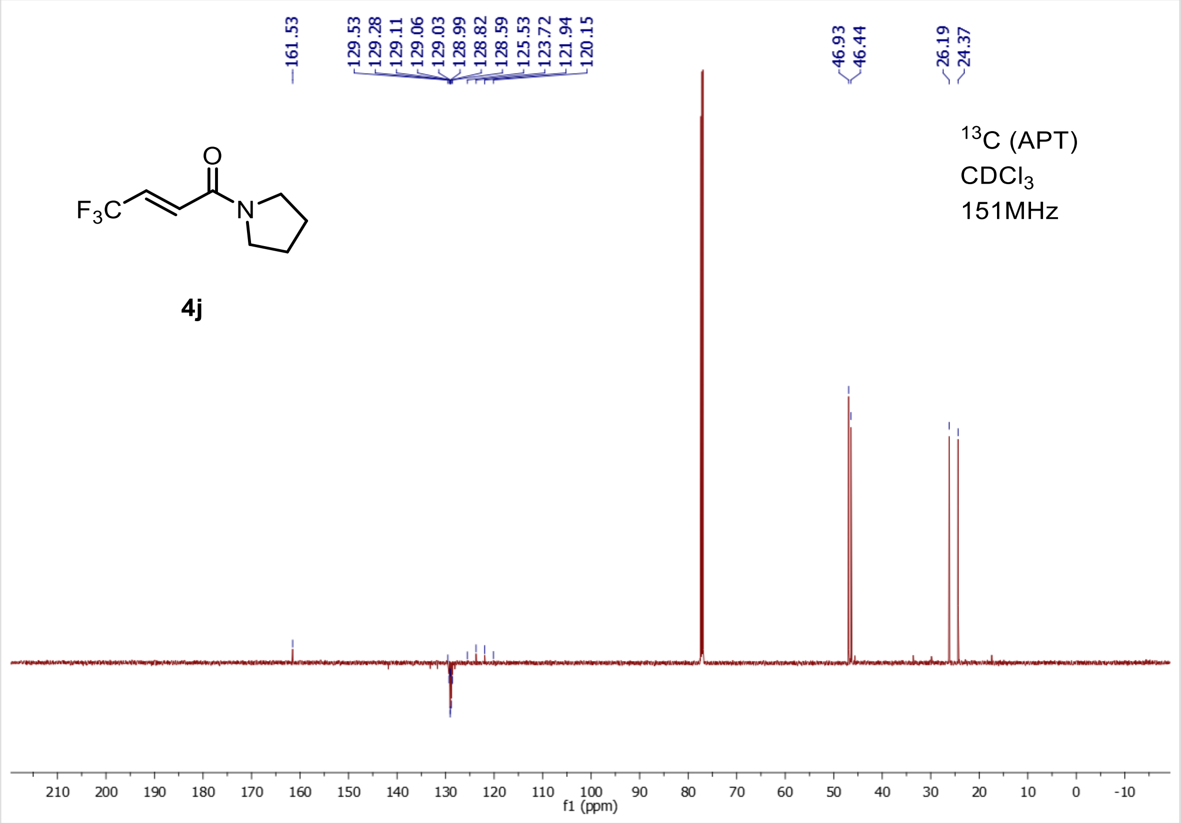
161.53
129.53
129.28
129.11
129.06
129.03
128.99
128.82
128.59
125.53
123.72
121.94
120.15
46.93
46.44
26.19
24.37
F_3C
O
N
4j
^{13}C (APT)
$CDCl_3$
151MHz
f1 (ppm)

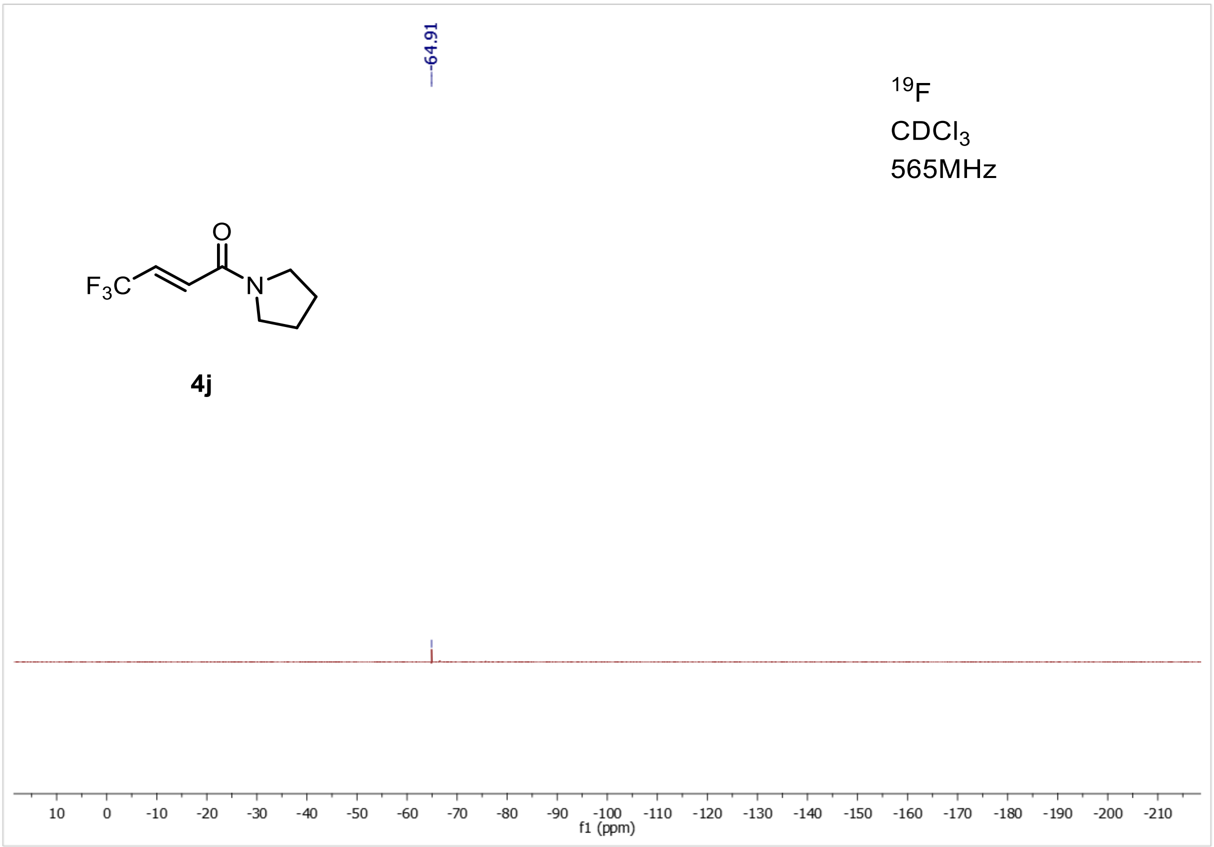

-64.91
^{19}F
$CDCl_3$
565MHz
F_3C
O
N
4j
10 0 -10 -20 -30 -40 -50 -60 -70 -80 -90 -100 -110 -120 -130 -140 -150 -160 -170 -180 -190 -200 -210
f1 (ppm)

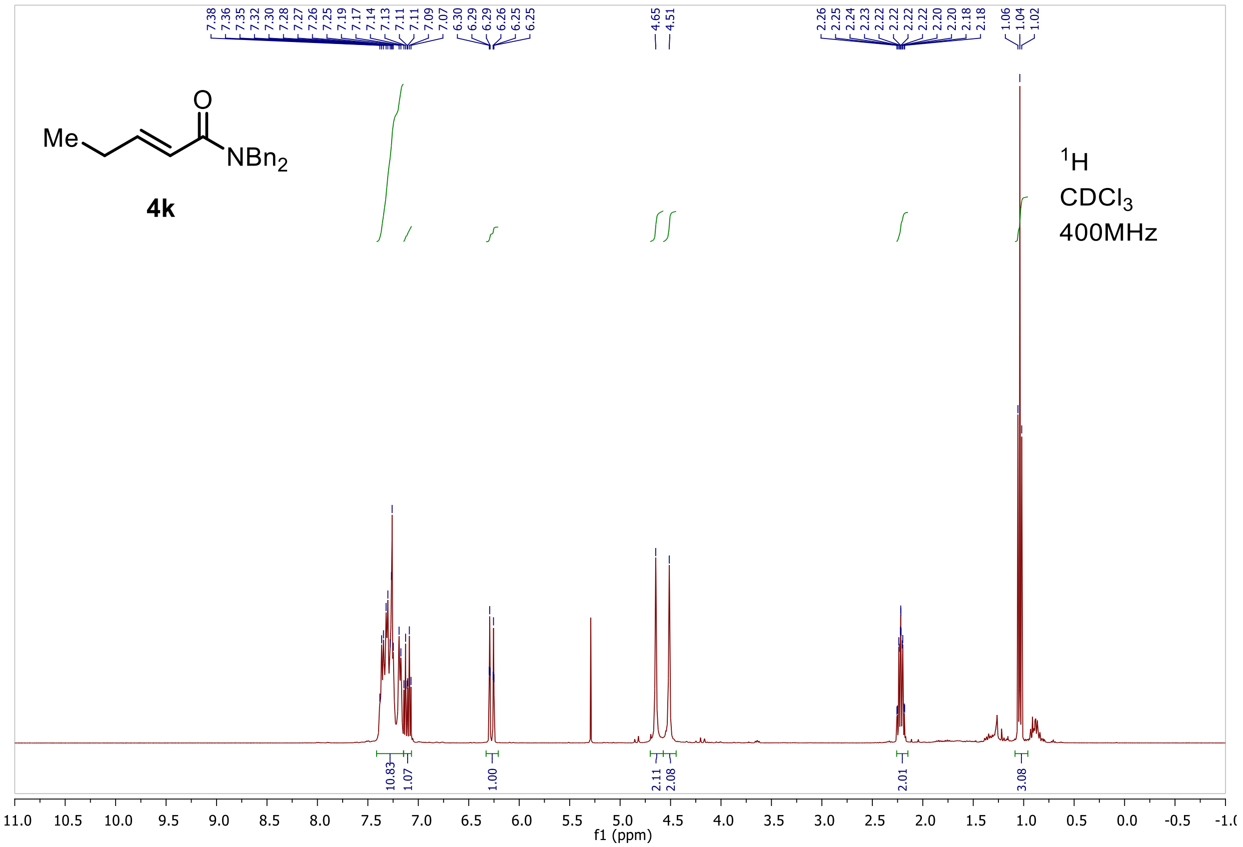

Me
O
NBn2
4k
1H
CDCl3
400MHz
f1 (ppm)

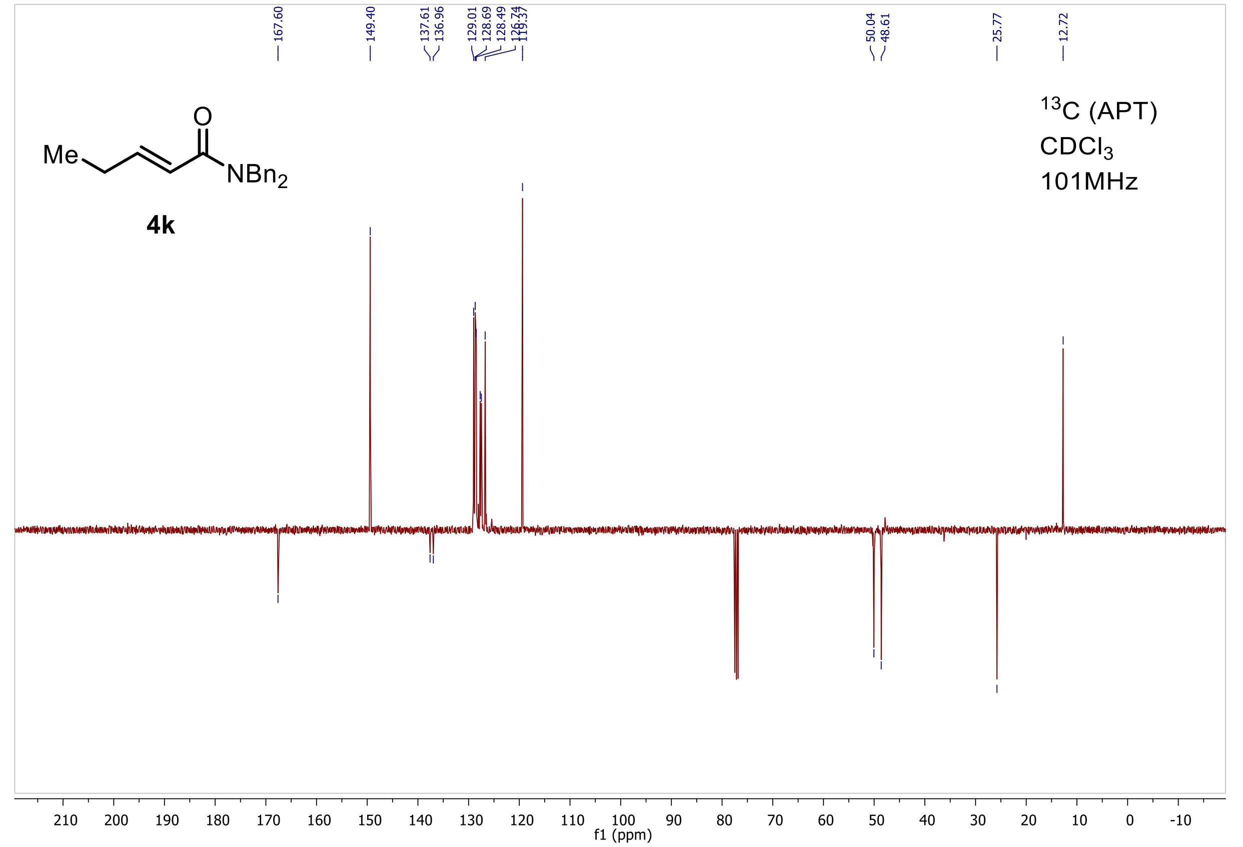

Me
O
NBn2
4k
13C (APT)
CDCl3
101MHz
f1 (ppm)

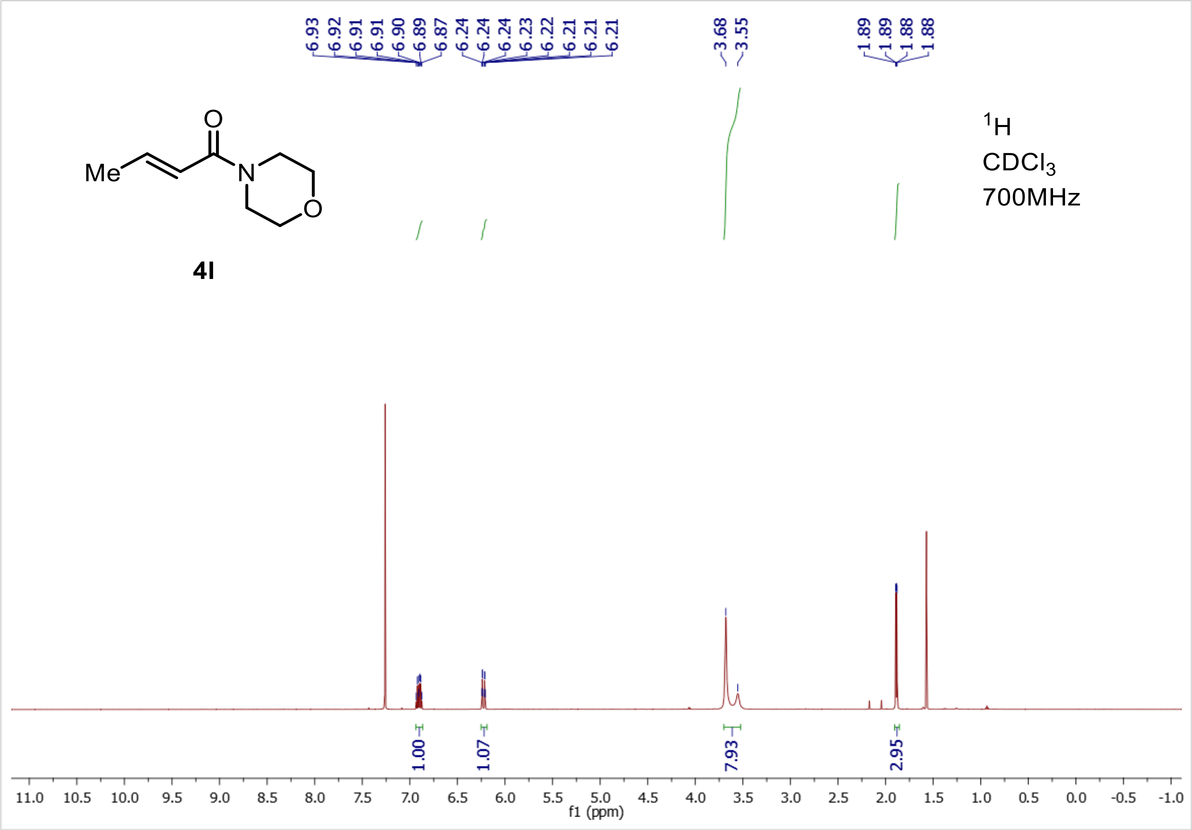
6.93
6.92
6.91
6.91
6.90
6.89
6.87
6.24
6.24
6.24
6.23
6.22
6.21
6.21
6.21
3.68
3.55
1.89
1.89
1.88
1.88
O
Me
N
O
4l
^{1}H
CDCl$_3$
700MHz
1.00
1.07
7.93
2.95
11.0 10.5 10.0 9.5 9.0 8.5 8.0 7.5 7.0 6.5 6.0 5.5 5.0 4.5 4.0 3.5 3.0 2.5 2.0 1.5 1.0 0.5 0.0 -0.5 -1.0
f1 (ppm)

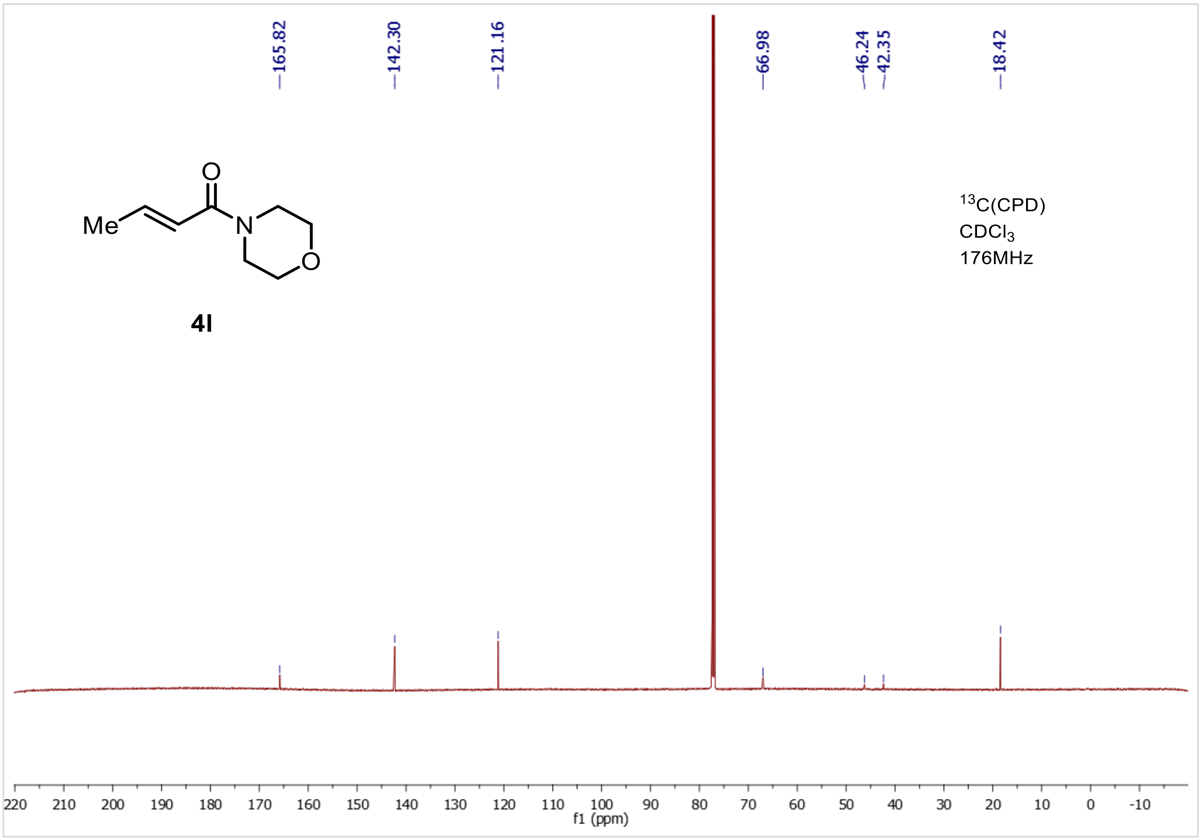
165.82
142.30
121.16
66.98
46.24
42.35
18.42
O
Me
N
O
4l
^{13}C(CPD)
CDCl$_3$
176MHz
220 210 200 190 180 170 160 150 140 130 120 110 100 90 80 70 60 50 40 30 20 10 0 -10
f1 (ppm)

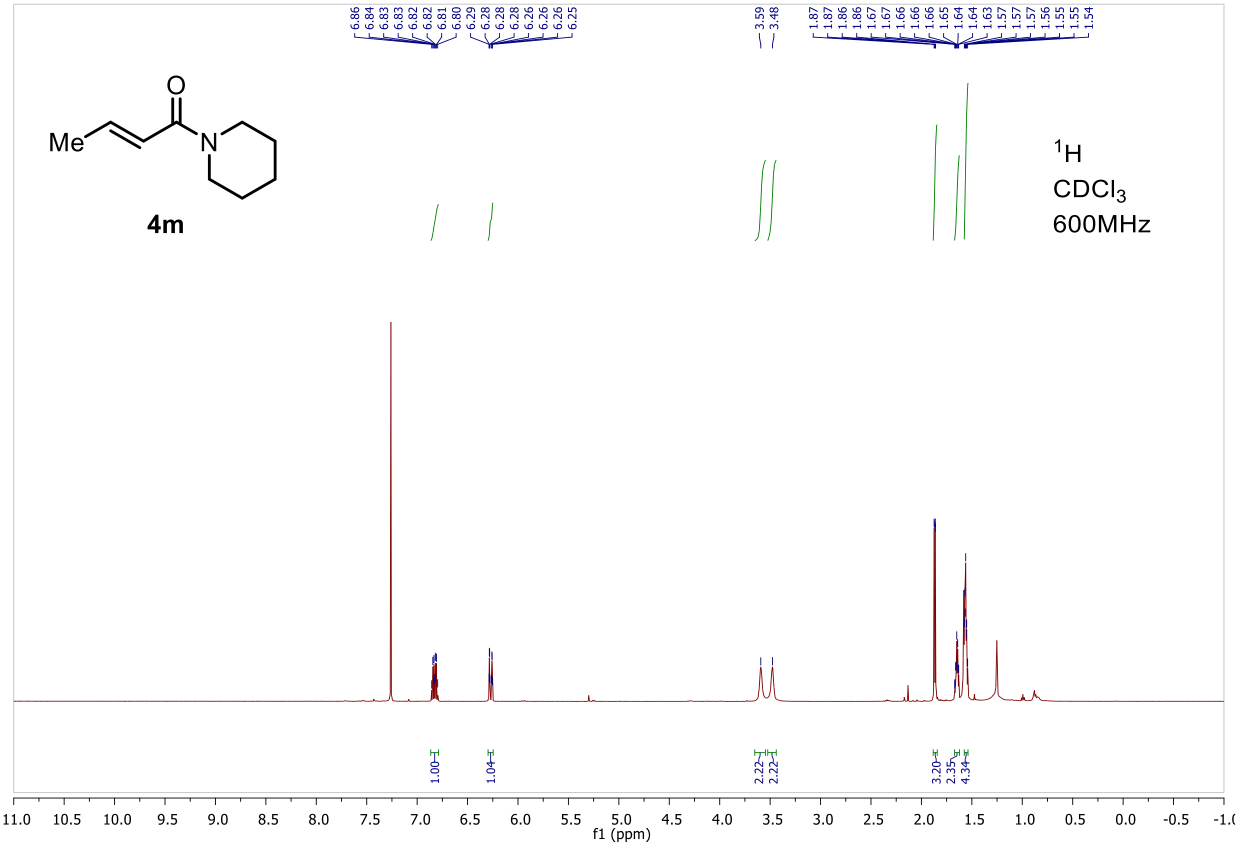
6.86 6.84 6.83 6.83 6.82 6.82 6.81 6.80 6.29 6.28 6.28 6.28 6.26 6.26 6.26 6.25
3.59 3.48
1.87 1.87 1.86 1.86 1.67 1.67 1.66 1.66 1.65 1.64 1.64 1.63 1.57 1.57 1.57 1.56 1.55 1.55 1.54
Me
O
N
4m
1H
CDCl3
600MHz
1.00
1.04
2.22
2.22
3.20
2.35
4.34
f1 (ppm)

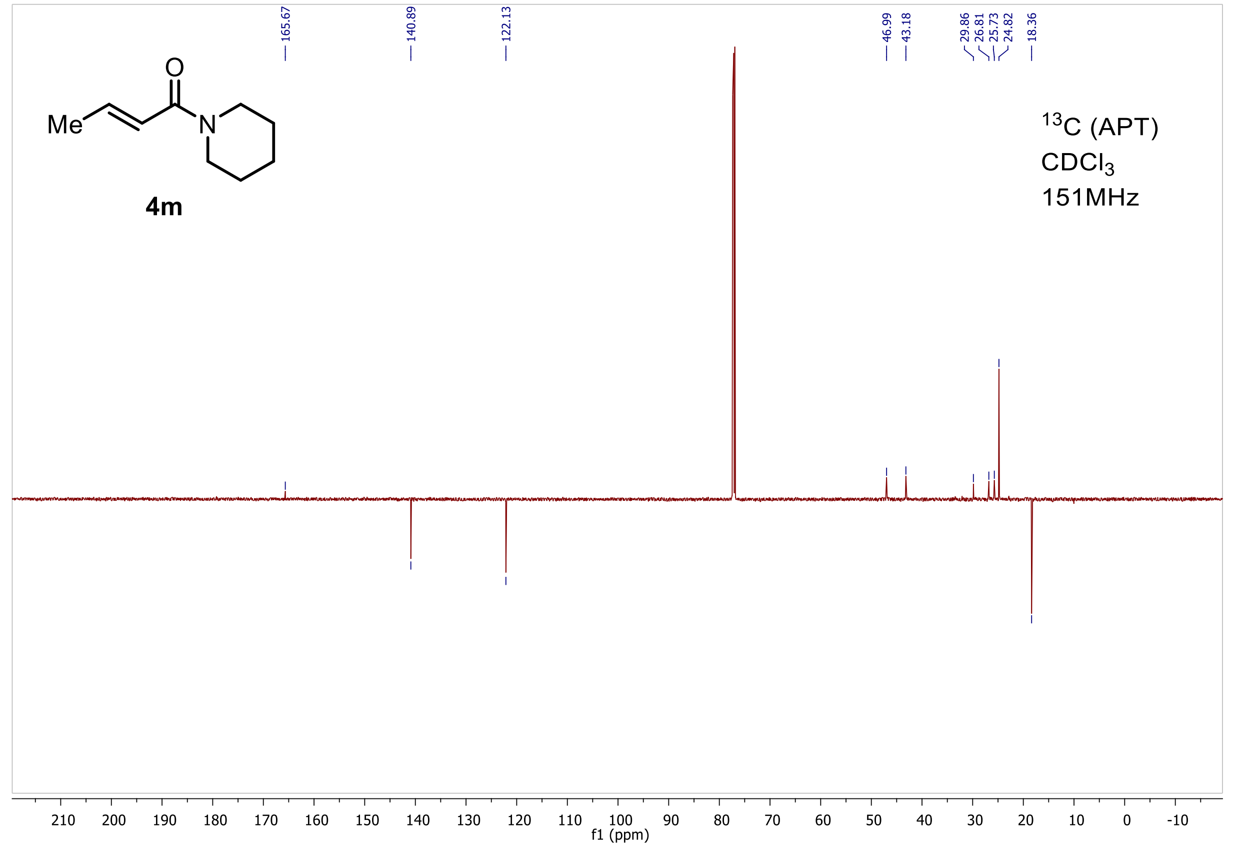
165.67
140.89
122.13
46.99
43.18
29.86 26.81 25.73 24.82
18.36
Me
O
N
4m
13C (APT)
CDCl3
151MHz
f1 (ppm)

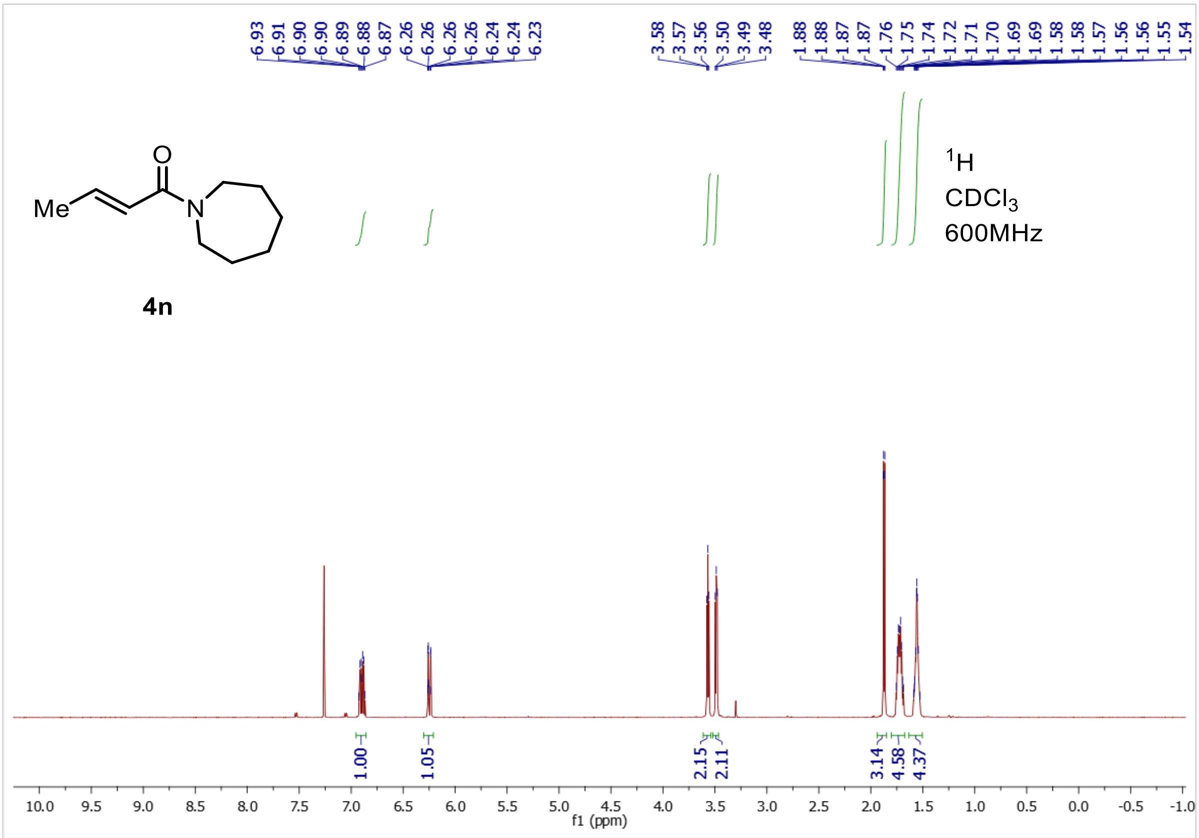

6.93
6.91
6.90
6.90
6.89
6.88
6.87
6.26
6.26
6.26
6.26
6.24
6.24
6.23
3.58
3.57
3.56
3.50
3.49
3.48
1.88
1.88
1.87
1.87
1.76
1.75
1.74
1.72
1.71
1.70
1.69
1.69
1.58
1.58
1.57
1.56
1.56
1.55
1.54
O
Me
N
4n
1H
CDCl3
600MHz
1.00
1.05
2.15
2.11
3.14
4.58
4.37
f1 (ppm)

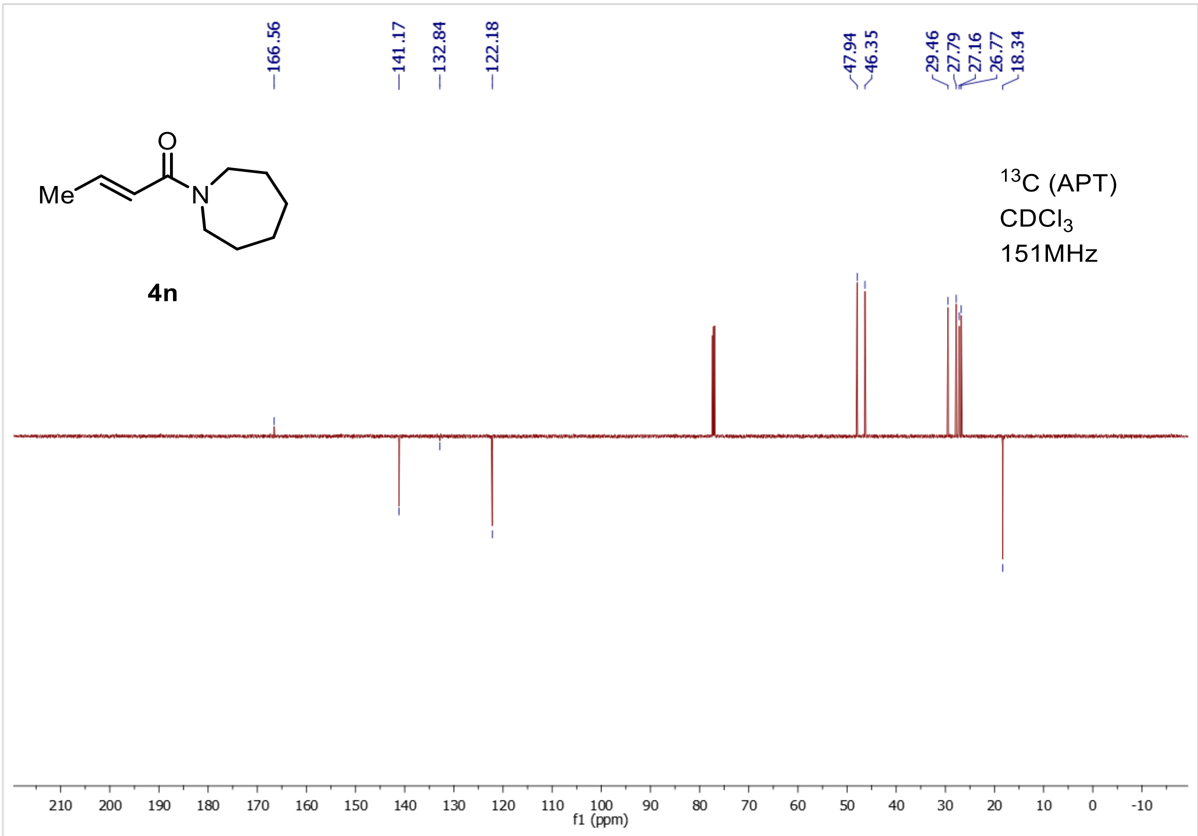

166.56
141.17
132.84
122.18
47.94
46.35
29.46
27.79
27.16
26.77
18.34
O
Me
N
4n
13C (APT)
CDCl3
151MHz
f1 (ppm)

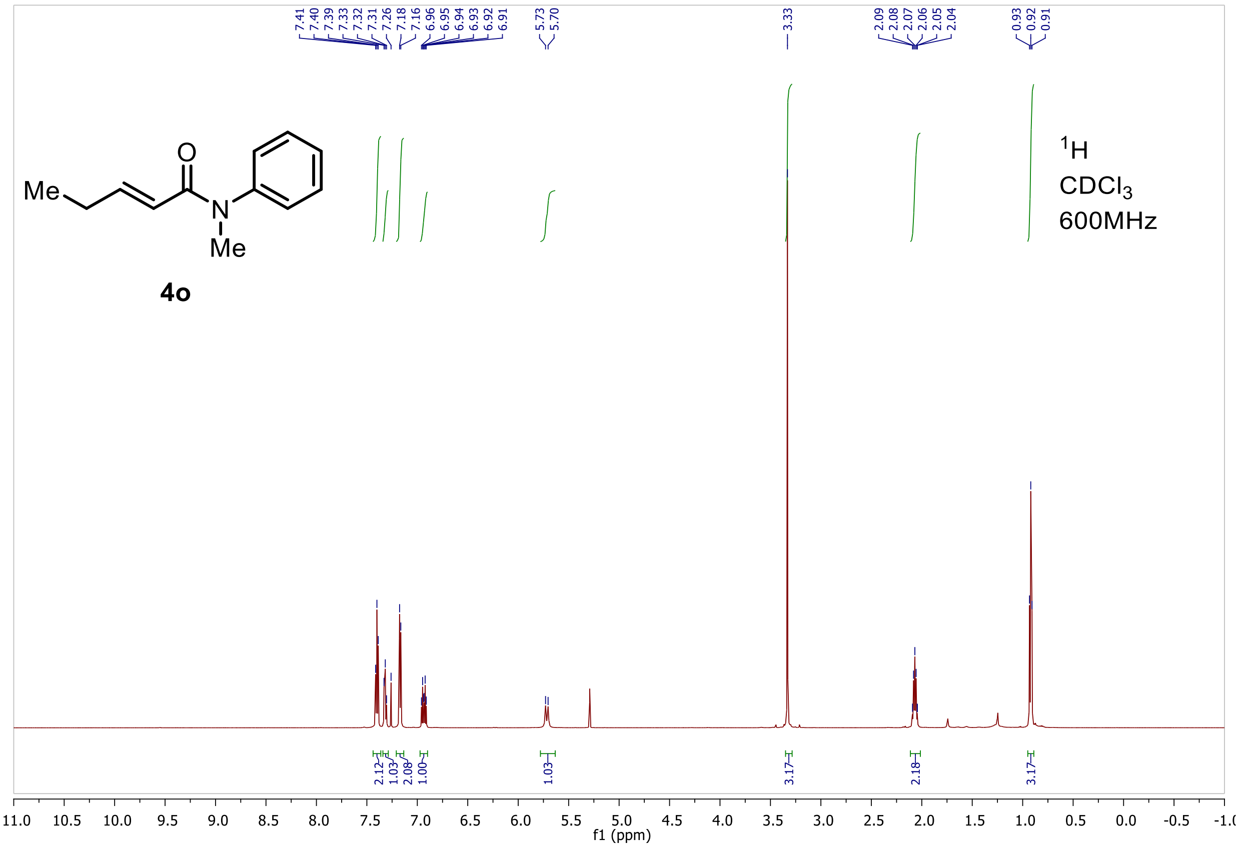
7.41
7.40
7.39
7.33
7.32
7.31
7.26
7.18
7.16
6.96
6.95
6.94
6.93
6.92
6.91
5.73
5.70
3.33
2.09
2.08
2.07
2.06
2.05
2.04
0.93
0.92
0.91
Me
O
N
Me
4o
^{1}H
$CDCl_3$
600MHz
2.12
1.03
2.08
1.00
1.03
3.17
2.18
3.17
11.0 10.5 10.0 9.5 9.0 8.5 8.0 7.5 7.0 6.5 6.0 5.5 5.0 4.5 4.0 3.5 3.0 2.5 2.0 1.5 1.0 0.5 0.0 -0.5
f1 (ppm)

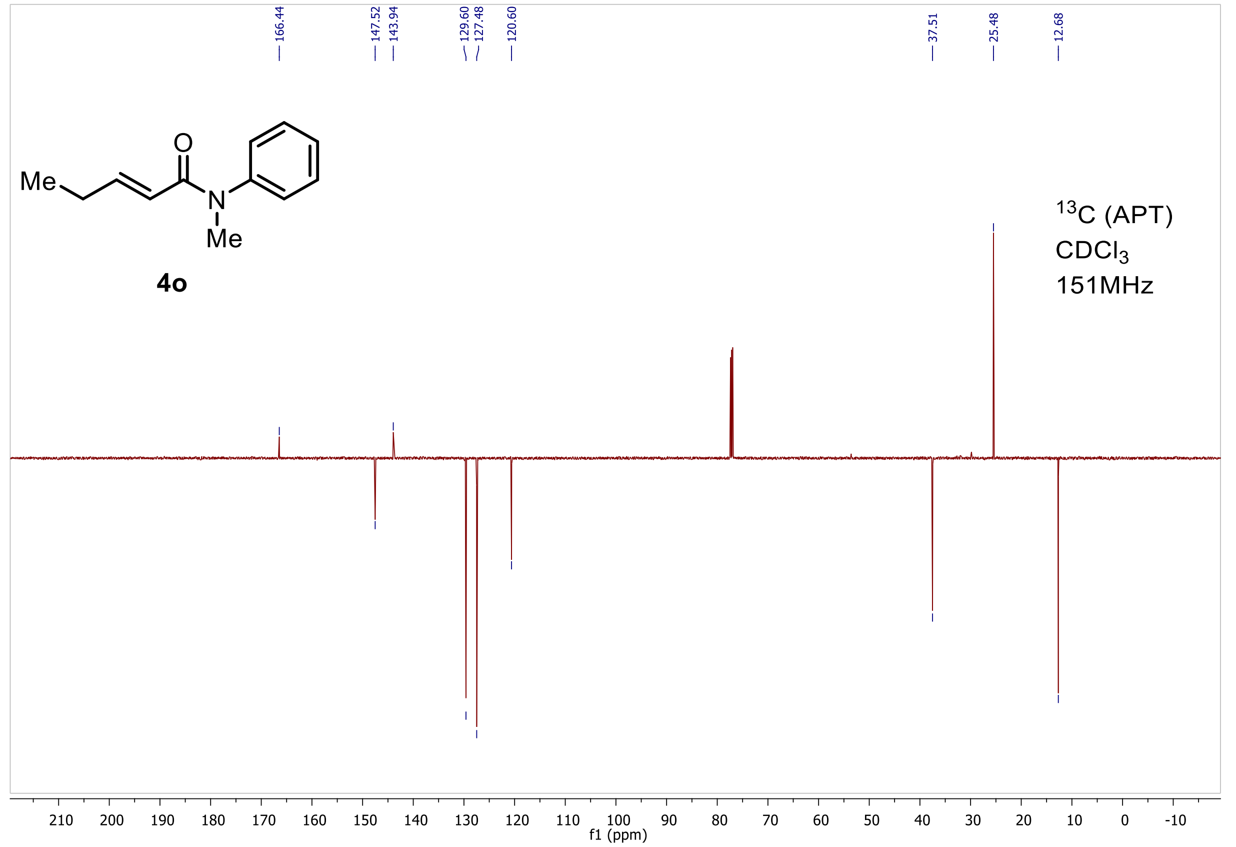
166.44
147.52
143.94
129.60
127.48
120.60
37.51
25.48
12.68
Me
O
N
Me
4o
^{13}C (APT)
$CDCl_3$
151MHz
210 200 190 180 170 160 150 140 130 120 110 100 90 80 70 60 50 40 30 20 10 0 -10
f1 (ppm)

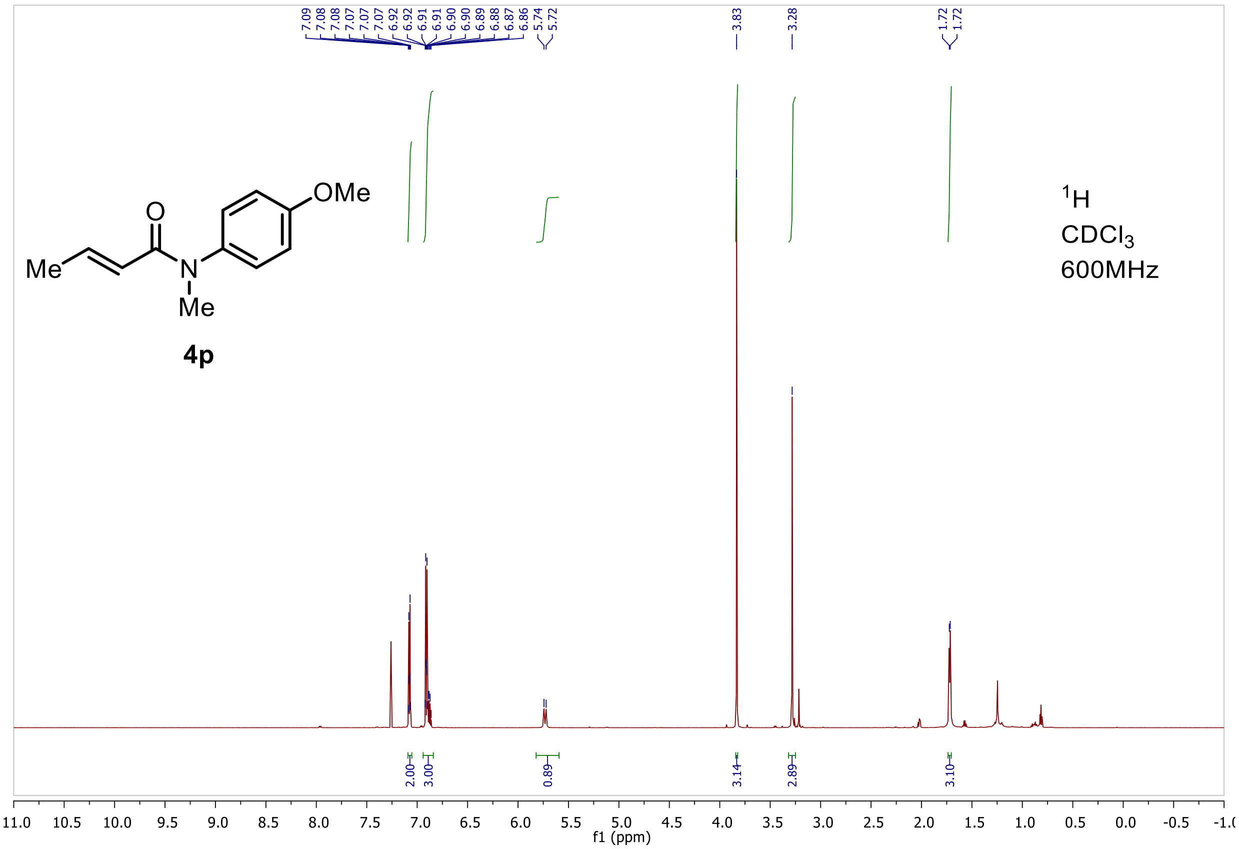
7.09
7.08
7.08
7.07
7.07
7.07
6.92
6.92
6.91
6.91
6.91
6.90
6.89
6.88
6.87
6.86
5.74
5.72
3.83
3.28
1.72
1.72
OMe
O
Me
N
Me
4p
1H
CDCl3
600MHz
2.00
3.00
0.89
3.14
2.89
3.10
11.0 10.5 10.0 9.5 9.0 8.5 8.0 7.5 7.0 6.5 6.0 5.5 5.0 4.5 4.0 3.5 3.0 2.5 2.0 1.5 1.0 0.5 0.0 -0.5
f1 (ppm)

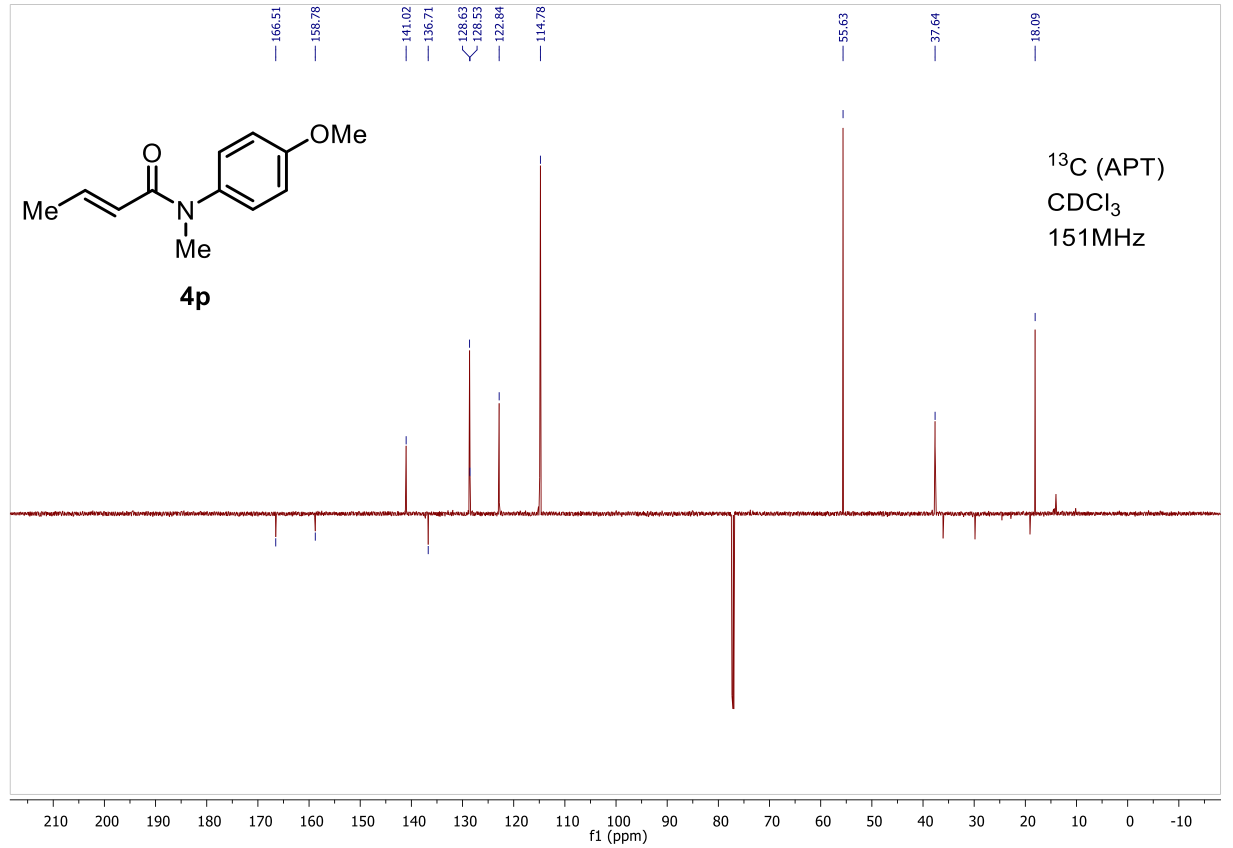
166.51
158.78
141.02
136.71
128.63
128.53
122.84
114.78
55.63
37.64
18.09
OMe
O
Me
N
Me
4p
13C (APT)
CDCl3
151MHz
210 200 190 180 170 160 150 140 130 120 110 100 90 80 70 60 50 40 30 20 10 0 -10
f1 (ppm)

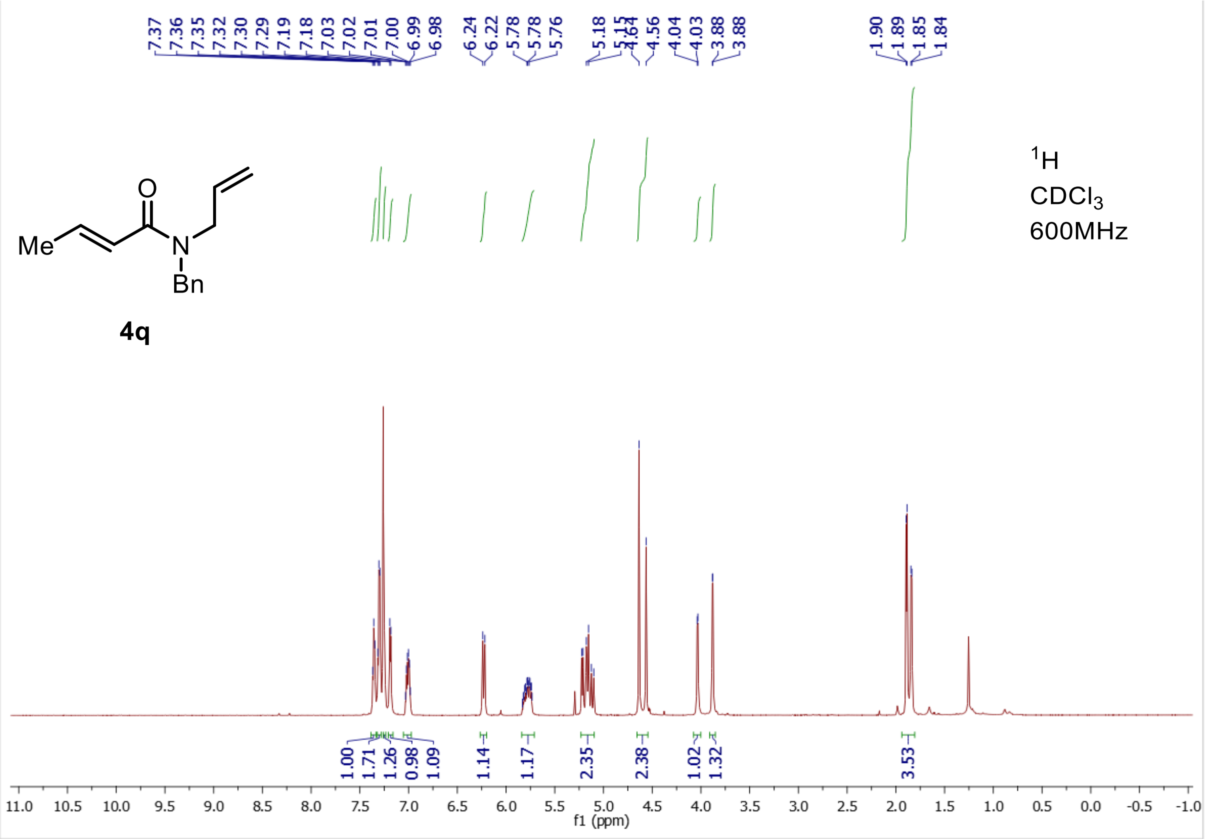
7.37 7.36 7.35 7.32 7.30 7.29 7.19 7.18 7.03 7.02 7.01 7.00 6.99 6.98
6.24 6.22 5.78 5.78 5.76
5.18 5.15 4.64 4.56 4.04 4.03 3.88 3.88
1.90 1.89 1.85 1.84
1H
CDCl3
600MHz
O
Me
N
Bn
4q
1.00 1.71 1.26 0.98 1.09 1.14 1.17 2.35 2.38 1.02 1.32 3.53
f1 (ppm)

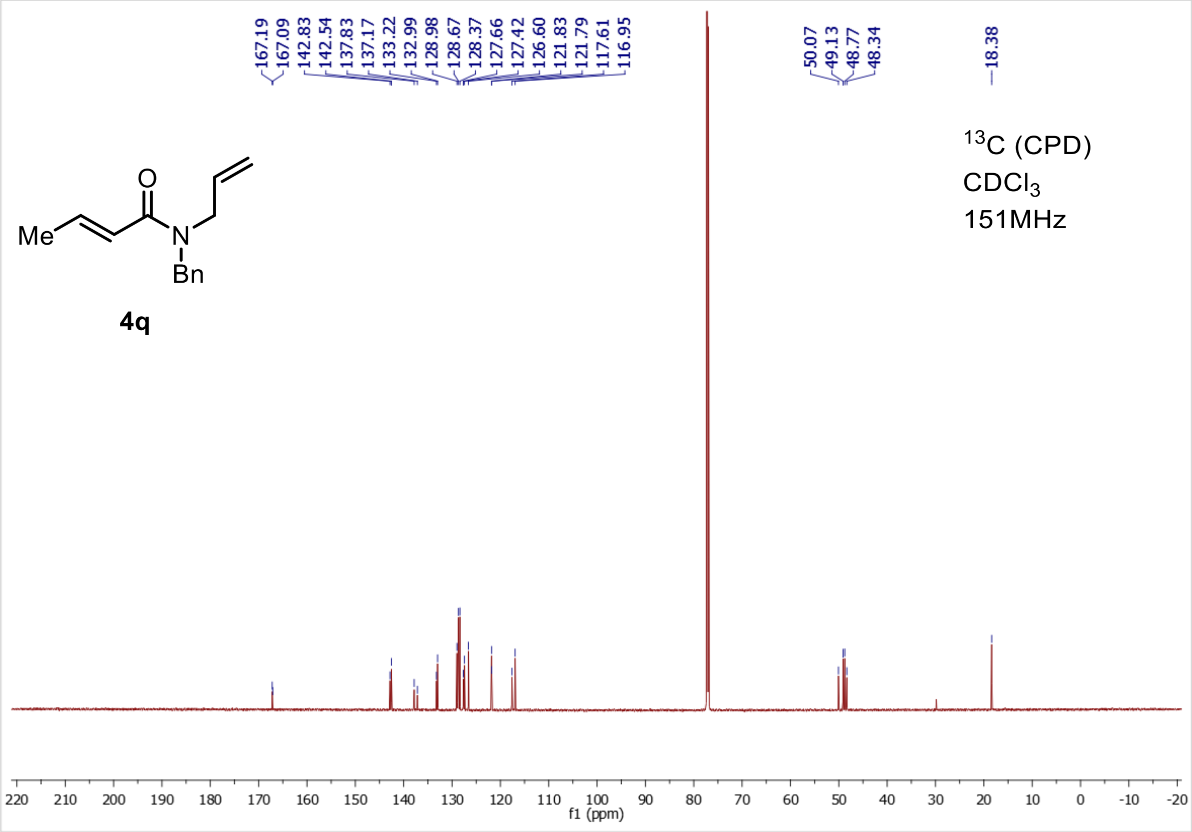
167.19 167.09 142.83 142.54 137.83 137.17 133.22 132.99 128.98 128.67 128.37 127.66 127.42 126.60 121.83 121.79 117.61 116.95
50.07 49.13 48.77 48.34
18.38
13C (CPD)
CDCl3
151MHz
O
Me
N
Bn
4q
f1 (ppm)

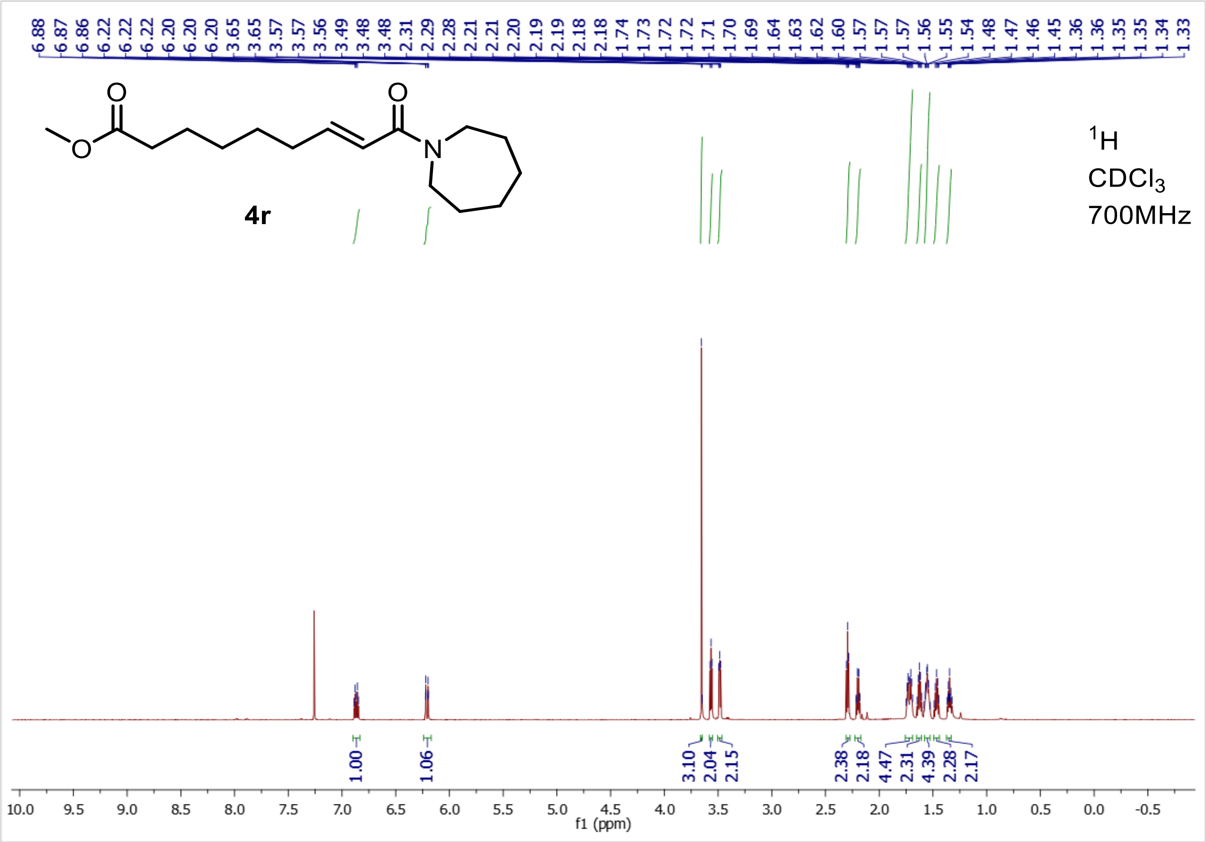

6.88
6.87
6.86
6.22
6.22
6.22
6.20
6.20
6.20
3.65
3.65
3.57
3.57
3.56
3.49
3.48
3.48
2.31
2.29
2.28
2.21
2.21
2.20
2.19
2.19
2.18
2.18
1.74
1.73
1.72
1.72
1.71
1.70
1.69
1.64
1.63
1.62
1.60
1.57
1.57
1.57
1.56
1.55
1.54
1.48
1.47
1.46
1.45
1.36
1.36
1.35
1.35
1.34
1.33
O
O
O
N
4r
^{1}H
CDCl$_3$
700MHz
1.00
1.06
3.10
2.04
2.15
2.38
2.18
4.47
2.31
4.39
2.28
2.17
10.0 9.5 9.0 8.5 8.0 7.5 7.0 6.5 6.0 5.5 5.0 4.5 4.0 3.5 3.0 2.5 2.0 1.5 1.0 0.5 0.0 -0.5
f1 (ppm)

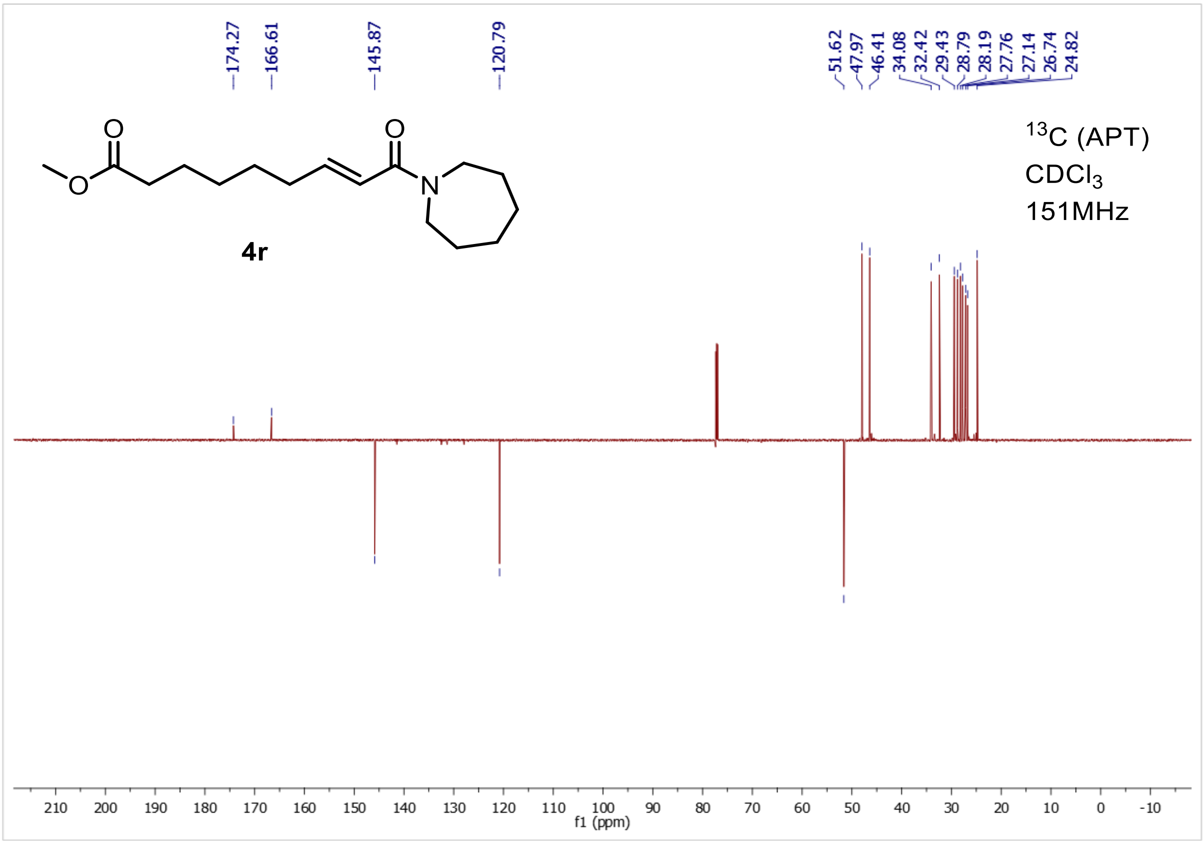

174.27
166.61
145.87
120.79
51.62
47.97
46.41
34.08
32.42
29.43
28.79
28.19
27.76
27.14
26.74
24.82
O
O
O
N
4r
^{13}C (APT)
CDCl$_3$
151MHz
210 200 190 180 170 160 150 140 130 120 110 100 90 80 70 60 50 40 30 20 10 0 -10
f1 (ppm)

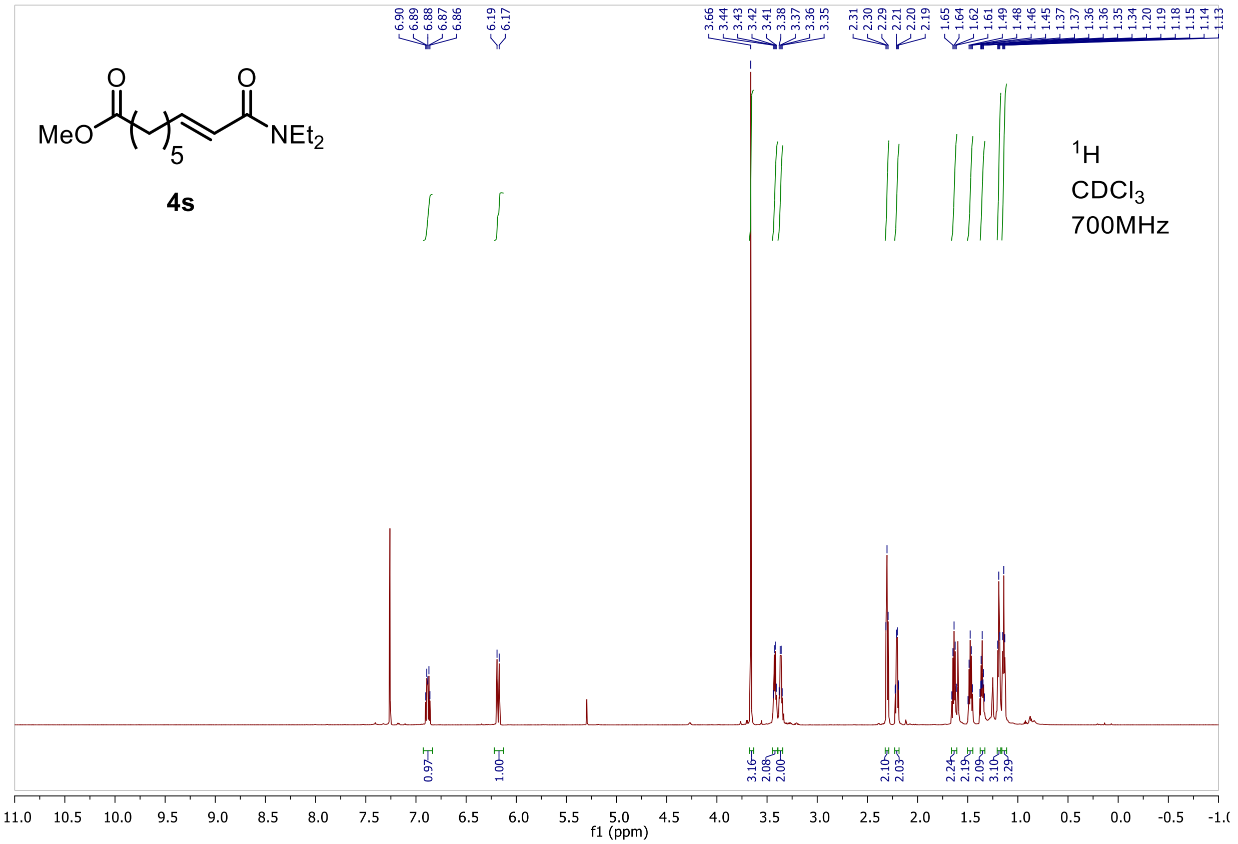
MeO
NEt2
5
4s
1H
CDCl3
700MHz
f1 (ppm)

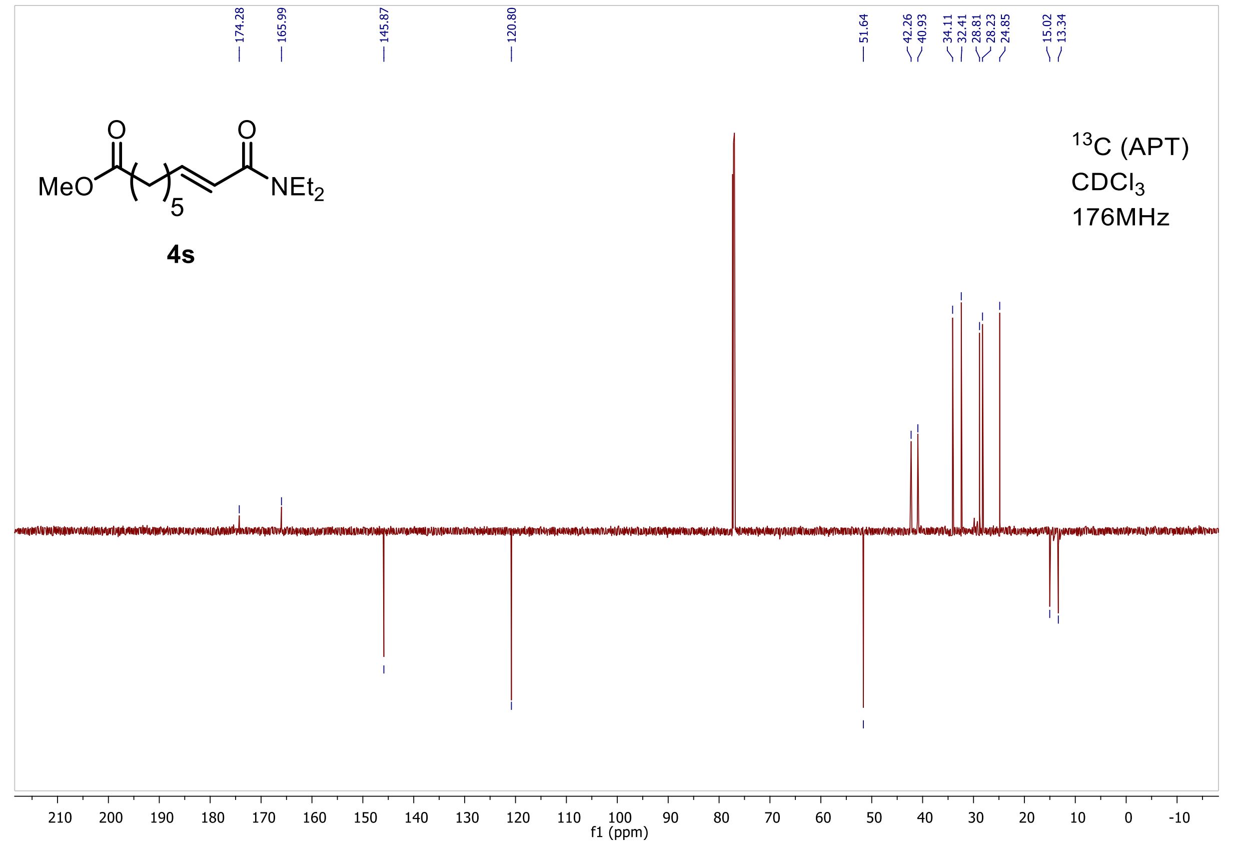
MeO
NEt2
5
4s
13C (APT)
CDCl3
176MHz
f1 (ppm)

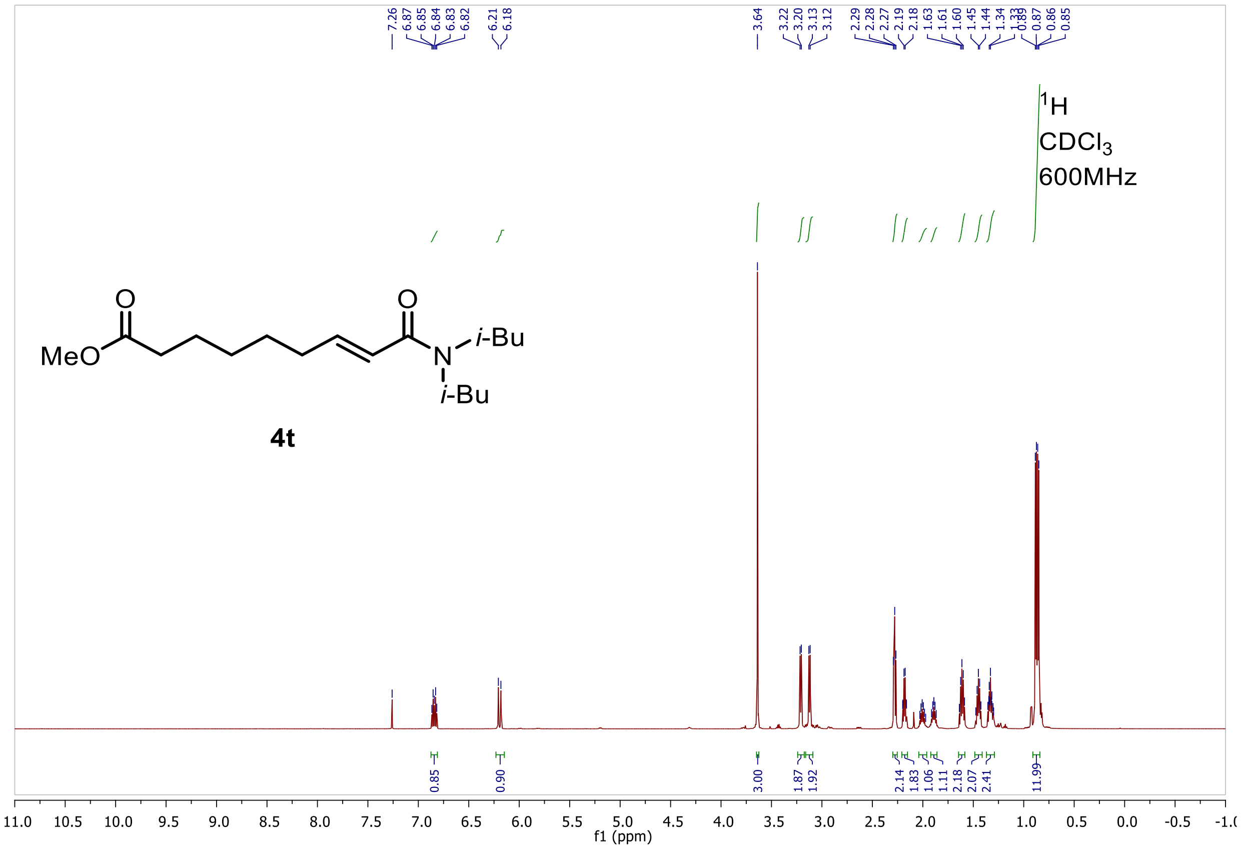
7.26 6.87 6.85 6.84 6.83 6.82 6.21 6.18 3.64 3.22 3.20 3.13 3.12 2.29 2.28 2.27 2.19 2.18 1.63 1.61 1.60 1.45 1.44 1.34 1.33 0.89 0.87 0.86 0.85
^{1}H
$CDCl_3$
600MHz
MeO
N
i-Bu
i-Bu
4t
0.85 0.90 3.00 1.87 1.92 2.14 1.83 1.06 1.11 2.18 2.07 2.41 11.99
f1 (ppm)

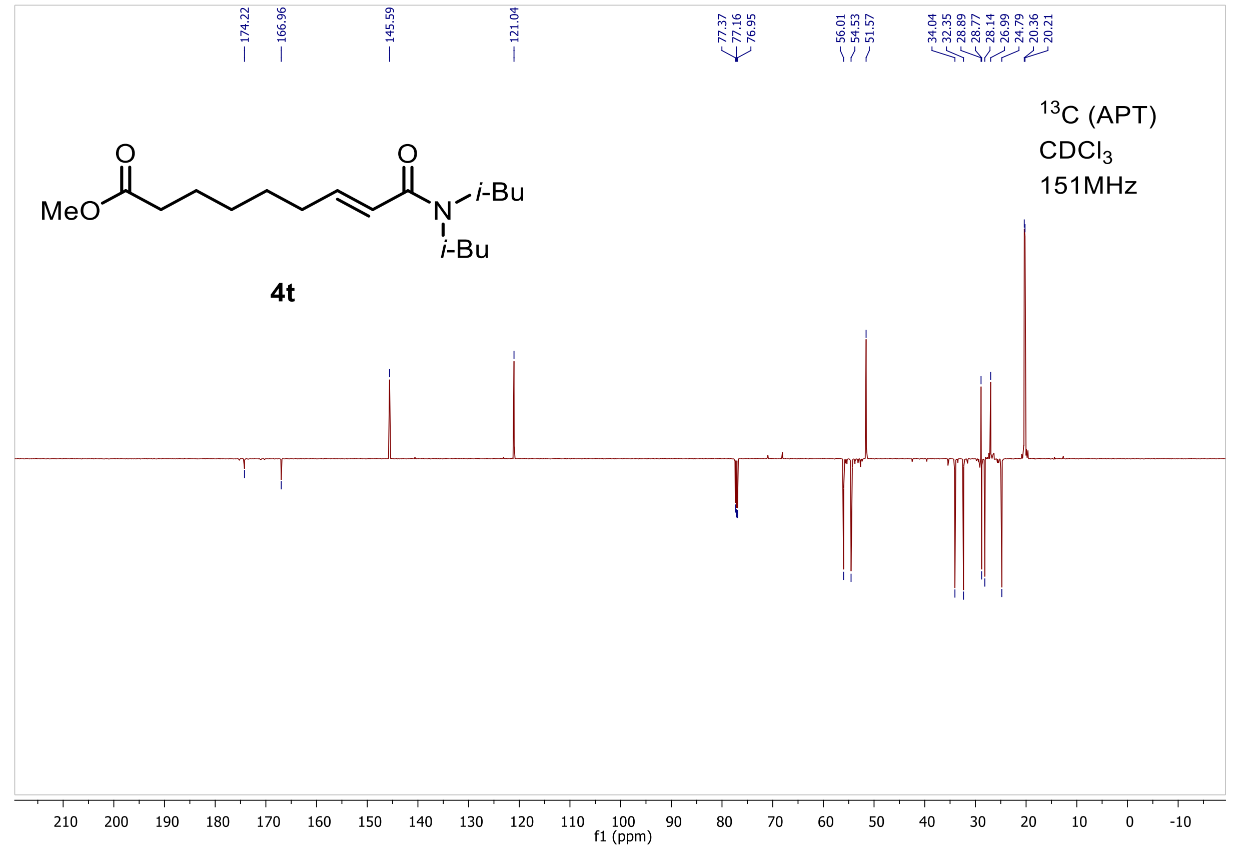
174.22 166.96 145.59 121.04 77.37 77.16 76.95 56.01 54.53 51.57 34.04 32.35 28.89 28.77 28.14 26.99 24.79 20.36 20.21
^{13}C (APT)
$CDCl_3$
151MHz
MeO
N
i-Bu
i-Bu
4t
f1 (ppm)

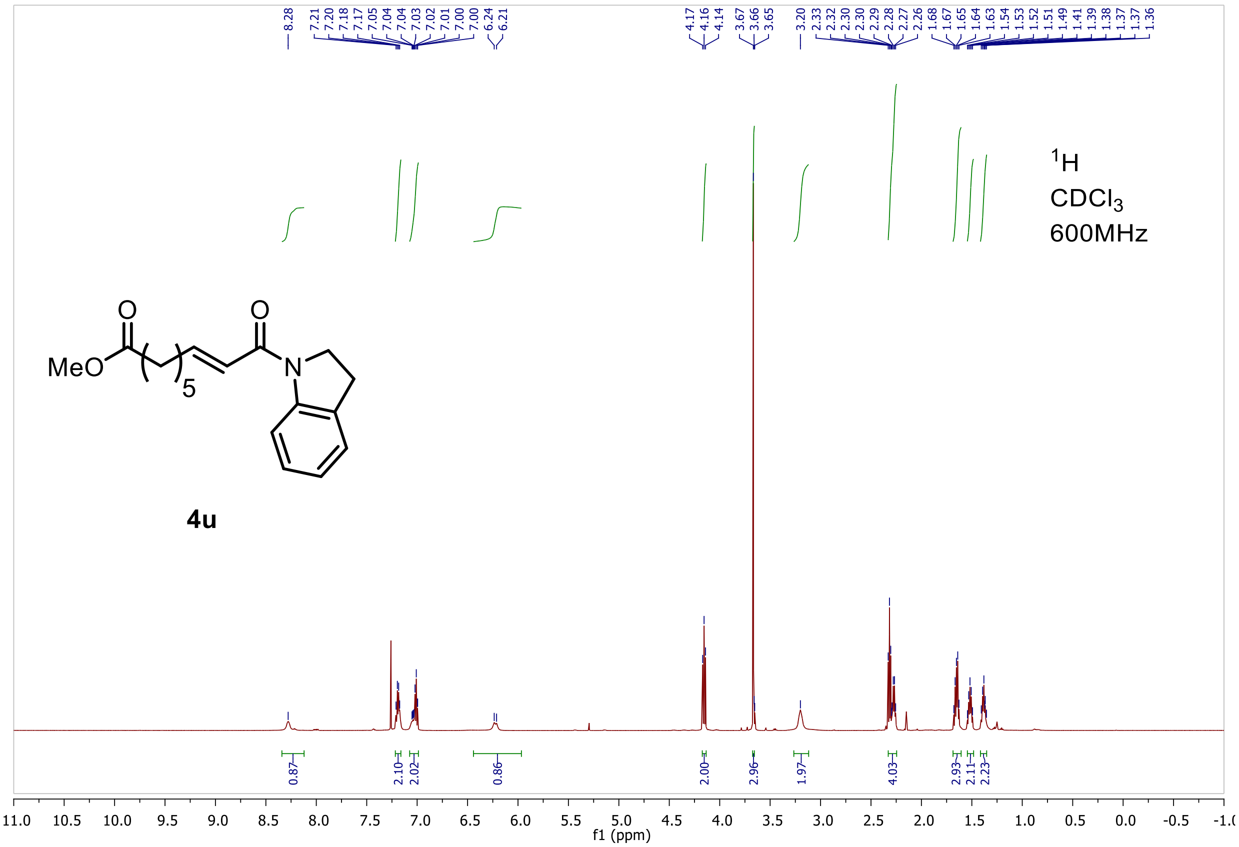

4u

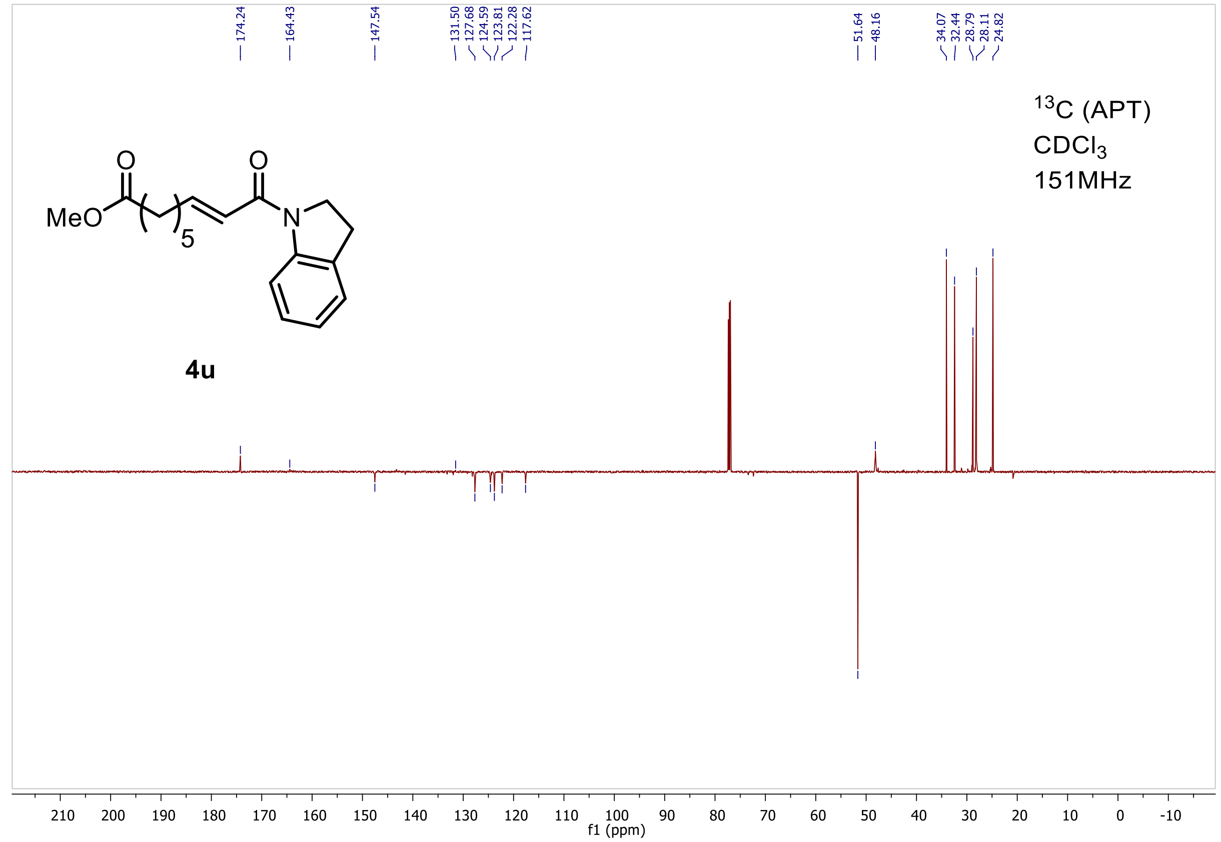

4u

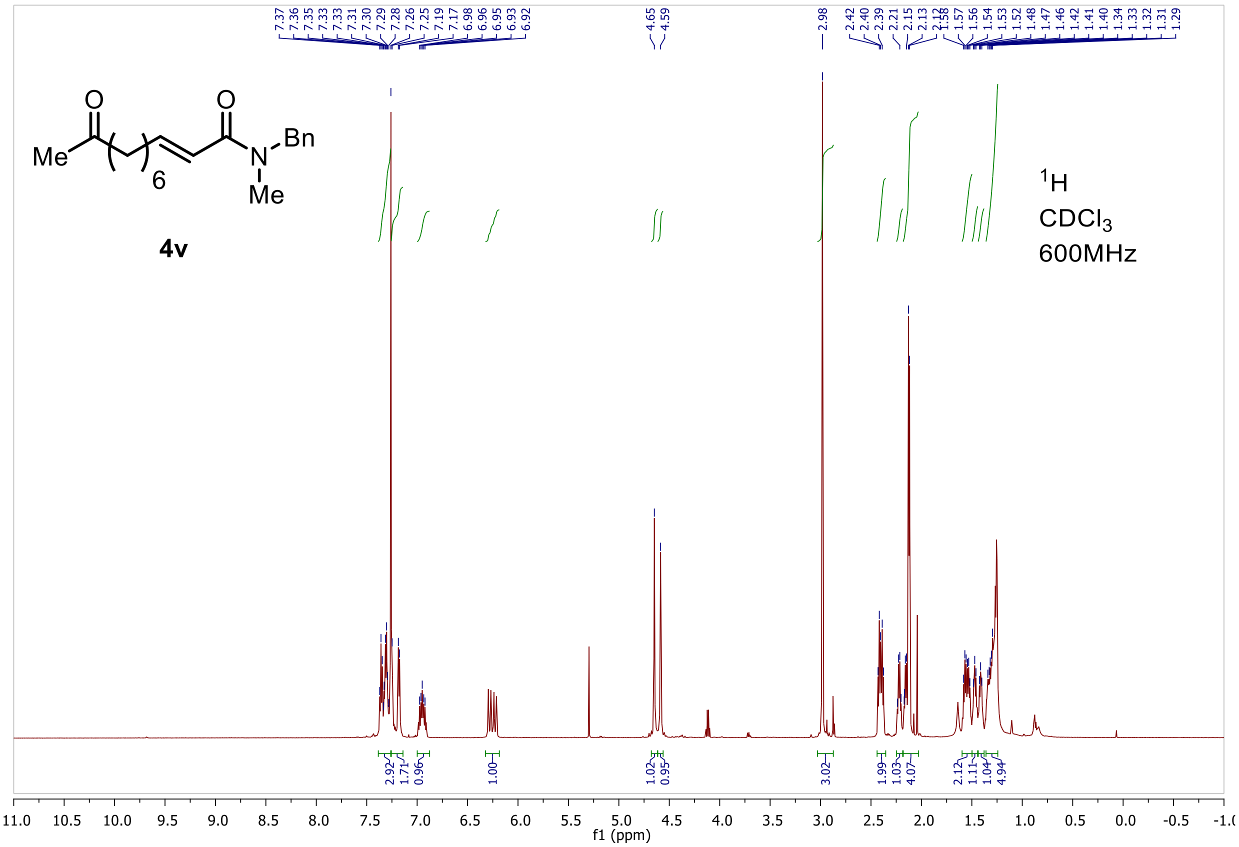

7.37
7.36
7.35
7.33
7.33
7.31
7.30
7.29
7.28
7.26
7.25
7.19
7.17
6.98
6.96
6.95
6.93
6.92
4.65
4.59
2.98
2.42
2.40
2.39
2.21
2.15
2.13
1.57
1.56
1.54
1.53
1.52
1.48
1.47
1.46
1.42
1.41
1.40
1.34
1.33
1.32
1.31
1.29
Me
O
O
N
Bn
Me
6
4v
^{1}H
CDCl$_3$
600MHz
2.92
1.71
0.96
1.00
1.02
0.95
3.02
1.99
1.03
4.07
2.12
1.11
1.04
4.94
11.0
10.5
10.0
9.5
9.0
8.5
8.0
7.5
7.0
6.5
6.0
5.5
5.0
4.5
4.0
3.5
3.0
2.5
2.0
1.5
1.0
0.5
0.0
-0.5
f1 (ppm)

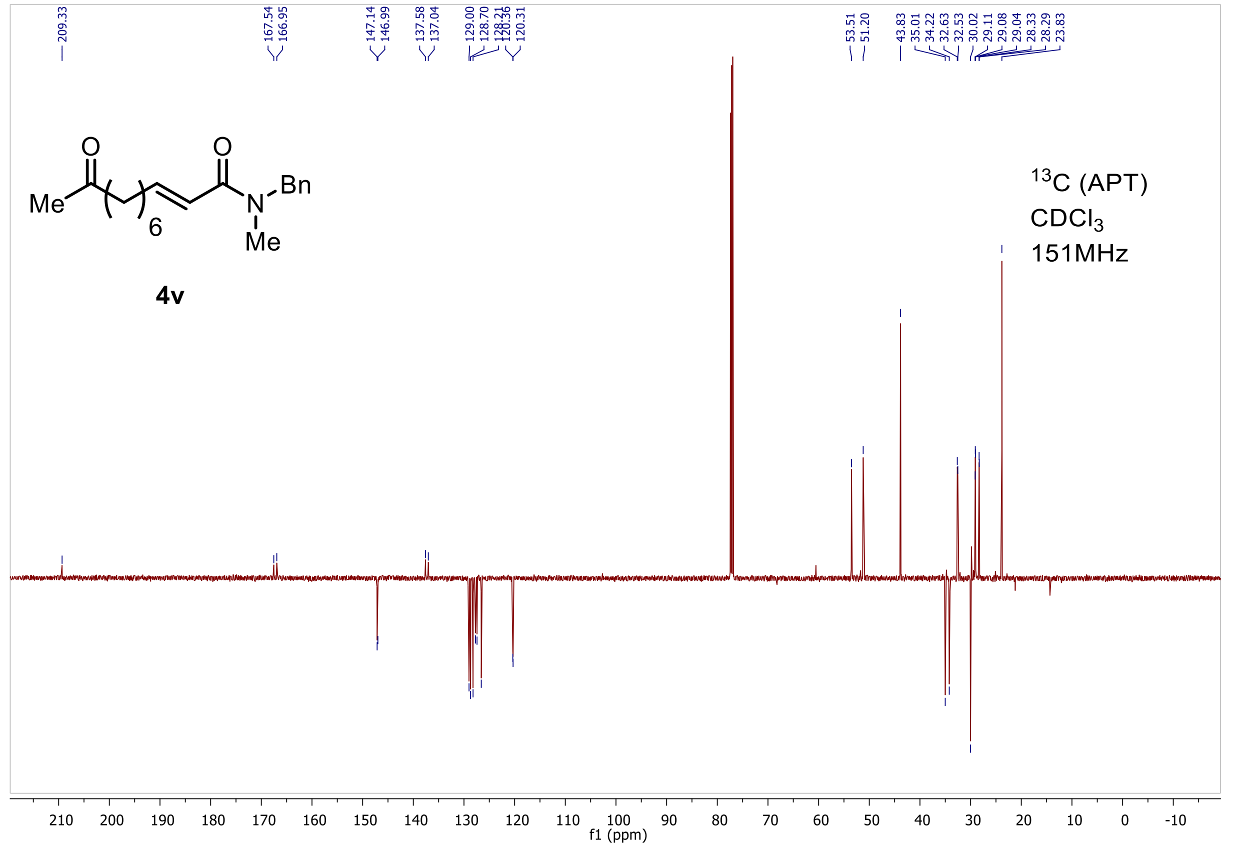

209.33
167.54
166.95
147.14
146.99
137.58
137.04
129.00
128.70
120.31
53.51
51.20
43.83
35.01
34.22
32.63
32.53
30.02
29.11
29.08
29.04
28.33
28.29
23.83
Me
O
O
N
Bn
Me
6
4v
^{13}C (APT)
CDCl$_3$
151MHz
210
200
190
180
170
160
150
140
130
120
110
100
90
80
70
60
50
40
30
20
10
0
-10
f1 (ppm)

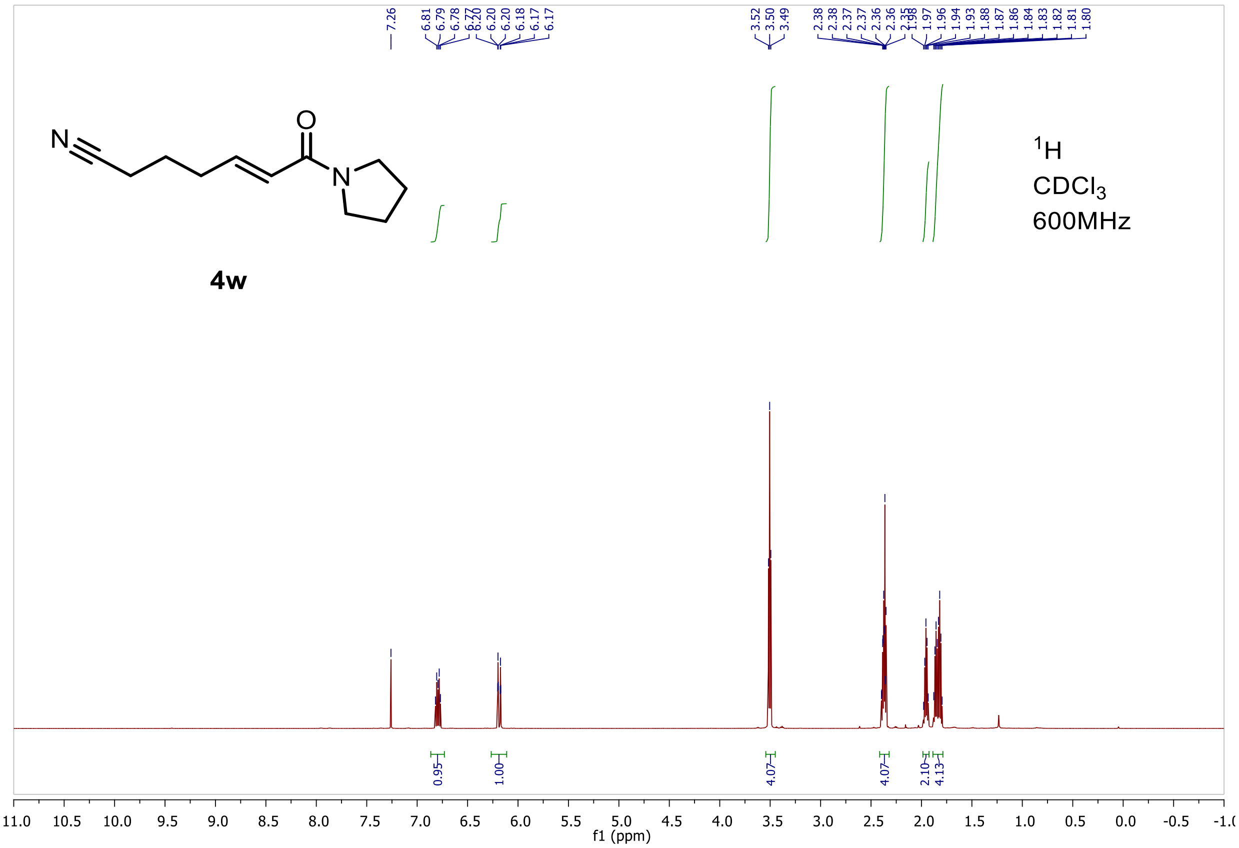
7.26
6.81
6.79
6.78
6.77
6.20
6.20
6.20
6.18
6.17
6.17
3.52
3.50
3.49
2.38
2.38
2.37
2.37
2.36
2.36
2.35
1.98
1.97
1.96
1.94
1.93
1.88
1.87
1.86
1.84
1.83
1.82
1.81
1.80
4w
^{1}H
CDCl$_3$
600MHz
0.95
1.00
4.07
4.07
2.10
4.13
f1 (ppm)

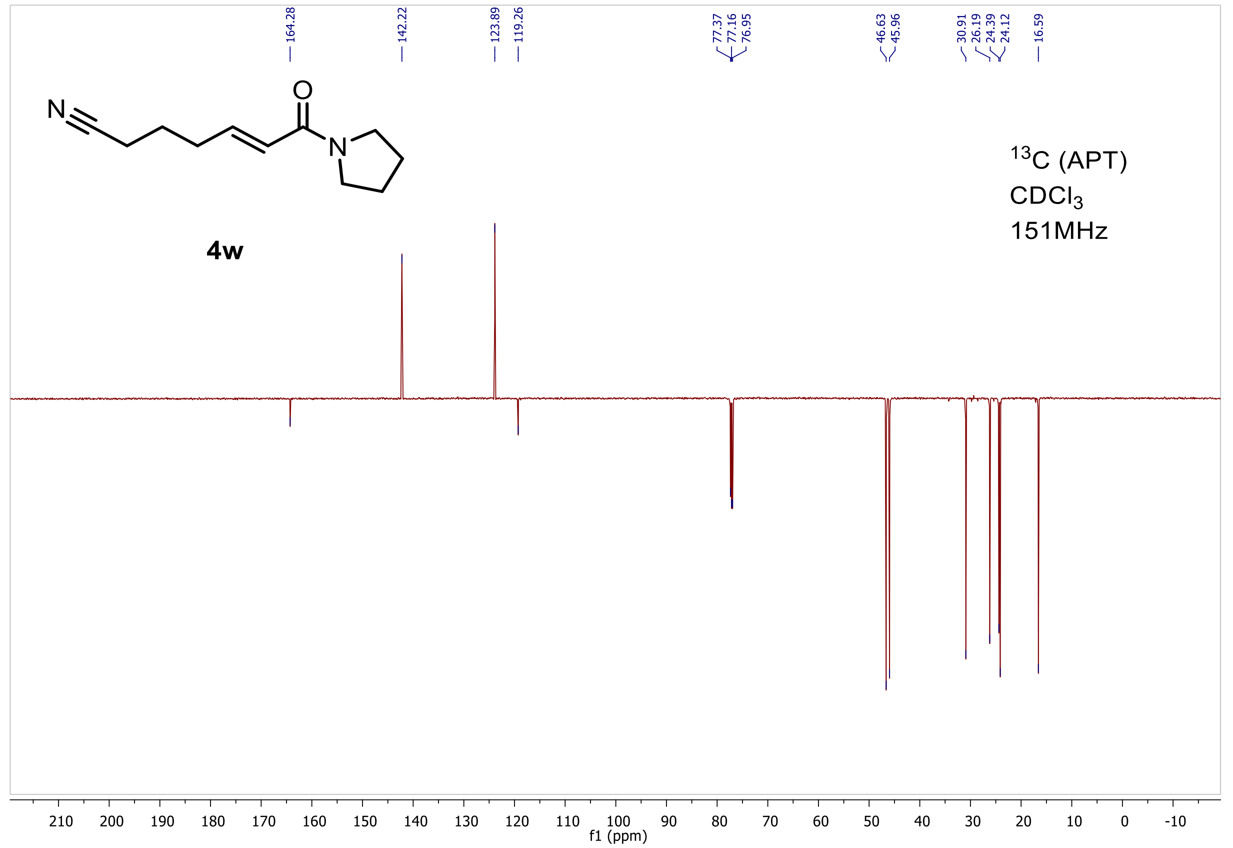
164.28
142.22
123.89
119.26
77.37
77.16
76.95
46.63
45.96
30.91
26.19
24.39
24.12
16.59
4w
^{13}C (APT)
CDCl$_3$
151MHz
f1 (ppm)

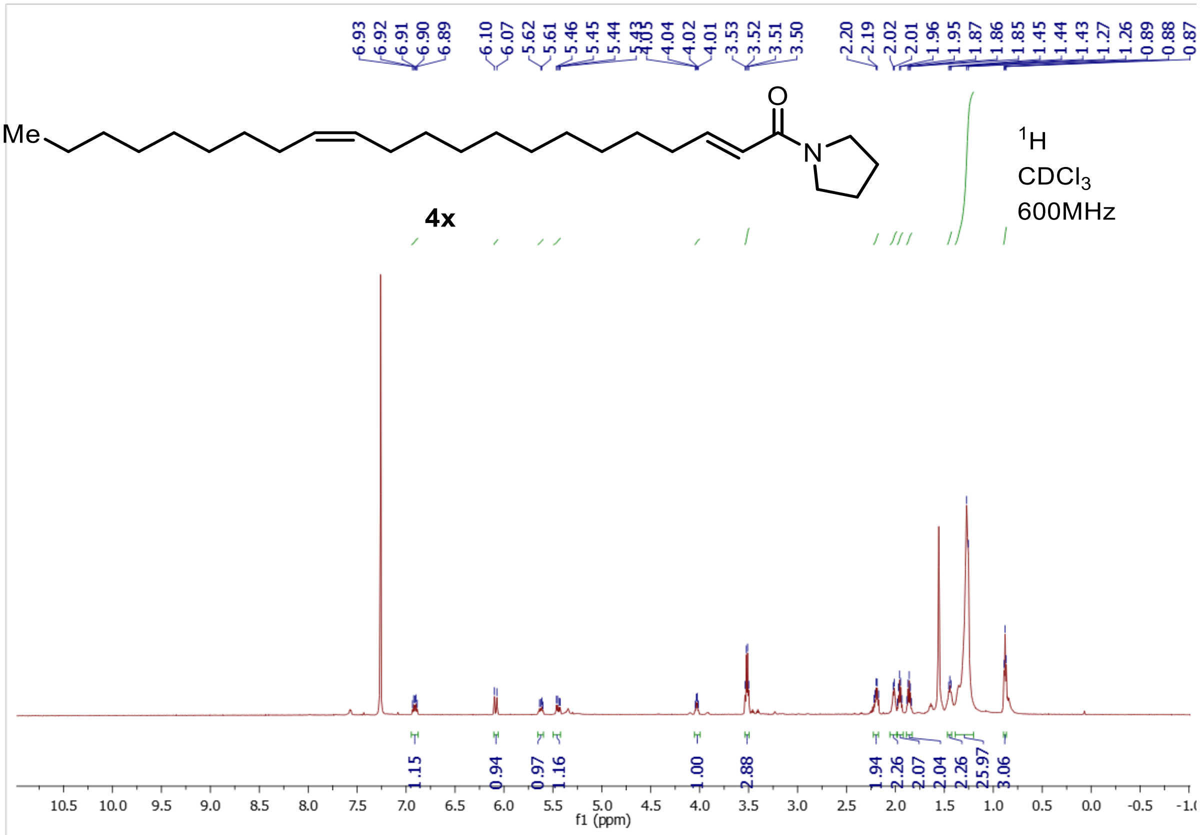
6.93
6.92
6.91
6.90
6.89
6.10
6.07
5.62
5.61
5.46
5.45
5.44
5.43
4.05
4.04
4.02
4.01
3.53
3.52
3.51
3.50
2.20
2.19
2.02
2.01
1.96
1.95
1.87
1.86
1.85
1.45
1.44
1.43
1.27
1.26
0.89
0.88
0.87
Me
O
N
4x
1H
CDCl3
600MHz
1.15
0.94
0.97
1.16
1.00
2.88
1.94
2.26
2.07
2.04
2.26
25.97
3.06
f1 (ppm)

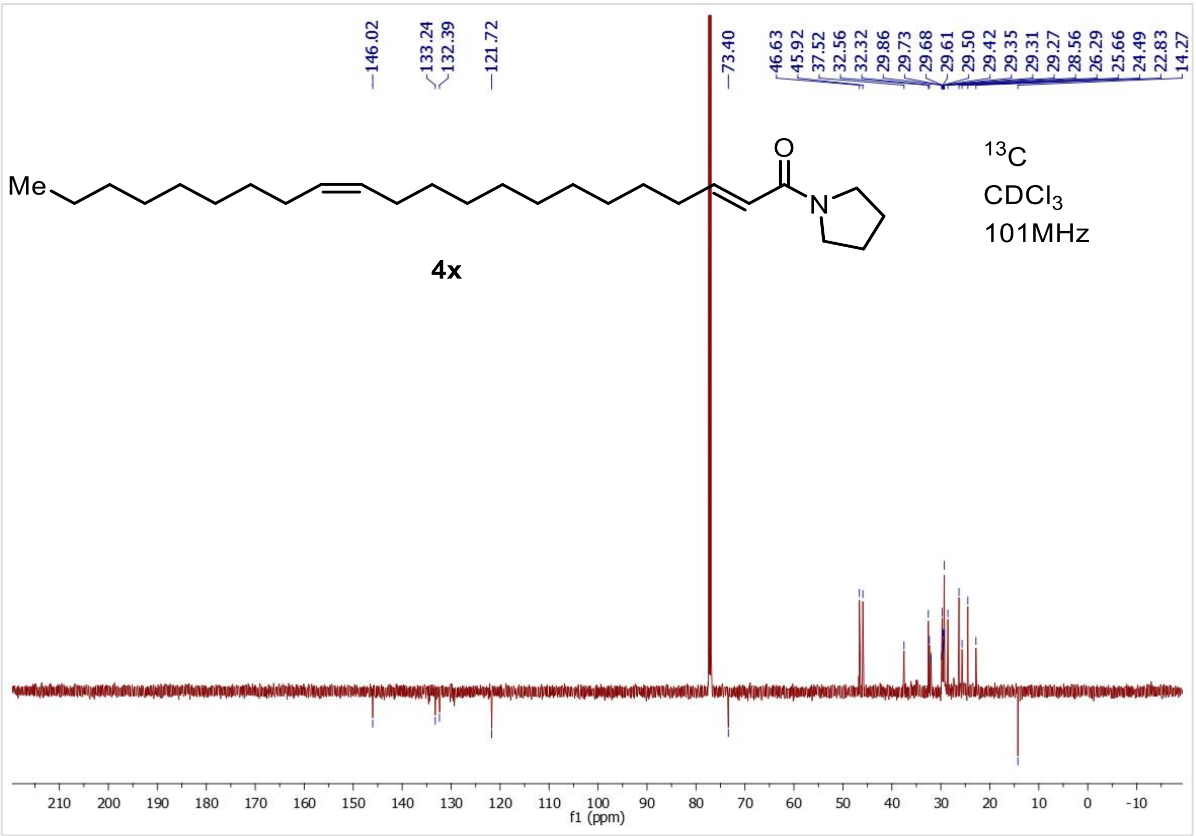
146.02
133.24
132.39
121.72
73.40
46.63
45.92
37.52
32.56
32.32
29.86
29.73
29.68
29.61
29.50
29.42
29.35
29.31
29.27
28.56
26.29
25.66
24.49
22.83
14.27
Me
O
N
4x
13C
CDCl3
101MHz
f1 (ppm)

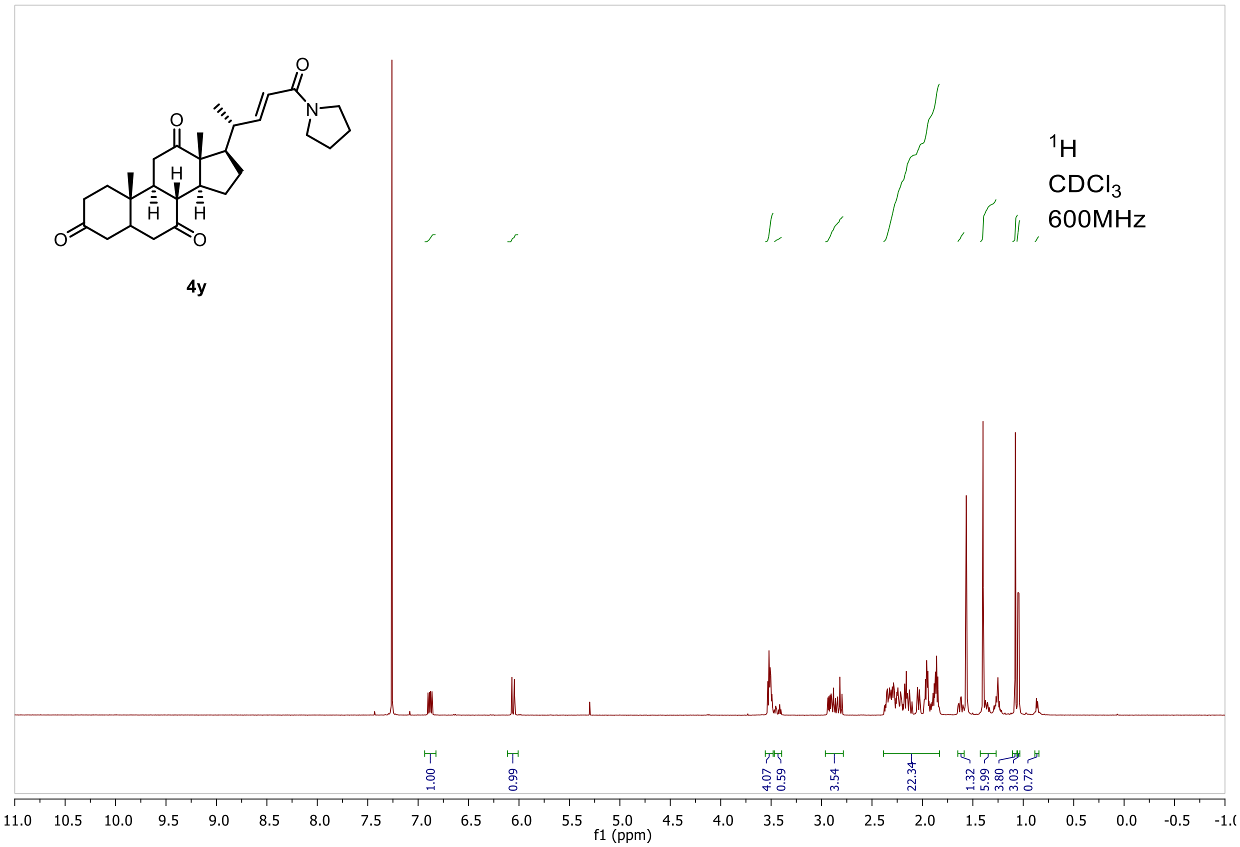
4y
^{1}H
$CDCl_3$
600MHz
f1 (ppm)

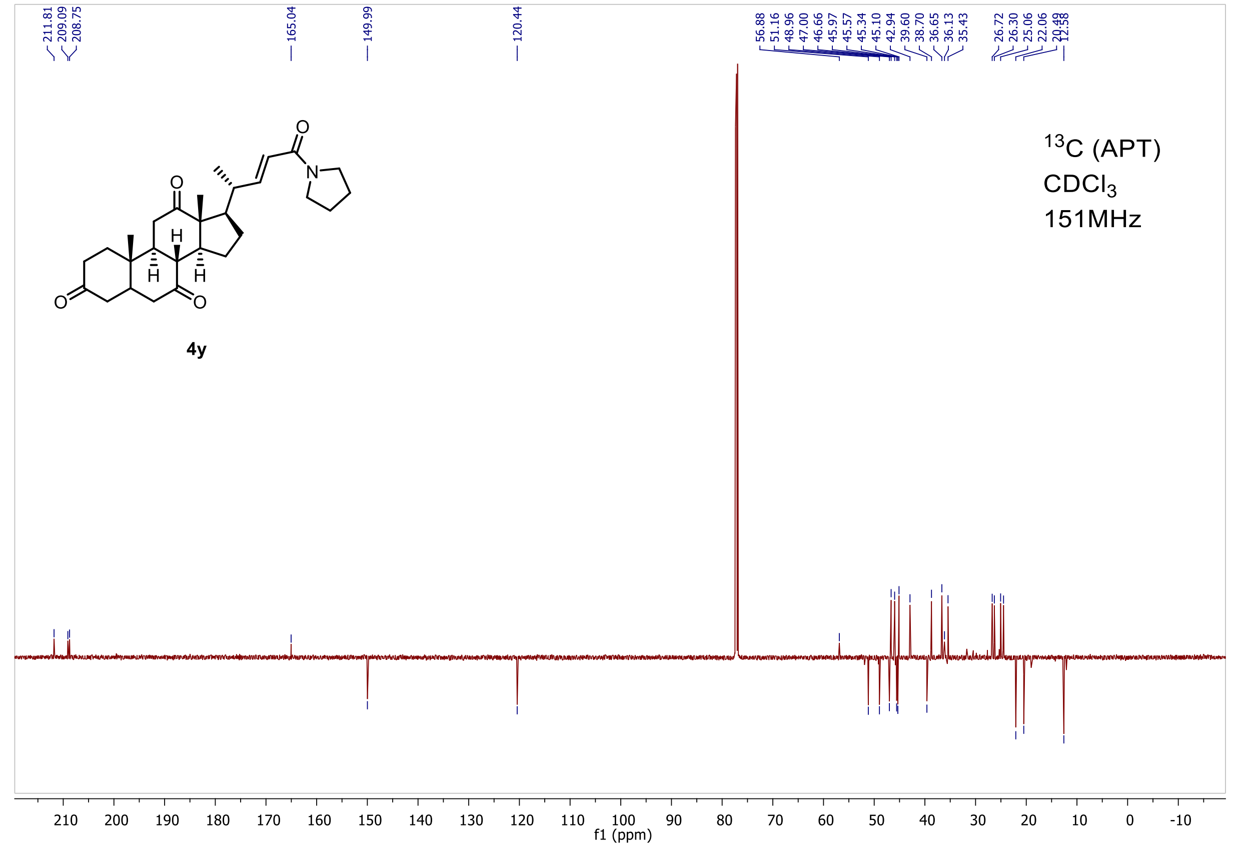
4y
^{13}C (APT)
$CDCl_3$
151MHz
f1 (ppm)

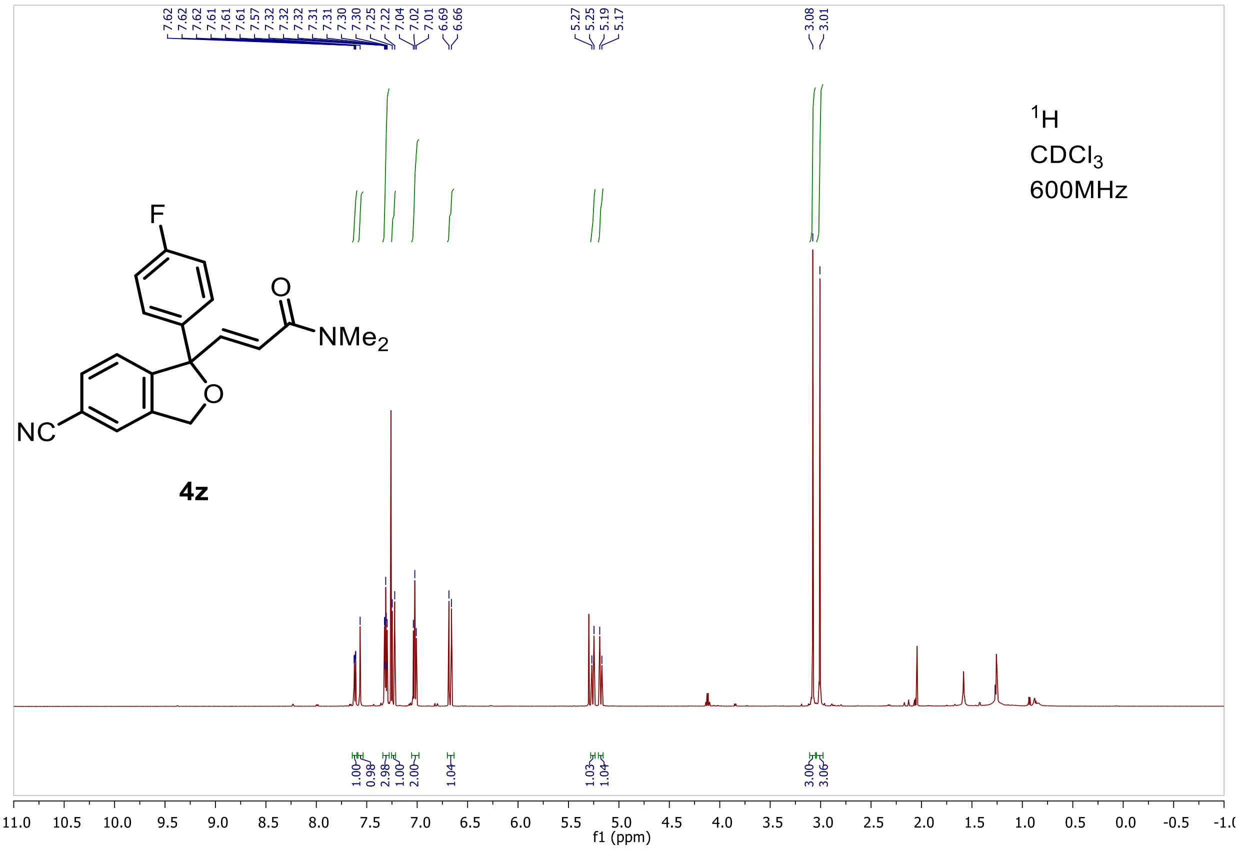
7.62 7.62 7.62 7.61 7.61 7.61 7.57 7.32 7.32 7.32 7.31 7.31 7.30 7.30 7.25 7.22 7.04 7.02 7.01 6.69 6.66
5.27 5.25 5.19 5.17
3.08 3.01
^{1}H
$CDCl_3$
600MHz
F
O
NMe_2
O
NC
4z
1.00 0.98 2.98 1.00 2.00 1.04 1.03 1.04 3.00 3.06
11.0 10.5 10.0 9.5 9.0 8.5 8.0 7.5 7.0 6.5 6.0 5.5 5.0 4.5 4.0 3.5 3.0 2.5 2.0 1.5 1.0 0.5 0.0 -0.5 -1.0
f1 (ppm)

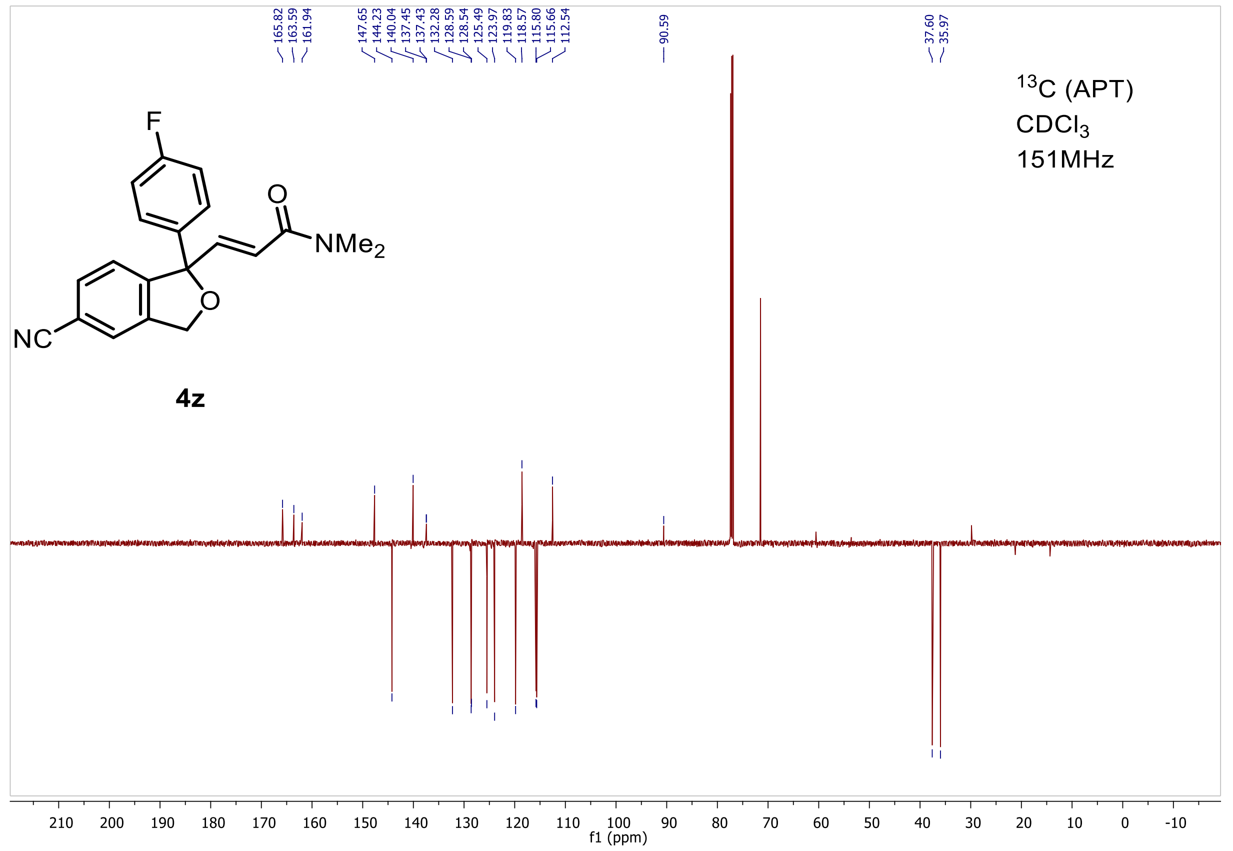
165.82 163.59 161.94 147.65 144.23 140.04 137.45 137.43 132.28 128.59 128.54 125.49 123.97 119.83 118.57 115.80 115.66 112.54 90.59 37.60 35.97
^{13}C (APT)
$CDCl_3$
151MHz
F
O
NMe_2
O
NC
4z
210 200 190 180 170 160 150 140 130 120 110 100 90 80 70 60 50 40 30 20 10 0 -10
f1 (ppm)

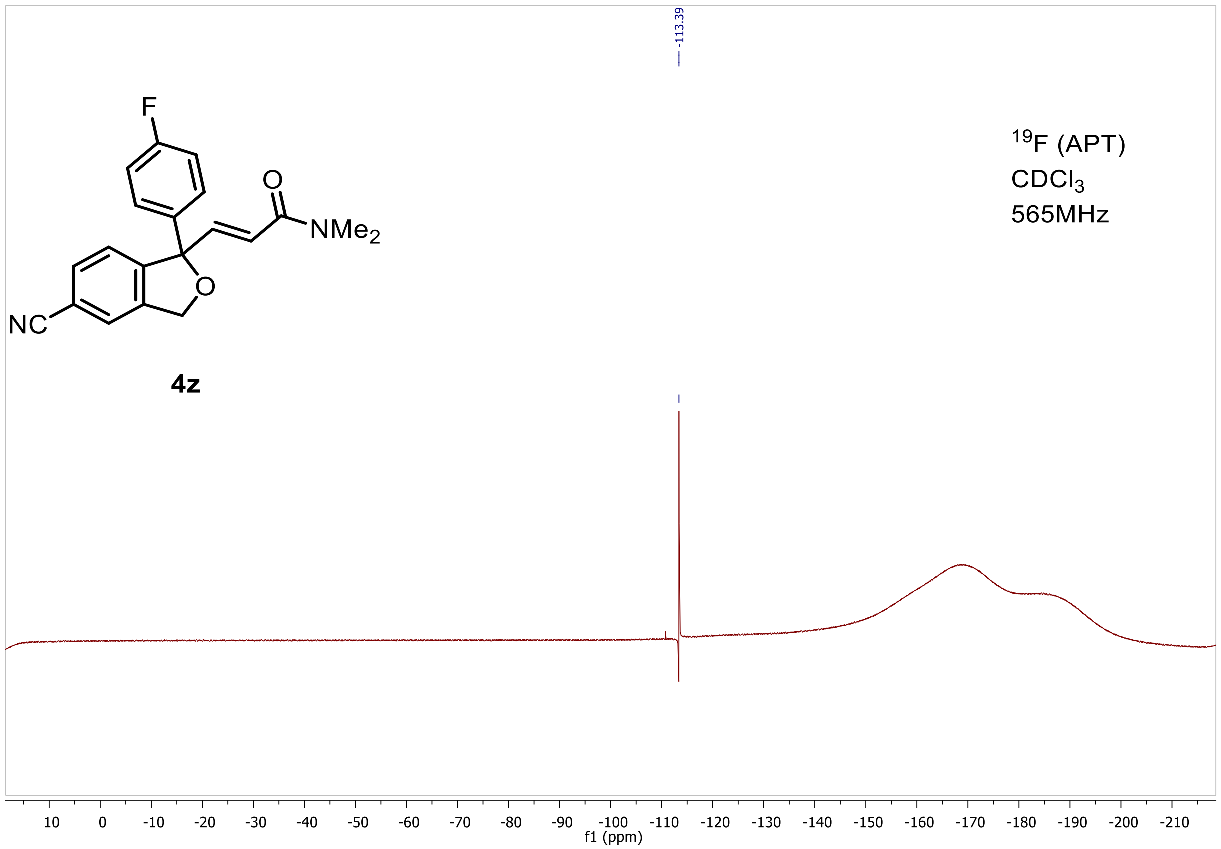
-113.39
F
O
NMe$_2$
O
NC
4z
^{19}F (APT)
CDCl$_3$
565MHz
10
0
-10
-20
-30
-40
-50
-60
-70
-80
-90
-100
-110
-120
-130
-140
-150
-160
-170
-180
-190
-200
-210
f1 (ppm)

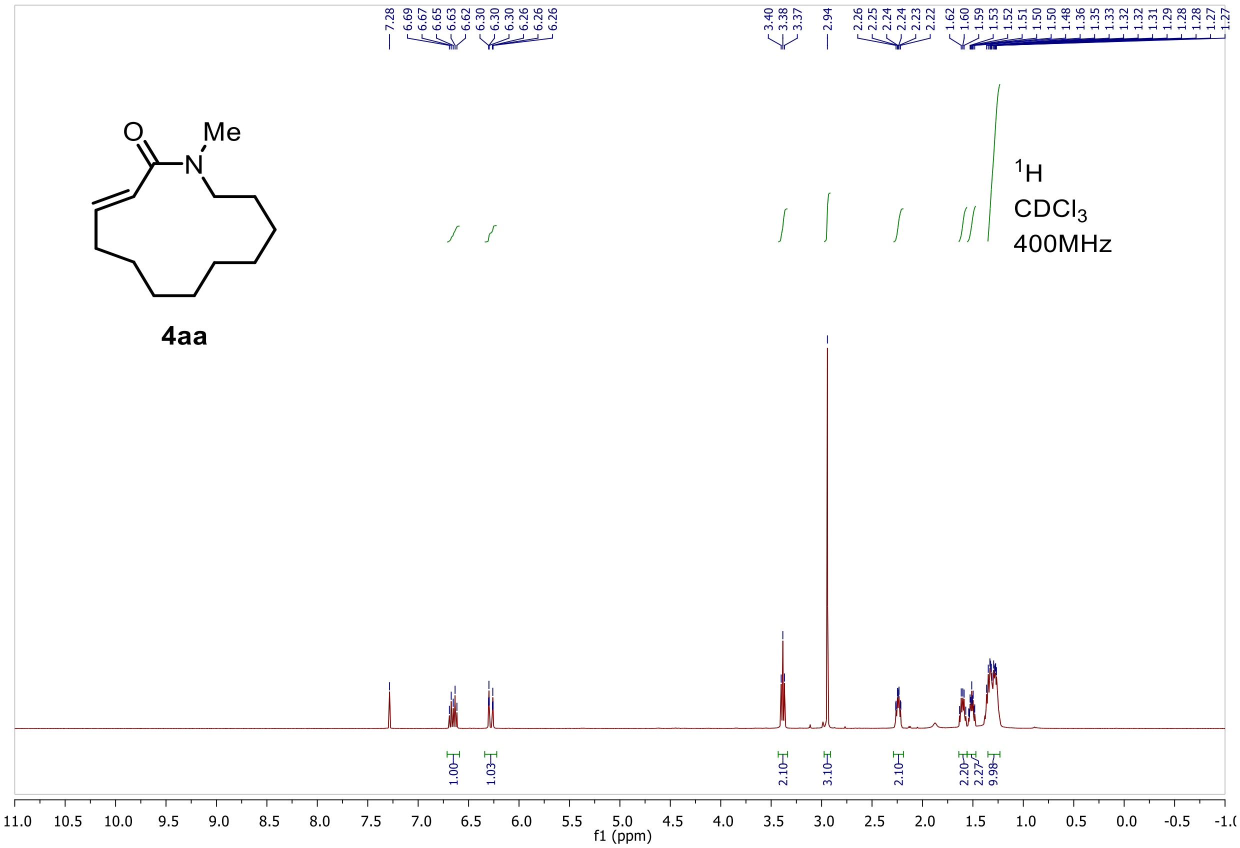

7.28
6.69
6.67
6.65
6.63
6.62
6.30
6.30
6.30
6.26
6.26
6.26
3.40
3.38
3.37
2.94
2.26
2.25
2.24
2.24
2.23
2.22
1.62
1.60
1.59
1.53
1.52
1.51
1.50
1.50
1.48
1.36
1.35
1.33
1.32
1.32
1.31
1.29
1.28
1.28
1.27
1.27
O
Me
N
4aa
^{1}H
$CDCl_3$
400MHz
1.00
1.03
2.10
3.10
2.10
2.20
2.27
9.98
11.0 10.5 10.0 9.5 9.0 8.5 8.0 7.5 7.0 6.5 6.0 5.5 5.0 4.5 4.0 3.5 3.0 2.5 2.0 1.5 1.0 0.5 0.0 -0.5
f1 (ppm)

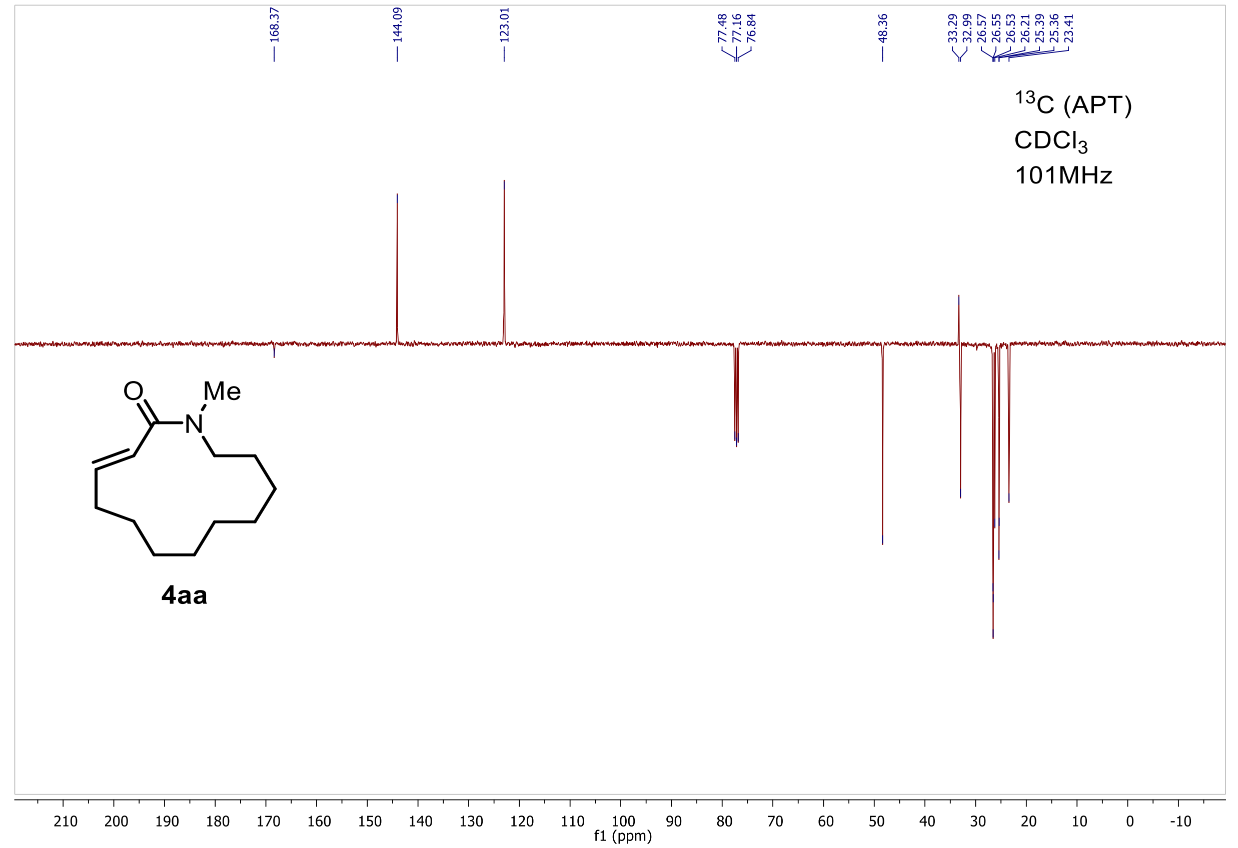

168.37
144.09
123.01
77.48
77.16
76.84
48.36
33.29
32.99
26.57
26.55
26.53
26.21
25.39
25.36
23.41
^{13}C (APT)
$CDCl_3$
101MHz
O
Me
N
4aa
210 200 190 180 170 160 150 140 130 120 110 100 90 80 70 60 50 40 30 20 10 0 -10
f1 (ppm)

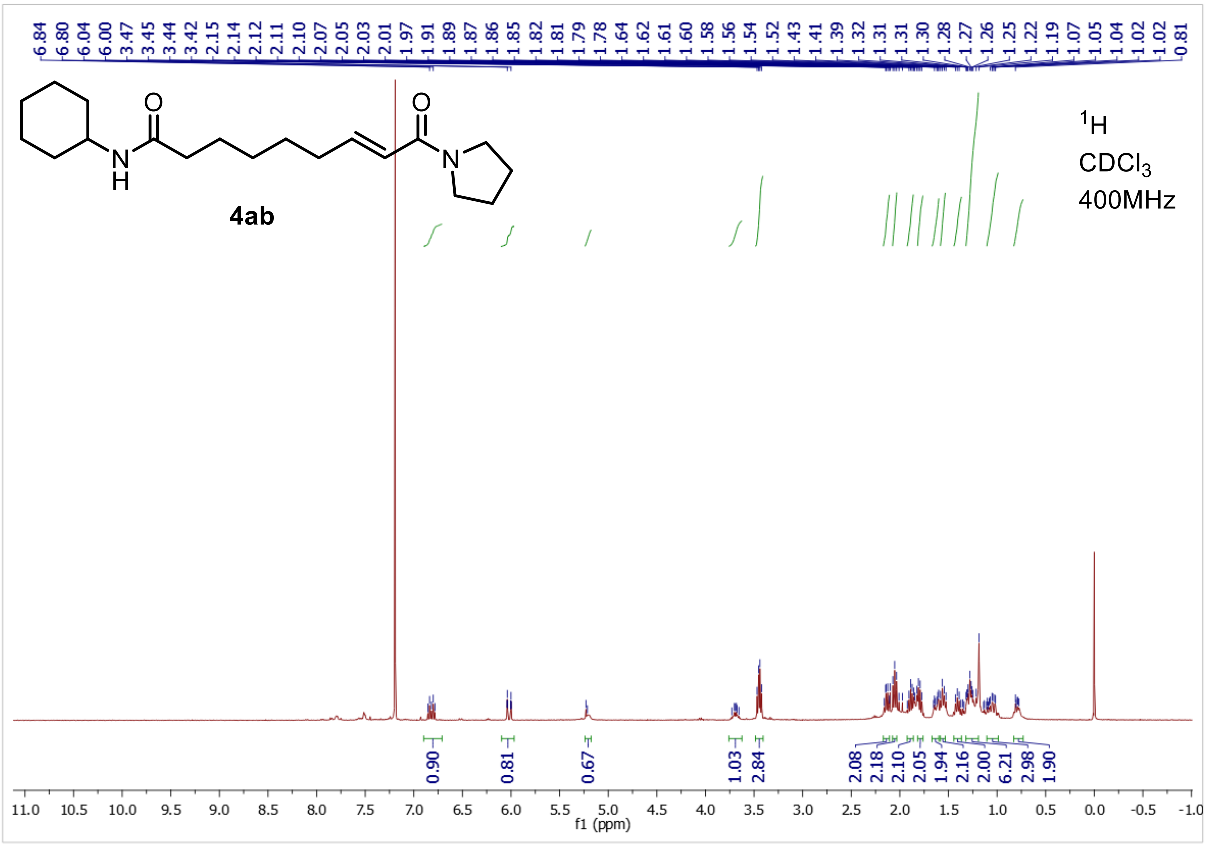
6.84 6.80 6.04 6.00 3.47 3.45 3.44 3.42 2.15 2.14 2.12 2.11 2.10 2.07 2.05 2.03 2.01 1.97 1.91 1.89 1.87 1.86 1.85 1.82 1.81 1.79 1.78 1.64 1.62 1.61 1.60 1.58 1.56 1.54 1.52 1.43 1.41 1.39 1.32 1.31 1.31 1.30 1.28 1.27 1.26 1.25 1.22 1.19 1.07 1.05 1.04 1.02 1.02 0.81
O
N
H
O
N
4ab
^{1}H
CDCl$_3$
400MHz
0.90
0.81
0.67
1.03
2.84
2.08
2.18
2.10
2.05
1.94
2.16
2.00
6.21
2.98
1.90
11.0 10.5 10.0 9.5 9.0 8.5 8.0 7.5 7.0 6.5 6.0 5.5 5.0 4.5 4.0 3.5 3.0 2.5 2.0 1.5 1.0 0.5 0.0 -0.5 -1.0
f1 (ppm)

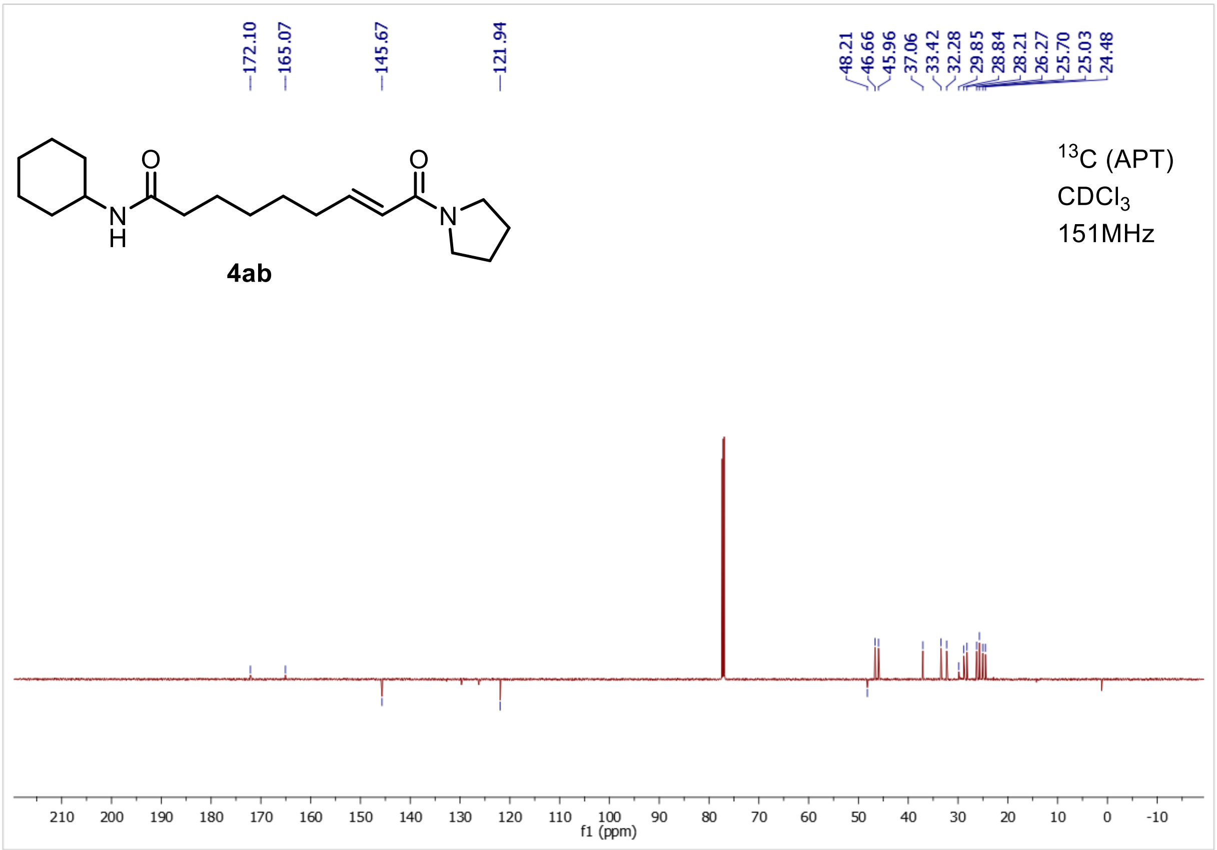
172.10
165.07
145.67
121.94
48.21 46.66 45.96 37.06 33.42 32.28 29.85 28.84 28.21 26.27 25.70 25.03 24.48
O
N
H
O
N
4ab
^{13}C (APT)
CDCl$_3$
151MHz
210 200 190 180 170 160 150 140 130 120 110 100 90 80 70 60 50 40 30 20 10 0 -10
f1 (ppm)

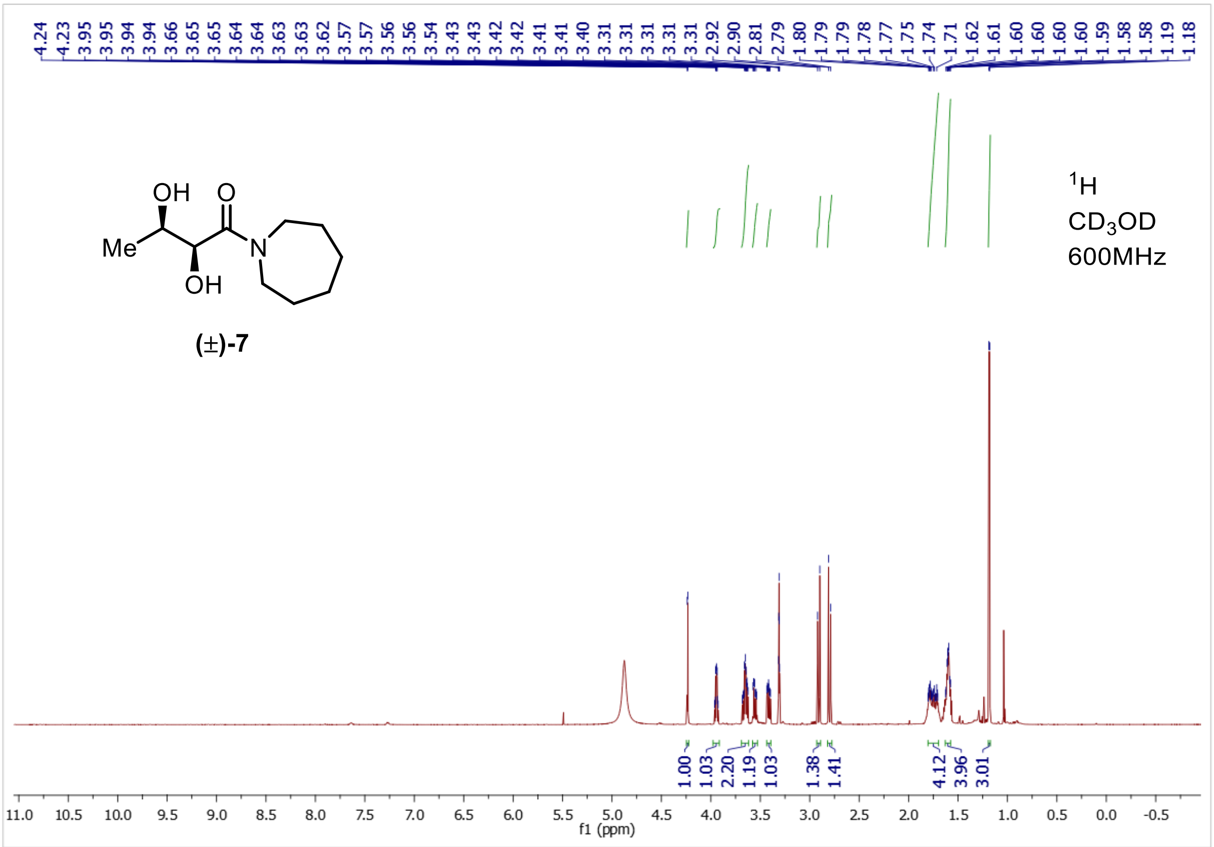
4.24 4.23 3.95 3.95 3.94 3.94 3.66 3.65 3.65 3.64 3.64 3.63 3.63 3.62 3.57 3.57 3.56 3.56 3.54 3.43 3.43 3.42 3.42 3.41 3.41 3.40 3.31 3.31 3.31 3.31 3.31 3.31 2.92 2.90 2.81 2.79 1.80 1.79 1.79 1.78 1.77 1.75 1.74 1.71 1.62 1.61 1.60 1.60 1.60 1.60 1.59 1.58 1.58 1.19 1.18
OH
O
Me
N
OH
(±)-7
1H
CD3OD
600MHz
1.00 1.03 2.20 1.19 1.03 1.38 1.41 4.12 3.96 3.01
11.0 10.5 10.0 9.5 9.0 8.5 8.0 7.5 7.0 6.5 6.0 5.5 5.0 4.5 4.0 3.5 3.0 2.5 2.0 1.5 1.0 0.5 0.0 -0.5
f1 (ppm)

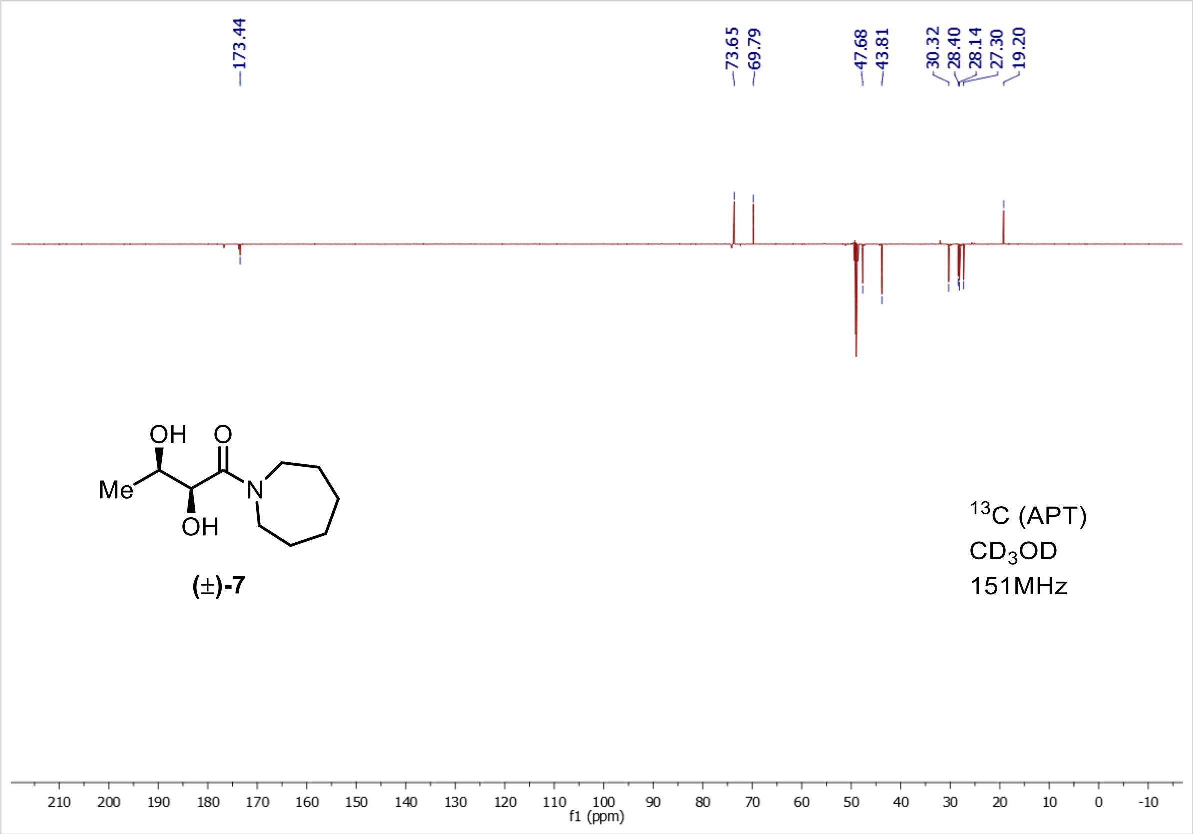
173.44
73.65 69.79
47.68 43.81
30.32 28.40 28.14 27.30 19.20
OH
O
Me
N
OH
(±)-7
13C (APT)
CD3OD
151MHz
210 200 190 180 170 160 150 140 130 120 110 100 90 80 70 60 50 40 30 20 10 0 -10
f1 (ppm)

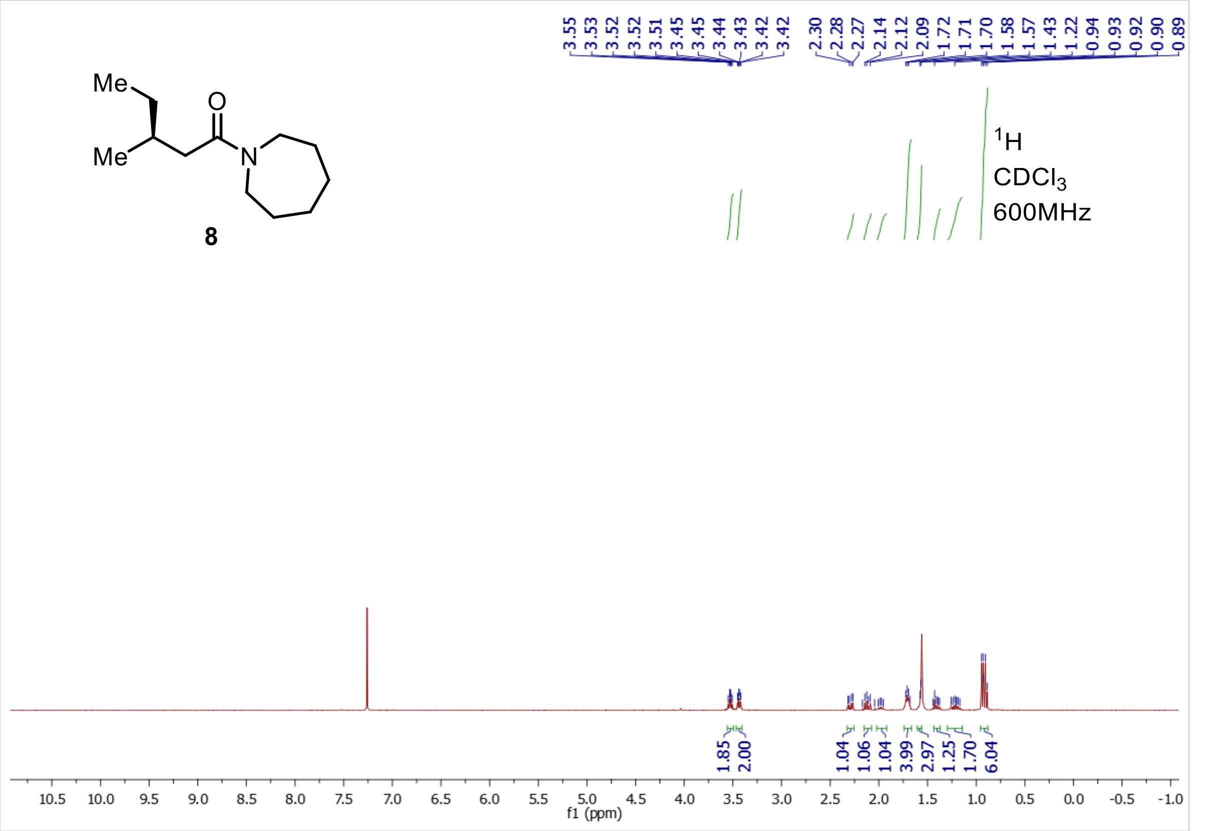

Me
Me
O
N
8
3.55
3.53
3.52
3.52
3.51
3.45
3.45
3.44
3.43
3.42
3.42
2.30
2.28
2.27
2.14
2.12
2.09
1.72
1.71
1.70
1.58
1.57
1.43
1.22
0.94
0.93
0.92
0.90
0.89
^{1}H
CDCl$_3$
600MHz
1.85
2.00
1.04
1.06
1.04
3.99
2.97
1.25
1.70
6.04
10.5 10.0 9.5 9.0 8.5 8.0 7.5 7.0 6.5 6.0 5.5 5.0 4.5 4.0 3.5 3.0 2.5 2.0 1.5 1.0 0.5 0.0 -0.5 -1.0
f1 (ppm)

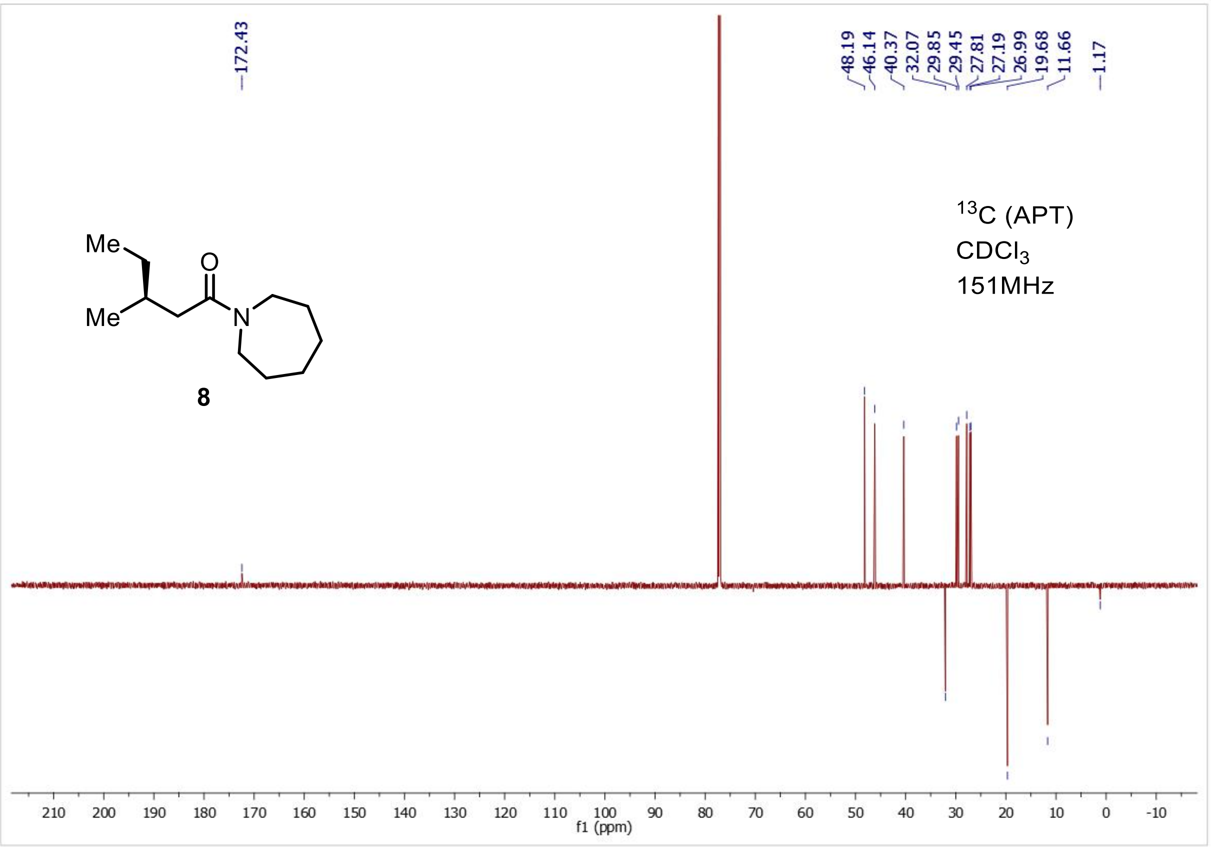

172.43
48.19
46.14
40.37
32.07
29.85
29.45
27.81
27.19
26.99
19.68
11.66
1.17
Me
Me
O
N
8
^{13}C (APT)
CDCl$_3$
151MHz
210 200 190 180 170 160 150 140 130 120 110 100 90 80 70 60 50 40 30 20 10 0 -10
f1 (ppm)

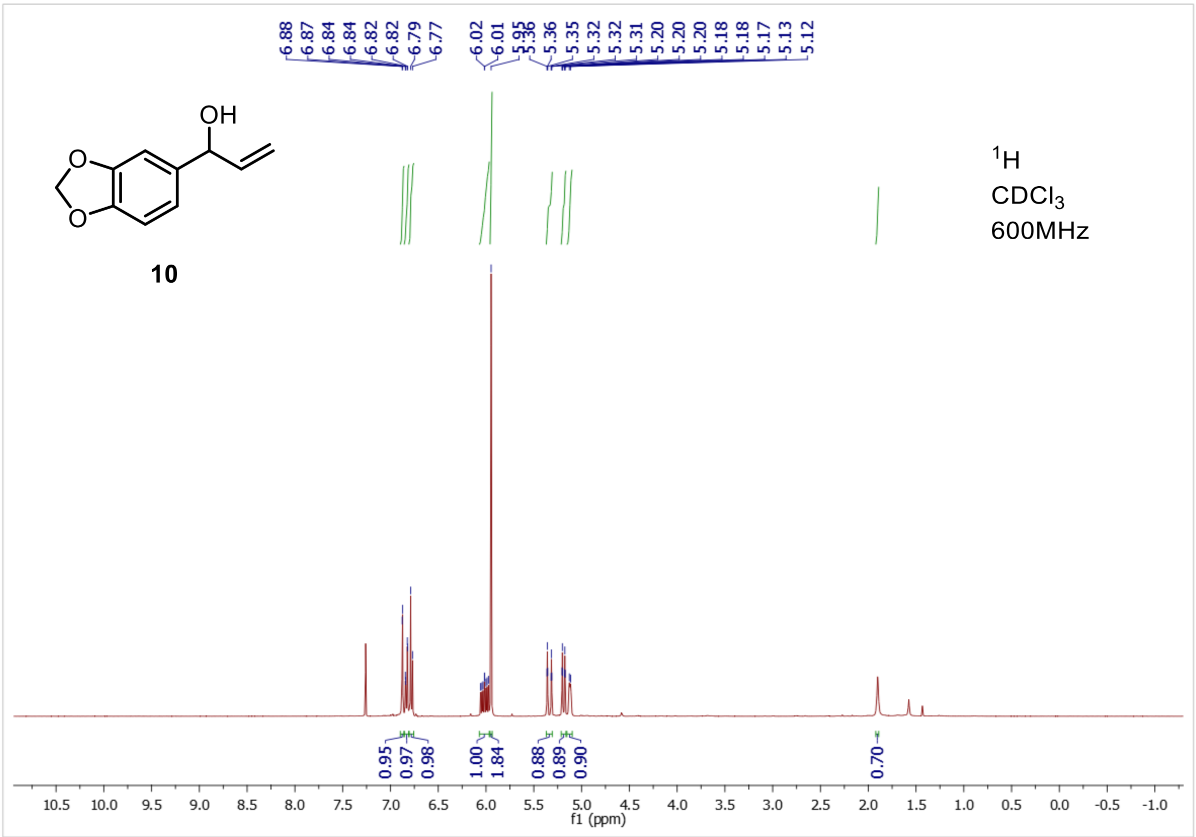
OH
O
O
10
1H
CDCl3
600MHz
f1 (ppm)

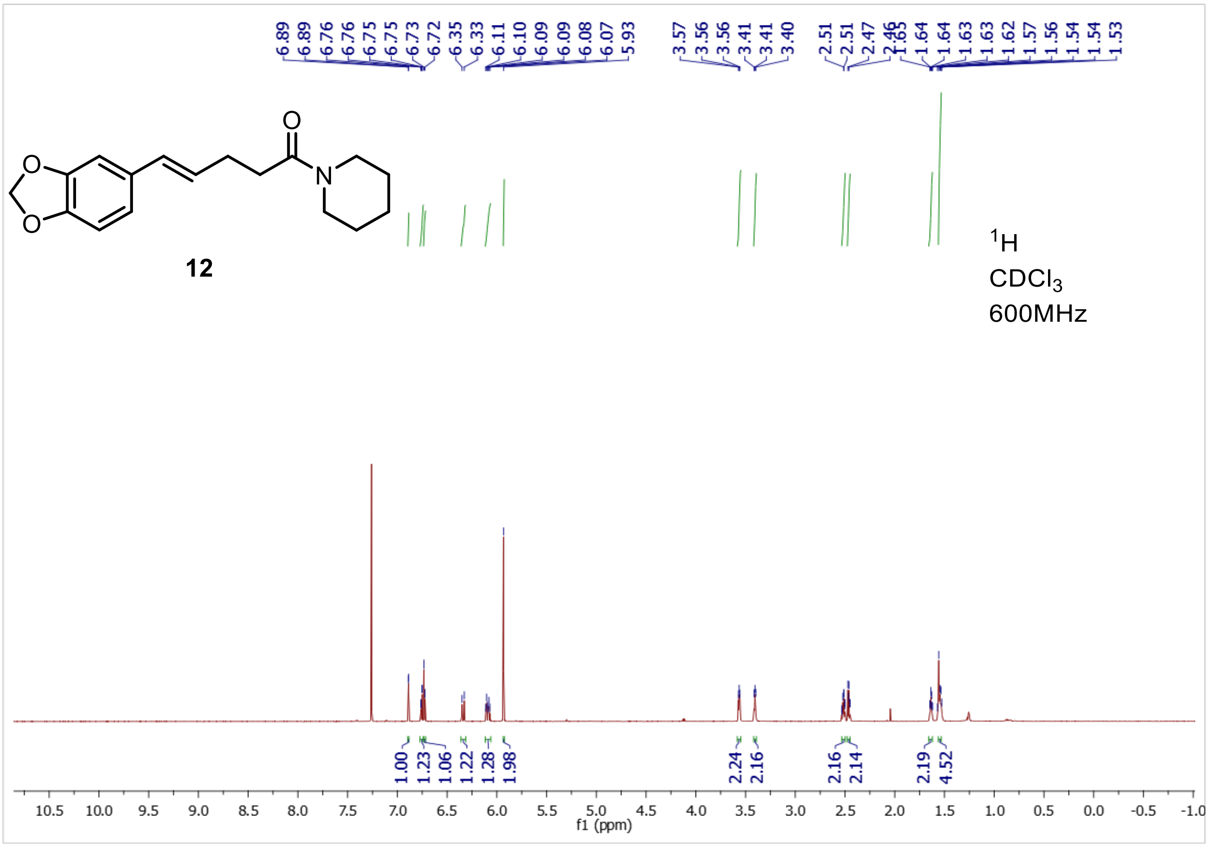
O
N
O
O
12
1H
CDCl3
600MHz
f1 (ppm)

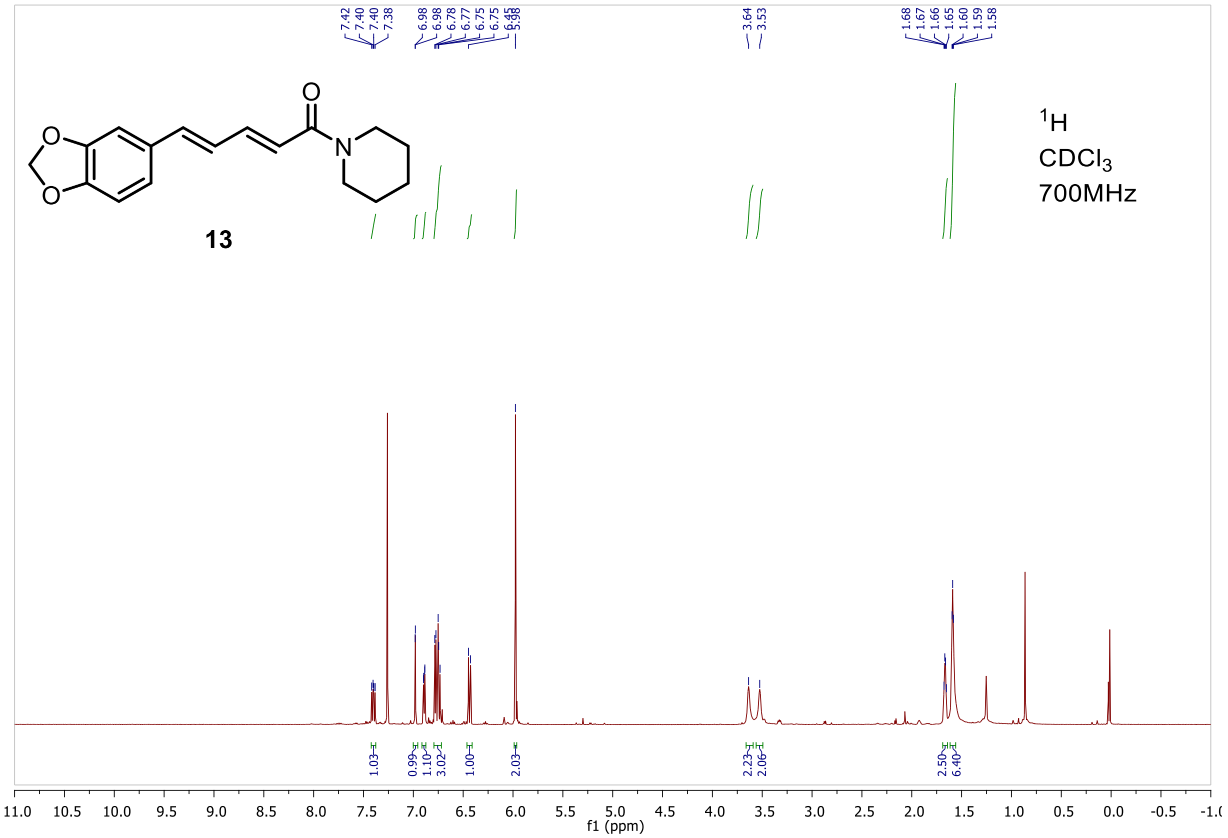
13
1H
CDCl3
700MHz
7.42
7.40
7.40
7.38
6.98
6.98
6.78
6.77
6.75
6.75
6.45
5.98
3.64
3.53
1.68
1.67
1.66
1.65
1.60
1.59
1.58
1.03
0.99
1.10
3.02
1.00
2.03
2.23
2.06
2.50
6.40
11.0 10.5 10.0 9.5 9.0 8.5 8.0 7.5 7.0 6.5 6.0 5.5 5.0 4.5 4.0 3.5 3.0 2.5 2.0 1.5 1.0 0.5 0.0 -0.5
f1 (ppm)

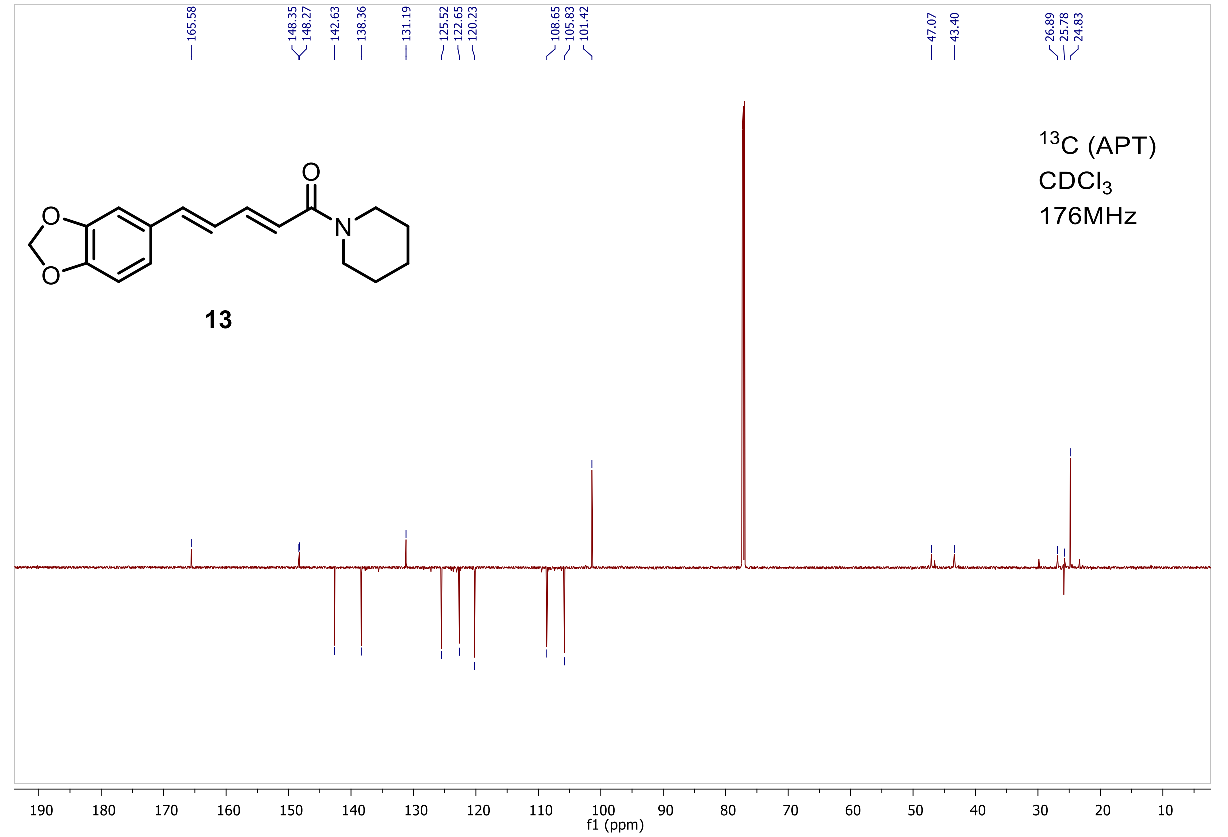
13
13C (APT)
CDCl3
176MHz
165.58
148.35
148.27
142.63
138.36
131.19
125.52
122.65
120.23
108.65
105.83
101.42
47.07
43.40
26.89
25.78
24.83
190 180 170 160 150 140 130 120 110 100 90 80 70 60 50 40 30 20 10
f1 (ppm)
